# Discovery and SAR analysis of phenylbenzo[d][1,3]dioxole-based proprotein convertase subtilisin/kexin type 9 inhibitors

**DOI:** 10.1080/14756366.2022.2101645

**Published:** 2022-07-19

**Authors:** Fahui Li, Lihui Zhang, Jinhong Feng, Lei Zhang

**Affiliations:** a Department of Medicinal Chemistry, School of Pharmacy, Weifang Medical University, Weifang, China; b School of Stomatology, Weifang Medical University, Weifang, China; c Shandong Analysis and Test Center, Qilu University of Technology (Shandong Academy of Sciences), Jinan, China

**Keywords:** PCSK9, LDLR, protein-protein interaction, LDL, small molecule

## Abstract

Proprotein convertase subtilisin/kexin type 9 (PCSK9) has emerged as a novel therapeutic target for the development of cholesterol-lowering drugs. In the discovery of PCSK9/LDLR (low-density lipoprotein receptor) protein-protein interaction (PPI) impairing small molecules, a total of 47 phenylbenzo[d][1,3] dioxole-based compounds were designed and synthesised. The result revealed that the 4-chlorobenzyl substitution in the amino group is important for the PPI disrupting activity. In the hepatocyte-based functional tests, active compounds such as **A12**, **B1**, **B3**, **B4** and **B14**, restored the LDLR levels on the surface of hepatic HepG2 cells and increased extracellular LDL uptake in the presence of PCSK9. It is notable that molecule **B14** exhibited good performance in all the evaluations. Collectively, novel structures targeting PCSK9/LDLR PPI have been developed with hypolipidemic potential. Further structural modification of derived active compounds is promising in the discovery of lead compounds with improved activity for the treatment of hyperlipidaemia-related disorders.

## Introduction

Lipid disorder conditions are common risk factors for cardiovascular diseases, which may occur with various factors, such as high fat diet^1^ or environmental pollutants^2^. Among the lipid disorder conditions, high blood level of low-density lipoprotein cholesterol (LDL-C) has been revealed to be a key risk factor for cardiovascular conditions such as adverse atherosclerotic cardiovascular events^3^. Low-density lipoprotein receptors (LDLRs) are responsible for the uptake of cholesterol-carrying lipoprotein particles into cells and are critical in regulating the amount of LDL-C in blood^4^. LDLRs on the surface of liver cells are of particular importance for the clearance of blood LDL-C due to their primary role in removing excess cholesterol from the body. The amount of hepatic LDLR proteins is mainly regulated by proprotein convertase subtilisin/kexin type 9 (PCSK9), a proprotein convertase family protease. It is identified that LDLR is degraded along with the complexed LDL particle in lysosomes by binding to PCSK9^5^. Thus, PCSK9 is closely related to dyslipidemia resulting from decreased LDLR levels and the consequent increased circulating LDL-C.

It was reported that PCSK9 binds to the EGF(A) domain of LDLR at the surface of liver cells^6^. Impairing the PCSK9/LDLR protein-protein interaction (PPI) has been employed to improve the hepatic LDLR population and decrease circulating LDL-C^7^. In 2015, two PCSK9 monoclonal antibodies (mAbs), alirocumab^8^ and evolocumab^9^, were approved by the FDA of the United States for the treatment of hyperlipidaemia. The PCSK9 mAbs exhibited remarkable clinical benefit without obvious side effects compared with the statin therapy^10^^,^^11^. However, the wide application of PCSK9 mAbs is restricted by the expensive price and inconvenient intravenous administration.

**Figure 1.**
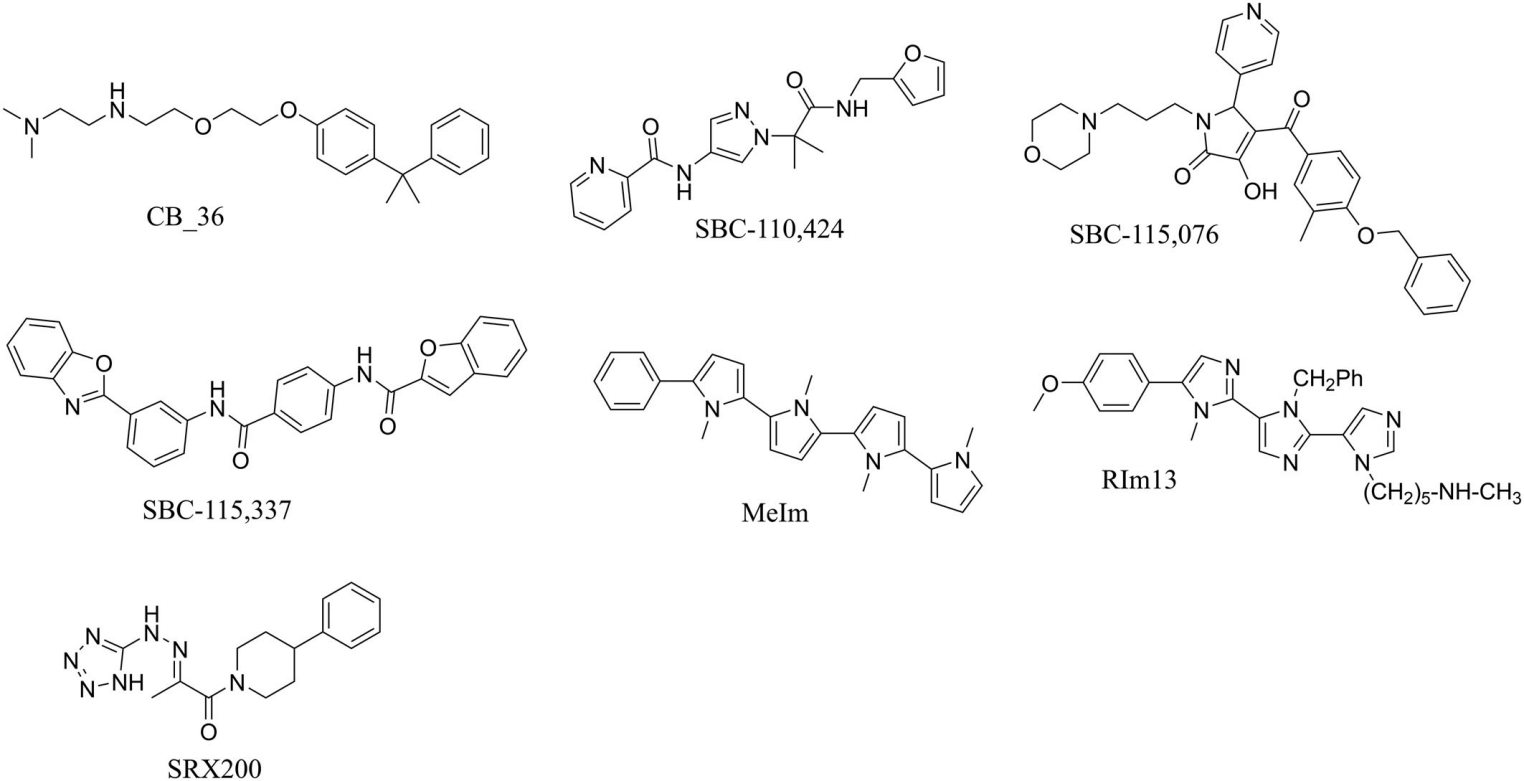
Structures of representative PCSK9/LDLR PPI inhibitors.

Development of small molecule PCSK9/LDLR PPI inhibitors is promising in the discovery of oral effective and low-cost drugs for hyperlipidemic therapy^12^^,^^13^. There are two kinds of small molecule PCSK9 inhibitors developed for blocking the function of PCSK9, PCSK9/LDLR PPI impairing molecules and PCSK9 synthesis/expression inhibitors^7^^,^^14^. Interfering with PCSK9/LDLR PPI has been explored by using human mAbs, peptides, peptidomimetics and small molecules^15–18^. By analysing the structure of PCSK9/LDLR binding domain, lead compounds, such as CB_36^19^, SBC-110,424 and SBC-115,076 (WO 2016/040305 A1) were derived by *in silico* virtual screening approach (Figure 1). Compound SBC-115,337 was developed by structural modification of previously derived molecules. The β-strand peptidomimetic MeIm^20^ and RIm13^21^ could effectively block the PCSK9/LDLR PPI by disturbing the β-strand mediated interactions. SRX200 (WO 2016/029037 A1) was considered to be an allosteric inhibitor by binding to an allosteric site of PCSK9.

**Figure 2.**
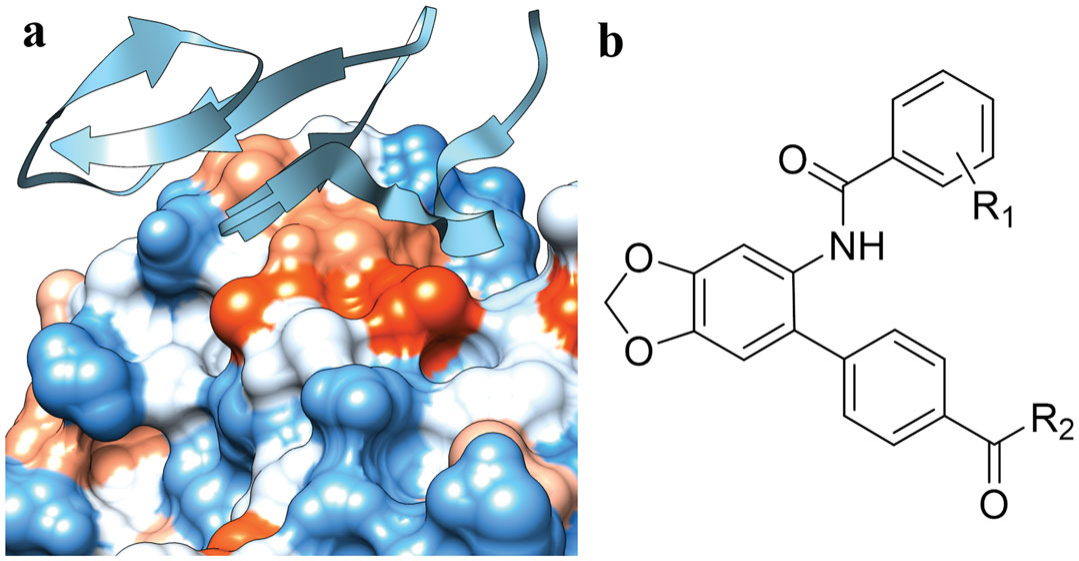
Design of PCSK9/LDLR PPI inhibitors. (a) Binding interface (PDB entry: 3GCX) of PCSK9 (hydrophobic surface) and EGFA domain of LDLR (ribbon); (b) structure of the designed PCSK9/LDLR PPI inhibitors.

In the current study, small molecule PCSK9 inhibitors were developed by targeting PCSK9/LDLR PPI. Due to the flat and featureless conformation of the PCSK9/LDLR binding domain (Figure 2), hydrophobic structures with large surface area were considered to be favourable for binding of inhibitors to PCSK9 and impairing the PPI. Because of the existence of fused rings and bi-aromatic rings in the structure of PCSK9/LDLR PPI inhibitors, 5-phenylbenzo[d][1,3]dioxole group was selected for occupying the PCSK9/LDLR PPI interface. In the current study, 4-(6-aminobenzo[d][1,3]dioxol-5-yl)benzoic acid was utilised as the core structure for the design of novel molecules to impair the PCSK9/LDLR PPI. Different substitutions were introduced to the carboxyl- and amino-group for the SAR analysis. The activities of the derived compounds were evaluated in the PCSK9/LDLR PPI inhibitory assay, hepatic cell-based LDLR expression test and LDL uptake investigation.

## Chemistry

**Scheme 1.**
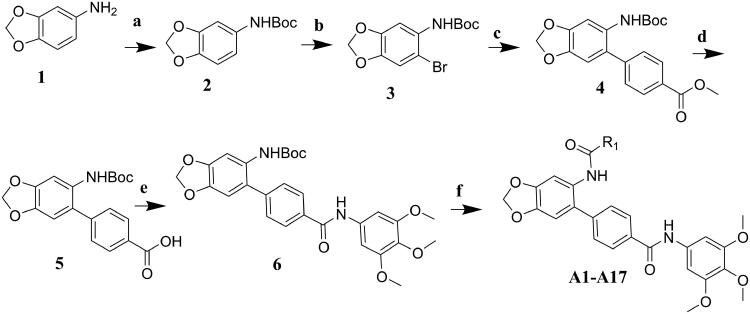
Reagents and conditions: (a) Boc_2_O, ACN, ice-bath; (b) NBS, ACN, rt; (c) K_2_CO_3_, trans-dichlorobis(triphenyl-phosphine)palladium(II), 1,4-dioxane, H_2_O, reflux; (d) 3 mol/l NaOH, MeOH, 40 °C; (e) TBTU, Et_3_N, DCM, ice-bath; (f) DCM, TFA; TBTU, Et_3_N, DCM, ice-bath.

**Scheme 2.**
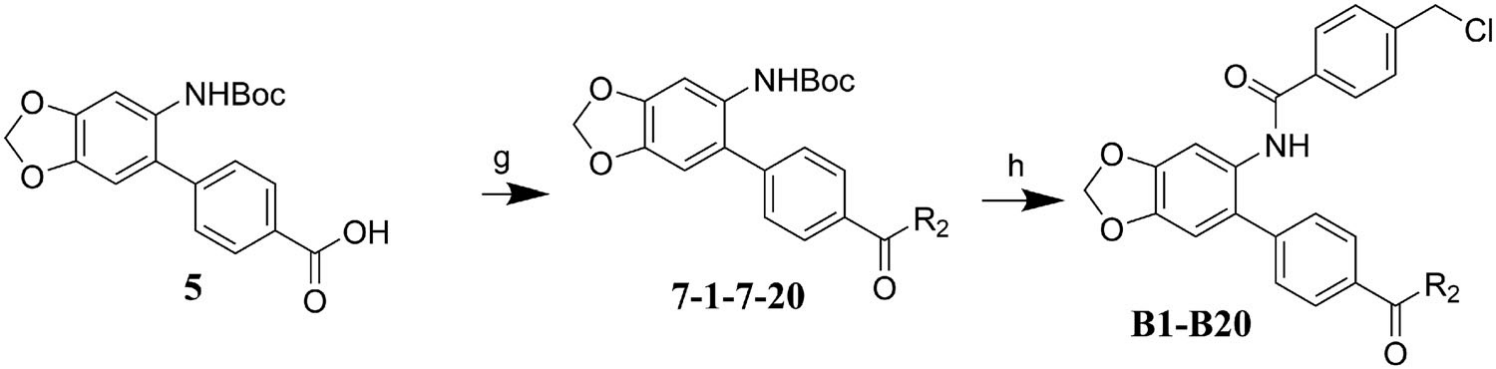
Reagents and conditions: (g) TBTU, Et_3_N, DCM, ice-bath; (h) DCM, TFA; TBTU, Et_3_N, DCM, ice-bath.

**Scheme 3.**
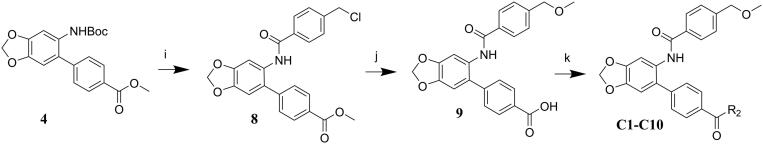
Reagents and conditions: (i) DCM, TFA; TBTU, Et_3_N, DCM, ice-bath; (j) 3 mol/l NaOH, MeOH, 40 °C; (k) TBTU, Et_3_N, DCM, ice-bath.

The target molecules were synthesised as described in Schemes 1–3. The commercially available benzo[d][1,3]dioxol-5-amine (**1**) was used as the starting material for the compound synthesis. Firstly, the amino group of **1** was protected by Boc group for bromine substitution. Then, the synthesised intermediate **3** was condensed with 4-(Methoxycarbonyl)benzeneboronic acid via Suzuki coupling to afford the key intermediate **4**. De-esterification under alkaline condition allowed the introduction of various substituted benzenamines and piperazines to the carboxy group. Substituted benzoic acids were introduced by deprotection of amino group and condensation reactions. Target molecules of the A series were synthesised by 3,4,5-trimethoxyaniline substitution in the carboxyl group and introduction of different benzoic acids to the amino group (Scheme 1). In the B series compounds, different amines were firstly introduced to the carboxyl group, then 4-(chloromethyl)benzoic acid substitution was performed on the amino group (Scheme 2). The C series compounds were derived by carboxyl substitution, demethylation and alcoholysis, and the final amino condensation (Scheme 3).

## Results and discussions

### PCSK9/LDLR PPI inhibitory screening

**Figure 3.**
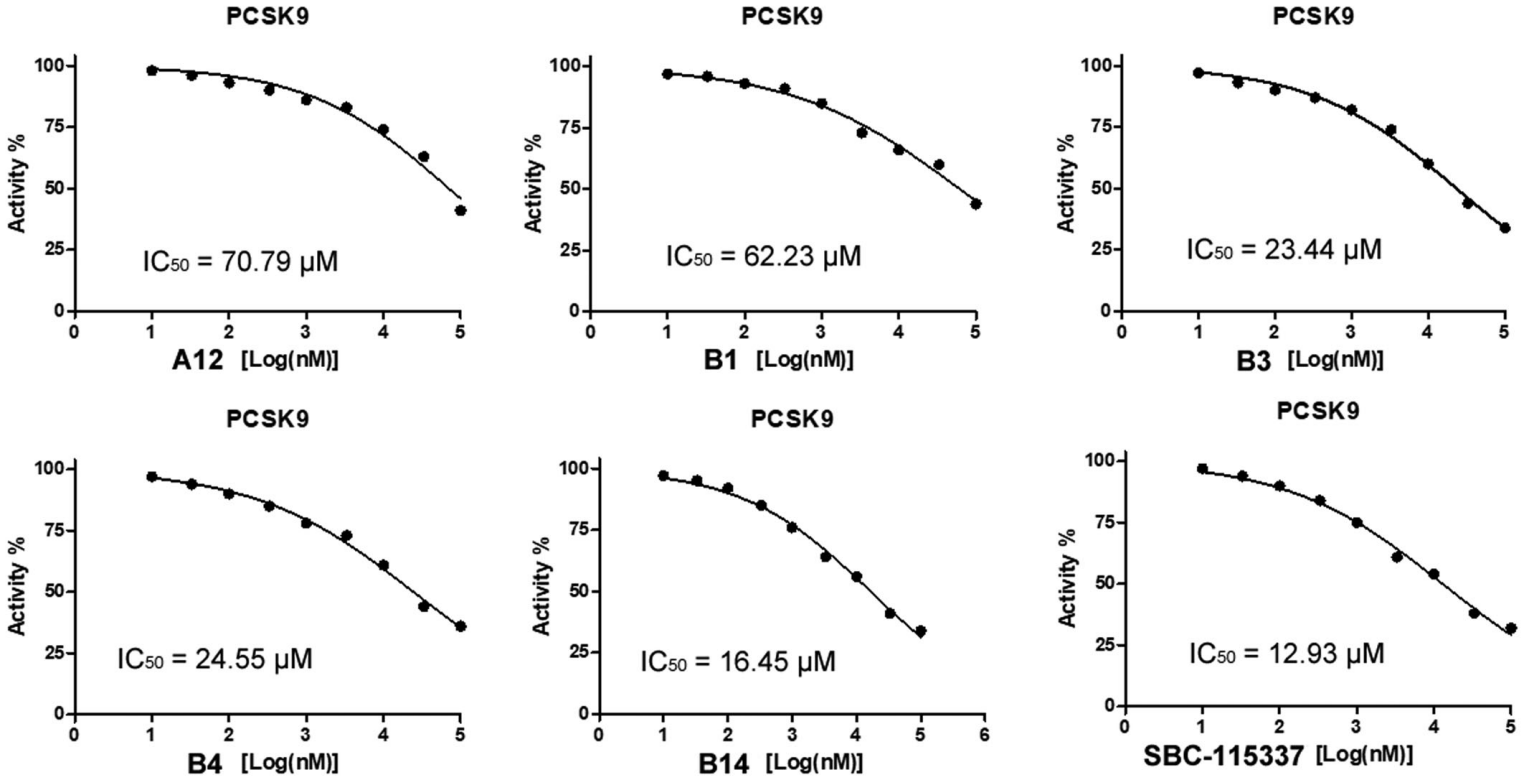
IC_50_ plot of representative molecules in the inhibition of PCSK9/LDLR PPI.

The PCSK9/LDLR time-resolved fluorescence resonance energy transfer (TR-FRET) assay was performed to evaluate the activity of synthesised compounds in inhibition of PCSK9-LDLR binding. The A series compounds were firstly synthesised for the PCSK9/LDLR PPI inhibitory test. The results revealed that the 4-chlorobenzyl substituted compound **A12** has the highest inhibitory activity with an inhibitory rate of 45.1% at the concentration of 10 µM (Table 1). Therefore, a group of **A12** derivatives (B series) were synthesised with 4-chlorobenzyl substituted in the amino group and various substitutions introduced to the carboxylic group. Among the B series compounds, several molecules exhibited improved inhibitory activity compared with **A12**, such as **B1**, **B3**, **B4** and **B14** (Table 2). To confirm whether the 4-chloromethyl group is essential for the PPI inhibitory activity, a new series of compounds (C series) were synthesised with the 4-chloromethyl group replaced by 4-methoxymethyl moiety. The inhibitory activity was observed, most likely resulting from the 4-methoxymethyl substitution (Table 3). The dose-dependent IC_50_ calculation revealed that molecule **B14** had high PCSK9/LDLR PPI inhibitory potential compared with SBC-115337 (Figure 3). These results suggested that molecules with 4-chlorobenzyl substitution had the potency in impairing PCSK9/LDLR PPI, indicating that the 4-chloromethyl group in the active compounds is essential for the inhibitory activity.

**Table 1.** Structure and activities of A series compounds. 
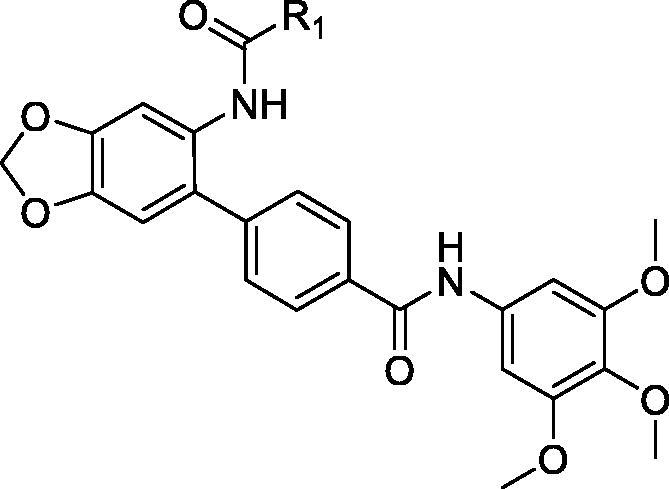

Componds	R_1_	PCSK9^a^	HepG2^a^
A1	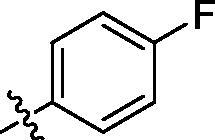	27.2 ± 1.85	12.48 ± 1.06
A2	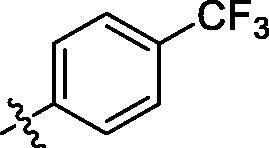	23.1 ± 2.17	16.37 ± 1.55
A3	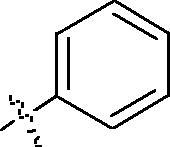	5.06 ± 0.09	8.78 ± 0.37
A4	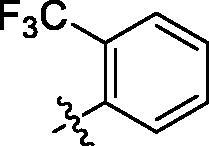	5.28 ± 0.40	7.93 ± 1.04
A5	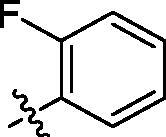	4.77 ± 0.31	5.96 ± 0.66
A6	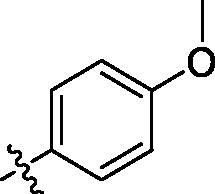	5.92 ± 0.52	5.90 ± 0.42
A7	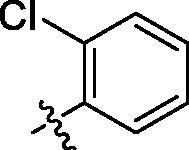	5.16 ± 0.31	12.43 ± 1.07
A8	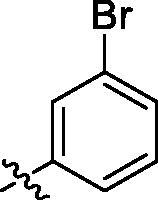	5.88 ± 0.12	3.66 ± 0.24
A9	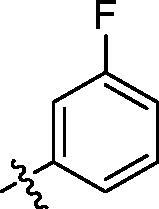	22.5 ± 1.36	11.71 ± 0.88
A10	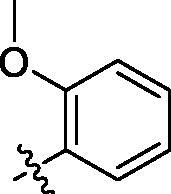	3.05 ± 0.05	4.27 ± 0.16
A11	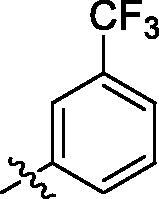	35.2 ± 3.15	7.97 ± 0.08
A12	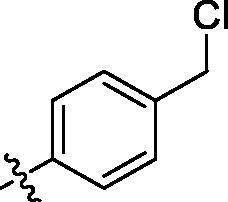	45.1 ± 0.12	7.85 ± 0.25
A13	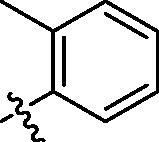	7.65 ± 0.01	5.89 ± 0.27
A14	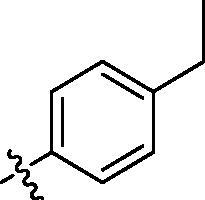	7.83 ± 0.26	5.21 ± 0.16
A15	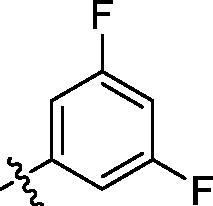	4.76 ± 0.37	4.63 ± 0.22
A16	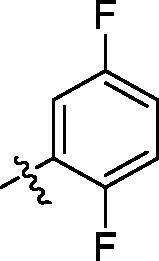	4.22 ± 0.25	3.33 ± 0.23
A17	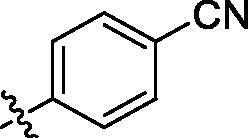	7.91 ± 0.71	3.84 ± 0.11
PCSK9 neutralising antibody	–	IC_50_ = 7.8 nM	ND
SAHA	–	ND	89.2 ± 1.76

^a^Illustrated as percentage inhibitory rate at concentration of 10 µM, and each value is the mean of three experiments. ND: Not determined.

**Table 2.** Structure and activities of B series compounds. 
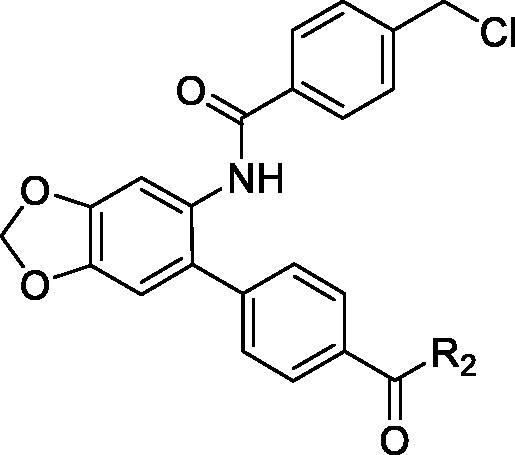

Componds	R_2_	PCSK9^a^	HepG2^a^
B1	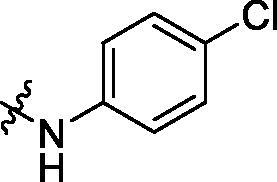	54.3 ± 1.54	9.97 ± 0.39
B2	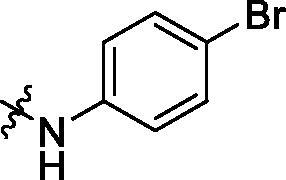	25.5 ± 2.22	15.64 ± 1.24
B3	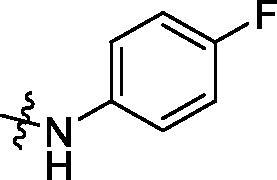	46.3 ± 2.77	8.09 ± 0.76
B4	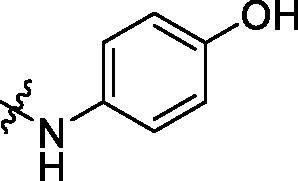	45.2 ± 2.85	5.42 ± 0.38
B5	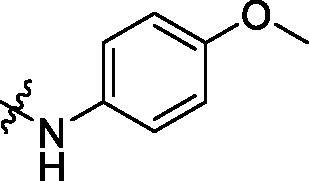	6.18 ± 0.08	7.22 ± 0.15
B6	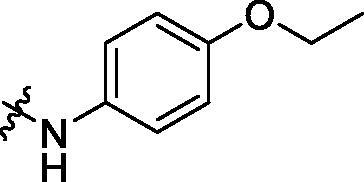	6.97 ± 0.43	7.04 ± 0.27
B7	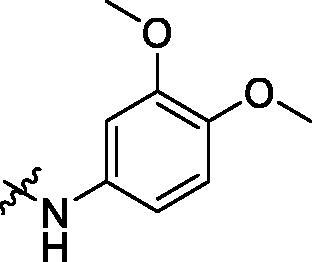	16.3 ± 0.62	5.45 ± 0.39
B8	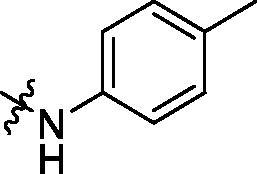	3.15 ± 0.02	3.35 ± 0.16
B9	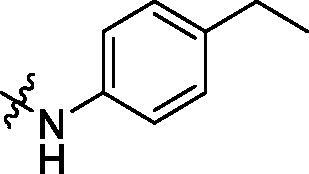	3.36 ± 0.23	6.36 ± 0.42
B10	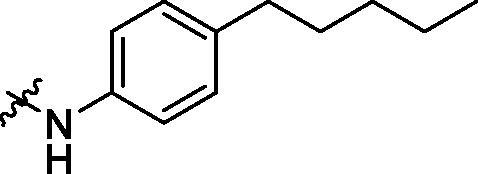	5.87 ± 0.01	2.40 ± 0.19
B11	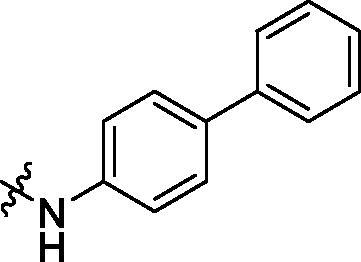	6.92 ± 0.53	3.26 ± 0.22
B12	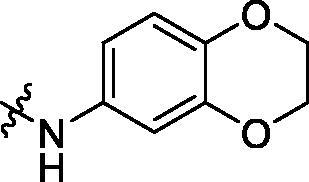	5.16 ± 0.20	5.76 ± 0.24
B13	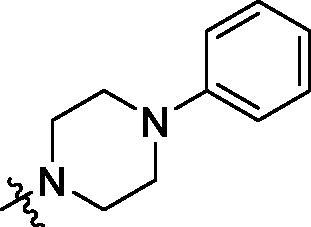	5.64 ± 0.04	3.21 ± 0.26
B14	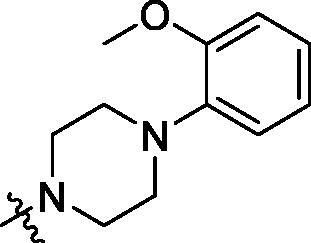	64.6 ± 1.54	5.71 ± 0.54
B15	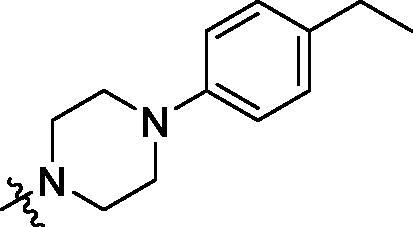	7.22 ± 0.71	8.91 ± 0.62
B16	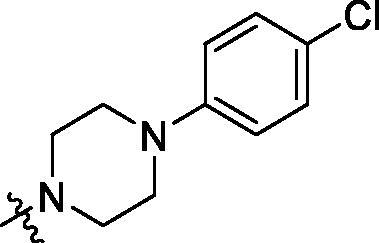	7.94 ± 0.03	10.25 ± 0.91
B17	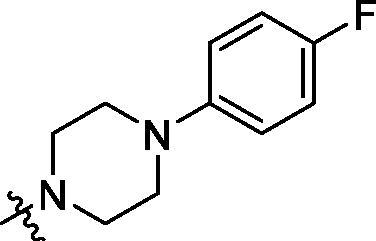	4.07 ± 0.25	16.43 ± 1.18
B18	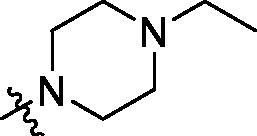	5.66 ± 0.02	6.76 ± 0.26
B19	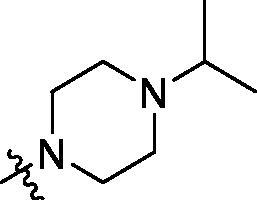	5.82 ± 0.31	8.38 ± 0.81
B20	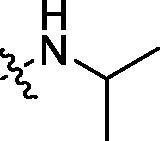	3.15 ± 0.13	9.55 ± 0.43
PCSK9 neutralising antibody	–	IC_50_ = 7.8 nM	ND
SAHA	–	ND	89.2 ± 1.76

^a^Illustrated as percentage inhibitory rate at concentration of 10 µM, and each value is the mean of three experiments. ND: Not determined.

**Table 3.** Structure and activities of C series compounds. 
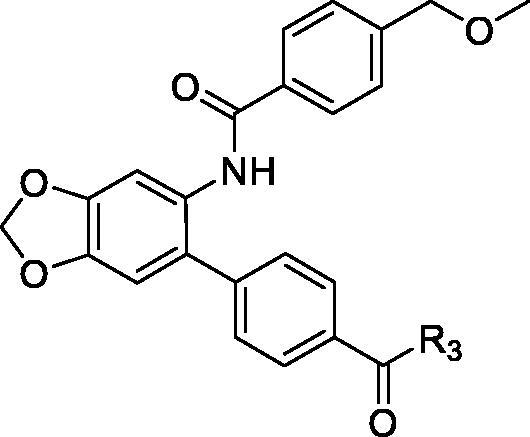

Componds	R_3_	PCSK9^a^	HepG2^a^
C1	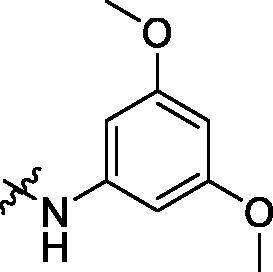	21.6 ± 1.67	6.21 ± 0.22
C2	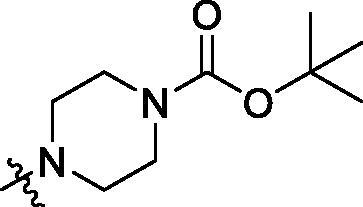	17.8 ± 1.24	13.52 ± 1.13
C3	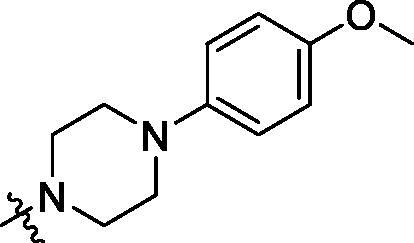	25.3 ± 2.11	6.62 ± 0.45
C4	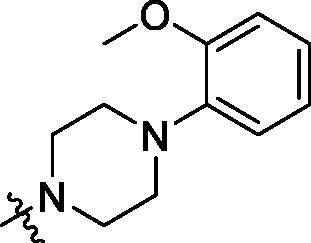	7.55 ± 0.71	6.77 ± 0.62
C5	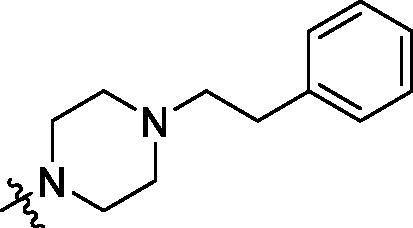	5.92 ± 0.32	5.63 ± 0.37
C6	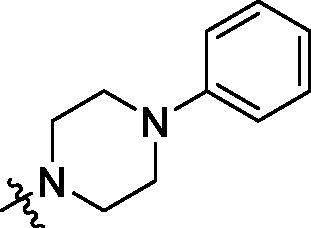	3.39 ± 1.67	6.15 ± 0.26
C7	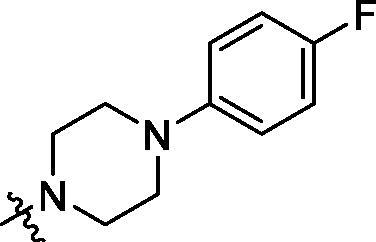	33.4 ± 1.52	12.85 ± 1.07
C8	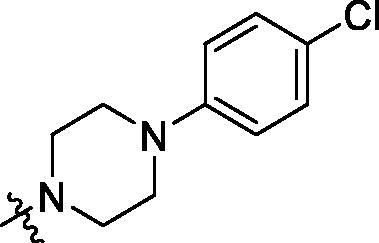	25.7 ± 1.20	14.54 ± 1.22
C9	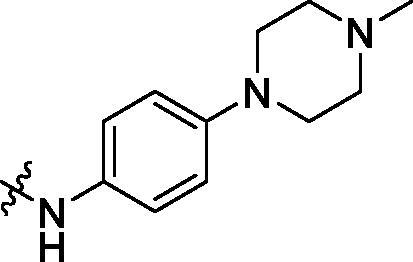	3.75 ± 0.33	6.22 ± 0.24
C10	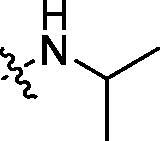	5.48 ± 0.20	6.76 ± 0.18
PCSK9 neutralising antibody	–	IC_50_ = 7.8 nM	ND
SAHA	–	ND	89.2 ± 1.76

^a^ Illustrated as percentage inhibitory rate at concentration of 10 µM, and each value is the mean of three experiments. ND: Not determined.

To acquire clues for the structural modification of the derived compounds, SAR analysis was performed based on the PCSK9/LDLR PPI inhibitory activities. Among the A series compounds (Table 1), fluorine and trifluoromethyl substitutions in the meta and para positions of phenyl ring (in R group) are favoured for the inhibitory activities, such as **A1**, **A2**, **A9**, and **A11**. While the difluoro substitutions did not significantly enhance the activities, such as **A15** and **A16**. Chlorine, methoxy and alkyl groups substituted in the phenyl ring did not improve the PPI inhibitory activities. Molecule **A12** with chloromethyl substitution in the R group exhibited the best inhibitory activity in the A series compounds.

With the 4-chloromethyl-benzoyl group substituted in the amino group, the B series compounds were synthesised by the introduction of different substitutions to the carboxyl group. From **B1** to **B12**, halogen and hydroxyl substitutions in the para position of phenyl ring increased the PPI inhibitory activities, such as **B1**, **B2**, **B3** and **B4** (Table 2). The alkoxyl-substituted compounds, such as **B5**, **B6**, **B7** and **B12**, did not show improved inhibitory activities. Alkyl and phenyl groups substituted in the para position of R moiety did not exhibit positive effects on enhancing the inhibitory activity. Among the piperazine containing R groups, methoxy substitution in the ortho position of phenyl ring obviously increased the PCSK9/LDLR PPI inhibitory activity.

Activity decrease caused by the replacement of 4-chloromethyl group with 4-methoxymethyl in the C series compounds revealed the importance of 4-chloromethyl substitution for the inhibitory potency of the compounds (Table 3). Among the C series molecules, methoxy, fluorine and chlorine substituted the para position of phenylpiperazine ring, such as **C3**, **C7** and **C8**. The methoxy group substituted in the ortho position decreased the inhibitory activity. Molecule **C1** with 3,5-dimethoxyphenylamino R group also exhibited potency in the inhibition of PCSK9/LDLR PPI.

### LDLR expression assay

**Figure 4.**
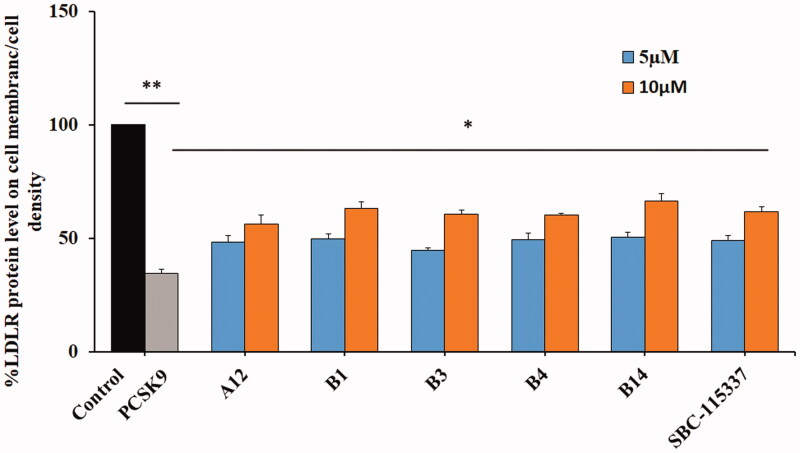
Representative molecules increase the LDLR protein level on the HepG2 cell surface in the presence of PCSK9. PCSK9 treatment obviously decreases the expression level of LDLR (**p < 0.01), and LDLR degradation mediated by PCSK9 is prevented by the derived compounds with a dose dependent manner comparing with SBC-115337 (*p < 0.05, the PCSK9 only control was used as a comparator).

LDLR is degraded in lysosomes by binding to PCSK9. Thus, inhibition of PCSK9/LDLR PPI could increase the LDLR level on the surface of hepatocytes. In the current study, human hepatic HepG2 cells were selected for the investigation of the active compounds for LDLR expression. SBC-115337, developed by Shifa Biomedical Corporation, as a potent PCSK9/LDLR PPI inhibitor was utilised as the positive control. All the synthesised compounds were evaluated to be safe with low inhibitory ratios at the dose of 10 µM in the MTT assay (Tables 1–3). Then, in cell western (ICW) assay was performed to detect the LDLR level on the surface of HepG2 cells. The results showed that the presence of PCSK9 significantly decreased the LDLR level compared with the control (Figure 4). The tested compound **A12**, **B1**, **B3**, **B4** and **B14** restored the LDLR protein level in a dose dependent manner compared with the positive control SBC-115337. Remarkably, the proportion of LDLR increased from 34.33% in cells treated with PCSK9 alone to 50.55% and 66.51% in cells treated with the molecule **B14** at the concentrations of 5 µM and 10 µM, respectively. These results indicated that these representative compounds are effective in improving the LDLR levels induced by inhibition of PCSK9/LDLR PPI.

### LDL uptake test

**Figure 5.**
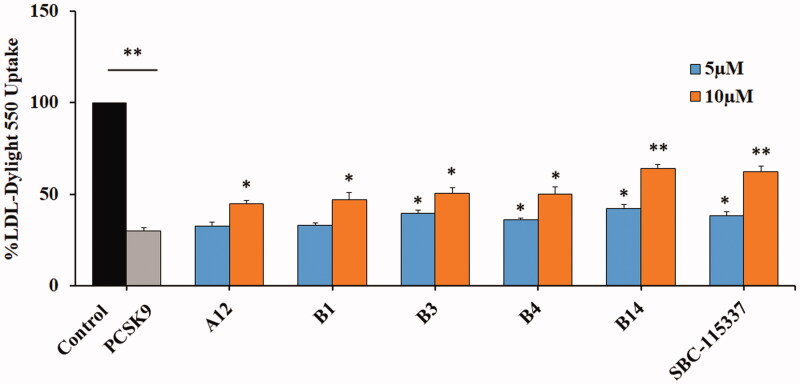
Representative molecules increase the LDL uptake ability of HepG2 cell in the presence of PCSK9. Compared with the control group, PCSK9 treatment significantly decreases the LDL uptake of HepG2 cells (**p < 0.01). The synthesised compounds restore the ability of HepG2 cells to uptake LDL with a dose dependent manner comparing with SBC-115337 (*p < 0.05, **p < 0.01, the PCSK9 only control was used as a comparator).

Extracellular LDL uptake assay was performed to evaluate the functional effects of PCSK9/LDLR PPI inhibition. The effects of active compounds in improving the capacity of HepG2 cells to uptake the fluorescent LDL were investigated using SBC-115337 as the positive control. It was found that PCSK9 treatment reduces the LDL uptake of HepG2 cells compared with the untreated cells in the control group (Figure 5). Treatment of hepatic cells with tested molecules was observed to restore the fluorescent LDL uptake in the presence of PCSK9. The tested compound **A12**, **B1**, **B3**, **B4** and **B14** strengthened the ability of HepG2 cells to absorb LDL from 29.82% of the PCSK9 treatment group up to 32.78%, 33.29%, 39.58%, 36.24% and 42.41% at the doses of 5 µM, and 44.80%, 47.15%, 50.67%, 49.94 and 64.27% at the doses of 10 µM, respectively. These results demonstrated that the derived molecules restore the LDL uptake of HepG2 cells in a concentration dependent manner. Notably, molecule **B14** showed the best performance in the extracellular LDL uptake test compared with the positive control SBC-115337. The restoration of LDL uptake is suggested to be the result of disrupted PCSK9/LDLR PPI induced by the tested compounds.

### Binding pattern analysis

**Figure 6.**
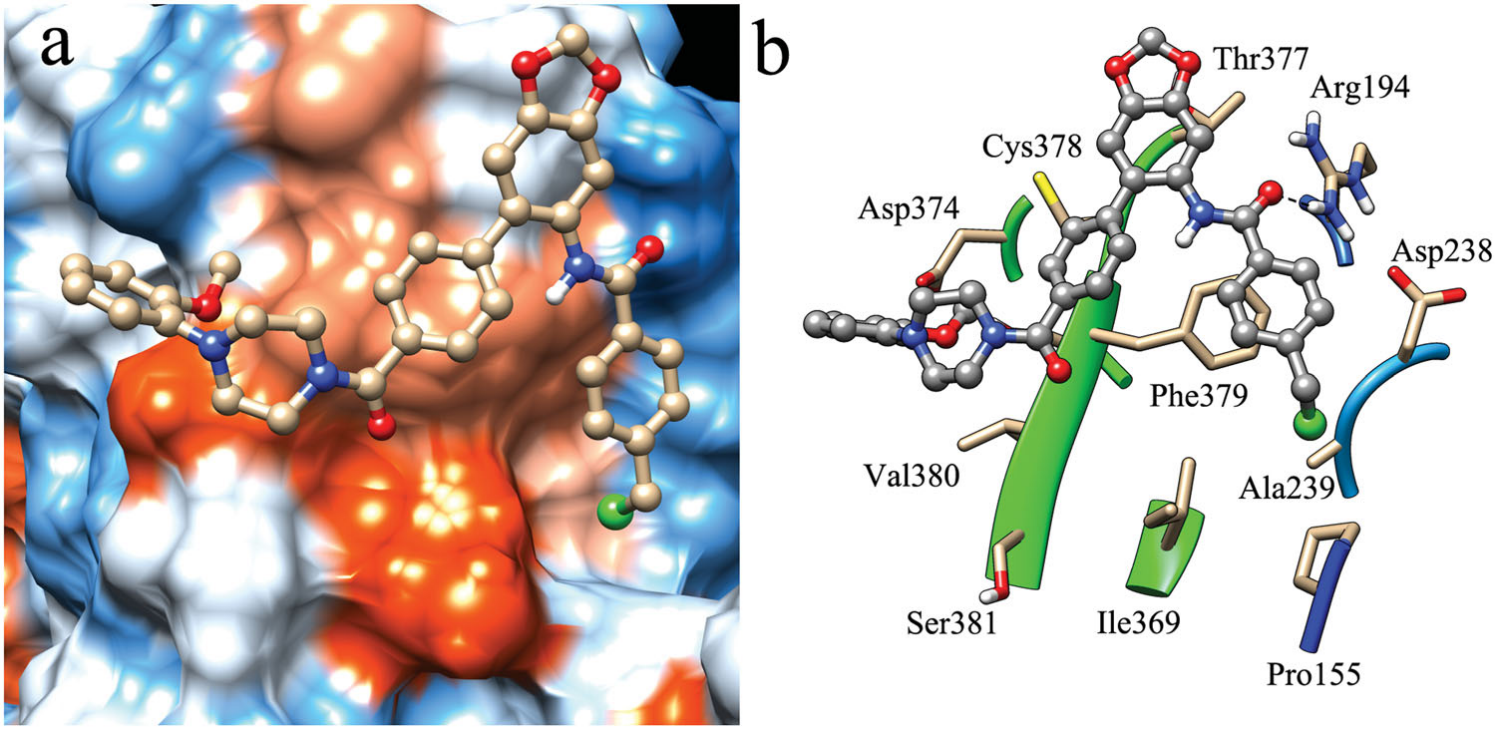
Molecular docking result of molecule **B14** in the LDLR binding domain of PCSK9 (PDB entry: 3GCX). a: surface representation of molecule **B14** in the binding site; b: binding pattern of molecule **B14** with surrounding residues.

Molecular docking was performed to predict the binding mode of the active molecule **B14** in the LDLR binding domain of PCSK9. As illustrated in Figure 6(a), there was no obvious binding pocket in the PCSK9/LDLR PPI interface. Hydrophobic interactions play an important role in the binding of molecule **B14** to the LDLR binding site. It was revealed that Phe379 is a key residue with Pi-Pi stacking interactions with phenyl rings of molecule **B14** (Figure 6(b)). The surrounding residues, such as Pro155, Ala239, Ile369, Thr377, Cys378 and Val380 are also significant in the hydrophobic interactions formed between molecule **B14** and PCSK9. Hydrogen bond interaction generated between NH of Arg194 and CO of **B14** also makes contributions to the ligand-receptor binding. These results indicated that the flat structure of molecule **B14** is suitable for matching the surface of PCSK9/LDLR PPI. Further structural modification of molecule **B14** could be performed by increasing hydrogen bond interactions with surrounding polar residues.

## Conclusion

Hyperlipidaemia is a major risk factor for cardiovascular diseases, and PCSK9 inhibition has emerged as a novel cholesterol-lowering therapy. In order to discover small molecules that could disrupt PCSK9/LDLR PPI, a total of 47 phenylbenzo[d][1,3]dioxole containing compounds were designed and synthesised. The derived compounds were tested in the molecular PCSK9/LDLR PPI inhibitory screening, LDLR expression on the surface of hepatocytes, and hepatic-cell-based extracellular LDL uptake. Several compounds, such as **A12**, **B1**, **B3**, **B4** and **B14** exhibited potency in impairing the PCSK9/LDLR PPI. SAR analysis revealed the importance of 4-chlorobenzyl substitution in the amino group for inhibitory activity. In the LDLR expression and LDL uptake studies, the tested molecules restored both LDLR expression and fluorescent LDL uptake of HepG2 cells in the presence of PCSK9. Comparing with SBC-115337, molecule **B14** showed remarkable performance in the cell based functional tests. In summary, a potent lead compound (**B14**) was developed in the current study for the discovery of small molecule PCSK9 inhibitors that directly disrupt PCSK9/LDLR PPI. Further structural modification of the derived active compounds would be promising in the development of hypolipidemic small molecules with improved potency.

## Materials and methods

All chemicals were obtained from commercial suppliers and can be used without further refinement. All reactions were detected by TLC using 0.25 mm silica gel plate (60GF-254). UV light and ferric chloride were used to show TLC spots. ^1^H NMR and ^13 ^C NMR spectra were recorded on a Bruker DRX spectrometer at 500 MHz, using TMS as an internal standard. High-resolution mass spectra were recorded using a Thermo Scientific Q Exactive hybrid quadrupole-orbitrap mass spectrometer from Weifang Medical University.

Compounds **1–5** have been synthesised as described in our previous work^22^.

Methyl 4-(6-((tert-butoxycarbonyl)amino)benzo[d][1,3]dioxol-5-yl)benzoate(**4**). ^1^H NMR (400 MHz, DMSO-d_6_) *δ* 8.43 (s, 1H), 7.96 (d, *J* = 8.2 Hz, 2H), 7.48 (d, *J* = 8.1 Hz, 2H), 6.91 (s, 1H), 6.85 (s, 1H), 6.08 (s, 2H), 3.87 (s, 3H), 1.26 (s, 9H).

4-(6-((Tert-butoxycarbonyl)amino)benzo[d][1,3]dioxol-5-yl)benzoic acid (**5**). ^1^H NMR (400 MHz, DMSO-d_6_) *δ* 12.91 (s, 1H), 8.40 (s, 1H), 7.93 (d, *J* = 8.2 Hz, 2H), 7.44 (d, *J* = 8.0 Hz, 2H), 6.91 (s, 1H), 6.85 (s, 1H), 6.07 (s, 2H), 1.25 (s, 9H).

***Tert-butyl(6-(4-((3,4,5-trimethoxyphenyl)carbamoyl)phenyl)benzo[d][1,3]dioxol-5-yl)carbamate(6)***

To a solution of compound **5** (0.50 g, 2.12 mmol) in DCM (5 ml), Et_3_N (0.25 g, 2.54 mmol) and TBTU (0.81 g, 2.54 mmol) were sequentially added. After 20 min, 3,4,5-trimethoxyaniline (0.46 g, 2.54 mmol) and Et_3_N (0.25 g, 2.54 mmol) were added. The reaction was stirred at room temperature for 5 h. After the reaction, the solvent was evaporated *in vacuo*, and the residue was taken up in EtOAc (30 ml). The EtOAc solution was washed with saturated citric acid (3 × 30 ml), NaHCO_3_ (3 × 30 ml) and brine (3 × 30 ml), dried over MgSO_4_ and concentrated by evaporation *in vacuo*. The desired compound **6** (0.4 g, 57% yield) was derived by crystallisation in EtOAc as white powder. ^1^H NMR (400 MHz, DMSO) *δ* 10.13 (s, 1H), 8.39 (s, 1H), 7.99 (d, *J* = 8.4 Hz, 2H), 7.49 (d, *J* = 8.2 Hz, 2H), 7.28 (s, 2H), 6.93 (s, 1H), 6.88 (s, 1H), 6.08 (d, *J* = 3.7 Hz, 2H), 3.78 (s, 6H), 3.64 (s, 3H), 1.28 (s, 10H).

***4-Fluoro-N-(6-(4-((3,4,5-trimethoxyphenyl)carbamoyl)phenyl)benzo[d][1,3]dioxol-5-yl)benzamide (A1)***

Compound **6** (0.3 g, 0.57 mmol) was dissolved in dry DCM. After addition of TFA, the solvent was stirred at room temperature, monitored by TLC. Until the raw materials were completely consumed, the reagents were evaporated under vacuum and dissolved with a mixed solution of tetrahydrofuran and water (50:1) at 0 °C with addition of sodium bicarbonate. After stirred for 10 min, 4-fluorobenzoyl chloride (0.14 g, 0.92 mmol) was added, and the mixture was stirred at room temperature for 4 h. After that, the reagents were evaporated under vacuum and dissolved in EtOAc. The solvent was washed with saturated NaHCO_3_ (3 × 30 ml) and brine (3 × 30 ml), dried over MgSO_4_, and evaporated under vacuo. The desired compound **A1** (0.23 g, 74% yield) was derived by crystallisation in EtOAc as white powder. HRMS *m/z* [M + Na]^+^ calcd for C_30_H_25_FN_2_NaO_7_: 567.15435, found: 567.15021. ^1^H NMR (400 MHz, DMSO) *δ* 10.13 (s, 1H), 8.39 (s, 1H), 7.99 (d, *J* = 8.4 Hz, 2H), 7.49 (d, *J* = 8.2 Hz, 2H), 7.28 (s, 2H), 6.93 (s, 1H), 6.88 (s, 1H), 6.08 (d, *J* = 3.7 Hz, 2H), 3.78 (s, 6H), 3.64 (s, 3H), 1.28 (s, 10H). ^13 ^C NMR (101 MHz, DMSO) *δ* 165.25, 153.04, 147.49, 146.73, 135.75, 134.13, 133.45, 130.61 (d, *J* = 9.0 Hz), 129.13, 127.85, 115.90, 115.68, 109.82, 102.23, 98.47, 60.59, 56.18.

***4-(Trifluoromethyl)-N-(6-(4-((3,4,5-trimethoxyphenyl)carbamoyl)phenyl)benzo[d][1,3]dioxol-5-yl)benzamide(A2)***

Crystallised from EtOAc to give a white powder (0.25 g, 72%); HRMS *m/z* [M + Na]^+^ calcd for C_31_H_25_F_3_N_2_NaO_7_: 617.15116, found: 617.14728. ^1^H NMR (400 MHz, DMSO) *δ* 10.10 (d, *J* = 11.0 Hz, 2H), 7.96 (dd, *J* = 17.4, 8.3 Hz, 4H), 7.85 (d, *J* = 8.3 Hz, 2H), 7.55 (d, *J* = 8.4 Hz, 2H), 7.22 (s, 2H), 7.06 (d, *J* = 12.0 Hz, 2H), 6.14 (s, 2H), 3.75 (s, 6H), 3.63 (s, 3H). ^13 ^C NMR (101 MHz, DMSO) *δ* 165.19 (d, *J* = 13.6 Hz), 163.57, 161.14, 153.05, 147.52, 146.82, 142.83, 137.09 (d, *J* = 6.7 Hz), 135.75, 134.18, 133.50, 131.82, 131.06 (d, *J* = 8.0 Hz), 129.13, 128.86, 127.88, 124.12, 119.26–119.04 (m), 118.87 (d, *J* = 21.3 Hz), 114.85, 114.63, 109.81 (d, *J* = 6.9 Hz), 102.27, 98.53, 60.58, 60.23, 56.19, 21.22, 14.55.

***4-(6-Benzamidobenzo[d][1,3]dioxol-5-yl)-N-(3,4,5-trimethoxyphenyl)benzamide (A3)***

Crystallised from EtOAc to give a white powder (0.25 g, 72%); HRMS *m/z* [M + Na]^+^ calcd for C_30_H_26_N_2_NaO_7_: 543.16377, found: 543.15430. ^1^H NMR (400 MHz, DMSO) *δ* 10.09 (s, 1H), 9.85 (s, 1H), 7.94 (d, *J* = 8.5 Hz, 2H), 7.79 (d, *J* = 7.2 Hz, 2H), 7.57–7.50 (m, 3H), 7.45 (t, *J* = 7.4 Hz, 2H), 7.22 (s, 2H), 7.03 (d, *J* = 4.1 Hz, 2H), 6.13 (s, 2H), 3.75 (s, 6H), 3.63 (s, 3H). ^13 ^C NMR (101 MHz, DMSO) *δ* 165.28, 163.35, 157.28, 153.09, 147.42, 144.70, 142.06, 135.80, 134.26 (d, *J* = 8.1 Hz), 133.74, 131.52, 130.00 (d, *J* = 6.7 Hz), 128.41, 126.74, 121.87, 121.40, 112.66, 110.01, 104.88, 102.06, 98.45, 60.61, 56.19 (d, *J* = 7.0 Hz).

***2-(Trifluoromethyl)-N-(6-(4-((3,4,5-trimethoxyphenyl)carbamoyl)phenyl)benzo[d][1,3]dioxol-5-yl)benzamide(A4)***

Crystallised from EtOAc to give a white powder (0.23 g, 67%); HRMS *m/z* [M + Na]^+^ calcd for C_31_H_25_F_3_N_2_NaO_7_: 617.15116, found: 617.14728. ^1^H NMR (400 MHz, DMSO) *δ* 10.15 (s, 1H), 10.03 (s, 1H), 8.03 (d, *J* = 8.5 Hz, 2H), 7.79 (d, *J* = 7.7 Hz, 1H), 7.69 (dd, *J* = 25.4, 7.6 Hz, 2H), 7.57 (d, *J* = 8.4 Hz, 2H), 7.42 (d, *J* = 7.5 Hz, 1H), 7.27 (s, 2H), 6.14 (s, 2H), 3.78 (s, 6H), 3.64 (s, 3H). ^13 ^C NMR (101 MHz, DMSO) *δ* 165.31 (d, *J* = 7.1 Hz), 153.05, 147.54, 146.84, 142.82, 138.65, 135.76, 134.17, 133.54, 131.80, 129.11, 128.86 (d, *J* = 6.0 Hz), 127.89, 125.86, 109.79 (d, *J* = 15.3 Hz), 102.28, 98.52, 60.58, 56.19.

***2-Fluoro-N-(6-(4-((3,4,5-trimethoxyphenyl)carbamoyl)phenyl)benzo[d][1,3]dioxol-5-yl)benzamide(A5)***

Crystallised from EtOAc to give a white powder (0.25 g, 72%); HRMS *m/z* [M + Na]^+^ calcd for C_30_H_25_FN_2_NaO_7_: 567.15435, found: 567.15051. ^1^H NMR (400 MHz, DMSO) *δ* 10.14 (s, 1H), 9.77 (d, *J* = 2.0 Hz, 1H), 8.00 (d, *J* = 8.5 Hz, 2H), 7.54 (ddd, *J* = 9.0, 7.3, 5.4 Hz, 4H), 7.31–7.23 (m, 4H), 7.13 (s, 1H), 7.01 (s, 1H), 6.13 (s, 2H), 3.77 (s, 6H), 3.64 (s, 3H). ^13 ^C NMR (101 MHz, DMSO) *δ* 165.31, 163.70, 160.80, 158.32, 153.07, 147.48, 146.53, 142.65, 135.78, 134.19, 133.59, 133.03, 131.02, 130.39, 129.34, 128.73, 127.90, 124.91, 124.44 (d, *J* = 14.6 Hz), 116.73, 116.51, 109.91, 108.82, 102.26, 98.53, 60.60, 60.23, 56.21, 21.23, 14.55.

***4-Methoxy-N-(6-(4-((3,4,5-trimethoxyphenyl)carbamoyl)phenyl)benzo[d][1,3]dioxol-5-yl)benzamide(A6)***

Crystallised from EtOAc to give a white powder (0.25 g, 78%); HRMS *m/z* [M + Na]^+^ calcd for C_31_H_28_NaO_8_: 579.17434, found: 579.17035. ^1^H NMR (400 MHz, DMSO) *δ* 10.09 (s, 1H), 9.69 (s, 1H), 7.93 (d, *J* = 8.5 Hz, 2H), 7.79 (d, *J* = 8.8 Hz, 2H), 7.54 (d, *J* = 8.5 Hz, 2H), 7.23 (s, 2H), 7.02 (d, *J* = 1.7 Hz, 2H), 7.01–6.95 (m, 2H), 6.13 (s, 2H), 3.79 (s, 3H), 3.76 (s, 6H), 3.63 (s, 3H). ^13 ^C NMR (101 MHz, DMSO) *δ* 165.91, 165.27, 162.26, 153.05, 147.44, 146.55, 142.97, 135.76, 134.17, 133.39, 131.62, 129.85, 129.44, 129.17, 127.83, 126.88, 114.03, 109.83 (d, *J* = 13.4 Hz), 102.18, 98.52, 60.59, 56.19, 55.84.

***2-Chloro-N-(6-(4-((3,4,5-trimethoxyphenyl)carbamoyl)phenyl)benzo[d][1,3]dioxol-5-yl)benzamide(A7)***

Crystallised from EtOAc to give a white powder (0.26 g, 80%); HRMS *m/z* [M + Na]^+^ calcd for C_30_H_25_ClN_2_NaO_7_: 583.12480, found: 583.12065. ^1^H NMR (400 MHz, DMSO) *δ* 10.15 (s, 1H), 9.94 (s, 1H), 8.01 (d, *J* = 8.4 Hz, 2H), 7.57 (d, *J* = 8.4 Hz, 2H), 7.49 (d, *J* = 7.4 Hz, 1H), 7.46–7.40 (m, 1H), 7.40–7.36 (m, 2H), 7.27 (s, 2H), 7.07 (s, 1H), 7.01 (s, 1H), 6.13 (s, 2H), 3.78 (s, 6H), 3.65 (d, *J* = 4.1 Hz, 3H). ^13 ^C NMR (101 MHz, DMSO) *δ* 170.82, 166.27, 165.37, 153.08, 147.50, 146.70, 142.68, 137.07, 135.81, 134.19, 133.63, 131.40 (d, *J* = 6.6 Hz), 130.49, 130.10, 129.46, 129.13, 128.52, 127.88, 127.47, 109.86, 109.16, 102.28, 98.51, 60.61, 60.23, 56.22, 21.23, 14.55.

***3-Bromo-N-(6-(4-((3,4,5-trimethoxyphenyl)carbamoyl)phenyl)benzo[d][1,3]dioxol-5-yl)benzamide(A8)***

Crystallised from EtOAc to give a white powder (0.30 g, 87%); HRMS *m/z* [M + Na]^+^ calcd for C_30_H_25_BrN_2_NaO_7_: 627.07428, found: 627.07031. ^1^H NMR (400 MHz, DMSO) *δ* 10.09 (s, 1H), 9.99 (s, 1H), 7.99–7.92 (m, 3H), 7.81–7.72 (m, 2H), 7.53 (d, *J* = 8.4 Hz, 2H), 7.44 (t, *J* = 7.9 Hz, 1H), 7.23 (s, 2H), 7.03 (d, *J* = 2.9 Hz, 2H), 6.13 (s, 2H), 3.76 (s, 6H), 3.63 (s, 3H). ^13 ^C NMR (101 MHz, DMSO) *δ* 166.71, 165.38, 153.09, 147.53, 146.69, 142.64, 136.40, 135.81, 134.20, 133.69, 132.87, 131.30, 130.43, 129.39, 128.57 (d, *J* = 19.7 Hz), 127.91, 126.73 (d, *J* = 16.5 Hz), 126.34, 125.59, 122.87, 109.92, 108.69, 102.31, 98.52, 60.61, 56.22, 14.55.

***3-Fluoro-N-(6-(4-((3,4,5-trimethoxyphenyl)carbamoyl)phenyl)benzo[d][1,3]dioxol-5-yl)benzamide(A9)***

Crystallised from EtOAc to give a white powder (0.24 g, 77%); HRMS *m/z* [M + Na]^+^ calcd for C_30_H_25_FN_2_NaO_7_: 567.15435, found: 567.15039. ^1^H NMR (400 MHz, DMSO) *δ* 10.09 (s, 1H), 9.96 (s, 1H), 7.94 (d, *J* = 8.5 Hz, 2H), 7.65 (d, *J* = 7.8 Hz, 1H), 7.58 (d, *J* = 9.7 Hz, 1H), 7.56–7.48 (m, 3H), 7.39 (td, *J* = 8.3, 1.9 Hz, 1H), 7.22 (s, 2H), 7.04 (d, *J* = 2.4 Hz, 2H), 6.14 (s, 2H), 3.77 (d, *J* = 8.6 Hz, 6H), 3.64 (d, *J* = 5.5 Hz, 3H). ^13 ^C NMR (101 MHz, DMSO) *δ* 165.19 (d, *J* = 13.5 Hz), 163.58, 161.15, 153.06, 147.52, 146.82, 142.83, 137.09 (d, *J* = 6.6 Hz), 135.75, 134.18, 133.51, 131.81, 131.06 (d, *J* = 8.1 Hz), 129.13, 128.86, 127.87, 124.14, 118.87 (d, *J* = 20.9 Hz), 118.70–118.40 (m), 114.85, 114.62, 109.81 (d, *J* = 7.7 Hz), 102.27, 98.53, 60.58, 60.23, 56.19, 21.22, 14.55.

***2-Methoxy-N-(6-(4-((3,4,5-trimethoxyphenyl)carbamoyl)phenyl)benzo[d][1,3]dioxol-5-yl)benzamide(A10)***

Crystallised from EtOAc to give a white powder (0.26 g, 82%); HRMS *m/z* [M + Na]^+^ calcd for C_31_H_28_N_2_NaO_7_: 579.17434, found: 579.16992 [M + Na]^+^. ^1^H NMR (400 MHz, DMSO) *δ* 10.09 (s, 1H), 9.89 (s, 1H), 7.93 (d, *J* = 8.5 Hz, 2H), 7.86 (dd, *J* = 8.6, 5.6 Hz, 2H), 7.53 (d, *J* = 8.4 Hz, 2H), 7.30 (t, *J* = 8.9 Hz, 2H), 7.22 (s, 2H), 7.03 (d, *J* = 0.9 Hz, 2H), 6.13 (s, 2H), 3.76 (s, 6H), 3.63 (s, 3H). ^13 ^C NMR (101 MHz, DMSO) *δ* 165.23, 165.02, 153.05, 147.52, 146.82, 142.85, 136.95, 135.76, 134.68, 134.17, 133.51, 131.80, 131.13, 130.65, 129.11, 128.87, 127.87, 127.06, 125.87, 122.14, 109.80 (d, *J* = 6.5 Hz), 102.27, 98.52, 60.59, 56.20, 21.23, 14.55.

***3-(Trifluoromethyl)-N-(6-(4-((3,4,5-trimethoxyphenyl)carbamoyl)phenyl)benzo[d][1,3]dioxol-5-yl)benzamide(A11)***

Crystallised from EtOAc to give a white powder (0.28 g, 82%); HRMS *m/z* [M + Na]^+^ calcd for C_31_H_25_F_3_N_2_NaO_7_: 617.15116, found: 617.14703. ^1^H NMR (400 MHz, DMSO) *δ* 10.13 (s, 1H), 10.08 (s, 1H), 8.13–8.06 (m, 2H), 7.93 (t, *J* = 8.5 Hz, 3H), 7.72 (s, 1H), 7.55 (d, *J* = 8.3 Hz, 2H), 7.22 (s, 2H), 7.06 (d, *J* = 10.2 Hz, 2H), 6.14 (s, 2H), 3.75 (s, 6H), 3.63 (s, 3H). ^13 ^C NMR (101 MHz, DMSO) *δ* 165.19 (d, *J* = 9.0 Hz), 153.05, 147.55, 146.89, 142.80, 135.72 (d, *J* = 6.2 Hz), 134.18, 133.54, 131.92 (d, *J* = 14.5 Hz), 130.21, 129.13, 128.79, 128.55, 127.88, 125.75, 124.64, 123.04, 109.82 (d, *J* = 5.5 Hz), 102.29, 98.51, 60.58, 56.19, 21.21, 14.54.

***4-(Chloromethyl)-N-(6-(4-((3,4,5-trimethoxyphenyl)carbamoyl)phenyl)benzo[d][1,3]dioxol-5-yl)benzamide(A12)***

Crystallised from EtOAc to give a white powder (0.26 g, 79%); HRMS *m/z* [M + Na]^+^ calcd for C_31_H_27_ClN_2_NaO_7_: 597.14045, found: 597.13586. ^1^H NMR (400 MHz, DMSO) *δ* 10.11 (s, 1H), 9.91 (s, 1H), 7.95 (d, *J* = 8.2 Hz, 2H), 7.81 (d, *J* = 8.0 Hz, 2H), 7.54 (dd, *J* = 12.9, 8.2 Hz, 4H), 7.24 (s, 2H), 7.05 (d, *J* = 4.3 Hz, 2H), 6.14 (s, 2H), 4.80 (s, 2H), 3.76 (s, 6H), 3.64 (s, 3H). ^13 ^C NMR (101 MHz, DMSO) *δ* 166.00, 165.26, 153.05, 147.49, 146.72, 142.88, 141.42, 135.78, 134.61, 134.13, 133.47, 131.73, 129.42–128.91 (m), 128.33, 127.87, 109.83, 102.25, 98.48, 67.49, 60.58, 56.17,25.60.

***2-Methyl-N-(6-(4-((3,4,5-trimethoxyphenyl)carbamoyl)phenyl)benzo[d][1,3]dioxol-5-yl)benzamide(A13)***

Crystallised from EtOAc to give a white powder (0.24 g, 77%); HRMS *m/z* [M + Na]^+^ calcd for C_31_H_28_N_2_NaO_7_: 563.17942, found: 563.17566. ^1^H NMR (400 MHz, DMSO) *δ* 10.14 (s, 1H), 9.73 (s, 1H), 8.00 (d, *J* = 8.3 Hz, 2H), 7.55 (d, *J* = 8.2 Hz, 2H), 7.30 (dd, *J* = 14.6, 7.3 Hz, 4H), 7.22 (dd, *J* = 6.9, 4.4 Hz, 2H), 7.06 (s, 1H), 7.00 (s, 1H), 6.13 (s, 2H), 3.77 (s, 6H), 3.64 (s, 3H), 3.33 (s, 2H), 2.21 (s, 3H). ^13 ^C NMR (101 MHz, DMSO) *δ* 168.84, 165.32, 153.06, 147.48, 146.62, 143.07, 137.10, 135.90 (d, *J* = 18.8 Hz), 134.14, 133.55, 131.92, 130.93, 129.91, 129.37, 129.02, 127.79, 127.43, 125.88, 109.71 (d, *J* = 15.1 Hz), 102.21, 98.45, 60.60, 56.20, 19.60.

***4-Ethyl-N-(6-(4-((3,4,5-trimethoxyphenyl)carbamoyl)phenyl)benzo[d][1,3]dioxol-5-yl)benzamide(A14)***

Crystallised from EtOAc to give a white powder (0.22 g, 69%); HRMS *m/z* [M + Na]^+^ calcd for C_32_H_30_N_2_NaO_7_: 577.19507, found: 577.19098. ^1^H NMR (400 MHz, DMSO) *δ* 10.10 (s, 1H), 9.77 (s, 1H), 7.94 (d, *J* = 8.4 Hz, 2H), 7.73 (d, *J* = 8.1 Hz, 2H), 7.54 (d, *J* = 8.4 Hz, 2H), 7.28 (d, *J* = 8.2 Hz, 2H), 7.23 (s, 2H), 7.03 (d, *J* = 2.1 Hz, 2H), 6.13 (s, 2H), 3.76 (s, 6H), 3.63 (s, 3H). ^13 ^C NMR (101 MHz, DMSO) *δ* 166.34, 165.27, 153.05, 148.05, 147.45, 146.60, 142.95, 135.76, 134.17, 133.42, 132.22, 131.67, 129.25 (d, *J* = 15.8 Hz), 128.12 (d, *J* = 10.6 Hz), 127.84, 109.82, 102.20, 98.52, 60.59, 56.19, 28.49, 15.78.

***3,5-Difluoro-N-(6-(4-((3,4,5-trimethoxyphenyl)carbamoyl)phenyl)benzo[d][1,3]dioxol-5-yl)benzamide(A15)***

Crystallised from EtOAc to give a white powder (0.24 g, 75%); HRMS *m/z* [M + Na]^+^ calcd for C_30_H_24_F_2_N_2_NaO_7_: 585.14493, found: 585.14067. ^1^H NMR (400 MHz, DMSO) *δ* 10.09 (s, 1H), 10.03 (s, 1H), 7.96 (d, *J* = 8.4 Hz, 2H), 7.54 (d, *J* = 8.3 Hz, 2H), 7.51–7.44 (m, 3H), 7.23 (s, 2H), 7.04 (d, *J* = 1.8 Hz, 2H), 6.14 (s, 2H), 3.77 (s, 6H), 3.64 (s, 3H). ^13 ^C NMR (101 MHz, DMSO) *δ* 165.25, 163.93, 161.41 (d, *J* = 12.5 Hz), 153.05, 147.55, 146.95, 142.72, 138.20, 135.74, 134.18, 133.55, 131.84, 129.10, 128.52, 127.91, 111.48, 111.22, 109.77 (d, *J* = 21.1 Hz), 109.61–109.39 (m), 107.50, 102.32, 98.54, 60.58, 60.23, 56.19, 21.21, 14.54.

***2,5-Difluoro-N-(6-(4-((3,4,5-trimethoxyphenyl)carbamoyl)phenyl)benzo[d][1,3]dioxol-5-yl)benzamide(A16)***

Crystallised from EtOAc to give a white powder (0.25 g, 77%); HRMS *m/z* [M + Na]^+^ calcd for C_30_H_24_F_2_N_2_NaO_7_: 585.14493, found: 585.14106. ^1^H NMR (400 MHz, DMSO) *δ* 10.12 (s, 1H), 9.85 (s, 1H), 8.00 (d, *J* = 8.4 Hz, 2H), 7.57 (s, 2H), 7.40–7.31 (m, 3H), 7.26 (s, 2H), 7.14 (s, 1H), 7.01 (s, 1H), 6.13 (s, 2H), 3.77 (s, 6H), 3.64 (s, 3H). ^13 ^C NMR (101 MHz, DMSO) *δ* 165.29, 162.55, 156.90, 153.07, 147.50, 146.66, 142.55, 135.78, 134.19, 133.63, 131.09, 129.33, 128.42, 127.92, 118.69, 116.71, 109.91, 108.83, 102.30, 98.54, 60.60, 56.21.

***4-Cyano-N-(6-(4-((3,4,5-trimethoxyphenyl)carbamoyl)phenyl)benzo[d][1,3]dioxol-5-yl)benzamide(A17)***

Crystallised from EtOAc to give a white powder (0.21 g, 67%); HRMS *m/z* [M + Na]^+^ calcd for C_31_H_25_N_3_NaO_7_: 574.15902, found: 574.15552. ^1^H NMR (400 MHz, DMSO) *δ* 10.11 (s, 1H), 10.07 (s, 1H), 7.96–7.91 (m, 6H), 7.53 (d, *J* = 8.3 Hz, 2H), 7.22 (s, 2H), 7.05 (d, *J* = 8.7 Hz, 2H), 6.14 (s, 2H), 3.76 (s, 6H), 3.64 (s, 3H). ^13 ^C NMR (101 MHz, DMSO) *δ* 165.19 (d, *J* = 13.3 Hz), 153.06, 147.55, 146.92, 142.76, 138.76, 135.75, 134.18, 133.55, 132.97, 131.84, 129.10, 128.70 (d, *J* = 9.6 Hz), 127.90, 118.73, 109.78 (d, *J* = 18.9 Hz), 102.31, 98.52, 60.59, 56.20.

***Tert-butyl(6-(4-((4-chlorophenyl)carbamoyl)phenyl)benzo[d][1,3]dioxol-5-yl)carbamate(7–1)***

To a solution of compound **5** (0.60 g, 2.54 mmol) in DCM (6 ml), Et_3_N (0.31 g, 3.05 mmol) and TBTU (0.98 g, 3.05 mmol) were sequentially added. After 20 min, 4-chloroaniline (0.39 g, 3.05 mmol) and Et_3_N (0.31 g, 3.05 mmol) were added. The reaction was stirred at room temperature for 5 h. After the reaction, the solvent was evaporated *in vacuo*, and the residue was taken up in EtOAc (30 ml). The EtOAc solution was washed with saturated citric acid (3 × 30 ml), NaHCO_3_ (3 × 30 ml) and brine (3 × 30 ml), dried over MgSO_4_ and concentrated by evaporation *in vacuo*. The desired compound **7–1** (0.34 g, 40% yield) was derived by crystallisation in EtOAc as white powder. ^1^H NMR (400 MHz, DMSO) *δ* 10.33 (s, 1H), 9.90 (s, 1H), 7.93 (d, *J* = 8.3 Hz, 2H), 7.80 (d, *J* = 8.1 Hz, 2H), 7.75 (d, *J* = 8.9 Hz, 2H), 7.57–7.49 (m, 6H), 7.03 (d, *J* = 5.7 Hz, 2H), 6.13 (s, 2H), 4.79 (s, 2H).

***Tert-butyl(6-(4-((4-bromophenyl)carbamoyl)phenyl)benzo[d][1,3]dioxol-5-yl)carbamate(7–2)***

^1^H NMR (400 MHz, DMSO) *δ* 10.33 (s, 1H), 9.90 (s, 1H), 7.93 (d, *J* = 8.3 Hz, 2H), 7.80 (d, *J* = 8.1 Hz, 2H), 7.75 (d, *J* = 8.9 Hz, 2H), 7.57–7.49 (m, 6H), 7.03 (d, *J* = 5.7 Hz, 2H), 6.13 (s, 2H), 4.79 (s, 2H).

***Tert-butyl(6-(4-((4-fluorophenyl)carbamoyl)phenyl)benzo[d][1,3]dioxol-5-yl)carbamate(7–3)***

^1^H NMR (400 MHz, DMSO) *δ* 10.34 (s, 1H), 8.38 (s, 1H), 8.01 (d, *J* = 8.2 Hz, 2H), 7.83 (dd, *J* = 8.9, 5.1 Hz, 2H), 7.49 (d, *J* = 8.1 Hz, 2H), 7.20 (t, *J* = 8.9 Hz, 2H), 6.90 (d, *J* = 13.7 Hz, 2H), 6.08 (s, 2H).

***Tert-butyl(6-(4-((4-hydroxyphenyl)carbamoyl)phenyl)benzo[d][1,3]dioxol-5-yl)carbamate(7–4)***

^1^H NMR (400 MHz, DMSO) *δ* 10.33 (s, 1H), 9.90 (s, 1H), 7.93 (d, *J* = 8.3 Hz, 2H), 7.80 (d, *J* = 8.1 Hz, 2H), 7.75 (d, *J* = 8.9 Hz, 2H), 7.57–7.49 (m, 6H), 7.03 (d, *J* = 5.7 Hz, 2H), 6.13 (s, 2H), 4.79 (s, 2H).

***Tert-butyl(6-(4-((4-methoxyphenyl)carbamoyl)phenyl)benzo[d][1,3]dioxol-5-yl)carbamate(7–5)***

^1^H NMR (400 MHz, DMSO) *δ* 10.13 (s, 1H), 8.38 (s, 1H), 7.99 (d, *J* = 8.2 Hz, 2H), 7.71 (d, *J* = 9.0 Hz, 2H), 7.47 (d, *J* = 8.1 Hz, 2H), 6.92 (dd, *J* = 13.3, 11.4 Hz, 4H), 6.08 (s, 2H), 3.75 (s, 3H), 1.29 (s, 9H).

***Tert-butyl(6-(4-((4-ethoxyphenyl)carbamoyl)phenyl)benzo[d][1,3]dioxol-5-yl)carbamate(7–6)***

^1^H NMR (400 MHz, DMSO) *δ* 10.12 (s, 1H), 8.37 (s, 1H), 7.99 (d, *J* = 8.2 Hz, 2H), 7.69 (d, *J* = 8.9 Hz, 2H), 7.47 (d, *J* = 8.1 Hz, 2H), 6.91 (dd, *J* = 10.9, 7.2 Hz, 4H), 6.08 (s, 2H), 1.32 (dd, *J* = 15.2, 8.2 Hz, 12H).

***Tert-butyl(6-(4-((3,4-dimethoxyphenyl)carbamoyl)phenyl)benzo[d][1,3]dioxol-5-yl)carbamate(7–7)***

^1^H NMR (400 MHz, DMSO) *δ* 10.11 (s, 1H), 8.39 (s, 1H), 7.99 (d, *J* = 8.2 Hz, 2H), 7.52 (d, *J* = 2.2 Hz, 1H), 7.48 (d, *J* = 8.1 Hz, 2H), 7.37 (dd, *J* = 8.7, 2.2 Hz, 1H), 6.94 (d, *J* = 9.7 Hz, 2H), 6.88 (s, 1H), 6.08 (s, 2H), 3.75 (d, *J* = 7.7 Hz, 6H), 1.28 (s, 9H).

***Tert-butyl (6-(4-(p-tolylcarbamoyl)phenyl)benzo[d][1,3]dioxol-5-yl)carbamate(7–8)***

^1^H NMR (400 MHz, DMSO) *δ* 10.16 (s, 1H), 8.38 (s, 1H), 7.99 (d, *J* = 8.2 Hz, 2H), 7.68 (d, *J* = 8.3 Hz, 2H), 7.48 (d, *J* = 8.1 Hz, 2H), 7.16 (d, *J* = 8.3 Hz, 2H), 6.90 (d, *J* = 14.6 Hz, 2H), 6.08 (s, 2H), 2.29 (s, 3H), 1.28 (s, 9H).

***Tert-butyl(6-(4-((4-ethylphenyl)carbamoyl)phenyl)benzo[d][1,3]dioxol-5-yl)carbamate(7–9)***

^1^H NMR (400 MHz, DMSO) *δ* 10.17 (s, 1H), 8.38 (s, 1H), 7.99 (d, *J* = 8.2 Hz, 2H), 7.70 (d, *J* = 8.3 Hz, 2H), 7.48 (d, *J* = 8.1 Hz, 2H), 7.19 (d, *J* = 8.3 Hz, 2H), 6.90 (d, *J* = 14.0 Hz, 2H), 6.08 (s, 2H), 2.58 (q, *J* = 7.6 Hz, 2H), 1.29 (s, 9H), 1.18 (t, *J* = 7.6 Hz, 3H).

***Tert-butyl(6-(4-((4-pentylphenyl)carbamoyl)phenyl)benzo[d][1,3]dioxol-5-yl)carbamate(7–10)***

^1^H NMR (400 MHz, DMSO) *δ* 10.18 (s, 1H), 8.37 (s, 1H), 8.00 (d, *J* = 7.5 Hz, 2H), 7.70 (d, *J* = 7.6 Hz, 2H), 7.48 (d, *J* = 7.2 Hz, 2H), 7.17 (d, *J* = 7.6 Hz, 2H), 6.90 (d, *J* = 13.0 Hz, 2H), 6.08 (s, 2H), 2.54 (d, *J* = 7.4 Hz, 2H), 1.57 (s, 2H), 1.28 (s, 12H), 0.86 (d, *J* = 6.5 Hz, 3H).

***Tert-butyl(6-(4-([1,1′-biphenyl]-4-ylcarbamoyl)phenyl)benzo[d][1,3]dioxol-5-yl)carbamate(7–11)***

^1^H NMR (400 MHz, DMSO) *δ* 10.36 (s, 1H), 8.40 (s, 1H), 8.03 (d, *J* = 8.2 Hz, 2H), 7.93 (d, *J* = 8.6 Hz, 2H), 7.69 (d, *J* = 8.6 Hz, 4H), 7.53–7.43 (m, 4H), 7.34 (t, *J* = 7.3 Hz, 1H), 6.91 (d, *J* = 15.6 Hz, 2H), 6.09 (s, 2H), 1.29 (s, 9H).

***Tert-butyl(6-(4-((2,3-dihydrobenzo[b][1,4]dioxin-6-yl)carbamoyl)phenyl)benzo[d][1,3]dioxol-5-yl)carbamate(7–12)***

^1^H NMR (400 MHz, DMSO) *δ* 10.08 (s, 1H), 8.37 (s, 1H), 7.97 (d, *J* = 8.2 Hz, 2H), 7.47 (d, *J* = 8.1 Hz, 2H), 7.42 (d, *J* = 2.2 Hz, 1H), 7.23 (dd, *J* = 8.8, 2.3 Hz, 1H), 6.89 (d, *J* = 11.5 Hz, 2H), 6.83 (d, *J* = 8.7 Hz, 1H), 6.08 (s, 2H), 5.76 (s, 1H), 4.23 (q, *J* = 4.8 Hz, 4H), 1.28 (s, 9H).

***Tert-butyl(6-(4-(4-phenylpiperazine-1-carbonyl)phenyl)benzo[d][1,3]dioxol-5-yl)carbamate(7–13)***

^1^H NMR (400 MHz, DMSO) *δ* 8.40 (s, 1H), 7.43 (dd, *J* = 18.0, 8.0 Hz, 4H), 7.23 (t, *J* = 7.7 Hz, 2H), 6.97 (d, *J* = 8.3 Hz, 2H), 6.90 (s, 1H), 6.82 (dd, *J* = 13.6, 6.2 Hz, 2H), 6.07 (s, 2H), 3.65 (d, *J* = 77.3 Hz, 4H), 3.17 (s, 4H), 1.26 (s, 9H).

***Tert-butyl(6-(4-(4-(2-methoxyphenyl)piperazine-1-carbonyl)phenyl)benzo[d][1,3]dioxol-5-yl)carbamate(7–14)***

^1^H NMR (400 MHz, DMSO) *δ* 8.39 (s, 1H), 7.43 (dd, *J* = 18.3, 8.1 Hz, 4H), 7.01–6.83 (m, 6H), 6.07 (s, 2H), 3.79 (s, 3H), 3.78–3.43 (m, 4H), 2.97 (s, 4H), 2.69 (s, 1H), 1.22 (d, *J* = 24.8 Hz, 9H).

***Tert-butyl(6-(4-(4-(4-ethylphenyl)piperazine-1-carbonyl)phenyl)benzo[d][1,3]dioxol-5-yl)carbamate(7–15)***

^1^H NMR (400 MHz, DMSO) *δ* 8.39 (s, 1H), 7.39 (s, 4H), 7.31–7.12 (m, 6H), 6.86 (d, *J* = 20.2 Hz, 2H), 6.06 (s, 2H), 3.50 (d, *J* = 95.1 Hz, 4H), 2.79–2.71 (m, 2H), 2.69 (s, 1H), 2.60–2.51 (m, 3H), 2.44 (s, 3H), 1.22 (d, *J* = 20.9 Hz, 9H).

***Tert-butyl(6-(4-(4-(4-chlorophenyl)piperazine-1-carbonyl)phenyl)benzo[d][1,3]dioxol-5-yl)carbamate(7–16)***

^1^H NMR (400 MHz, DMSO) *δ* 8.40 (s, 1H), 7.43 (dd, *J* = 18.0, 8.0 Hz, 4H), 7.25 (d, *J* = 8.8 Hz, 2H), 6.98 (d, *J* = 8.9 Hz, 2H), 6.90 (s, 1H), 6.85 (s, 1H), 6.07 (s, 2H), 3.64 (d, *J* = 74.8 Hz, 4H), 3.18 (s, 4H), 1.26 (s, 9H).

***Tert-butyl(6-(4-(4-(4-fluorophenyl)piperazine-1-carbonyl)phenyl)benzo[d][1,3]dioxol-5-yl)carbamate(7–17)***

^1^H NMR (400 MHz, DMSO) *δ* 8.39 (d, *J* = 10.4 Hz, 1H), 7.93 (d, *J* = 8.1 Hz, 1H), 7.43 (q, *J* = 8.2 Hz, 4H), 7.11–6.97 (m, 3H), 6.90 (s, 1H), 6.85 (d, *J* = 3.5 Hz, 1H), 6.07 (s, 2H), 3.65 (d, *J* = 77.7 Hz, 4H), 3.23–2.97 (m, 4H), 1.26 (s, 9H).

***Tert-butyl(6-(4-(4-ethylpiperazine-1-carbonyl)phenyl)benzo[d][1,3]dioxol-5-yl)carbamate(7–18)***

^1^H NMR (400 MHz, DMSO) *δ* 8.39 (s, 1H), 7.39 (s, 4H), 6.86 (d, *J* = 20.2 Hz, 2H), 6.06 (s, 2H), 3.61 (s, 4H), 2.35 (dd, *J* = 14.2, 7.1 Hz, 6H), 1.37–1.15 (m, 9H), 1.00 (t, *J* = 7.1 Hz, 3H).

***Tert-butyl(6-(4-(4-isopropylpiperazine-1-carbonyl)phenyl)benzo[d][1,3]dioxol-5-yl)carbamate(7–19)***

^1^H NMR (400 MHz, DMSO) *δ* 8.38 (s, 1H), 7.38 (s, 4H), 6.86 (d, *J* = 18.7 Hz, 2H), 6.06 (s, 2H), 3.60 (s, 4H), 2.74–2.64 (m, 1H), 2.42 (s, 4H), 1.22 (d, *J* = 21.5 Hz, 9H), 0.97 (d, *J* = 6.5 Hz, 6H).

***Tert-butyl(6-(4-(isopropylcarbamoyl)phenyl)benzo[d][1,3]dioxol-5-yl)carbamate(7–20)***

^1^H NMR (400 MHz, DMSO) *δ* 8.31 (s, 1H), 8.21 (d, *J* = 7.8 Hz, 1H), 7.87 (d, *J* = 8.1 Hz, 2H), 7.40 (d, *J* = 8.1 Hz, 2H), 6.88 (s, 2H), 6.07 (s, 2H), 4.12 (dq, *J* = 13.4, 6.7 Hz, 1H), 1.28 (s, 9H), 1.17 (d, *J* = 6.6 Hz, 7H).

***4-(Chloromethyl)-N-(6-(4-((4-chlorophenyl)carbamoyl)phenyl)benzo[d][1,3]dioxol-5-yl)benzamide(B1)***

Compound **7–1** (0.3 g, 0.65 mmol) was dissolved in dry DCM. After addition of TFA, the solvent was stirred at room temperature, monitored by TLC. Until the raw materials were completely consumed, the reagents were evaporated under vacuum and dissolved with a mixed solution of tetrahydrofuran and water (50:1) at 0 °C with addition of sodium bicarbonate. After stirred for 10 min, 4-(chloromethyl)benzoyl chloride (0.15 g, 0.77 mmol) was added, and the mixture was stirred at room temperature for 4 h. After that, the reagents were evaporated under vacuum and dissolved in EtOAc. The solvent was washed with saturated NaHCO_3_ (3 × 30 ml) and brine (3 × 30 ml), dried over MgSO_4_, and evaporated under vacuo. The desired compound **B1** (0.3 g, 89% yield) was derived by crystallisation in EtOAc as white powder. HRMS *m/z* [M + H]^+^ calcd for C_28_H_21_Cl_2_N_2_O_4_: 519.08784, found: 519.08398. ^1^H NMR (400 MHz, DMSO) *δ* 10.33 (s, 1H), 9.90 (s, 1H), 7.93 (d, *J* = 8.3 Hz, 2H), 7.85–7.77 (m, 4H), 7.53 (dd, *J* = 13.9, 8.2 Hz, 4H), 7.40 (d, *J* = 8.8 Hz, 2H), 7.03 (d, *J* = 5.7 Hz, 2H), 6.13 (s, 2H), 4.79 (s, 2H). ^13 ^C NMR (101 MHz, DMSO) *δ* 165.99, 165.64, 147.51, 146.72, 143.05, 141.42, 138.62, 134.61, 133.29, 131.71, 129.41–128.86 (m), 128.32, 128.02, 127.70, 122.32, 109.81 (d, *J* = 4.0 Hz), 102.25, 45.86, 25.60.

***N-(4-bromophenyl)-4-(6-(4-(chloromethyl)benzamido)benzo[d][1,3]dioxol-5-yl)benzamide(B2)***

Crystallised from EtOAc to give a white powder (0.22 g, 86%); HRMS *m/z* [M + H]^+^ calcd for C_28_H_21_BrClN_2_O_4_: 565.03528, found: 565.03082. ^1^H NMR (400 MHz, DMSO) *δ* 10.33 (s, 1H), 9.90 (s, 1H), 7.93 (d, *J* = 8.3 Hz, 2H), 7.80 (d, *J* = 8.1 Hz, 2H), 7.75 (d, *J* = 8.9 Hz, 2H), 7.57–7.49 (m, 6H), 7.03 (d, *J* = 5.7 Hz, 2H), 6.13 (s, 2H), 4.79 (s, 2H). ^13 ^C NMR (101 MHz, DMSO) *δ* 165.99, 165.64, 147.51, 146.72, 143.06, 141.42, 139.04, 134.61, 133.28, 131.79 (d, *J* = 17.0 Hz), 129.41–128.92 (m), 128.32, 128.02, 122.70, 115.77, 109.79, 102.25, 67.50, 45.86, 25.60.

***4-(Chloromethyl)-N-(6-(4-((4-fluorophenyl)carbamoyl)phenyl)benzo[d][1,3]dioxol-5-yl)benzamide(B3)***

Crystallised from EtOAc to give a white powder (0.28 g, 85%); HRMS *m/z* [M + Na]^+^ calcd for C_28_H_20_ClFN_2_NaO_4_: 525.09933, found: 525.09540. ^1^H NMR (400 MHz, DMSO) *δ* 10.26 (s, 1H), 9.89 (s, 1H), 7.94 (d, *J* = 8.2 Hz, 2H), 7.83–7.75 (m, 4H), 7.53 (dd, *J* = 12.6, 8.2 Hz, 4H), 7.18 (t, *J* = 8.9 Hz, 2H), 7.03 (d, *J* = 6.5 Hz, 2H), 6.13 (s, 2H), 4.79 (s, 2H). ^13 ^C NMR (101 MHz, DMSO) *δ* 165.99, 165.45, 157.54, 147.49, 146.71, 142.93, 141.41, 135.98, 134.60, 133.38, 131.73, 129.33–128.91 (m), 128.32, 127.95, 122.66 (d, *J* = 7.9 Hz), 115.73, 115.50, 109.79, 102.24, 45.85.

***4-(Chloromethyl)-N-(6-(4-((4-hydroxyphenyl)carbamoyl)phenyl)benzo[d][1,3]dioxol-5-yl)benzamide(B4)***

Crystallised from EtOAc to give a white powder (0.13 g, 52%); HRMS *m/z* [M-H]^−^ calcd for C_28_H_20_ClN_2_O_5_: 499.10607, found: 499.10464. ^1^H NMR (400 MHz, DMSO) *δ* 9.98 (s, 1H), 9.88 (s, 1H), 9.24 (s, 1H), 7.92 (d, *J* = 8.2 Hz, 2H), 7.80 (d, *J* = 8.0 Hz, 2H), 7.54–7.47 (m, 6H), 7.03 (d, *J* = 8.5 Hz, 2H), 6.72 (d, *J* = 8.8 Hz, 2H), 6.13 (s, 2H), 4.79 (s, 2H). ^13 ^C NMR (101 MHz, DMSO) *δ* 166.01, 164.92, 154.15, 147.44, 146.69, 142.55, 141.40, 134.62, 133.77, 131.79, 131.14, 129.15 (d, *J* = 14.2 Hz), 128.32, 127.81, 122.73, 115.40, 109.78, 102.22, 45.86, 14.56.

***4-(Chloromethyl)-N-(6-(4-((4-methoxyphenyl)carbamoyl)phenyl)benzo[d][1,3]dioxol-5-yl)benzamide(B5)***

Crystallised from EtOAc to give a white powder (0.2 g, 61%); HRMS *m/z* [M + H]^+^ calcd for C_29_H_24_ClN_2_O_5_: 515.13737, found: 515.13367. ^1^H NMR (400 MHz, DMSO) *δ* 10.09 (s, 1H), 9.89 (s, 1H), 7.93 (d, *J* = 8.3 Hz, 2H), 7.80 (d, *J* = 8.1 Hz, 2H), 7.66 (d, *J* = 9.0 Hz, 2H), 7.52 (dd, *J* = 7.8, 6.8 Hz, 4H), 7.03 (d, *J* = 6.7 Hz, 2H), 6.91 (d, *J* = 9.0 Hz, 2H), 6.13 (s, 2H), 4.79 (s, 2H), 3.74 (s, 3H). ^13 ^C NMR (101 MHz, DMSO) *δ* 166.01, 165.08, 156.00, 147.46, 146.71, 142.68, 141.41, 134.63, 133.66, 132.69, 131.77, 129.17 (d, *J* = 10.8 Hz), 128.32, 127.87, 122.45, 114.18, 109.81 (d, *J* = 4.7 Hz), 102.23, 55.64, 45.86, 14.56.

***4-(Chloromethyl)-N-(6-(4-((4-ethoxyphenyl)carbamoyl)phenyl)benzo[d][1,3]dioxol-5-yl)benzamide(B6)***

Crystallised from EtOAc to give a white powder (0.18 g, 53%); HRMS *m/z* [M + Na]^+^ calcd for C_30_H_25_ClN_2_NaO_5_: 551.13497, found: 551.13086. ^1^H NMR (400 MHz, DMSO) *δ* 10.08 (s, 1H), 9.88 (s, 1H), 7.93 (d, *J* = 8.2 Hz, 2H), 7.80 (d, *J* = 8.0 Hz, 2H), 7.65 (d, *J* = 8.9 Hz, 2H), 7.52 (t, *J* = 7.6 Hz, 4H), 7.03 (d, *J* = 8.2 Hz, 2H), 6.89 (d, *J* = 9.0 Hz, 2H), 6.13 (s, 2H), 4.79 (s, 2H), 1.31 (t, *J* = 6.9 Hz, 3H). ^13 ^C NMR (101 MHz, DMSO) *δ* 166.00, 165.07, 155.25, 147.45, 146.70, 142.67, 141.41, 134.62, 133.67, 132.58, 131.77, 129.17 (d, *J* = 11.4 Hz), 128.32, 127.86, 122.43, 114.69, 109.83, 102.23, 63.55, 45.86, 15.17.

***4-(Chloromethyl)-N-(6-(4-((3,4-dimethoxyphenyl)carbamoyl)phenyl)benzo[d][1,3]dioxol-5-yl)benzamide(B7)***

Crystallised from EtOAc to give a white powder (0.21 g, 64%); HRMS *m/z* [M + H]^+^ calcd for C_30_H_25_ClN_2_O_6_: 545.14794, found: 545.14398. ^1^H NMR (400 MHz, DMSO) *δ* 10.06 (s, 1H), 9.89 (s, 1H), 7.93 (d, *J* = 8.3 Hz, 2H), 7.80 (d, *J* = 8.1 Hz, 2H), 7.52 (t, *J* = 7.7 Hz, 4H), 7.46 (d, *J* = 2.2 Hz, 1H), 7.32 (dd, *J* = 8.7, 2.2 Hz, 1H), 7.03 (d, *J* = 4.1 Hz, 2H), 6.91 (d, *J* = 8.8 Hz, 1H), 6.13 (s, 2H), 4.79 (s, 2H), 3.73 (d, *J* = 2.8 Hz, 6H). ^13 ^C NMR (101 MHz, DMSO) *δ* 165.03, 148.85, 147.46, 146.71, 145.58, 142.70, 141.41, 134.61, 133.61, 133.15, 131.77, 129.17 (d, *J* = 12.0 Hz), 128.32, 127.83, 112.76, 112.30, 109.81, 105.95, 102.23, 67.49, 56.17, 55.84, 45.85.

***4-(Chloromethyl)-N-(6-(4-(p-tolylcarbamoyl)phenyl)benzo[d][1,3]dioxol-5-yl)benzamide(B8)***

Crystallised from EtOAc to give a white powder (0.22 g, 67%); HRMS *m/z* [M + H]^+^ calcd for C_29_H_24_ClN_2_O_4_: 499.14246, found: 499.13864. ^1^H NMR (400 MHz, DMSO) *δ* 10.17 (s, 1H), 8.38 (s, 1H), 7.99 (d, *J* = 8.2 Hz, 2H), 7.70 (d, *J* = 8.3 Hz, 2H), 7.48 (d, *J* = 8.1 Hz, 2H), 7.19 (d, *J* = 8.3 Hz, 2H), 6.90 (d, *J* = 14.0 Hz, 2H), 6.08 (s, 2H), 2.58 (q, *J* = 7.6 Hz, 2H), 1.29 (s, 9H), 1.18 (t, *J* = 7.6 Hz, 3H). ^13 ^C NMR (101 MHz, DMSO) *δ* 166.00, 165.30, 147.46, 146.71, 142.75, 141.41, 137.09, 134.61, 133.63, 133.03, 131.76, 129.53–128.75 (m), 128.32, 127.92, 120.85, 109.83, 102.23, 45.86, 20.97.

***4-(Chloromethyl)-N-(6-(4-((4-ethylphenyl)carbamoyl)phenyl)benzo[d][1,3]dioxol-5-yl)benzamide(B9)***

Crystallised from EtOAc to give a white powder (0.25 g, 68%); HRMS *m/z* [M + H]^+^ calcd for C_30_H_26_ClN_2_O_4_: 513.15811, found: 513.15448. ^1^H NMR (400 MHz, DMSO) *δ* 10.13 (s, 1H), 9.89 (s, 1H), 7.93 (d, *J* = 8.2 Hz, 2H), 7.80 (d, *J* = 8.0 Hz, 2H), 7.66 (d, *J* = 8.4 Hz, 2H), 7.53 (t, *J* = 8.3 Hz, 4H), 7.17 (d, *J* = 8.4 Hz, 2H), 7.03 (d, *J* = 6.6 Hz, 2H), 6.13 (s, 2H), 4.79 (s, 2H), 2.57 (q, *J* = 7.6 Hz, 2H), 1.17 (t, *J* = 7.6 Hz, 3H). ^13 ^C NMR (101 MHz, DMSO) *δ* 165.99, 165.30, 147.46, 146.71, 142.76, 141.41, 139.52, 137.29, 134.61, 133.62, 131.76, 129.17 (d, *J* = 11.3 Hz), 128.28 (d, *J* = 8.5 Hz), 127.92, 120.92, 109.83, 102.23, 45.85, 28.11, 16.20.

***4-(Chloromethyl)-N-(6-(4-((4-pentylphenyl)carbamoyl)phenyl)benzo[d][1,3]dioxol-5-yl)benzamide(B10)***

Crystallised from EtOAc to give a white powder (0.24 g, 73%); HRMS *m/z* [M + H]^+^ calcd for C_33_H_31_ClN_2_O_4_: 555.20506, found: 555.1996. ^1^H NMR (400 MHz, DMSO) *δ* 10.19 (s, 1H), 9.96 (s, 1H), 7.95 (d, *J* = 8.2 Hz, 2H), 7.82 (d, *J* = 8.0 Hz, 2H), 7.67 (d, *J* = 8.3 Hz, 2H), 7.53 (dd, *J* = 12.0, 8.2 Hz, 4H), 7.14 (d, *J* = 8.3 Hz, 2H), 7.03 (d, *J* = 5.8 Hz, 2H), 6.13 (s, 2H), 4.79 (s, 2H), 2.58–2.51 (m, 2H), 1.61–1.50 (m, 2H), 1.37–1.20 (m, 4H), 0.86 (t, *J* = 6.9 Hz, 3H). ^13 ^C NMR (101 MHz, DMSO) *δ* 165.98, 165.30, 147.44, 146.69, 142.77, 141.38, 138.06, 137.31, 134.61, 133.59, 131.81, 129.15 (d, *J* = 10.0 Hz), 128.73, 128.35, 127.95, 120.91, 109.81, 102.22, 45.87, 35.03, 31.24 (d, *J* = 12.7 Hz), 22.43, 14.41.

***N-([1,1′-biphenyl]-4-yl)-4-(6-(4-(chloromethyl)benzamido)benzo[d][1,3]dioxol-5-yl)benzamide(B11)***

Crystallised from EtOAc to give a white powder (0.16 g, 64%); HRMS *m/z* [M + H]^+^ calcd for C_34_H_26_ClN_2_O_4_: 561.15811, found: 561.15411. ^1^H NMR (400 MHz, DMSO) *δ* 10.31 (s, 1H), 9.90 (s, 1H), 7.97 (d, *J* = 8.2 Hz, 2H), 7.88 (d, *J* = 8.5 Hz, 2H), 7.81 (d, *J* = 8.0 Hz, 2H), 7.67 (d, *J* = 7.4 Hz, 4H), 7.54 (dd, *J* = 15.6, 8.1 Hz, 4H), 7.45 (t, *J* = 7.6 Hz, 2H), 7.33 (t, *J* = 7.3 Hz, 1H), 7.04 (d, *J* = 4.9 Hz, 2H), 6.14 (s, 2H), 4.80 (s, 2H). ^13 ^C NMR (101 MHz, DMSO) *δ* 166.01, 165.56, 147.50, 146.72, 142.94, 141.42, 140.18, 139.15, 135.72, 134.62, 133.52, 131.74, 129.23 (dd, *J* = 16.6, 10.3 Hz), 128.33, 128.02, 127.54, 127.24, 126.76, 121.13, 109.82 (d, *J* = 4.4 Hz), 102.25, 67.50, 45.86, 25.60.

***4-(Chloromethyl)-N-(6-(4-((2,3-dihydrobenzo[b][1,4]dioxin-6-yl)carbamoyl)phenyl)benzo[d][1,3]dioxol-5-yl)benzamide(B12)***

Crystallised from EtOAc to give a white powder (0.12 g, 54%); HRMS *m/z* [M + Na]^+^ calcd for C_30_H_23_ClN_2_O_6_: 565.11423, found: 565.10828. ^1^H NMR (400 MHz, DMSO) *δ* 10.12 (s, 1H), 9.97 (s, 1H), 7.94 (d, *J* = 8.1 Hz, 2H), 7.82 (d, *J* = 7.8 Hz, 2H), 7.52 (t, *J* = 8.4 Hz, 4H), 7.40 (s, 1H), 7.22 (d, *J* = 8.7 Hz, 1H), 7.02 (d, *J* = 6.3 Hz, 2H), 6.80 (d, *J* = 8.7 Hz, 1H), 6.13 (s, 2H), 4.79 (s, 2H), 4.22 (d, *J* = 2.6 Hz, 4H). ^13 ^C NMR (101 MHz, DMSO) *δ* 165.97, 165.10, 147.43, 146.69, 143.24, 142.73, 141.38, 140.06, 134.60, 133.53, 133.30, 131.82, 129.14 (d, *J* = 11.8 Hz), 128.35, 127.91, 117.01, 114.13, 109.89 (d, *J* = 15.9 Hz), 102.22, 64.84–64.67 (m), 64.54 (d, *J* = 20.9 Hz), 45.87.

***4-(Chloromethyl)-N-(6-(4-(4-phenylpiperazine-1-carbonyl)phenyl)benzo[d][1,3]dioxol-5-yl)benzamide(B13)***

Crystallised from EtOAc to give a white powder (0.25 g, 76%); HRMS *m/z* [M + Na]^+^ calcd for C_32_H_28_ClN_3_NaO_4_: 576.16660, found: 576.16376. ^1^H NMR (400 MHz, DMSO) *δ* 9.83 (s, 1H), 7.75 (d, *J* = 7.9 Hz, 2H), 7.51 (d, *J* = 8.0 Hz, 2H), 7.43 (dd, *J* = 18.0, 8.1 Hz, 4H), 7.23 (t, *J* = 7.8 Hz, 2H), 7.03 (d, *J* = 10.1 Hz, 2H), 6.95 (d, *J* = 8.3 Hz, 2H), 6.81 (t, *J* = 7.2 Hz, 1H), 6.12 (s, 2H), 4.79 (s, 2H), 3.58 (d, *J* = 113.4 Hz, 4H), 3.15 (s, 4H). ^13 ^C NMR (101 MHz, DMSO) *δ* 169.31, 166.04, 151.23, 147.33, 146.62, 141.33, 140.82, 134.66 (d, *J* = 16.5 Hz), 131.86, 129.64–128.86 (m), 128.28, 127.37, 119.90, 116.45, 109.67 (d, *J* = 4.9 Hz), 102.17, 60.23, 48.96, 45.85, 21.24, 14.56.

***4-(Chloromethyl)-N-(6-(4-(4-(2-methoxyphenyl)piperazine-1-carbonyl)phenyl)benzo[d][1,3]dioxol-5-yl)benzamide(B14)***

Crystallised from EtOAc to give a white powder (0.23 g, 58%); HRMS *m/z* [M + H]^+^ calcd for C_33_H_31_ClN_3_O_5_: 584.19522, found: 584.19269. ^1^H NMR (400 MHz, DMSO) *δ* 9.82 (s, 1H), 7.74 (d, *J* = 8.0 Hz, 2H), 7.51–7.38 (m, 6H), 7.04 (s, 1H), 7.02–6.92 (m, 3H), 6.88 (s, 2H), 6.12 (s, 2H), 4.76 (s, 2H), 3.79 (s, 3H), 3.77–3.35 (m, 4H), 2.93 (d, *J* = 30.2 Hz, 4H). ^13 ^C NMR (101 MHz, DMSO) *δ* 169.24, 166.03, 152.51, 147.32, 146.61, 141.23 (d, *J* = 15.6 Hz), 140.75, 134.71 (d, *J* = 9.0 Hz), 131.87, 129.17 (d, *J* = 15.0 Hz), 128.27, 127.36, 123.43, 121.26, 118.90, 112.35, 109.65 (d, *J* = 6.4 Hz), 102.17, 55.82, 50.68, 45.82, 14.56.

***4-(Chloromethyl)-N-(6-(4-(4-(4-ethylphenyl)piperazine-1-carbonyl)phenyl)benzo[d][1,3]dioxol-5-yl)benzamide(B15)***

Crystallised from EtOAc to give a white powder (0.23 g, 58%); HRMS *m/z* [M + H]^+^ calcd for C_34_H_33_ClN_3_O_4_: 582.21596, found: 582.21332. ^1^H NMR (400 MHz, DMSO) *δ* 9.81 (s, 1H), 7.73 (d, *J* = 8.0 Hz, 2H), 7.49 (d, *J* = 8.0 Hz, 2H), 7.43 (d, *J* = 8.0 Hz, 2H), 7.35 (d, *J* = 8.0 Hz, 2H), 7.27 (t, *J* = 7.5 Hz, 2H), 7.24–7.14 (m, 3H), 7.02 (d, *J* = 13.3 Hz, 2H), 6.12 (s, 2H), 4.79 (s, 2H), 3.59 (s, 2H), 3.27 (s, 1H), 2.78–2.70 (m, 2H), 2.58–2.51 (m, 2H), 2.37 (s, 3H). ^13 ^C NMR (101 MHz, DMSO) *δ* 169.19, 166.00, 147.31, 146.59, 141.31, 140.70, 134.73, 131.90, 129.17 (d, *J* = 14.2 Hz), 128.72, 128.25, 127.22, 126.35, 109.64 (d, *J* = 5.7 Hz), 102.16, 59.94, 45.86, 33.05.

***4-(Chloromethyl)-N-(6-(4-(4-(4-chlorophenyl)piperazine-1-carbonyl)phenyl)benzo[d][1,3]dioxol-5-yl)benzamide(B16)***

Crystallised from EtOAc to give a white powder (0.18 g, 60%); HRMS *m/z* [M + Na]^+^ calcd for C_32_H_27_Cl_2_N_3_NaO_4_: 610.12763, found: 610.12469. ^1^H NMR (400 MHz, DMSO) *δ* 9.83 (s, 1H), 7.74 (d, *J* = 8.0 Hz, 2H), 7.51 (d, *J* = 8.0 Hz, 2H), 7.43 (dd, *J* = 18.3, 8.1 Hz, 4H), 7.25 (d, *J* = 8.8 Hz, 2H), 7.02 (d, *J* = 8.8 Hz, 2H), 6.96 (d, *J* = 8.9 Hz, 2H), 6.12 (s, 2H), 4.79 (s, 2H), 3.87–3.48 (m, 4H), 3.15 (s, 4H). ^13 ^C NMR (101 MHz, DMSO) *δ* 150.02, 129.20 (d, *J* = 11.0 Hz), 128.27, 127.37, 117.87, 109.68, 102.17, 45.86.

***4-(Chloromethyl)-N-(6-(4-(4-(4-fluorophenyl)piperazine-1-carbonyl)phenyl)benzo[d][1,3]dioxol-5-yl)benzamide(B17)***

Crystallised from EtOAc to give a white powder (0.18 g, 62%); HRMS *m/z* [M + Na]^+^ calcd for C_32_H_27_ClFN_3_NaO_4_: 594.15718, found: 594.15393. ^1^H NMR (400 MHz, DMSO) *δ* 9.83 (s, 1H), 7.75 (d, *J* = 8.0 Hz, 2H), 7.50 (d, *J* = 8.0 Hz, 2H), 7.43 (dd, *J* = 18.9, 8.1 Hz, 4H), 7.05 (dd, *J* = 20.4, 9.5 Hz, 4H), 6.97 (dd, *J* = 9.0, 4.7 Hz, 2H), 6.12 (s, 2H), 4.79 (s, 2H), 3.58 (d, *J* = 112.2 Hz, 4H), 3.08 (s, 4H). ^13 ^C NMR (101 MHz, DMSO) *δ* 169.30, 166.03, 155.65, 148.13, 147.33, 146.62, 141.33, 140.83, 134.64 (d, *J* = 18.3 Hz), 131.85, 129.20 (d, *J* = 12.8 Hz), 128.28, 127.36, 118.35 (d, *J* = 7.5 Hz), 115.92, 115.71, 109.65, 102.17, 60.23, 49.77, 45.85, 21.24, 14.56.

***4-(Chloromethyl)-N-(6-(4-(4-ethylpiperazine-1-carbonyl)phenyl)benzo[d][1,3]dioxol-5-yl)benzamide(B18)***

Crystallised from EtOAc to give a white powder (0.21 g, 64%); HRMS *m/z* [M + H]^+^ calcd for C_28_H_29_ClN_3_O_4_: 506.18466, found: 506.18207.3 ^1^H NMR (400 MHz, DMSO) *δ* 9.81 (s, 1H), 7.72 (d, *J* = 7.9 Hz, 2H), 7.49 (d, *J* = 8.0 Hz, 2H), 7.42 (d, *J* = 7.9 Hz, 2H), 7.34 (d, *J* = 8.0 Hz, 2H), 7.02 (d, *J* = 12.8 Hz, 2H), 6.12 (s, 2H), 4.79 (s, 2H), 3.58 (s, 2H), 3.26 (s, 2H), 2.38 (s, 1H), 2.33 (dd, *J* = 14.3, 7.1 Hz, 4H), 2.27 (s, 1H), 0.99 (t, *J* = 7.1 Hz, 3H). ^13 ^C NMR (101 MHz, DMSO) *δ* 169.17, 166.00, 147.30, 146.59, 141.31, 140.68, 134.73, 131.90, 129.17 (d, *J* = 13.3 Hz), 128.25, 127.21, 109.64 (d, *J* = 5.0 Hz), 102.16, 51.91, 45.85, 12.32.

***4-(Chloromethyl)-N-(6-(4-(4-isopropylpiperazine-1-carbonyl)phenyl)benzo[d][1,3]dioxol-5-yl)benzamide(B19)***

Crystallised from EtOAc to give a white powder (0.18 g, 54%); HRMS *m/z* [M + H]^+^ calcd for C_29_H_31_ClN_3_O_4_: 520.20031, found: 520.19751. ^1^H NMR (400 MHz, DMSO) *δ* 9.81 (s, 1H), 7.72 (d, *J* = 8.0 Hz, 2H), 7.49 (d, *J* = 8.1 Hz, 2H), 7.42 (d, *J* = 8.0 Hz, 2H), 7.34 (d, *J* = 8.0 Hz, 2H), 7.01 (d, *J* = 13.0 Hz, 2H), 6.12 (s, 2H), 4.79 (s, 2H), 3.50 (d, *J* = 47.9 Hz, 2H), 3.24 (s, 2H), 2.73–2.62 (m, 1H), 2.39 (d, *J* = 44.8 Hz, 4H), 0.96 (d, *J* = 6.5 Hz, 6H). ^13 ^C NMR (101 MHz, DMSO) *δ* 169.12, 166.01, 147.30, 146.59, 141.31, 140.65, 134.75 (d, *J* = 4.9 Hz), 131.93, 129.16 (d, *J* = 12.3 Hz), 128.25, 127.24, 109.64 (d, *J* = 5.6 Hz), 102.16, 54.19, 45.85, 18.48.

***4-(Chloromethyl)-N-(6-(4-(isopropylcarbamoyl)phenyl)benzo[d][1,3]dioxol-5-yl)benzamide(B20)***

Crystallised from EtOAc to give a white powder (0.2 g, 59%); HRMS *m/z* [M + Na]^+^ calcd for C_25_H_23_ClN_2_NaO_4_: 473.12440, found: 473.12238. ^1^H NMR (400 MHz, DMSO) *δ* 9.84 (s, 1H), 8.17 (d, *J* = 7.8 Hz, 1H), 7.80 (t, *J* = 8.8 Hz, 4H), 7.48 (dd, *J* = 20.5, 8.1 Hz, 4H), 7.01 (d, *J* = 13.3 Hz, 2H), 6.12 (s, 2H), 4.79 (s, 2H), 4.08 (dd, *J* = 13.6, 6.8 Hz, 1H), 1.14 (d, *J* = 6.6 Hz, 6H). ^13 ^C NMR (101 MHz, DMSO) *δ* 165.94, 165.34, 147.36, 146.66, 142.17, 141.40, 134.58, 133.51, 131.85, 129.21, 128.96 (d, *J* = 10.3 Hz), 128.29, 127.51, 109.78 (d, *J* = 4.4 Hz), 102.19, 45.86, 41.40, 22.80.

***Methyl 4-(6-(4-(chloromethyl)benzamido)benzo[d][1,3]dioxol-5-yl)benzoate (8)***

Compound **4** (2.00 g, 5.39 mmol) was dissolved in dry DCM. After addition of TFA, the solvent was stirred at room temperature, monitored by TLC. Until the raw materials were completely consumed, the reagents were evaporated under vacuum and dissolved with a mixed solution of tetrahydrofuran and water (50:1) at 0 °C with addition of sodium bicarbonate (1.36 g, 16.17 mmol). After stirred for 10 min, 4-(chloromethyl)benzoyl chloride (1.22 g, 6.47 mmol) was added, and the mixture was stirred at room temperature for 4 h. After that, the reagents were evaporated under vacuum and dissolved in EtOAc. The solvent was washed with saturated NaHCO_3_ (3 × 30 ml) and brine (3 × 30 ml), dried over MgSO_4_, and evaporated under vacuo. The desired compound **8** (2.00 g, 80% yield) was derived by crystallisation in EtOAc as yellow powder. ^1^H NMR (400 MHz, DMSO) *δ* 9.88 (s, 1H), 7.91 (d, *J* = 8.2 Hz, 2H), 7.75 (d, *J* = 8.0 Hz, 2H), 7.52 (dd, *J* = 11.2, 8.3 Hz, 4H), 7.02 (d, *J* = 6.4 Hz, 2H), 6.13 (s, 2H), 4.79 (s, 2H), 3.82 (s, 3H).

***4-(6-(4-(Methoxymethyl)benzamido)benzo[d][1,3]dioxol-5-yl)benzoic acid (9)***

Compound **8** (4.00 g, 10.78 mmol) was added to methanol and 3 mol/l NaOH (1:1, 80 ml), and reacted at 40 °C for about 10 h. After the reaction is over, the mixture was evaporated to dryness by rotary evaporation; appropriate amount of water was added, the PH was adjusted to acidity with 3 mol/l HCL, and EtOAc was added for extraction (3 × 100 ml). The ester layer was washed with brine, dried over MgSO_4_, and evaporated under vacuo. The desired compound **9** (3.43 g, 89% yield) was derived by crystallisation in EtOAc as yellow powder. ^1^H NMR (400 MHz, DMSO) *δ* 12.89 (s, 1H), 9.81 (s, 1H), 7.88 (d, *J* = 8.2 Hz, 2H), 7.73 (t, *J* = 7.6 Hz, 2H), 7.50 (d, *J* = 8.1 Hz, 2H), 7.37 (d, *J* = 8.0 Hz, 2H), 7.02 (d, *J* = 9.1 Hz, 2H), 6.12 (s, 2H), 4.45 (s, 2H), 3.30 (s, 3H).

***N-(3,5-dimethoxyphenyl)-4-(6-(4-(methoxymethyl)benzamido)benzo[d][1,3]dioxol-5-yl)benzamide(C1)***

To a solution of compound **9** (0.30 g, 0.73 mmol) in DCM (6 ml), Et3N (0.09 g, 0.88 mmol) and TBTU (0.27 g, 0.88 mmol) were sequentially added. After 20 min, 3,5-dimethoxyaniline (0.13 g, 0.88 mmol) was added. The reaction was stirred at room temperature for 5 h. After the reaction, the solvent was evaporated *in vacuo*, and the residue was taken up in EtOAc (30 ml). The EtOAc solution was washed with saturated citric acid (3 × 30 ml), NaHCO_3_ (3 × 30 ml) and brine (3 × 30 ml), dried over MgSO_4_ and concentrated by evaporation *in vacuo*. The desired compound **C1** (0.21 g, 53% yield) was derived by crystallisation in EtOAc as white powder. HRMS *m/z* [M + Na]^+^ calcd for C_31_H_28_N_2_NaO_7_: 563.17942, found: 563.17407. ^1^H NMR (400 MHz, DMSO) *δ* 10.12 (s, 1H), 9.84 (s, 1H), 7.92 (d, *J* = 8.3 Hz, 2H), 7.78 (t, *J* = 8.0 Hz, 2H), 7.54 (d, *J* = 8.2 Hz, 2H), 7.38 (d, *J* = 8.0 Hz, 2H), 7.08 (d, *J* = 2.1 Hz, 2H), 7.03 (d, *J* = 6.3 Hz, 2H), 6.25 (t, *J* = 2.0 Hz, 1H), 6.13 (s, 2H), 4.45 (s, 2H), 3.72 (s, 6H), 3.29 (d, *J* = 7.2 Hz, 3H). ^13 ^C NMR (101 MHz, DMSO) *δ* 166.22, 165.55, 160.82, 147.47, 146.66, 142.97, 142.42, 141.31, 133.79, 133.46, 131.72, 129.19 (d, *J* = 7.6 Hz), 127.96 (d, *J* = 5.2 Hz), 127.58, 109.81, 102.22, 98.98, 96.19, 73.49, 58.19, 55.57.

***Tert-butyl4-(4-(6-(4-(methoxymethyl)benzamido)benzo[d][1,3]dioxol-5-yl)benzoyl)piperazine-1-carboxylate(C2)***

Crystallised from EtOAc to give a white powder (0.28 g, 67%); HRMS *m/z* [M + Na]^+^ calcd for C_32_H_35_N_3_NaO_7_: 596.23727, found: 596.23071. ^1^H NMR (400 MHz, DMSO) *δ* 9.75 (s, 1H), 7.72 (d, *J* = 7.9 Hz, 2H), 7.44 (d, *J* = 8.0 Hz, 2H), 7.36 (dd, *J* = 8.2, 2.2 Hz, 4H), 7.01 (d, *J* = 15.1 Hz, 2H), 6.12 (s, 2H), 4.45 (s, 2H), 3.50 (d, *J* = 19.3 Hz, 2H), 3.34 (s, 1H), 3.31 (s, 4H), 3.29–3.25 (m, 1H), 1.41 (s, 9H). ^13 ^C NMR (101 MHz, DMSO) *δ* 169.50, 166.21, 154.26, 147.31, 146.56, 142.34, 140.88, 134.44, 133.89, 131.83, 129.26, 127.93, 127.37 (d, *J* = 12.0 Hz), 109.64, 102.15, 79.68, 73.48, 58.15, 28.49.

***4-(Methoxymethyl)-N-(6-(4-(4-(4-methoxyphenyl)piperazine-1-carbonyl)phenyl)benzo[d][1,3]dioxol-5-yl)benzamide(C3)***

Crystallised from EtOAc to give a white powder (0.3 g, 71%); HRMS *m/z* [M + Na]^+^ calcd for C_34_H_33_N_3_NaO_6_: 602.22671, found: 602.21985. ^1^H NMR (400 MHz, DMSO) *δ* 9.77 (s, 1H), 7.73 (d, *J* = 7.9 Hz, 2H), 7.45 (d, *J* = 8.1 Hz, 2H), 7.40–7.35 (m, 4H), 7.02 (d, *J* = 9.4 Hz, 2H), 6.91 (d, *J* = 9.1 Hz, 2H), 6.83 (d, *J* = 9.0 Hz, 2H), 6.12 (s, 2H), 4.44 (s, 2H), 3.70 (s, 1H), 3.69 (s, 3H), 3.66–3.35 (m, 3H), 3.28 (s, 3H), 2.97 (d, *J* = 31.4 Hz, 4H). ^13 ^C NMR (101 MHz, DMSO) *δ* 169.28, 166.22, 153.85, 147.30, 146.56, 145.57, 142.32, 140.82, 134.59, 133.89, 131.88, 129.27, 127.95, 127.38 (d, *J* = 16.0 Hz), 118.61, 114.75, 109.66, 102.14, 73.49, 58.20, 55.66, 50.45, 14.56.

***4-(Methoxymethyl)-N-(6-(4-(4-(2-methoxyphenyl)piperazine-1-carbonyl)phenyl)benzo[d][1,3]dioxol-5-yl)benzamide(C4)***

Crystallised from EtOAc to give a white powder (0.29 g, 69%); HRMS *m/z* [M + Na]^+^ calcd for C_34_H_33_N_3_NaO_6_: 602.22671, found: 602.21997. ^1^H NMR (400 MHz, DMSO) *δ* 9.77 (s, 1H), 7.73 (d, *J* = 7.9 Hz, 2H), 7.45 (d, *J* = 8.1 Hz, 2H), 7.40–7.35 (m, 4H), 7.02 (d, *J* = 9.4 Hz, 2H), 6.91 (d, *J* = 9.1 Hz, 2H), 6.83 (d, *J* = 9.0 Hz, 2H), 6.12 (s, 2H), 4.44 (s, 2H), 3.70 (s, 1H), 3.69 (s, 3H), 3.66–3.35 (m, 3H), 3.28 (s, 3H), 2.97 (d, *J* = 31.4 Hz, 4H). ^13 ^C NMR (101 MHz, DMSO) *δ* 169.25, 166.21, 152.50, 147.30, 146.55, 142.30, 141.16, 140.77, 134.64, 133.91, 131.90, 129.26, 127.93, 127.36 (d, *J* = 13.9 Hz), 123.43, 121.26, 118.88, 112.36, 109.65, 102.14, 73.46, 58.14, 55.81, 50.67.

***4-(Methoxymethyl)-N-(6-(4-(4-phenethylpiperazine-1-carbonyl)phenyl)benzo[d][1,3]dioxol-5-yl)benzamide(C5)***

Crystallised from EtOAc to give a white powder (0.24 g, 57%); HRMS *m/z* [M + H]^+^ calcd for C_35_H_36_N_3_O_5_: 578.26900, found: 578.25879. ^1^H NMR (400 MHz, DMSO) *δ* 9.74 (d, *J* = 18.3 Hz, 1H), 7.72 (d, *J* = 8.0 Hz, 2H), 7.43 (d, *J* = 8.0 Hz, 2H), 7.35 (t, *J* = 8.7 Hz, 4H), 7.27 (t, *J* = 7.5 Hz, 2H), 7.24–7.14 (m, 3H), 7.02 (d, *J* = 13.0 Hz, 2H), 6.11 (s, 2H), 4.44 (s, 2H), 3.49 (dd, *J* = 38.2, 22.8 Hz, 2H), 3.28 (s, 3H), 3.25 (s, 1H), 2.72 (dd, *J* = 17.1, 8.7 Hz, 2H), 2.54 (d, *J* = 8.5 Hz, 2H), 2.51 (d, *J* = 2.0 Hz, 1H), 2.35 (s, 3H). ^13 ^C NMR (101 MHz, DMSO) *δ* 169.20, 166.20, 147.29, 146.54, 142.30, 140.74 (d, *J* = 3.3 Hz), 134.71, 133.90, 131.89, 129.18 (d, *J* = 16.0 Hz), 128.72, 127.93, 127.44, 127.18, 126.35, 109.65, 102.14, 73.50, 59.96, 58.18, 33.04.

***4-(Methoxymethyl)-N-(6-(4-(4-phenylpiperazine-1-carbonyl)phenyl)benzo[d][1,3]dioxol-5-yl)benzamide(C6)***

Crystallised from EtOAc to give a white powder (0.23 g, 58%); HRMS *m/z* [M + H]^+^ calcd for C_33_H_32_N_3_O_5_: 550.23420, found: 550.22821. ^1^H NMR (400 MHz, DMSO) *δ* 9.75 (d, *J* = 18.1 Hz, 1H), 7.73 (d, *J* = 7.9 Hz, 2H), 7.45 (d, *J* = 8.1 Hz, 2H), 7.42–7.35 (m, 4H), 7.23 (t, *J* = 7.8 Hz, 2H), 7.02 (d, *J* = 8.8 Hz, 2H), 6.94 (d, *J* = 8.3 Hz, 2H), 6.82 (t, *J* = 7.2 Hz, 1H), 6.12 (s, 2H), 4.45 (s, 2H), 3.49 (dd, *J* = 80.3, 63.9 Hz, 4H), 3.29 (s, 3H), 3.23–2.99 (m, 4H). ^13 ^C NMR (101 MHz, DMSO) *δ* 169.32, 151.24, 147.31, 146.56, 142.32, 140.85, 134.55, 133.90, 131.88, 129.37 (d, *J* = 18.1 Hz), 127.95, 127.39 (d, *J* = 14.6 Hz), 119.90, 116.43, 109.66, 102.15, 73.50, 58.21.

***N-(6-(4-(4-(4-fluorophenyl)piperazine-1-carbonyl)phenyl)benzo[d][1,3]dioxol-5-yl)-4-(methoxymethyl)benzamide(C7)***

Crystallised from EtOAc to give a white powder (0.25 g, 60%); HRMS *m/z* [M + Na]^+^ calcd for C_33_H_30_FN_3_NaO_5_: 590.20672, found: 590.20026. ^1^H NMR (400 MHz, DMSO) *δ* 9.75 (d, *J* = 18.6 Hz, 1H), 7.73 (d, *J* = 7.9 Hz, 2H), 7.41 (dt, *J* = 16.0, 8.0 Hz, 6H), 7.01 (tt, *J* = 9.1, 6.8 Hz, 6H), 6.12 (s, 2H), 4.44 (s, 2H), 3.54 (dd, *J* = 79.1, 41.0 Hz, 4H), 3.28 (s, 3H), 3.09 (d, *J* = 16.0 Hz, 4H). ^13 ^C NMR (101 MHz, DMSO) *δ* 169.30, 166.22, 158.00, 155.65, 148.15, 147.31, 146.56, 142.32, 140.86, 134.53, 133.89, 131.87, 129.27, 127.95, 127.39 (d, *J* = 15.4 Hz), 118.33 (d, *J* = 7.5 Hz), 115.93, 115.71, 109.66, 102.15, 73.49, 60.23, 58.20, 49.75, 14.56.

***N-(6-(4-(4-(4-chlorophenyl)piperazine-1-carbonyl)phenyl)benzo[d][1,3]dioxol-5-yl)-4-(methoxymethyl)benzamide(C8)***

Crystallised from EtOAc to give a white powder (0.3 g, 70%); HRMS *m/z* [M + H]^+^ calcd for C_33_H_31_ClN_3_O_5_: 584.19522, found: 584.18884. ^1^H NMR (400 MHz, DMSO) *δ* 9.76 (d, *J* = 18.8 Hz, 1H), 7.73 (d, *J* = 7.9 Hz, 2H), 7.45 (d, *J* = 8.1 Hz, 2H), 7.42–7.35 (m, 4H), 7.25 (d, *J* = 8.9 Hz, 2H), 7.02 (d, *J* = 8.6 Hz, 2H), 6.95 (d, *J* = 8.9 Hz, 2H), 6.12 (s, 2H), 4.44 (s, 2H), 3.80–3.36 (m, 4H), 3.29 (s, 3H), 3.13 (d, *J* = 15.7 Hz, 4H). ^13 ^C NMR (101 MHz, DMSO) *δ* 169.33, 166.22, 150.04, 147.31, 146.57, 142.32, 140.88, 134.48, 133.89, 131.86, 129.21 (d, *J* = 11.7 Hz), 127.95, 127.40 (d, *J* = 14.7 Hz), 123.37, 117.84, 109.66, 102.15, 73.50, 58.21, 48.66.

### 4-(Methoxymethyl)-N-(6-(4-((4-(4-methylpiperazin-1-yl)phenyl)carbamoyl)phenyl)benzo[d][1,3]dioxol-5-yl)benzamide(C9)

Crystallised from EtOAc to give a white powder (0.29 g, 68%); HRMS *m/z* [M + H]^+^ calcd for C_34_H_35_N_4_O_5_: 579.26075, found: 579.25372. ^1^H NMR (400 MHz, DMSO) *δ* 10.01 (s, 1H), 9.83 (s, 1H), 7.92 (d, *J* = 8.2 Hz, 2H), 7.79 (d, *J* = 8.0 Hz, 2H), 7.59 (d, *J* = 8.9 Hz, 2H), 7.53 (d, *J* = 8.2 Hz, 2H), 7.38 (d, *J* = 8.0 Hz, 2H), 7.03 (d, *J* = 8.5 Hz, 2H), 6.90 (d, *J* = 9.0 Hz, 2H), 6.13 (s, 2H), 4.45 (s, 2H), 3.29 (d, *J* = 6.6 Hz, 3H), 3.12–3.05 (m, 4H), 2.48–2.40 (m, 4H), 2.21 (s, 3H). ^13 ^C NMR (101 MHz, DMSO) *δ* 166.22, 164.89, 147.97, 147.42, 146.64, 142.61, 142.41, 133.76 (d, *J* = 7.8 Hz), 131.77, 131.44, 129.15 (d, *J* = 9.7 Hz), 127.89 (d, *J* = 17.6 Hz), 127.57, 121.95, 115.89, 109.81, 102.20, 73.48, 58.19, 55.11, 48.95, 46.25.

### N-isopropyl-4-(6-(4-(methoxymethyl)benzamido)benzo[d][1,3]dioxol-5-yl)benzamide(C10)

Crystallised from EtOAc to give a white powder (0.25 g, 76%); HRMS *m/z* [M + H]^+^ calcd for C_26_H_27_N_2_O_5_: 477.19200, found: 447.18665. ^1^H NMR (400 MHz, DMSO) *δ* 9.80 (s, 1H), 8.17 (d, *J* = 7.7 Hz, 1H), 7.79 (dd, *J* = 12.9, 8.1 Hz, 4H), 7.46 (d, *J* = 8.2 Hz, 2H), 7.38 (d, *J* = 8.0 Hz, 2H), 7.00 (d, *J* = 13.1 Hz, 2H), 6.12 (s, 2H), 4.45 (s, 2H), 3.30 (s, 3H), 1.14 (d, *J* = 6.6 Hz, 5H). ^13 ^C NMR (101 MHz, DMSO) *δ* 166.17, 165.35, 147.34, 146.61, 142.31 (d, *J* = 18.8 Hz), 133.76, 133.47, 131.84, 129.12, 128.92, 127.95, 127.53 (d, *J* = 7.3 Hz), 109.79, 102.17, 73.48, 60.23, 58.19, 41.40, 22.79, 21.23, 14.56.

#### PCSK9-LDLR TR-FRET assay

The compounds were dissolved in 100% DMSO; then 2 µL of the dilution was added to a 20 µL of reaction to keep final concentration of DMSO less than 1% in all of reactions. The binding reaction was conducted at room temperature. The reaction mixture in assay buffer contains PCSK9, the indicated amount of the inhibitor, ligand LDLR, and the reaction dyes. The reaction mixture was incubated for 120 min before detection of the TR-FRET signal. Fluorescence signals for both the donor and acceptor dyes were measured using a Tecan Infinite M1000 plate reader. TR-FRET was recorded as the ratio of the fluorescence of the acceptor and the donor dyes (acceptor/donor). The TR-FRET data were analysed using Graphpad Prism software.

#### Molecular docking

Molecular docking was performed using Glide in Schrodinger Suites 2018. Crystal structure of PCSK9 (PDB Entry: 3GCX) was derived from RCSB protein data bank (www.rcsb.org). Structural modifications were performed by Protein Preparation Wizard. The LDLR chain and embedded water molecules in the protein structure were removed. The default OPLS3 force field was assigned to the refined protein. The structure of ligand **B14** was sketched by maestro and prepared by LigPrep. The active site was set to be an enclosing box centred on residue Phe379 with side length of 20 Å. Extra precision was selected for the docking process, and other parameters were set as default.

#### Cell culture and ICW assay

The HepG2 cell line was grown in a humidified incubator with 5% CO_2_ at 37 °C and cultured in DMEM-High glucose medium with 10% (v/v) heat-inactivation foetal bovine serum, 2 mM L-glutamine and 100 U/ml penicillin-streptomycin. The expression level of LDLR in HepG2 cells was determined by In-Cell Western (ICW) Assay as described previously^16^. Briefly, cells were plated in a 96-well plate (2 × 10^4^ cells per well) and cultured for more than 24 h, followed by washing with PBS and starving overnight in DMEM without FBS. HepG2 cells were treated with 4.0 μg/ml PCSK9 (K9) alone and the tested compounds with the presence of 4.0 μg/ml of K9, and vehicle (PBS) for 8 h. Then cells were washed with PBS one time and fixed in 4% paraformaldehyde for 20 min. Cells were washed 5 times with PBS, then were blocked with 5% BSA in PBS for 1 h. LDLR monoclonal Antibody solution (1:1000 in 5% BSA in PBS, 50.0 μL/well) was incubated overnight at 4 °C. Subsequently, the cells were washed 5 times with PBS. Goat anti-mouse IgG-HRP secondary antibody solution (1:2000 in 5% BSA in PBS, 50.0 μL/well) was added and incubated 1 h at RT. Then cells were washed 5 times with PBS and added with TMB substrate, then incubated at RT until desired colour was developed. 50.0 μL/well of 2 M H_2_SO_4_ were added to stop the reaction and the absorbance at 450 nm was measured using a plate reader (EnSpire, Perkin Elmer Corporation).

#### Fluorescent LDL uptake assay

HepG2 cells (2 × 10^4^ cells per well) were seeded in black 96-well plates and cultured for more than 24 h. The following day, cells were treated with 4.0 μg/ml K9 alone and the tested compounds with the presence of 4.0 μg/ml of K9, and vehicle (PBS) for 8 h. At the end of the treatments, the culture medium was replaced with 50.0 μl/well LDL-DyLight™ 550 working solution (Cayman Chemical Company, US). The cells were additionally incubated for 16 h at 37 °C. Then the cells were washed with PBS one time, then added PBS with 100.0 μl/well. The degree of LDL uptake was measured using a plate reader (EnSpire, Perkin Elmer Corporation) at excitation wavelengths 540 nm and emission wavelengths 570 nm.

#### Statistical analysis

All experiments were repeated at least three times unless otherwise stated. The data were represented as mean ± SD. Statistical analysis were performed with Student’s t test for two group comparisons and using one-way ANOVA with Tukey’s *post hoc* test for multigroup comparisons. *p* < 0.05 or *p* < 0.01 were considered statistically significant.

## Supplementary Material

Supplemental Material
