# Supplementary material for: Discovery and SAR analysis of phenylbenzo[d][1,3]dioxole-based proprotein convertase subtilisin/kexin type 9 inhibitors
Source: J Enzyme Inhib Med Chem. 2022 Jul 19;37(1):2017–35. doi: 10.1080/14756366.2022.2101645 (PMC9307114; doi:10.1080/14756366.2022.2101645)
Supplement: Supplemental Material [file IENZ_A_2101645_SM1915.pdf]

Discovery and SAR analysis of Phenylbenzo[d][1,3]dioxole-based Proprotein Convertase Subtilisin/Kexin type 9 inhibitors

Fahui Li[a†], Lihui Zhang[b†], Jinhong Feng[c]* and Lei Zhang[a]*

[a] *Department of Medicinal Chemistry, School of Pharmacy, Weifang Medical University, Weifang, Shandong, China;*

[b] *School of Stomatology, Weifang Medical University, Weifang, Shandong, China;*

[c] *Shandong Analysis and Test Center, Qilu University of Technology (Shandong Academy of sciences), Jinan, Shandong, China.*

[†]*These authors have contributed equally to this work.*

Author for correspondence: Jinhong Feng, Tel./fax: +86-531-68606193, E-mail: fengjinhong520@163.com; Lei Zhang, Tel./fax: +86-536-8462014, E-mail: leizhangchemical@gmail.com.

Contents

HRMS and ^{1}H-NMR spectrum of **A1**....................4

^{13}C-NMR spectrum of **A1** and HRMS spectrum of **A2**....................5

^{1}H-NMR spectrum and ^{13}C-NMR of **A2**....................6

HRMS and ^{1}H-NMR spectrum of **A3**....................7

^{13}C-NMR spectrum of **A3** and HRMS spectrum of **A4**....................8

^{1}H-NMR spectrum and ^{13}C-NMR of **A4**....................9

HRMS and ^{1}H-NMR spectrum of **A5**....................10

^{13}C-NMR spectrum of **A5** and HRMS spectrum of **A6**....................11

^{1}H-NMR spectrum and ^{13}C-NMR of **A6**....................12

HRMS and ^{1}H-NMR spectrum of **A7**....................13

^{13}C-NMR spectrum of **A7** and HRMS spectrum of **A8**....................14

^{1}H-NMR spectrum and ^{13}C-NMR of **A8**....................15

HRMS and ^{1}H-NMR spectrum of **A9**....................16

^{13}C-NMR spectrum of **A9** and HRMS spectrum of **A10**....................17

^{1}H-NMR spectrum and ^{13}C-NMR of **A10**....................18

HRMS and ^{1}H-NMR spectrum of **A11**....................19

^{13}C-NMR spectrum of **A11** and HRMS spectrum of **A12**....................20

^{1}H-NMR spectrum and ^{13}C-NMR of **A12**....................21

HRMS and ^{1}H-NMR spectrum of **A13**....................22

^{13}C-NMR spectrum of **A13** and HRMS spectrum of **A14**....................23

^{1}H-NMR spectrum and ^{13}C-NMR of **A14**....................24

HRMS and ^{1}H-NMR spectrum of **A15**....................25

^{13}C-NMR spectrum of **A15** and HRMS spectrum of **A16**....................26

^{1}H-NMR spectrum and ^{13}C-NMR of **A16** 27

HRMS and ^{1}H-NMR spectrum of **A17** 28

^{13}C-NMR spectrum of **A17** and HRMS spectrum of **B1** 29

^{1}H-NMR spectrum and ^{13}C-NMR of **B1** 30

HRMS and ^{1}H-NMR spectrum of **B2** 31

^{13}C-NMR spectrum of **B2** and HRMS spectrum of **B3** 32

^{1}H-NMR spectrum and ^{13}C-NMR of **B3** 33

HRMS and ^{1}H-NMR spectrum of **B4** 34

^{13}C-NMR spectrum of **B4** and HRMS spectrum of **B5** 35

^{1}H-NMR spectrum and ^{13}C-NMR of **B5** 36

HRMS and ^{1}H-NMR spectrum of **B6** 37

^{13}C-NMR spectrum of **B6** and HRMS spectrum of **B7** 38

^{1}H-NMR spectrum and ^{13}C-NMR of **B7** 39

HRMS and ^{1}H-NMR spectrum of **B8** 40

^{13}C-NMR spectrum of **B8** and HRMS spectrum of **B9** 41

^{1}H-NMR spectrum and ^{13}C-NMR of **B9** 42

HRMS and ^{1}H-NMR spectrum of **B10** 43

^{13}C-NMR spectrum of **B10** and HRMS spectrum of **B11** 44

^{1}H-NMR spectrum and ^{13}C-NMR of **B11** 45

HRMS and ^{1}H-NMR spectrum of **B12** 46

^{13}C-NMR spectrum of **B12** and HRMS spectrum of **B13** 47

^{1}H-NMR spectrum and ^{13}C-NMR of **B13** 48

HRMS and ^{1}H-NMR spectrum of **B14** 49

^{13}C-NMR spectrum of **B14** and HRMS spectrum of **B15** 50

^{1}H-NMR spectrum and ^{13}C-NMR of **B15** 51

HRMS and ^{1}H-NMR spectrum of **B16**....................52

^{13}C-NMR spectrum of **B16** and HRMS spectrum of **B17** 53

^{1}H-NMR spectrum and ^{13}C-NMR of **B17** 54

HRMS and ^{1}H-NMR spectrum of **B18**....................55

^{13}C-NMR spectrum of **B18** and HRMS spectrum of **B19** 56

^{1}H-NMR spectrum and ^{13}C-NMR of **B19** 57

HRMS and ^{1}H-NMR spectrum of **B20**....................58

^{13}C-NMR spectrum of **B20** and HRMS spectrum of **C1**....................59

^{1}H-NMR spectrum and ^{13}C-NMR of **C1** 60

HRMS and ^{1}H-NMR spectrum of **C2**....................61

^{13}C-NMR spectrum of **C2** and HRMS spectrum of **C3**....................62

^{1}H-NMR spectrum and ^{13}C-NMR of **C3** 63

HRMS and ^{1}H-NMR spectrum of **C4**....................64

^{13}C-NMR spectrum of **C4** and HRMS spectrum of **C5**....................65

^{1}H-NMR spectrum and ^{13}C-NMR of **C5** 66

HRMS and ^{1}H-NMR spectrum of **C6**....................67

^{13}C-NMR spectrum of **C6** and HRMS spectrum of **C7**....................68

^{1}H-NMR spectrum and ^{13}C-NMR of **C7** 69

HRMS and ^{1}H-NMR spectrum of **C8**....................70

^{13}C-NMR spectrum of **C8** and HRMS spectrum of **C9**....................71

^{1}H-NMR spectrum and ^{13}C-NMR of **C9** 72

HRMS and ^{1}H-NMR spectrum of **C10**....................73

^{13}C-NMR spectrum of **C10** 74

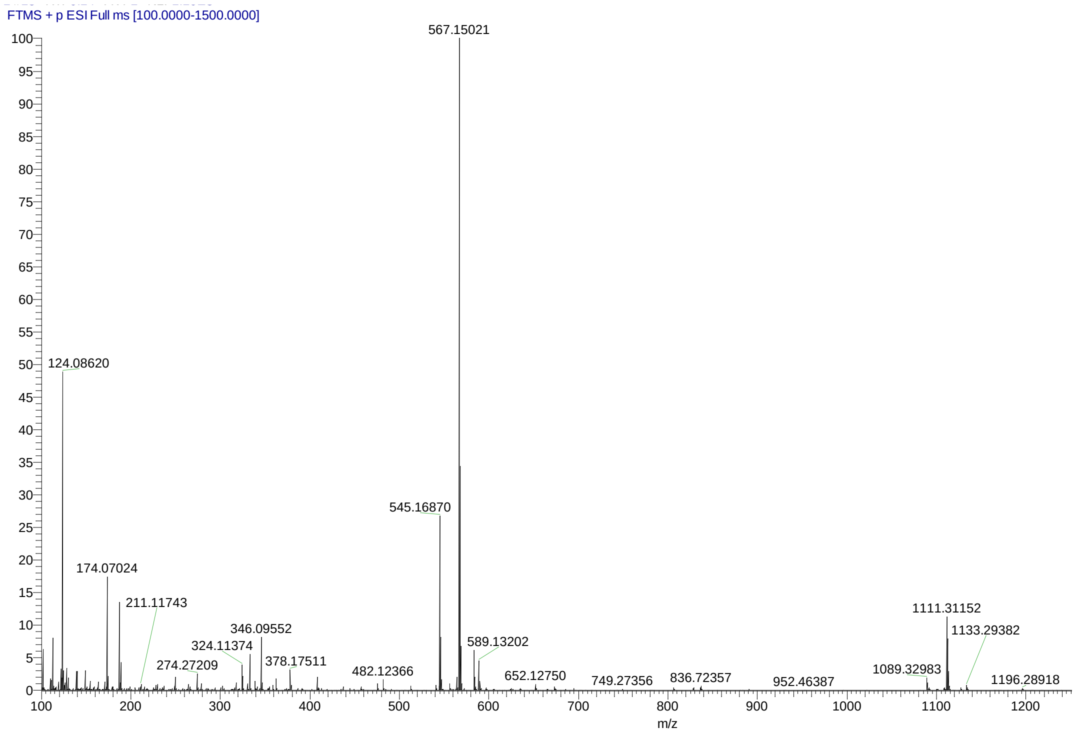

HRMS spectrum of **A1**

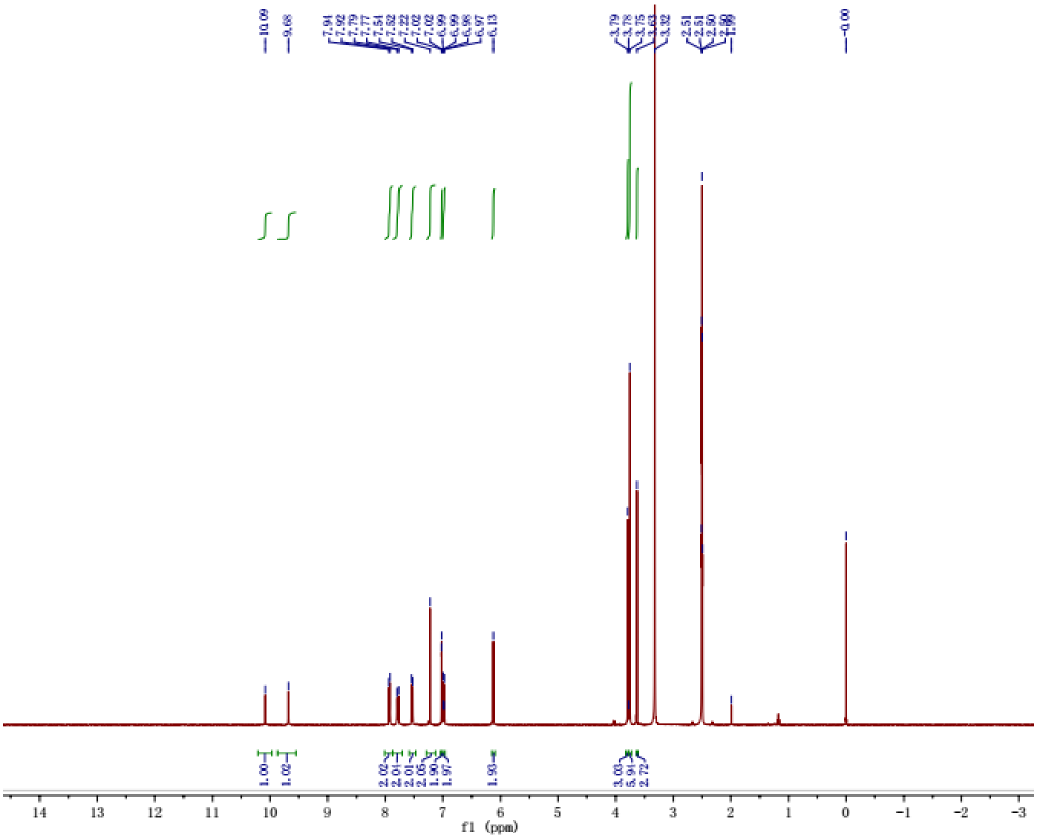

^{1}H-NMR spectrum of **A1**

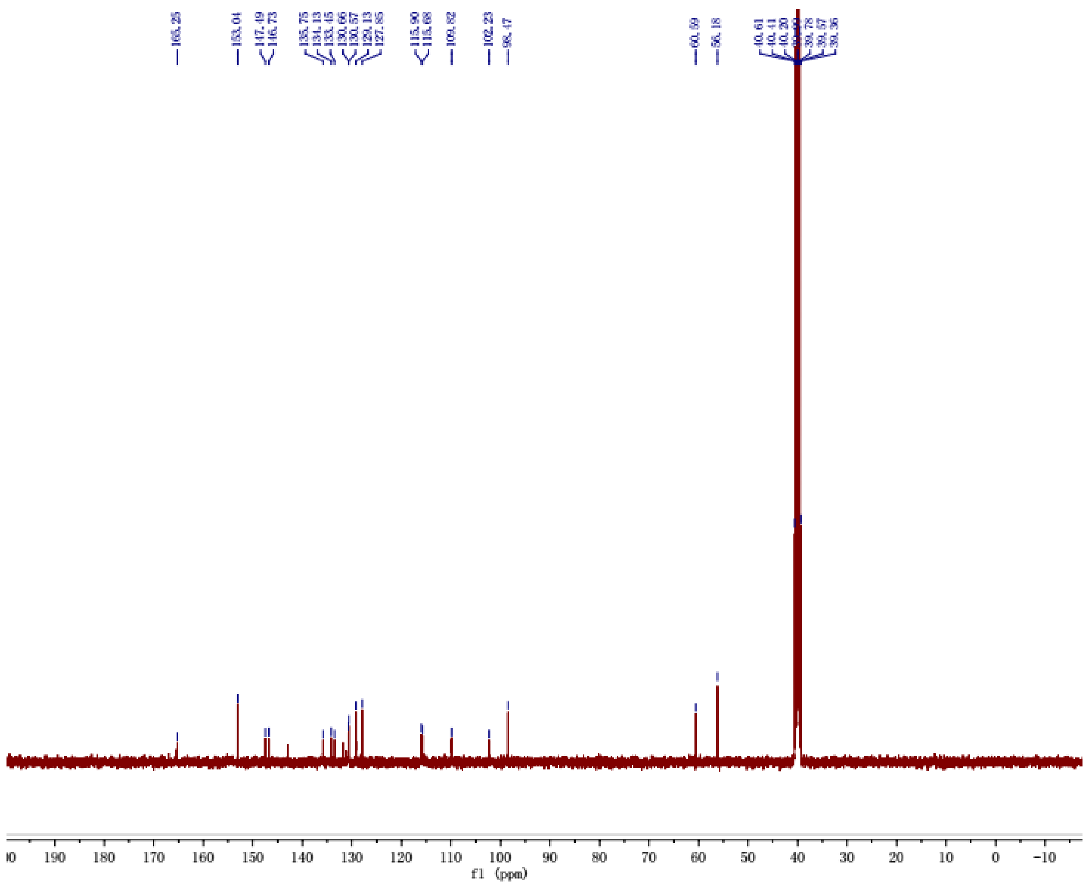

^{13}C-NMR spectrum of **A1**

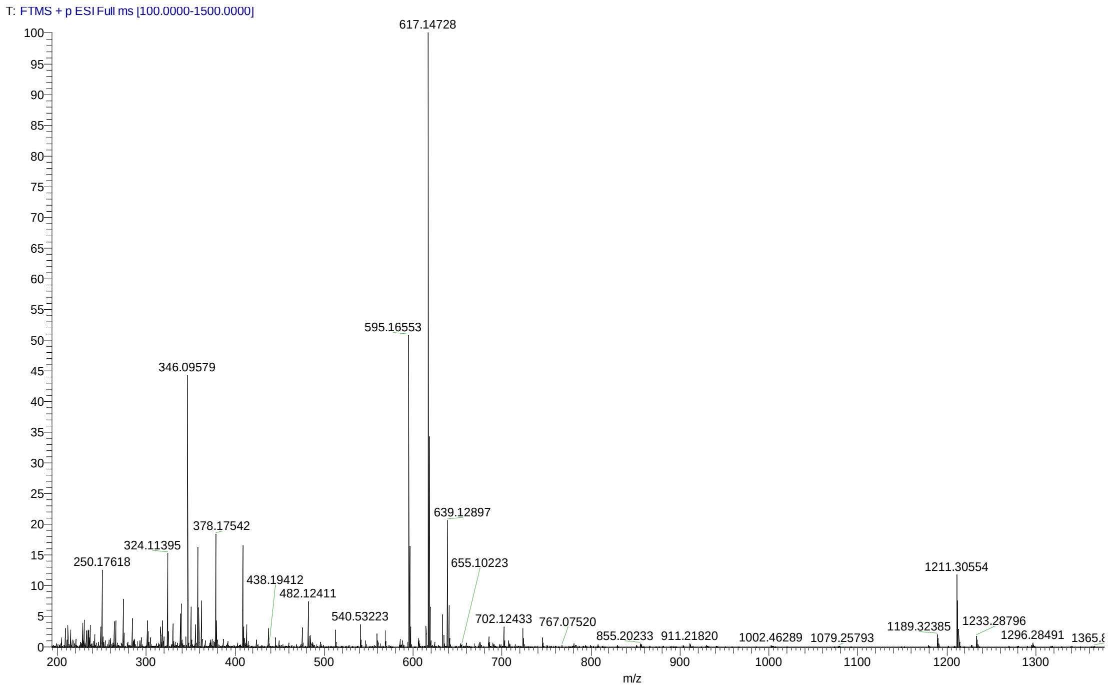

HRMS spectrum of **A2**

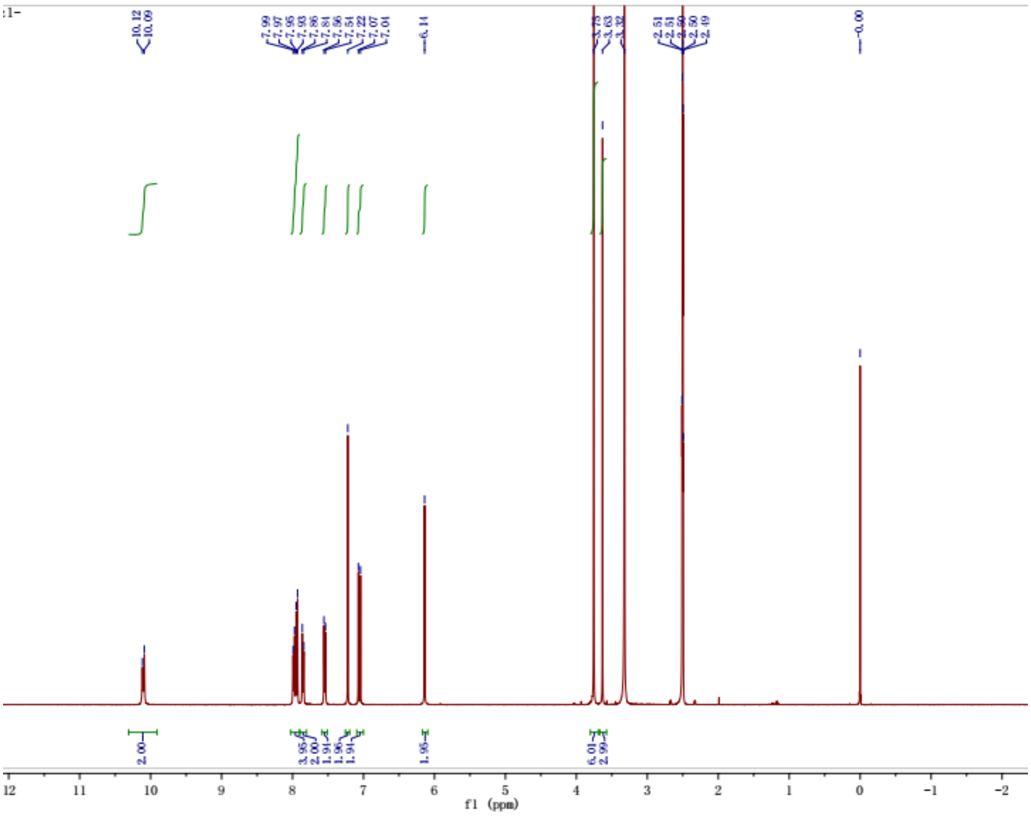

^{1}H-NMR spectrum of **A2**

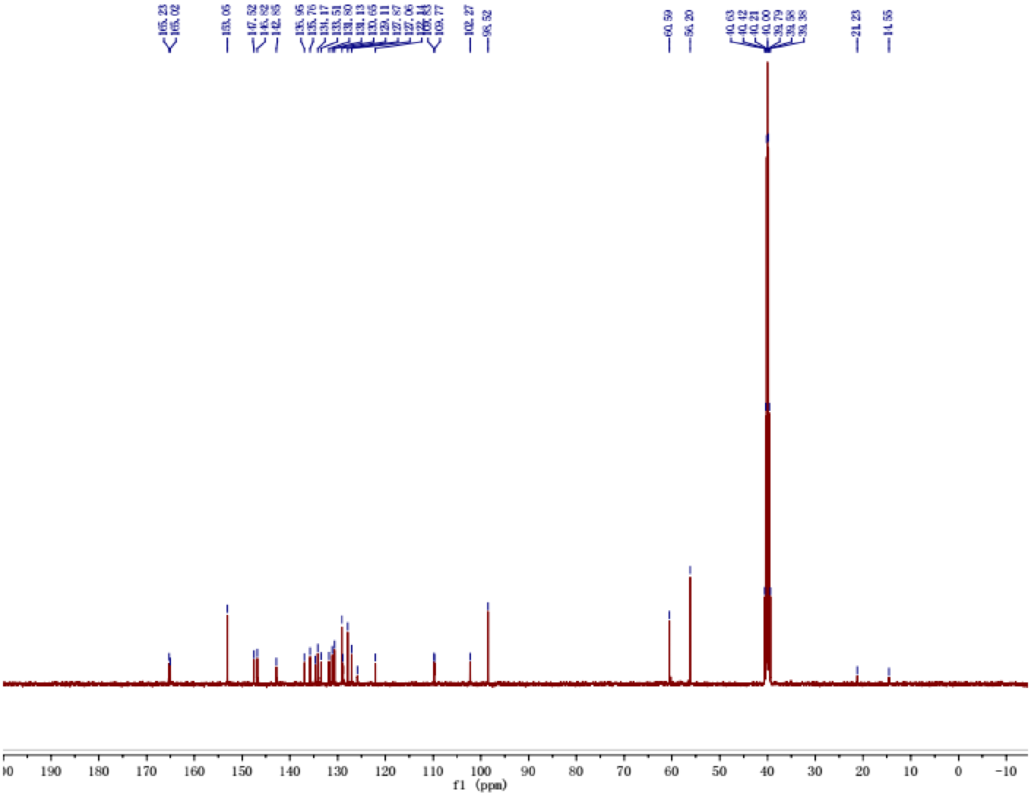

^{13}C-NMR spectrum of **A2**

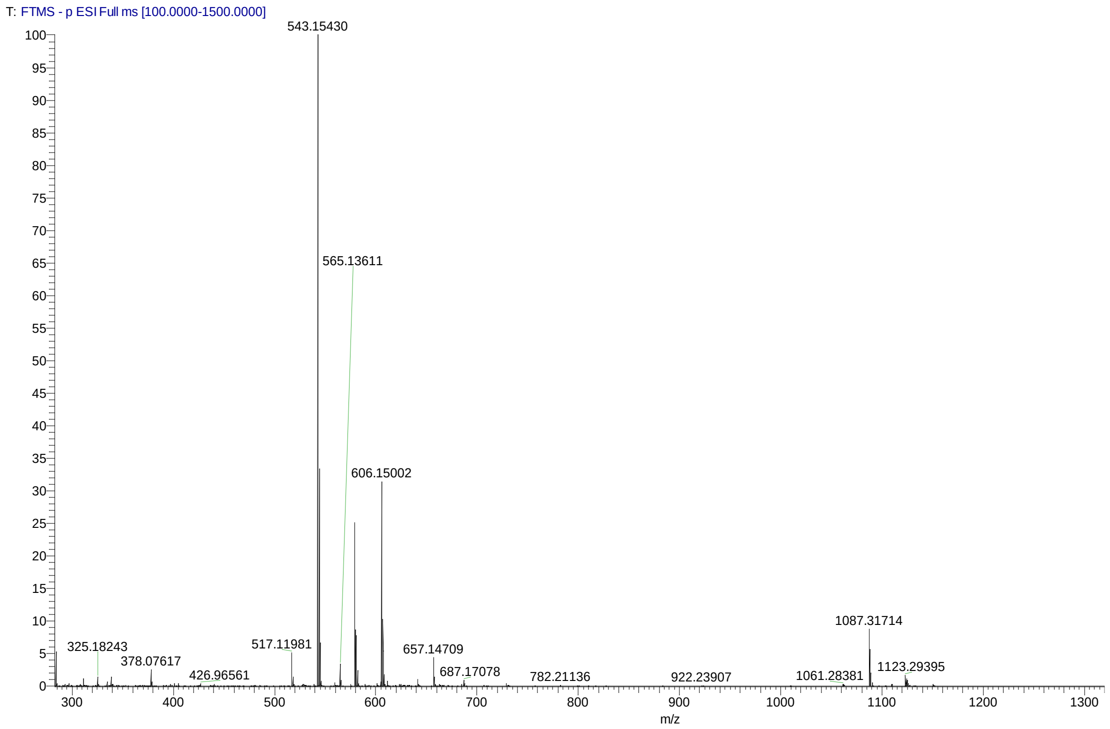

HRMS spectrum of **A3**

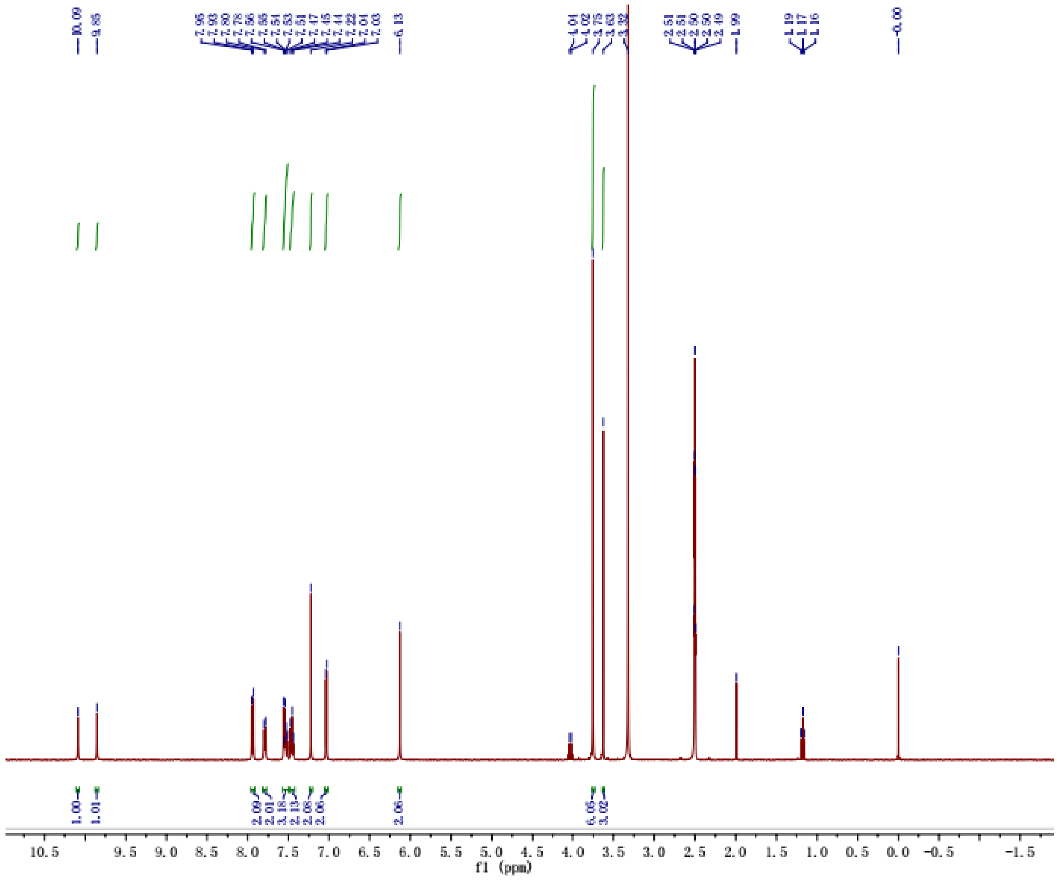

^{1}H-NMR spectrum of **A3**

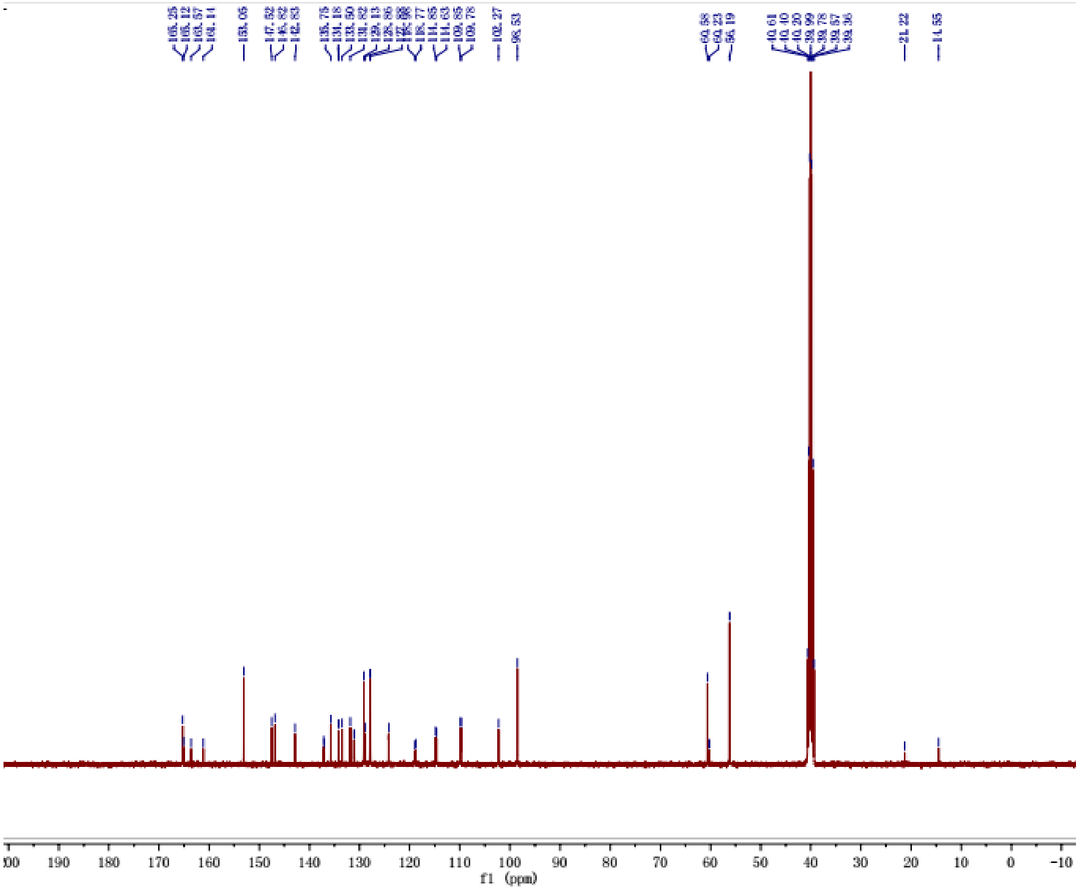

^{13}C-NMR spectrum of **A3**

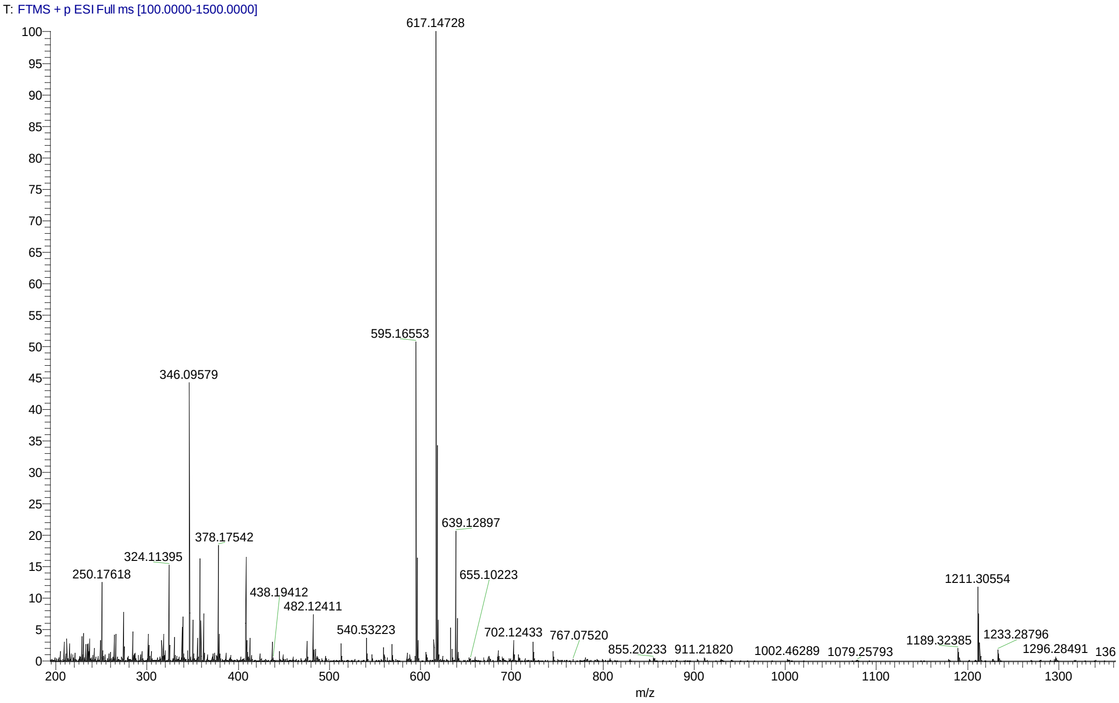

HRMS spectrum of **A4**

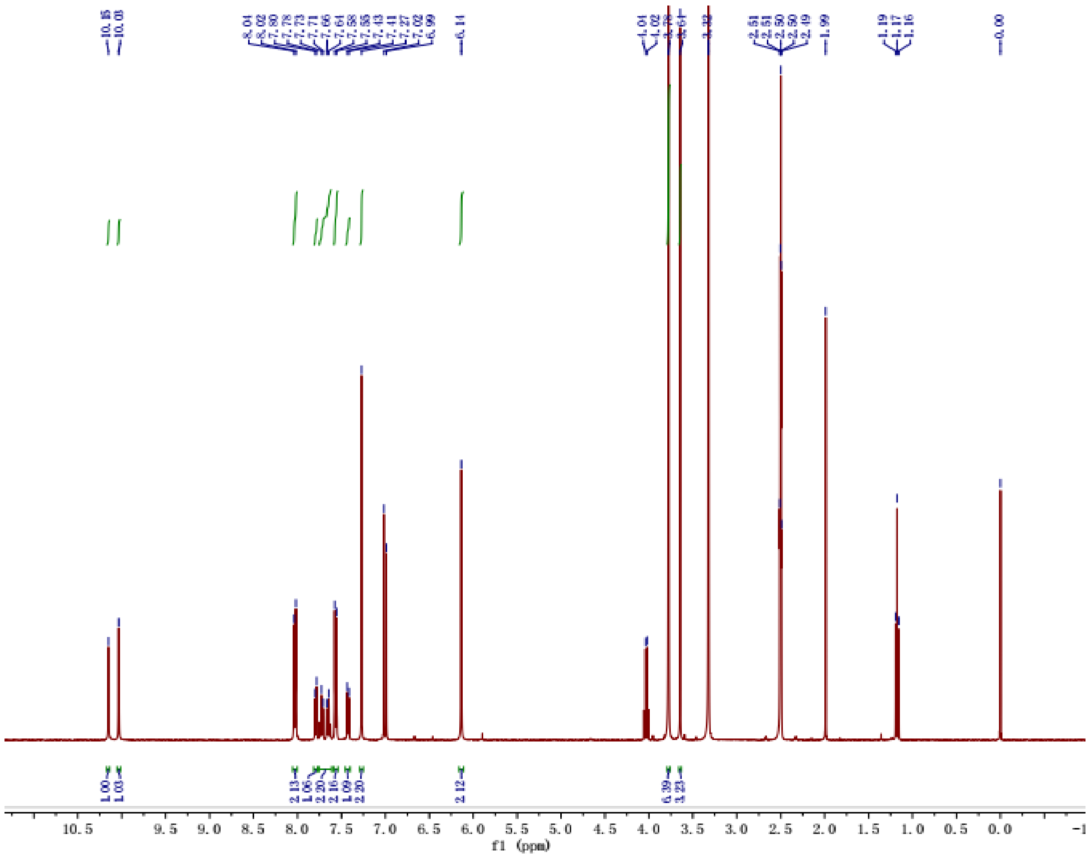

^{1}H-NMR spectrum of **A4**

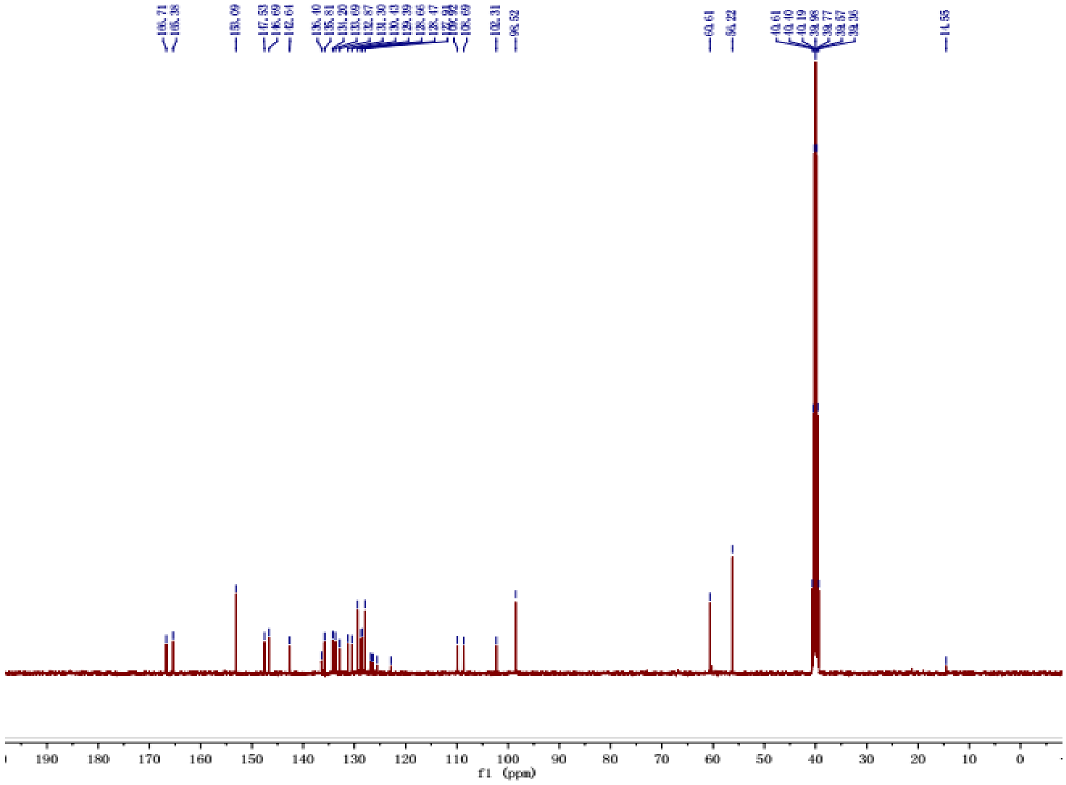

^{13}C-NMR spectrum of **A4**

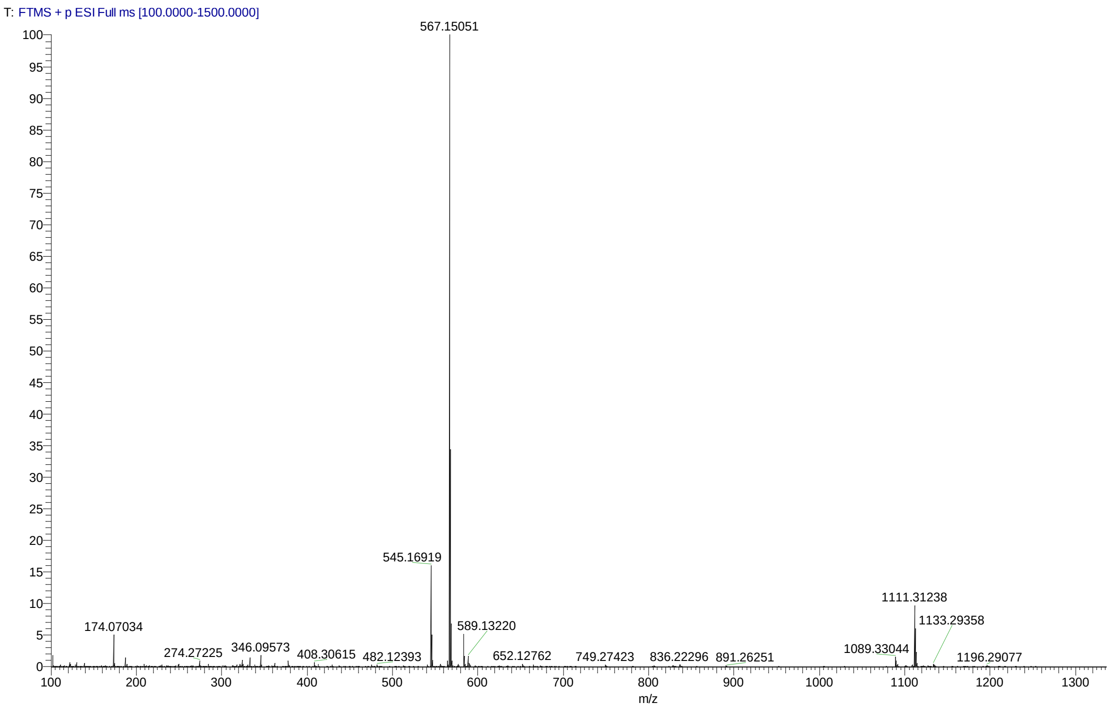

HRMS spectrum of **A5**

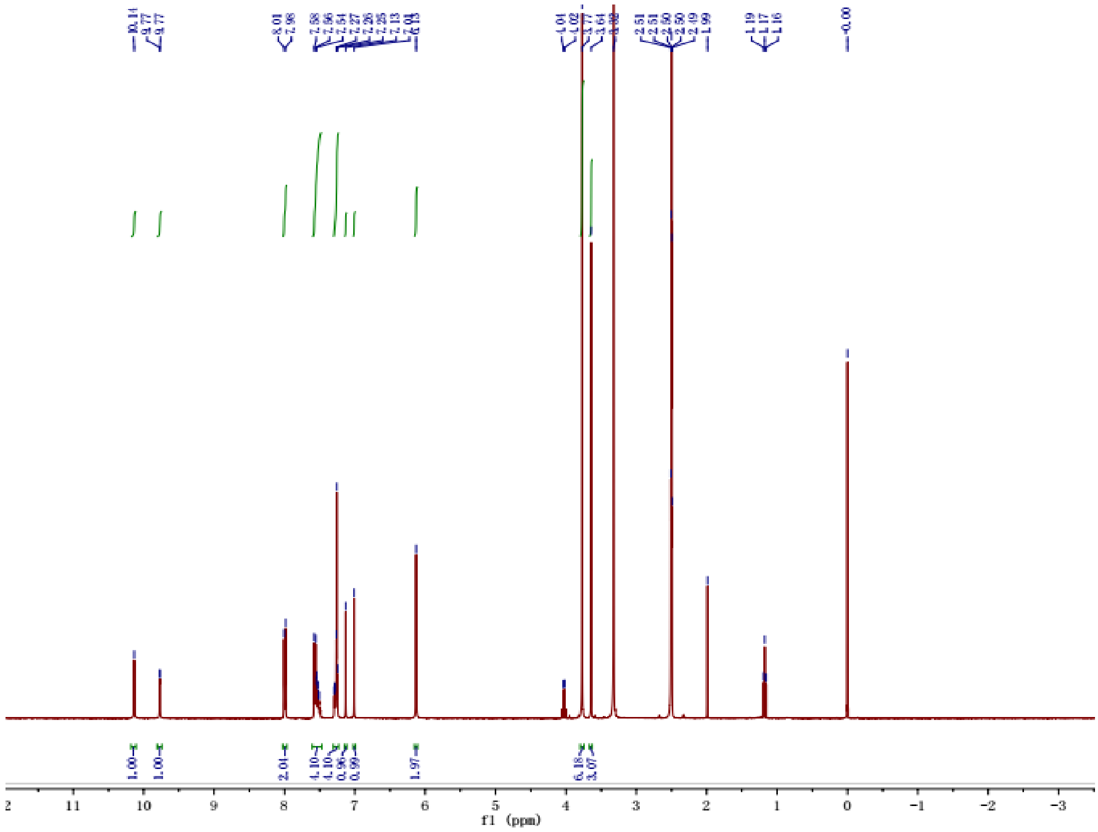

^{1}H-NMR spectrum of **A5**

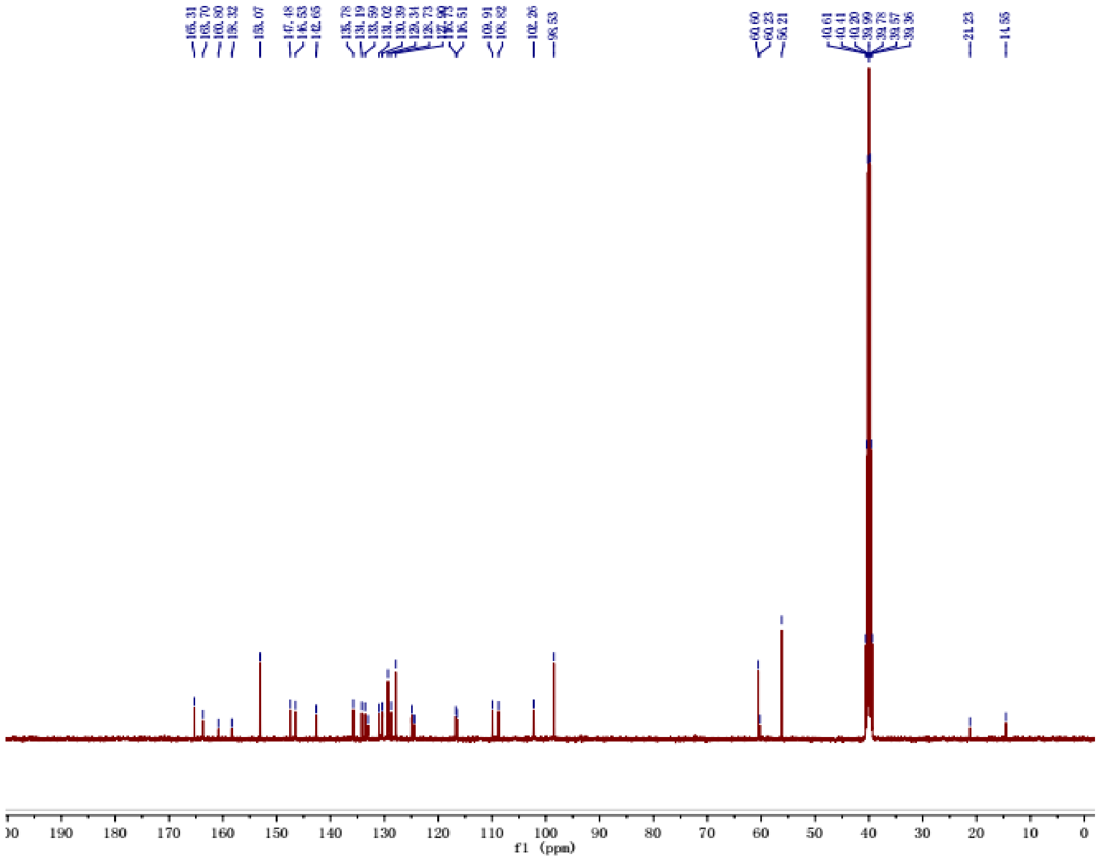

^{13}C-NMR spectrum of **A5**

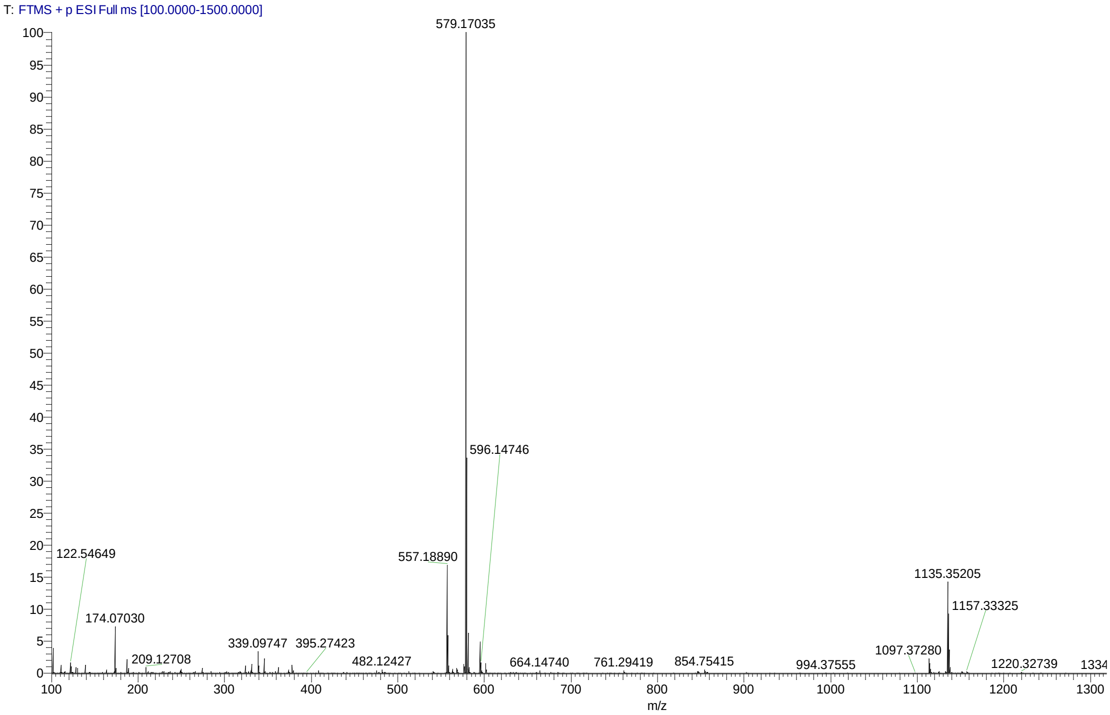

HRMS spectrum of **A6**

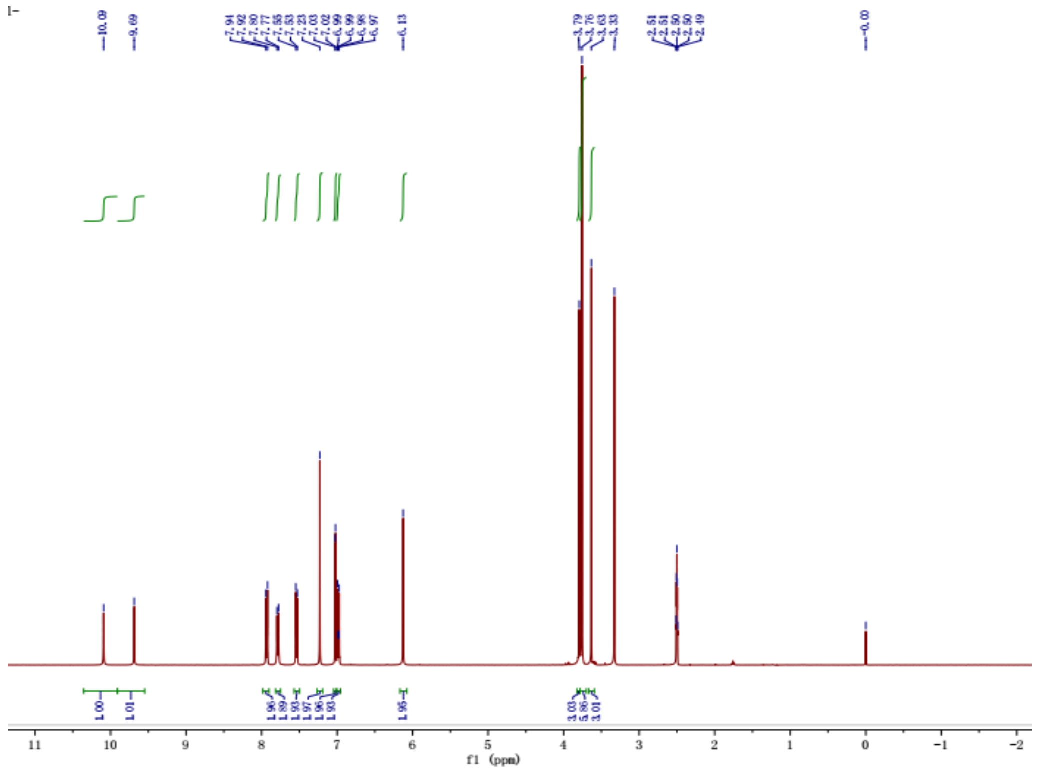

^{1}H-NMR spectrum of **A6**

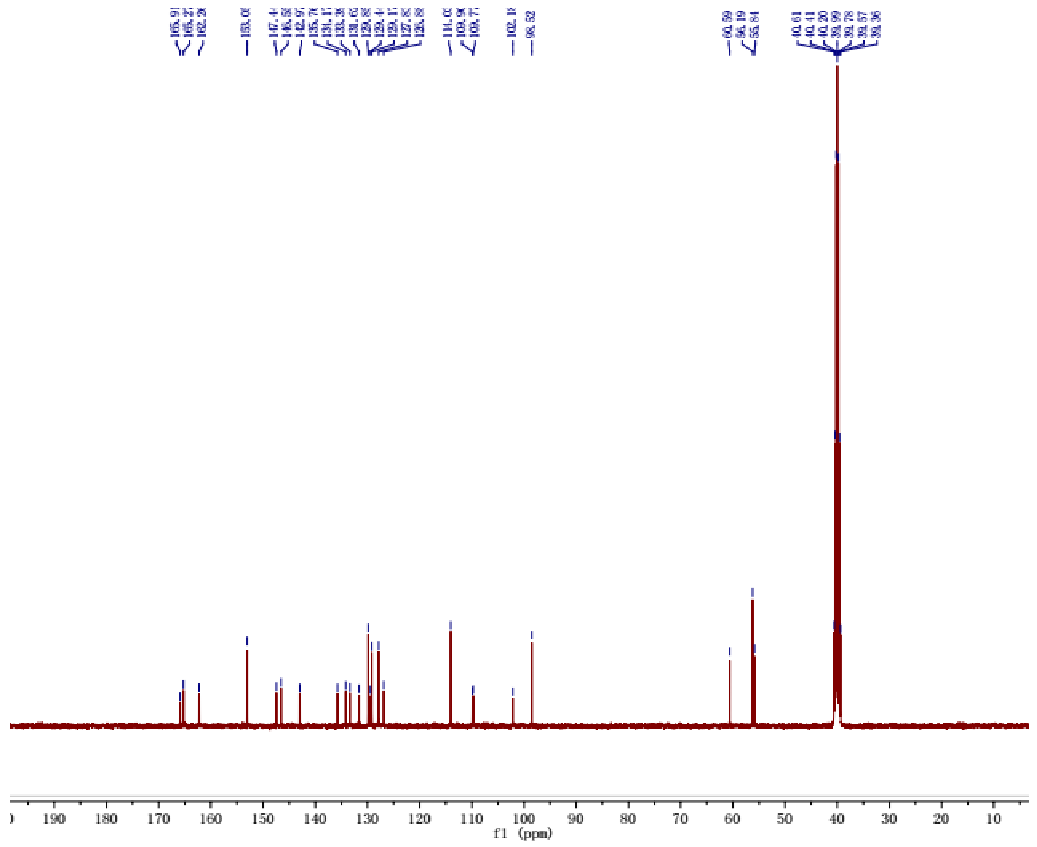

^{13}C-NMR spectrum of **A6**

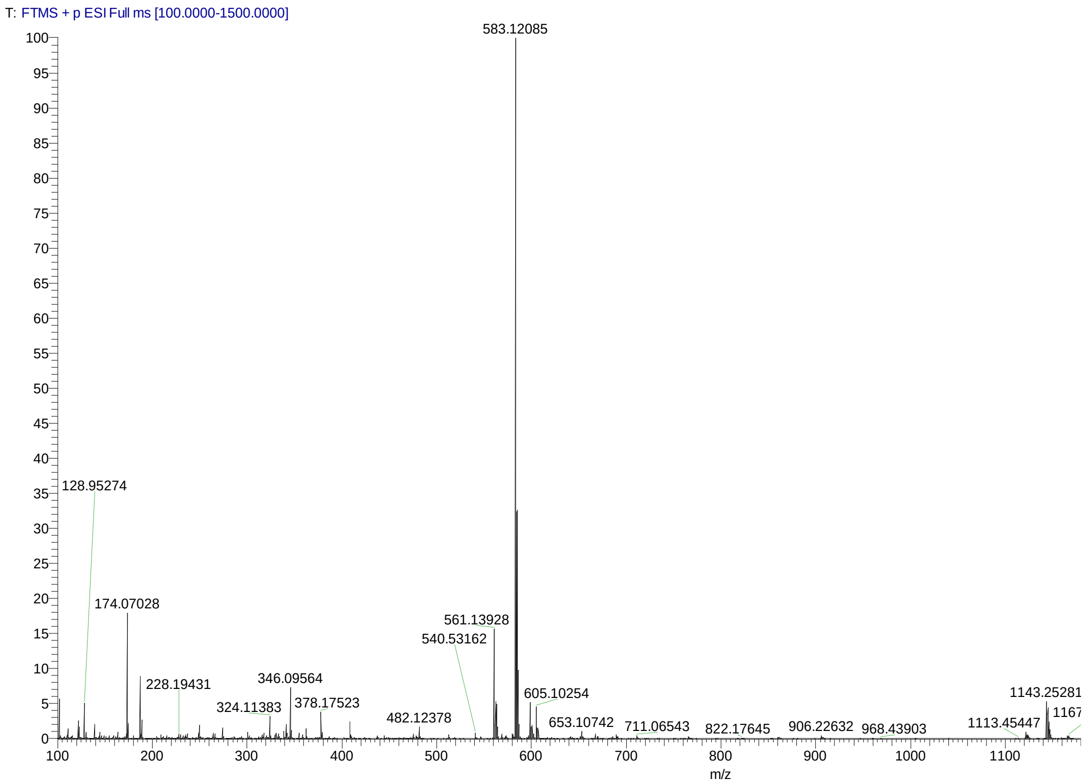

HRMS spectrum of **A7**

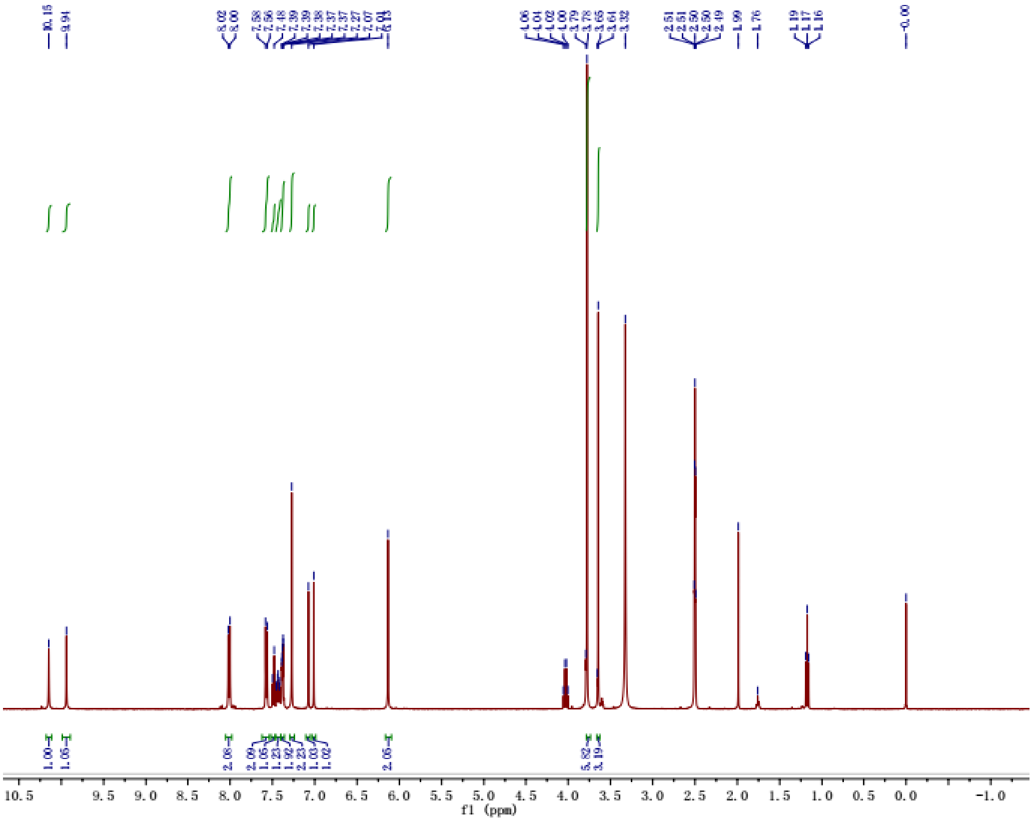

^{1}H-NMR spectrum of **A7**

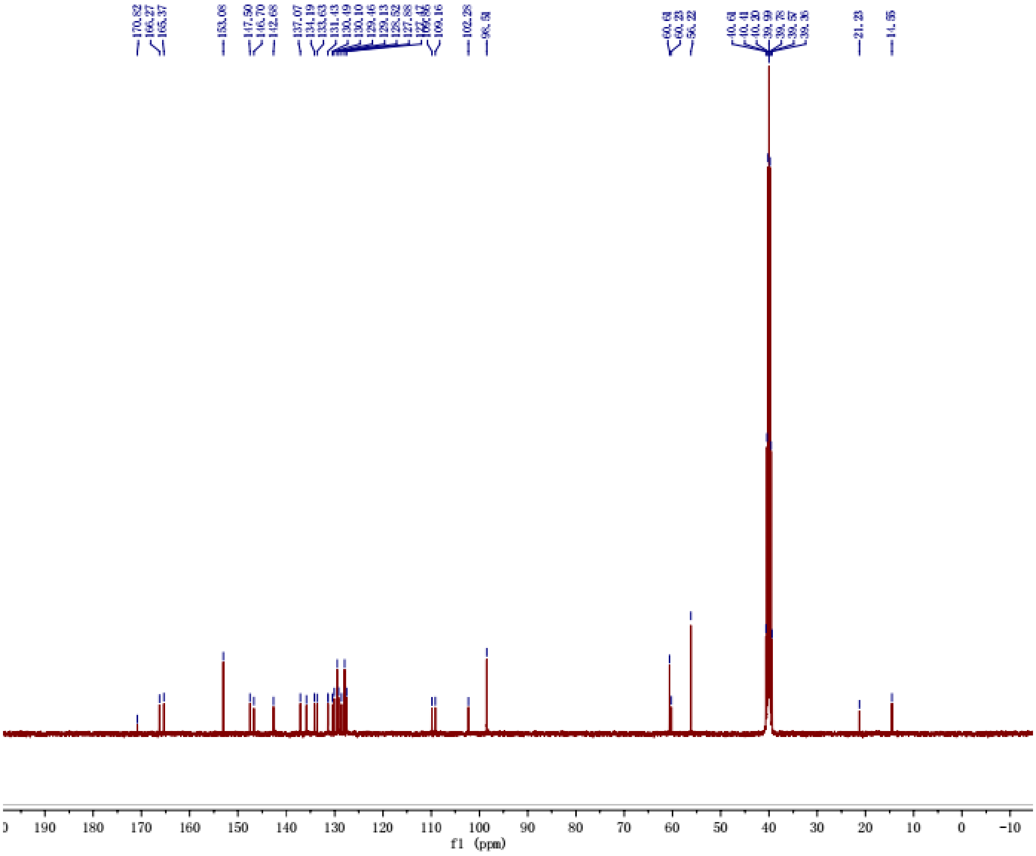

^{13}C-NMR spectrum of **A7**

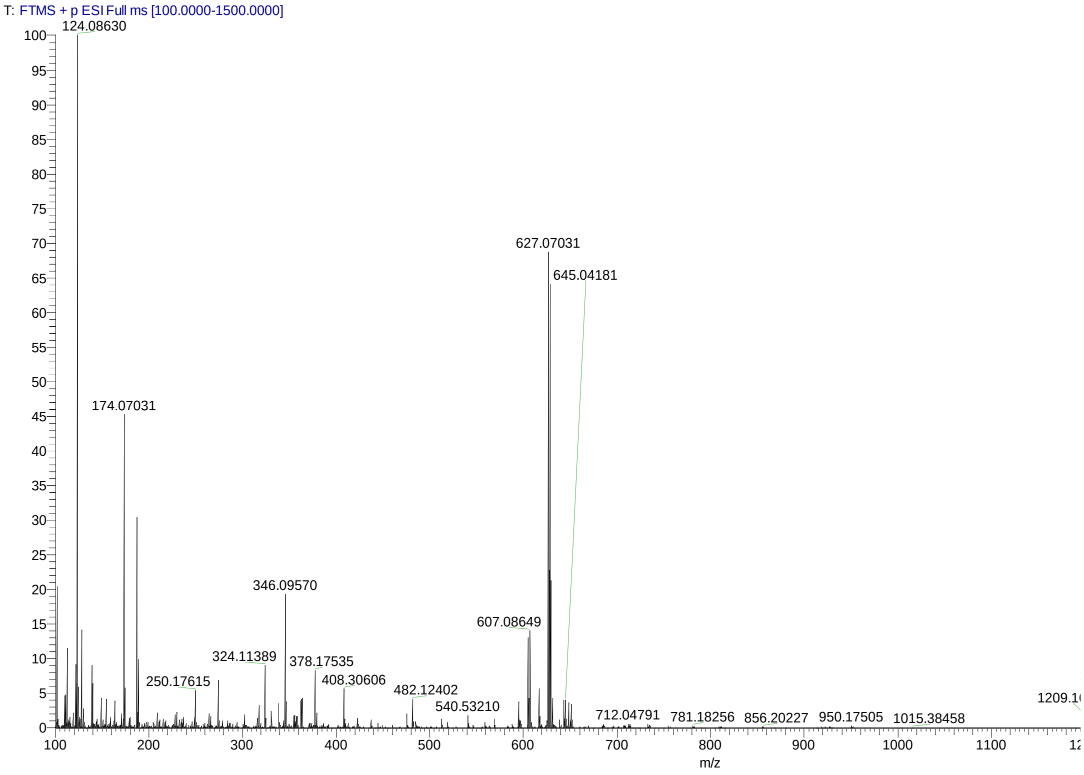

HRMS spectrum of **A8**

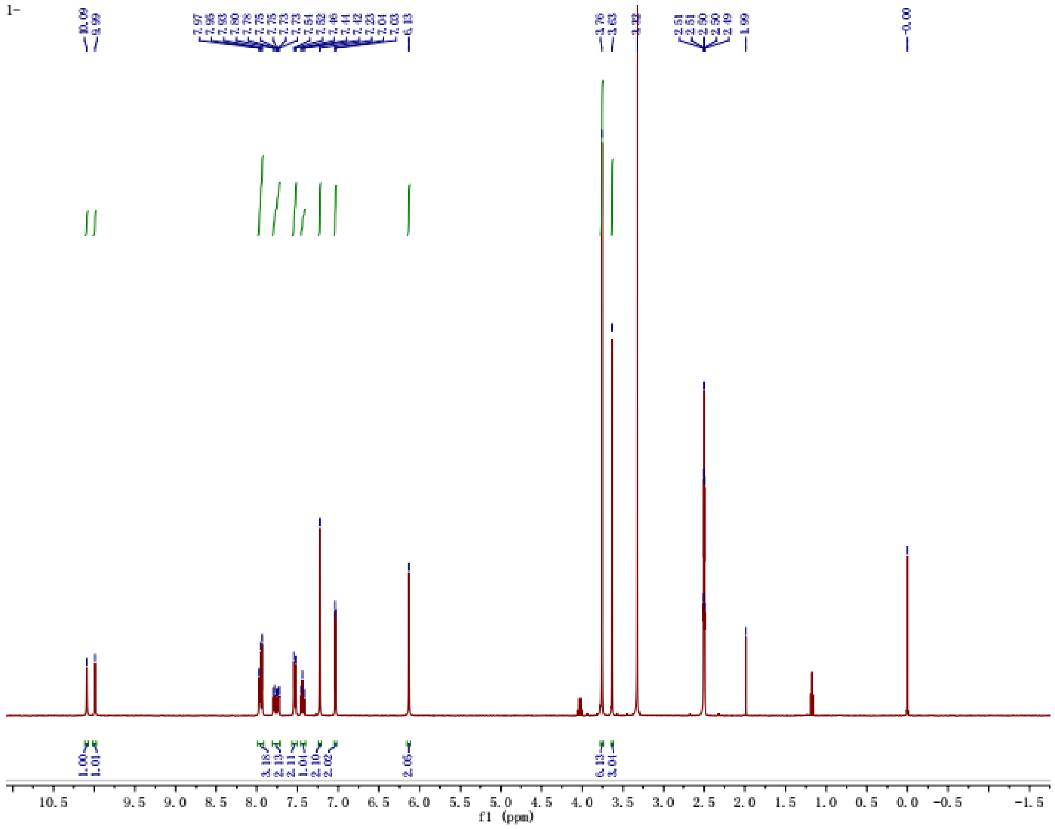

^{1}H-NMR spectrum of **A8**

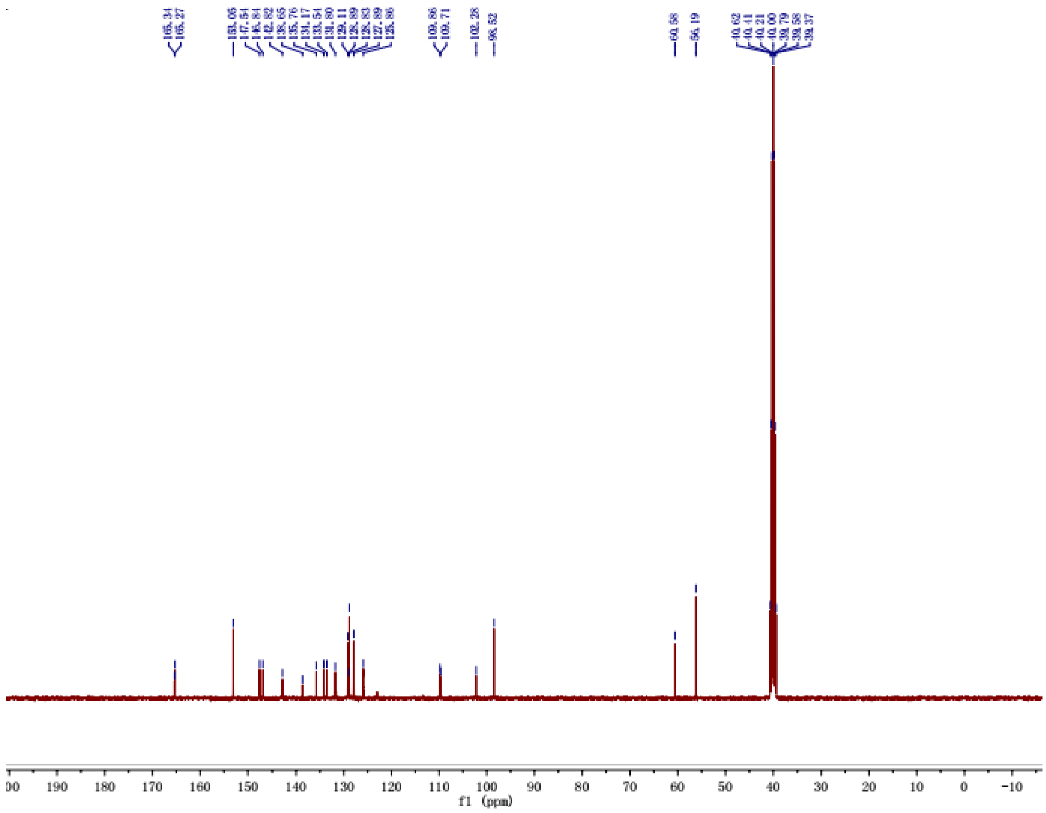

^{13}C-NMR spectrum of **A8**

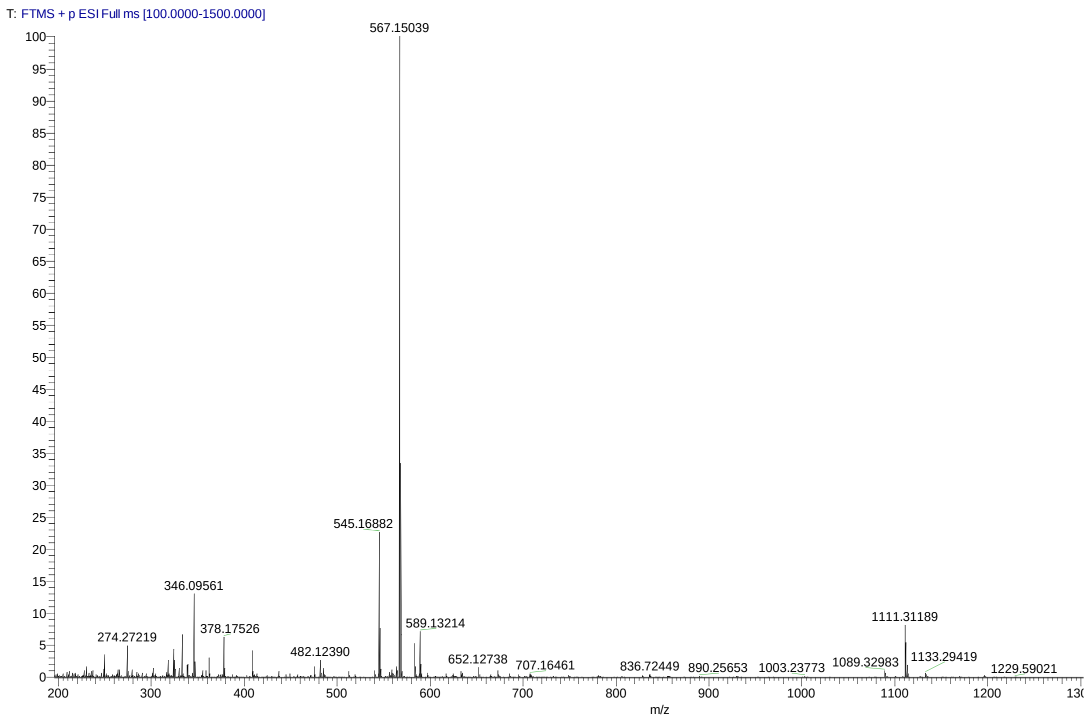

HRMS spectrum of **A9**

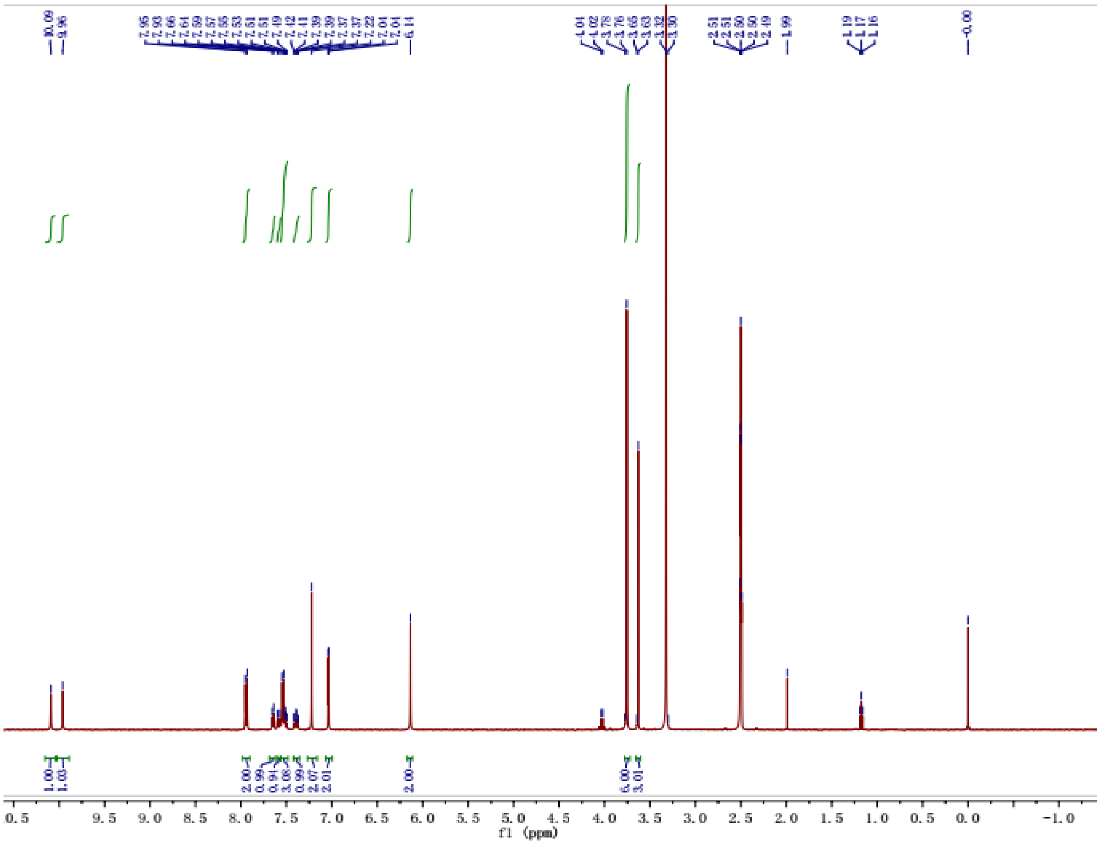

^{1}H-NMR spectrum of **A9**

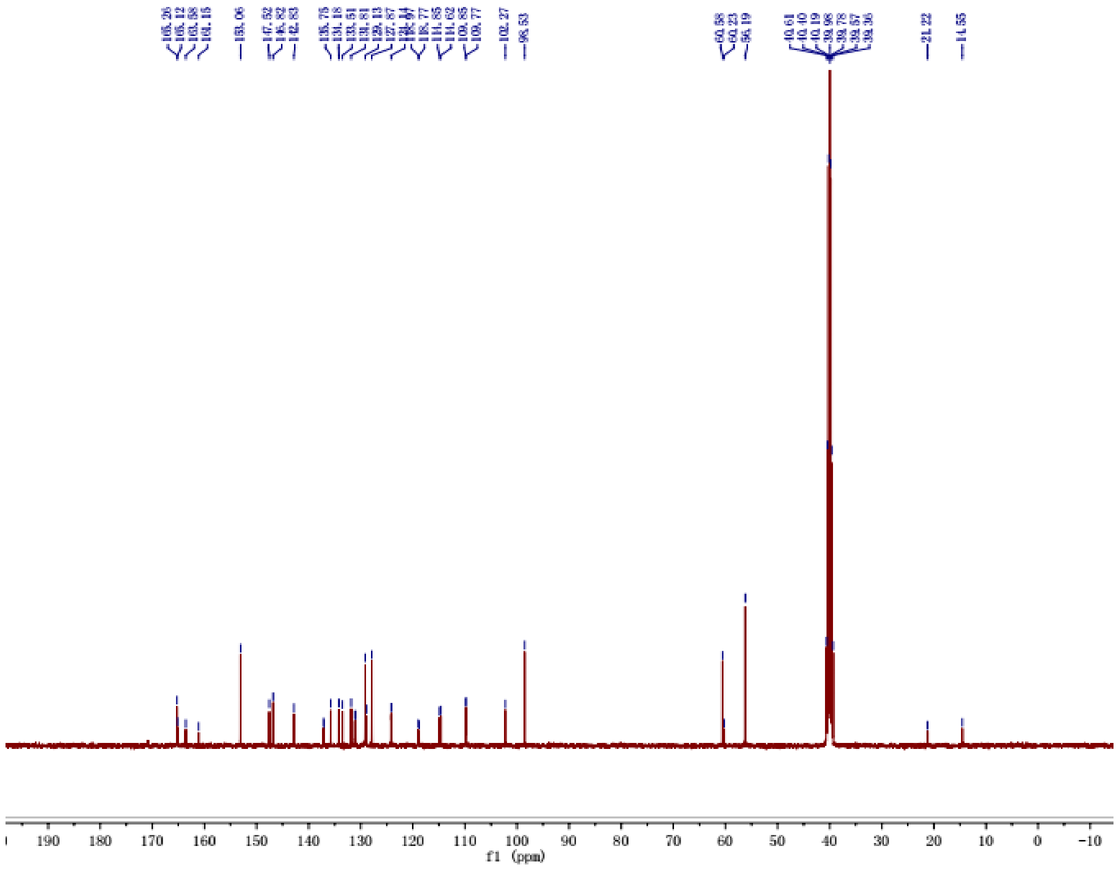

^{13}C-NMR spectrum of **A9**

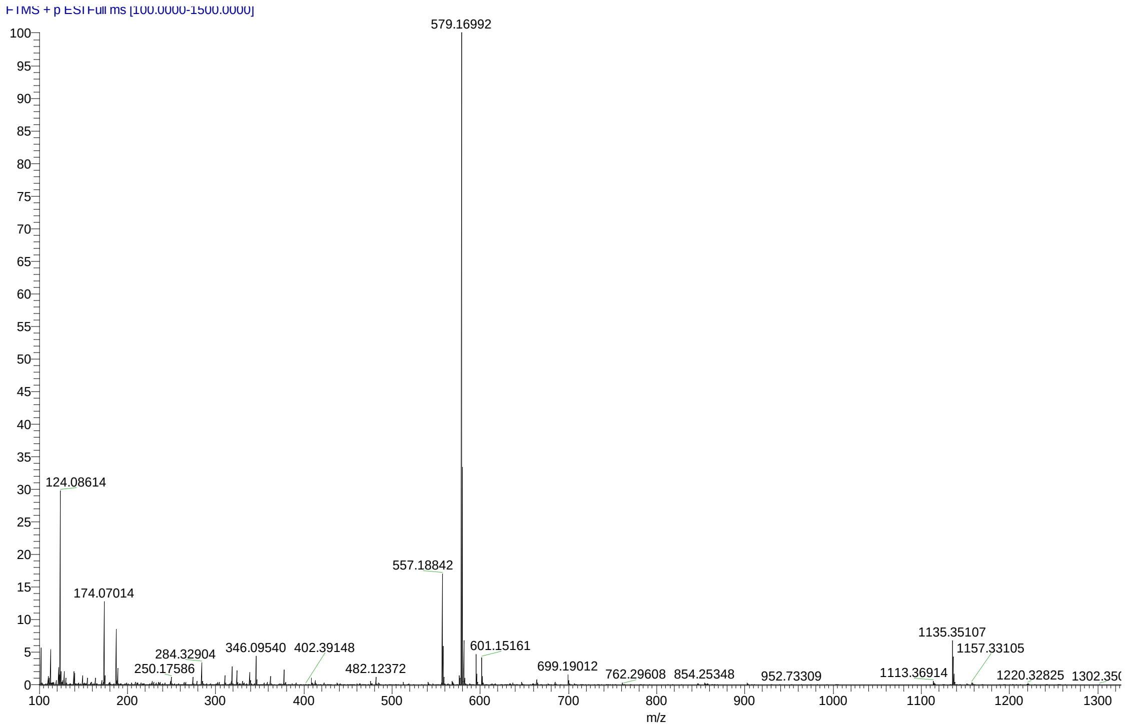

HRMS spectrum of **A10**

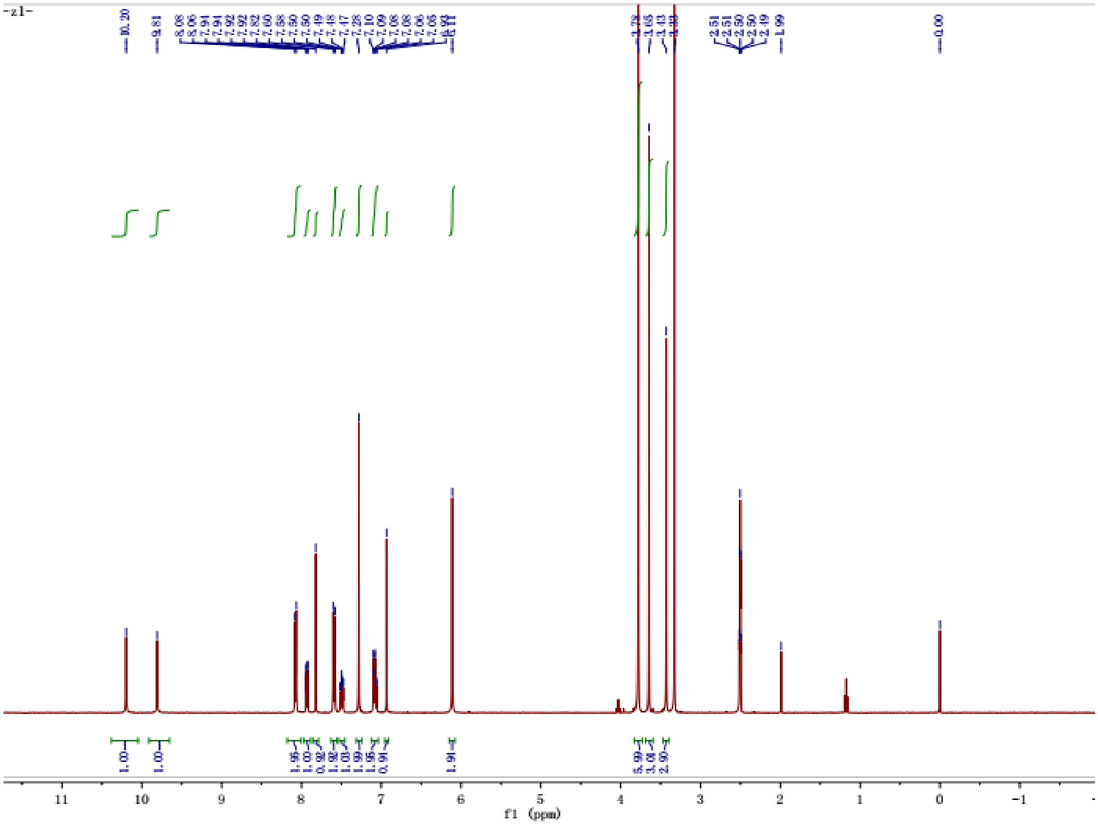

^{1}H-NMR spectrum of **A10**

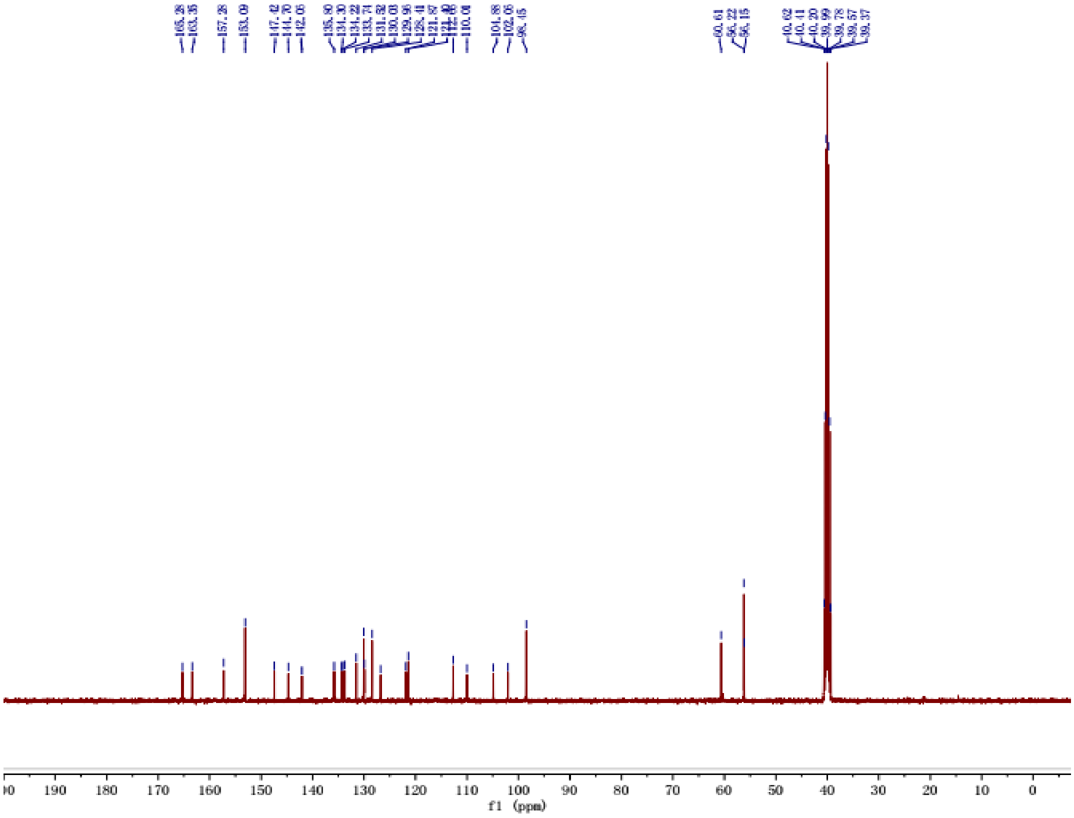

^{13}C-NMR spectrum of **A10**

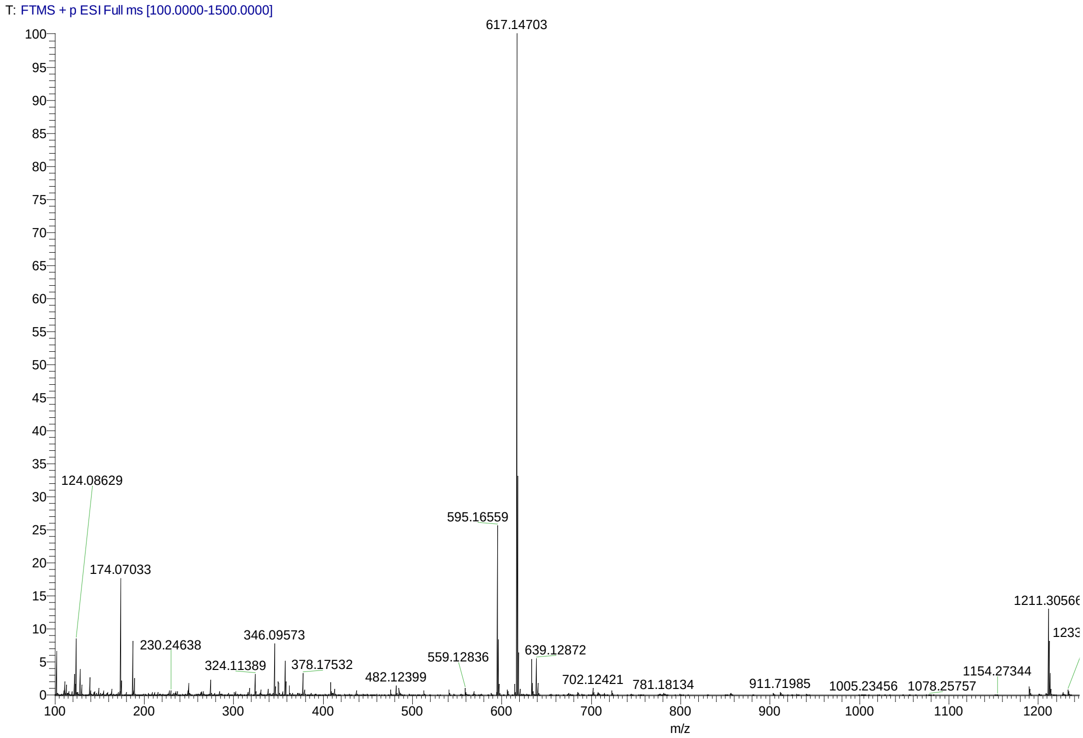

HRMS spectrum of **A11**

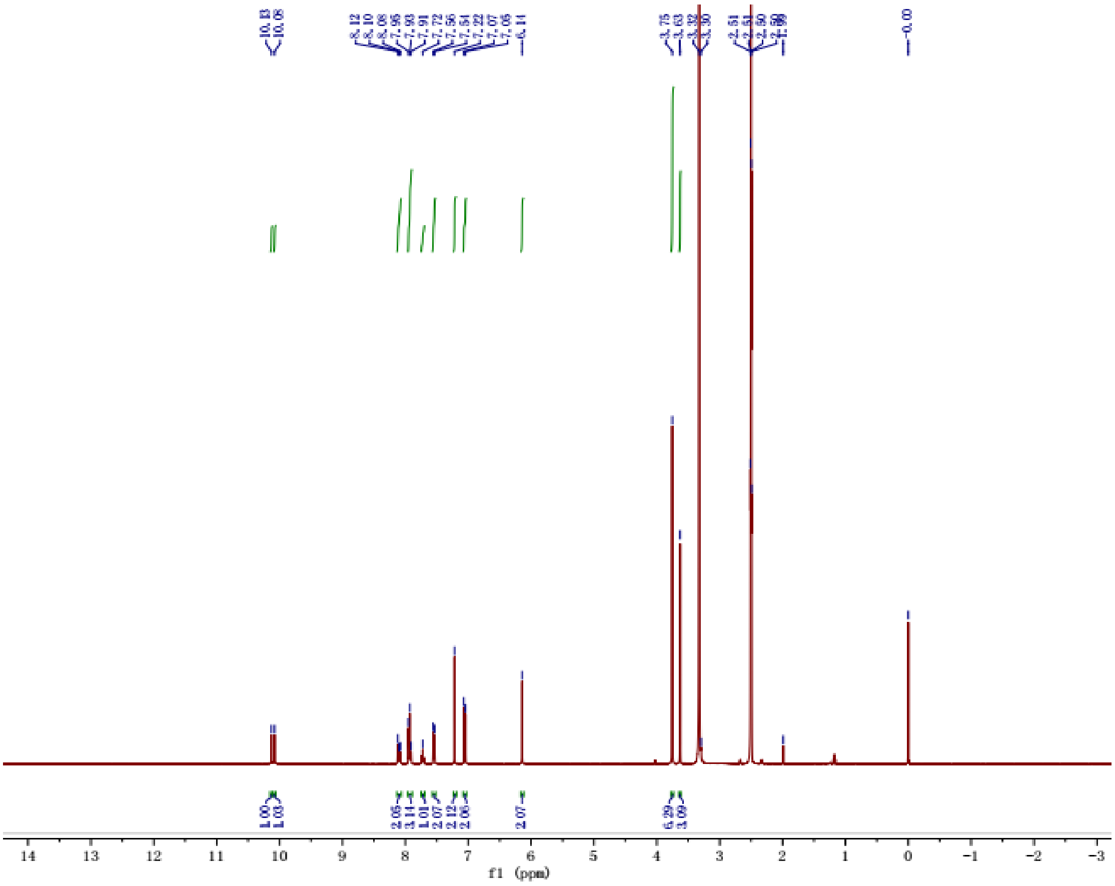

^{1}H-NMR spectrum of **A11**

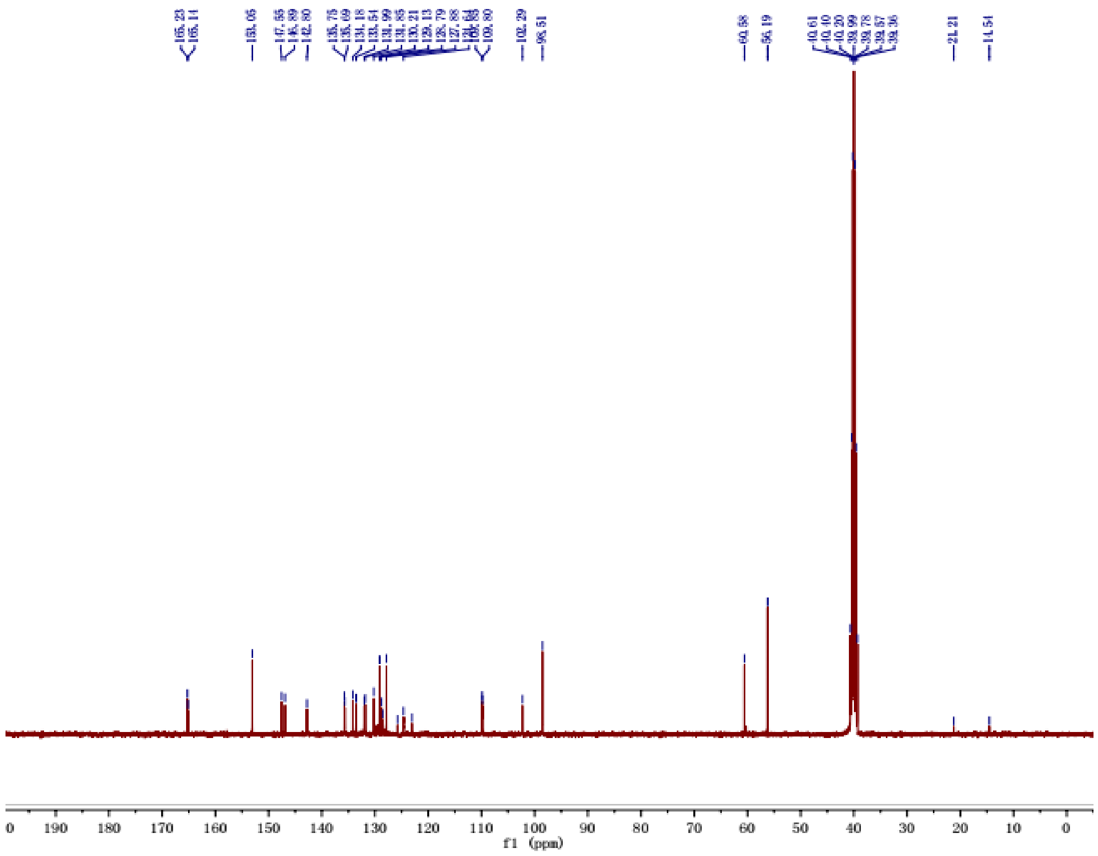

^{13}C-NMR spectrum of **A11**

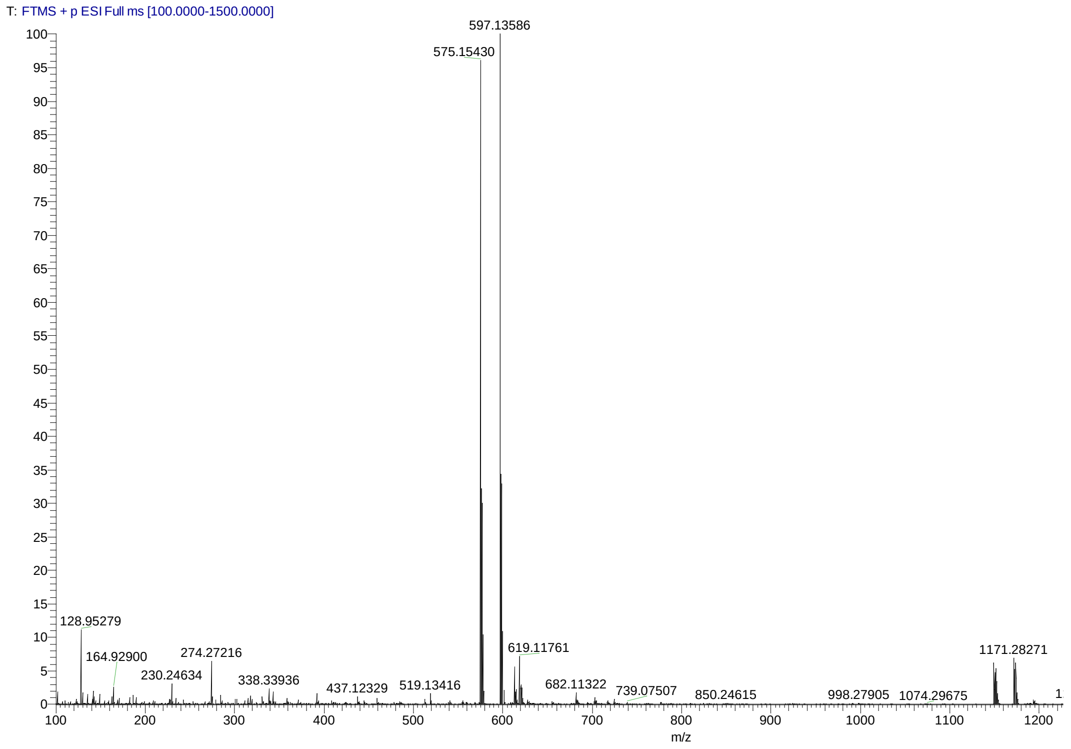

HRMS spectrum of **A12**

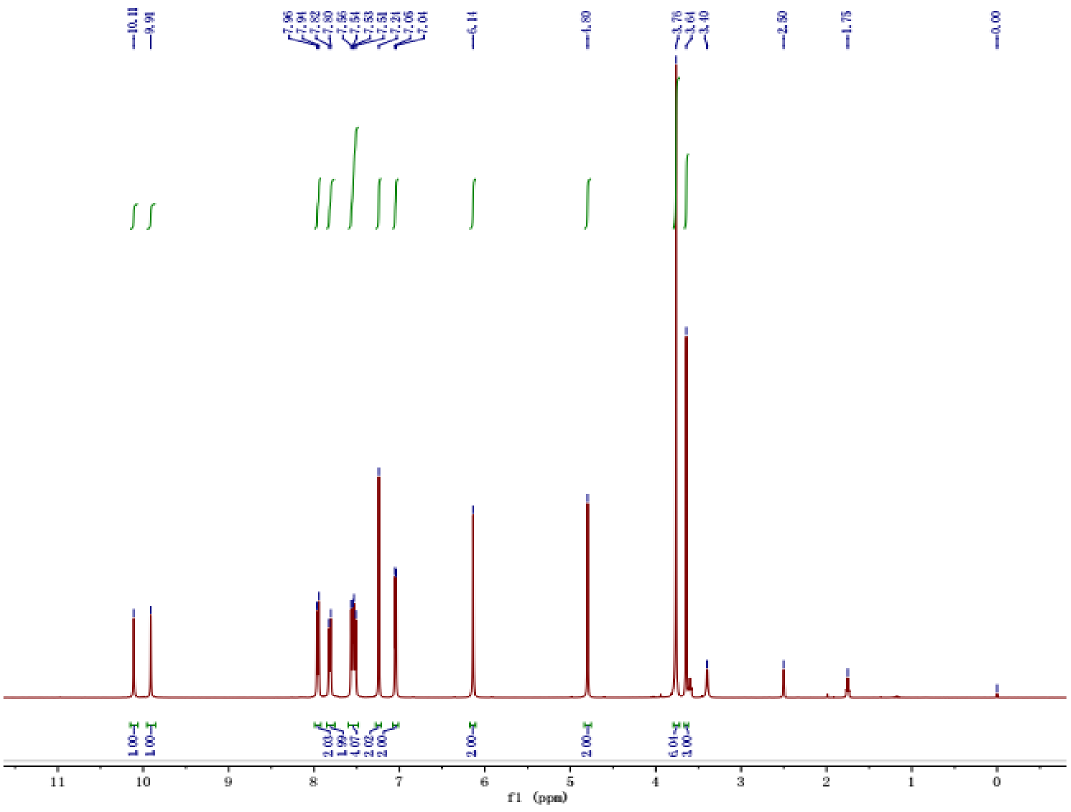

^{1}H-NMR spectrum of **A12**

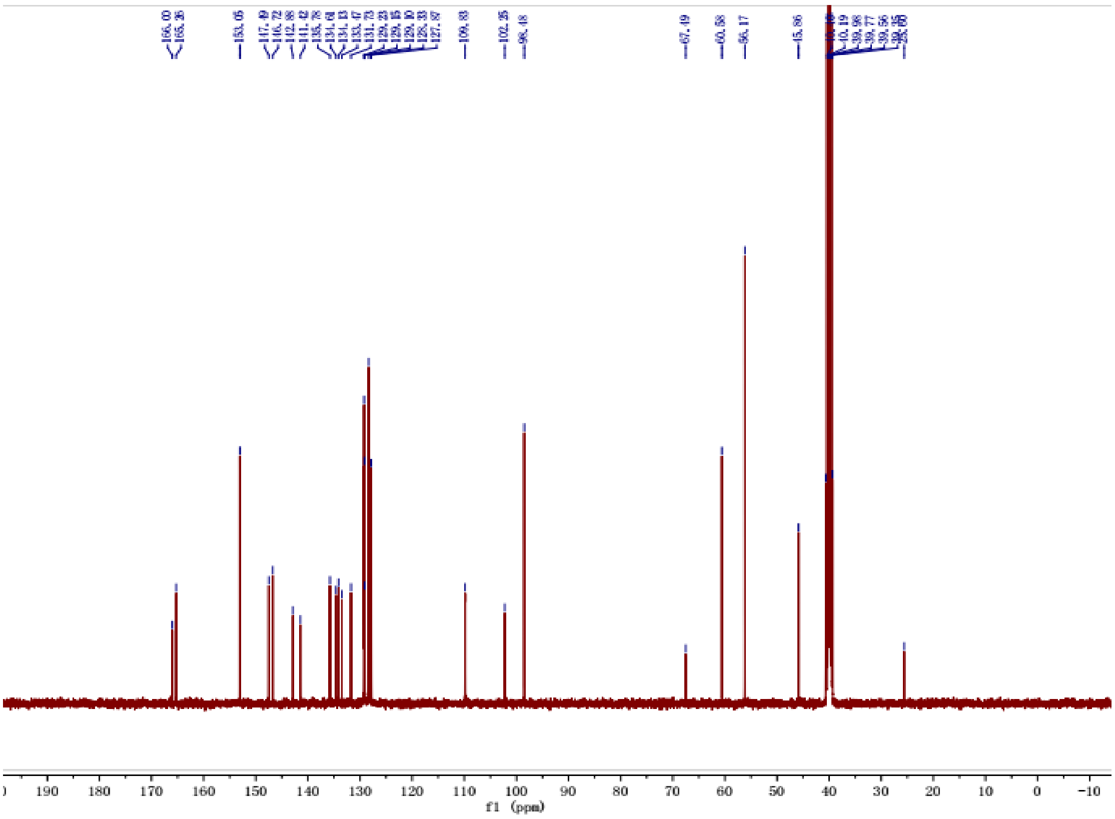

^{13}C-NMR spectrum of **A12**

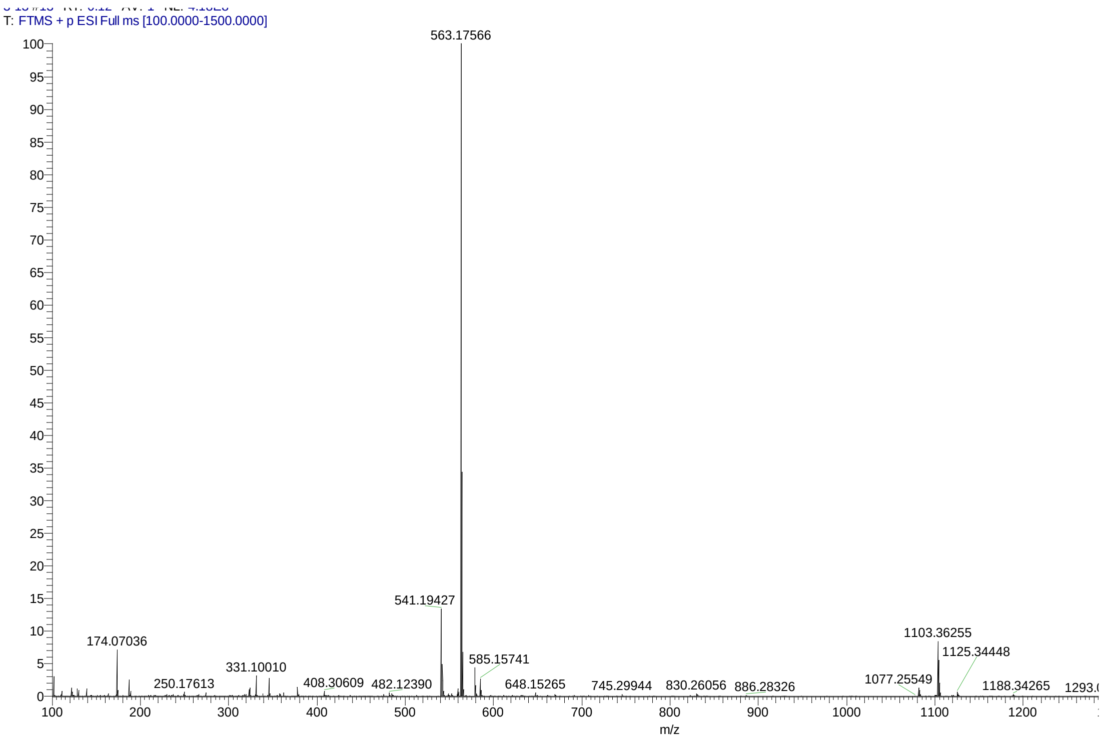

HRMS spectrum of **A13**

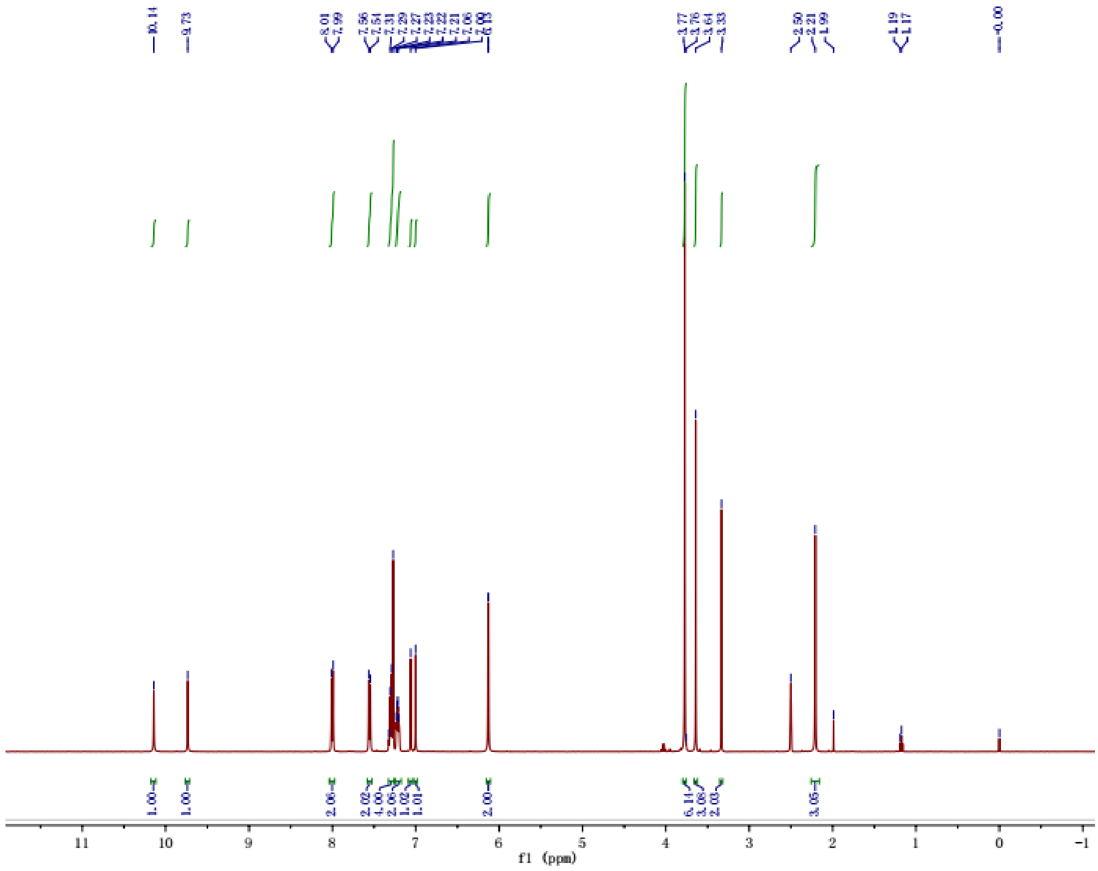

¹H-NMR spectrum of **A13**

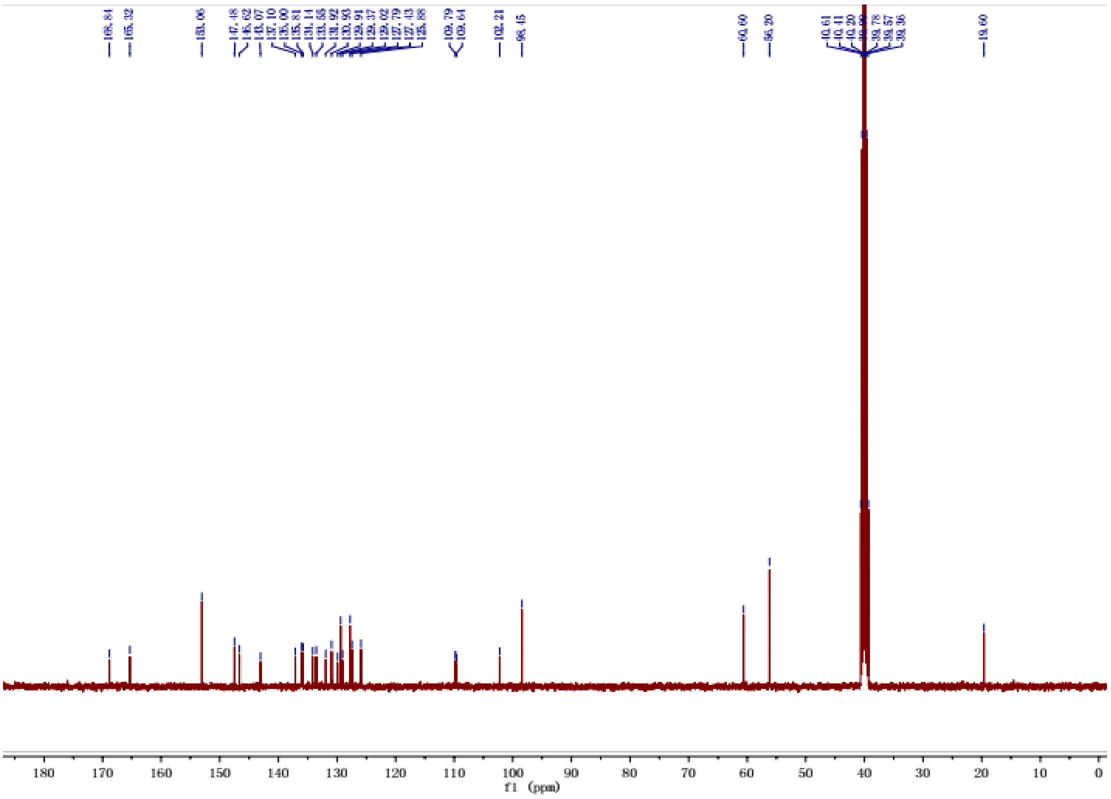

^{13}C-NMR spectrum of **A13**

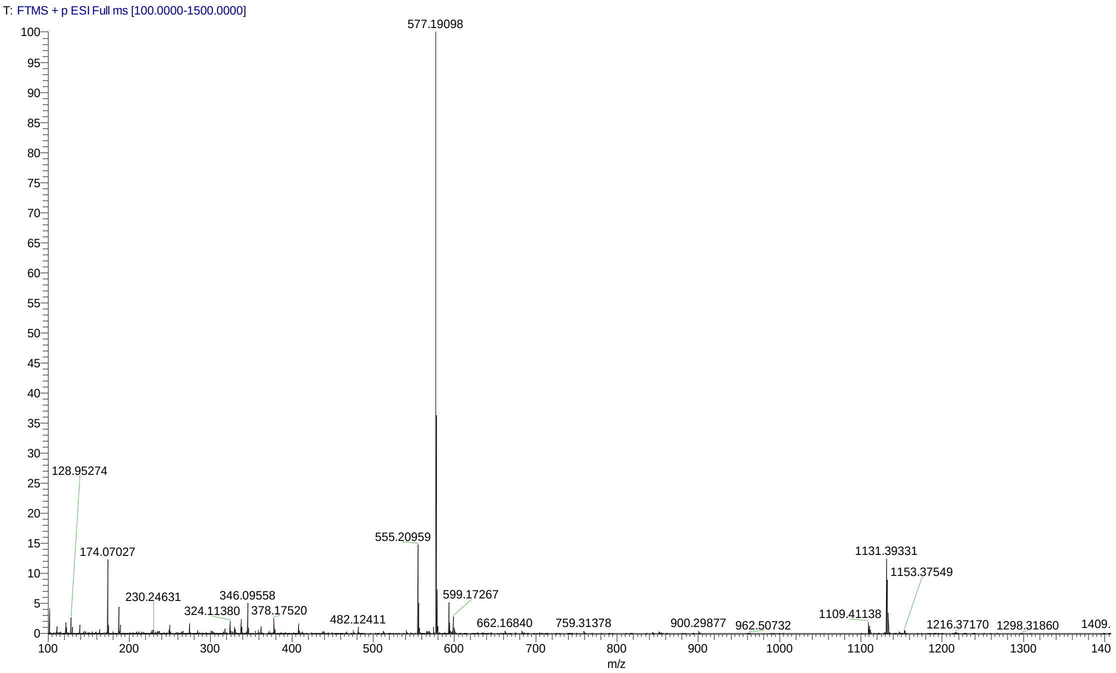

HRMS spectrum of **A14**

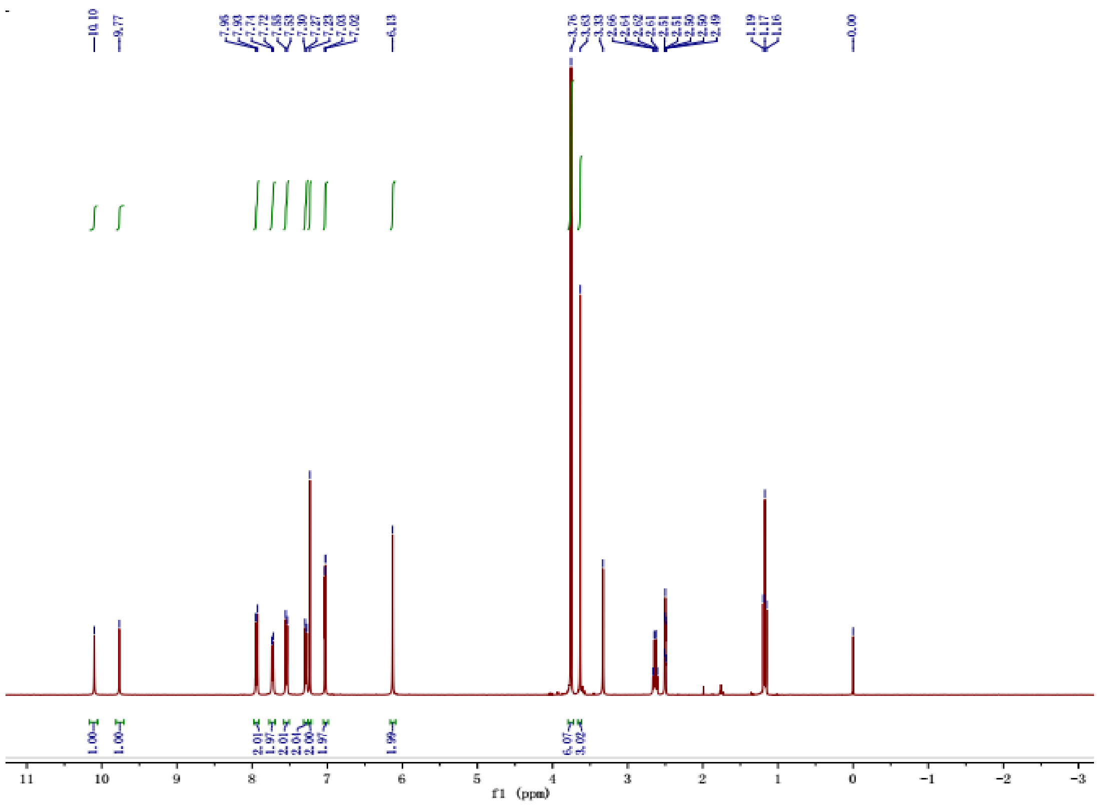

^{1}H-NMR spectrum of **A14**

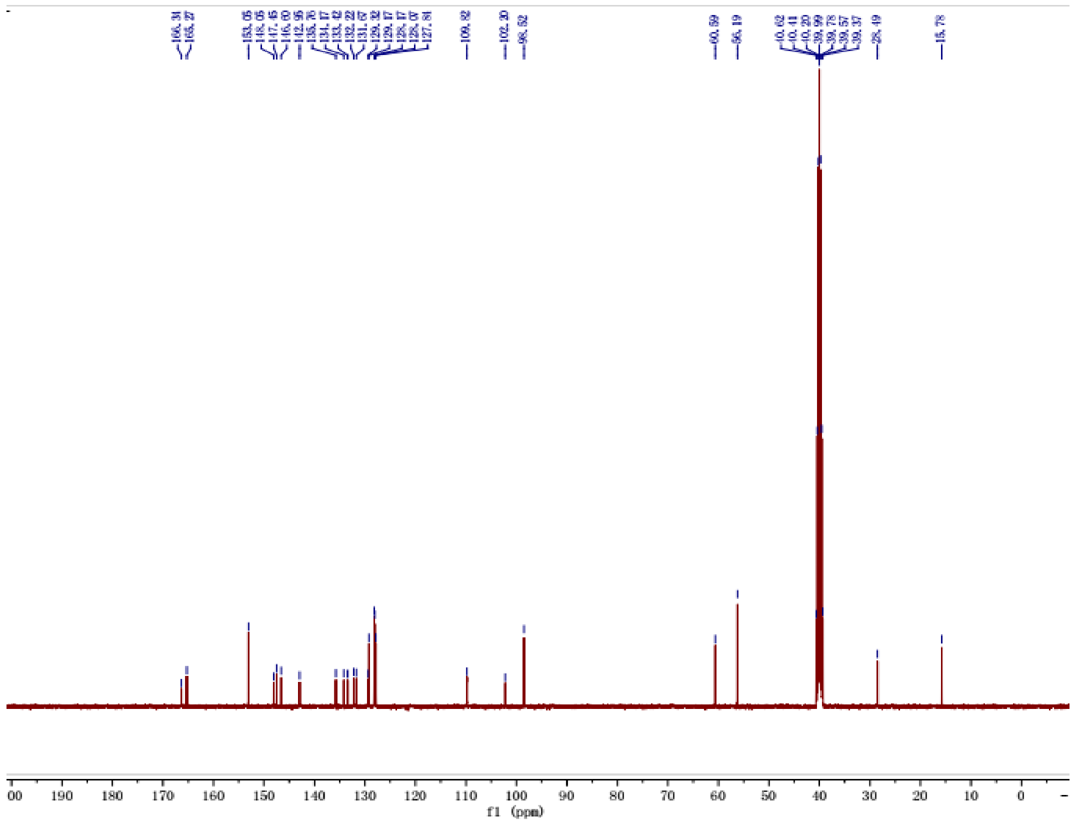

^{13}C-NMR spectrum of **A14**

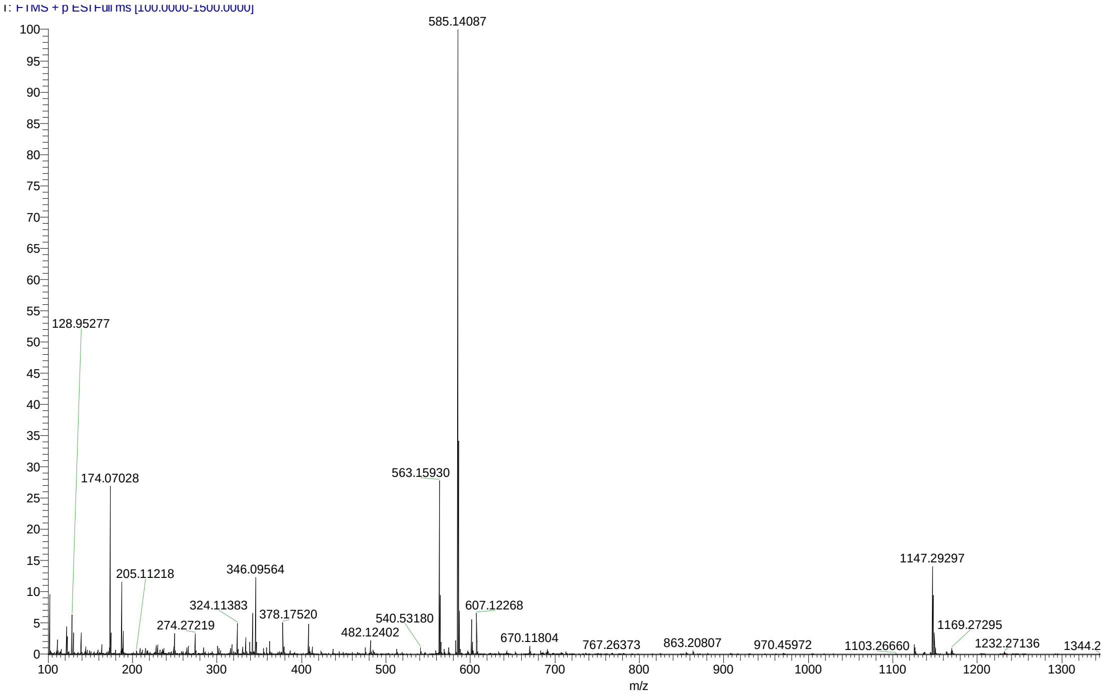

HRMS spectrum of **A15**

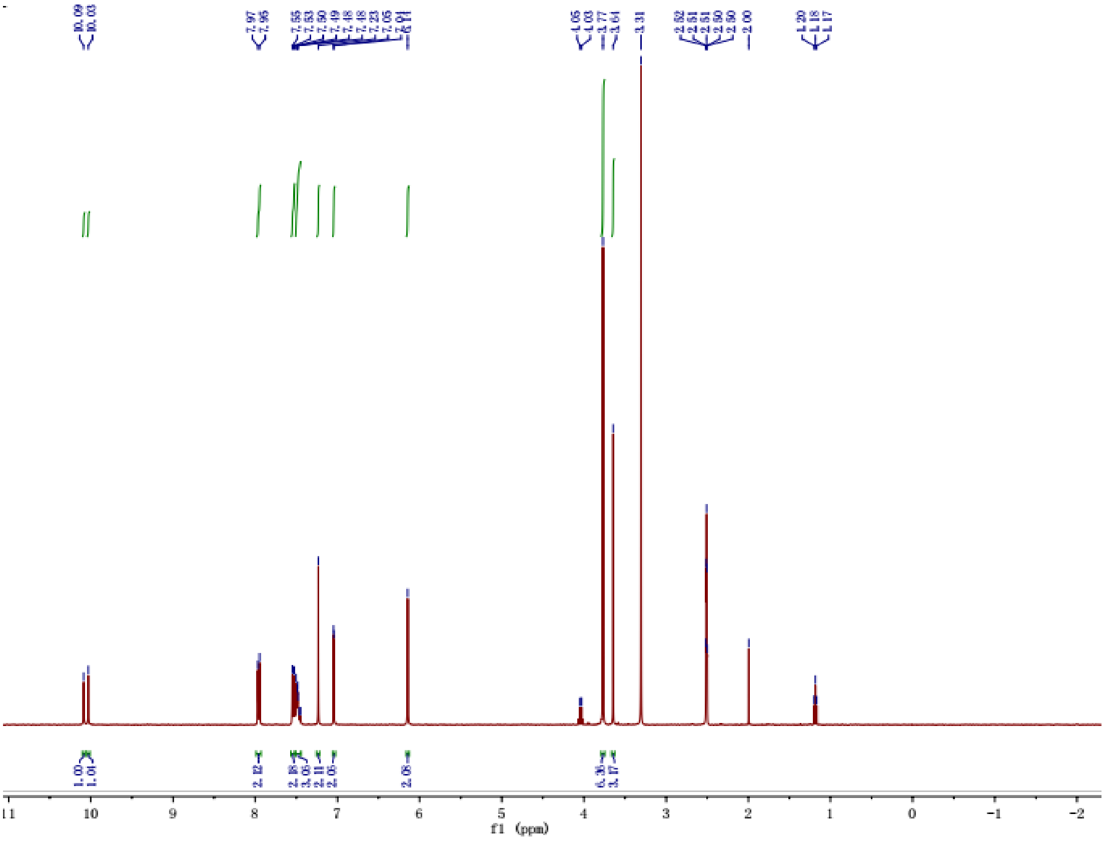

^{1}H-NMR spectrum of **A16**

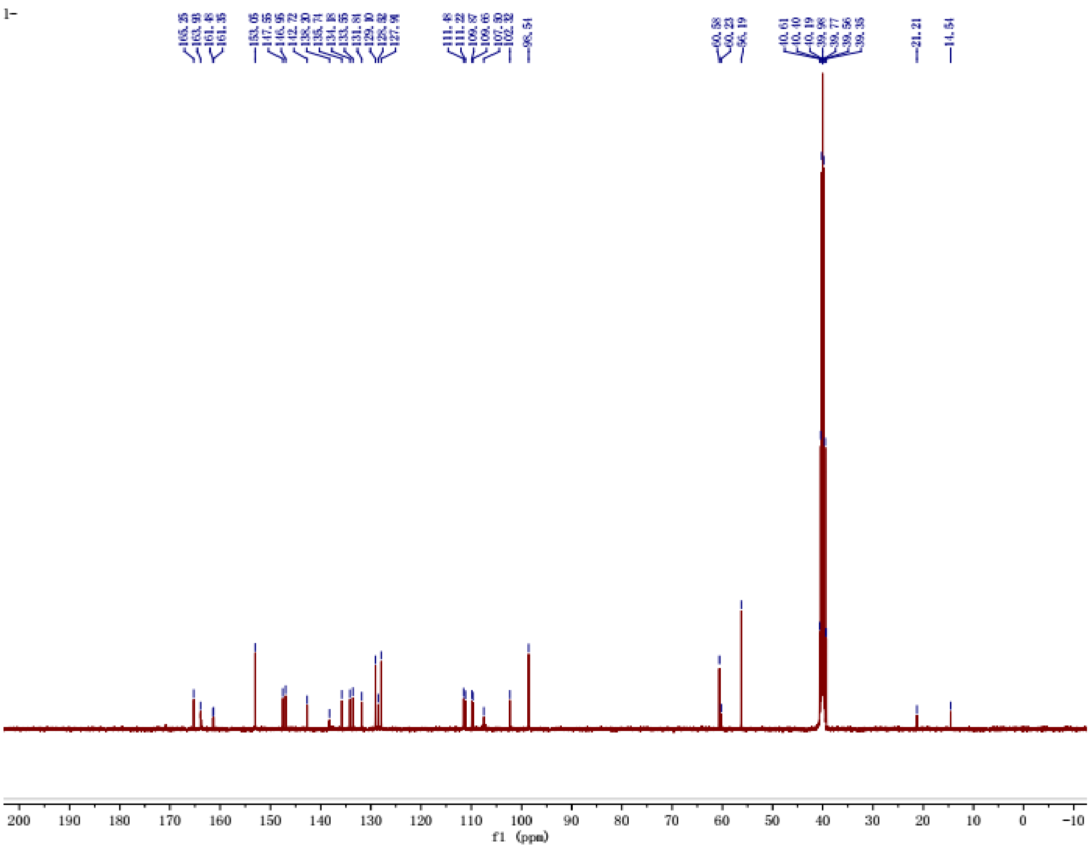

^{13}C-NMR spectrum of **A15**

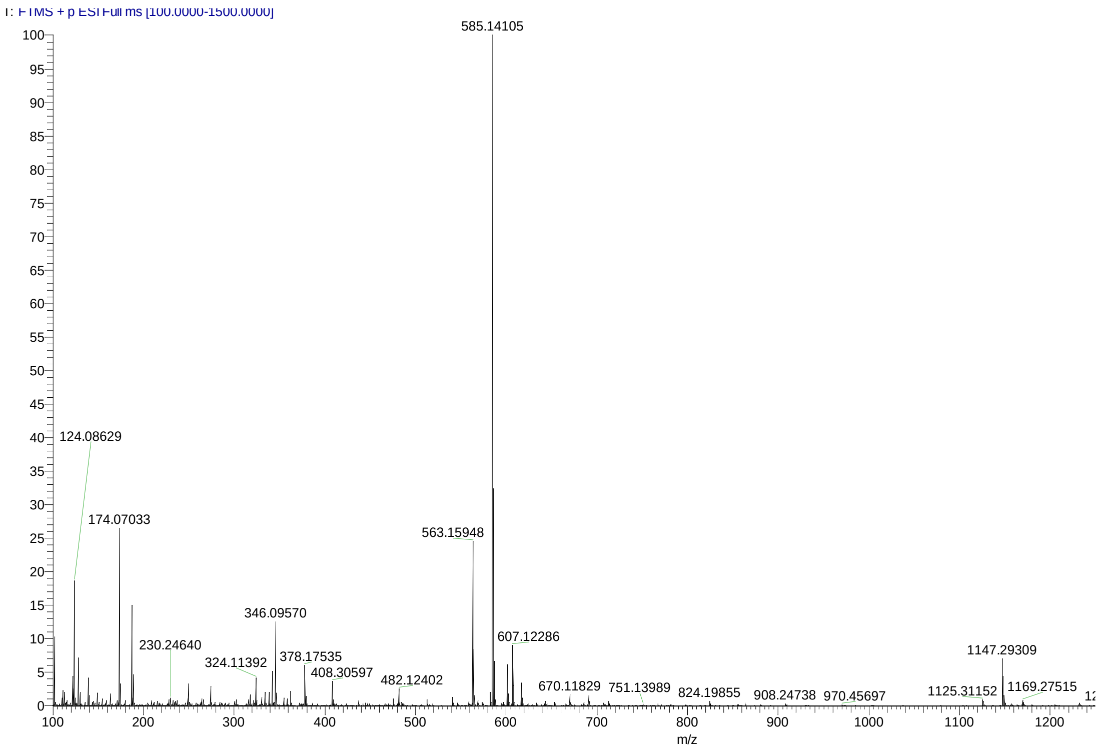

HRMS spectrum of **A16**

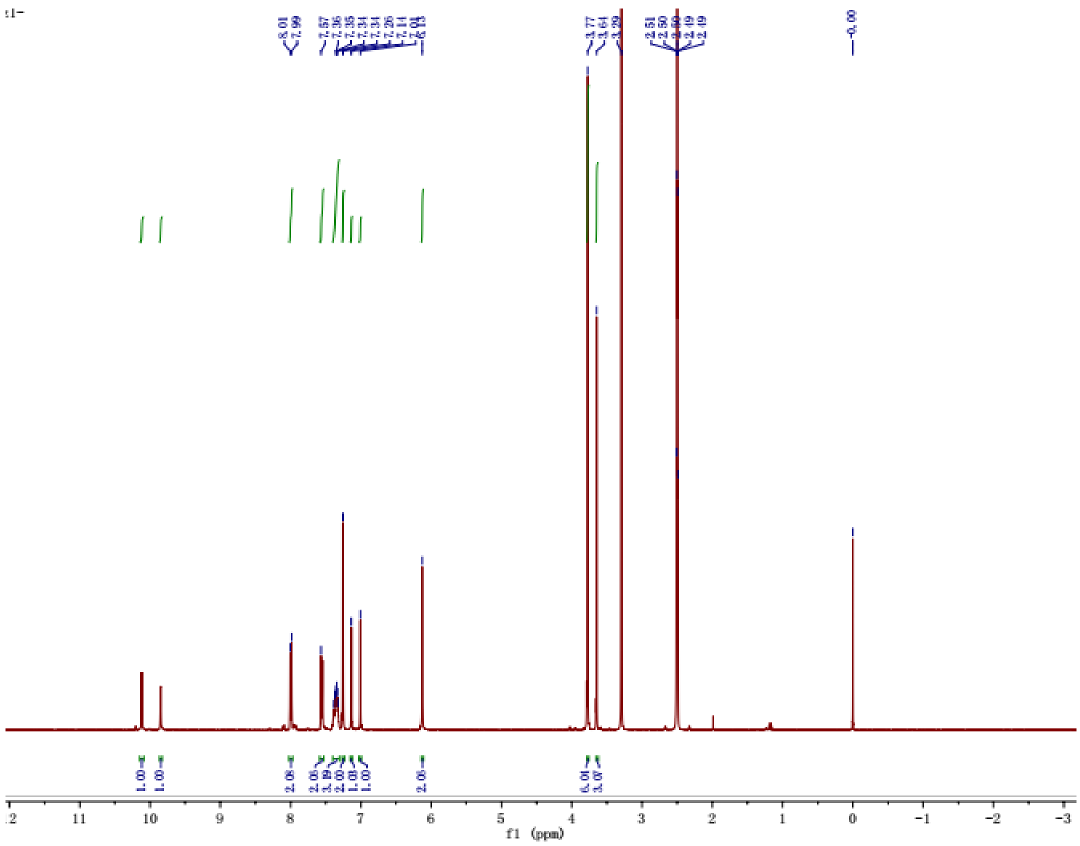

^{1}H-NMR spectrum of **A16**

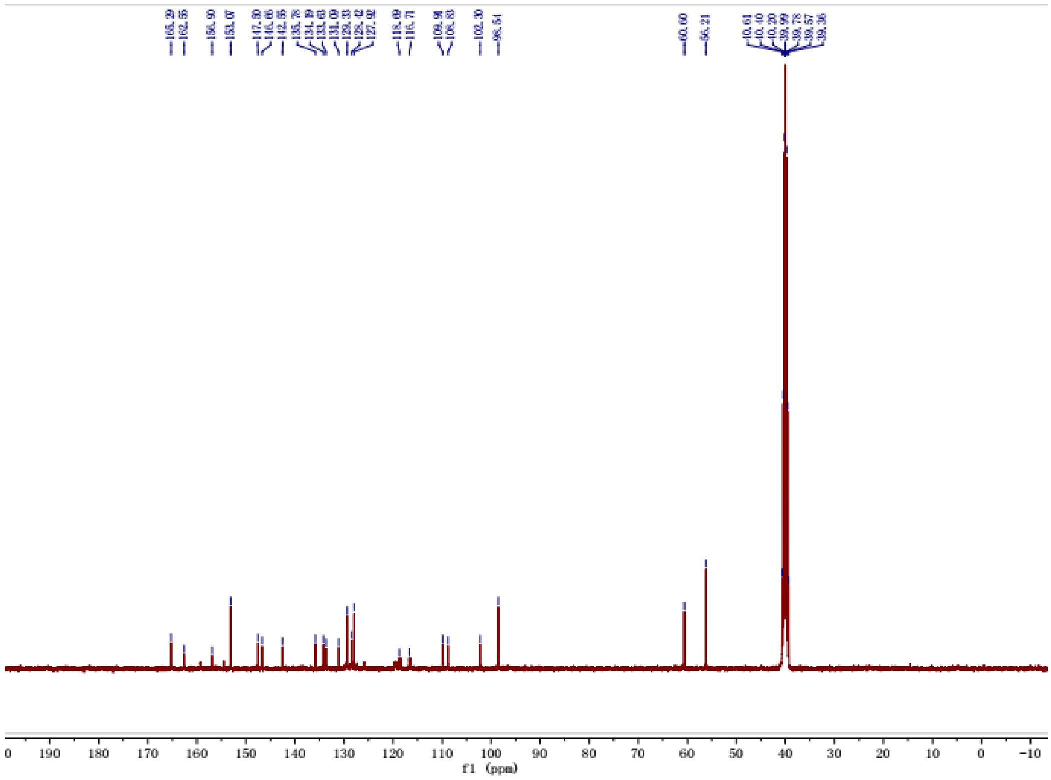

^{13}C-NMR spectrum of **A16**

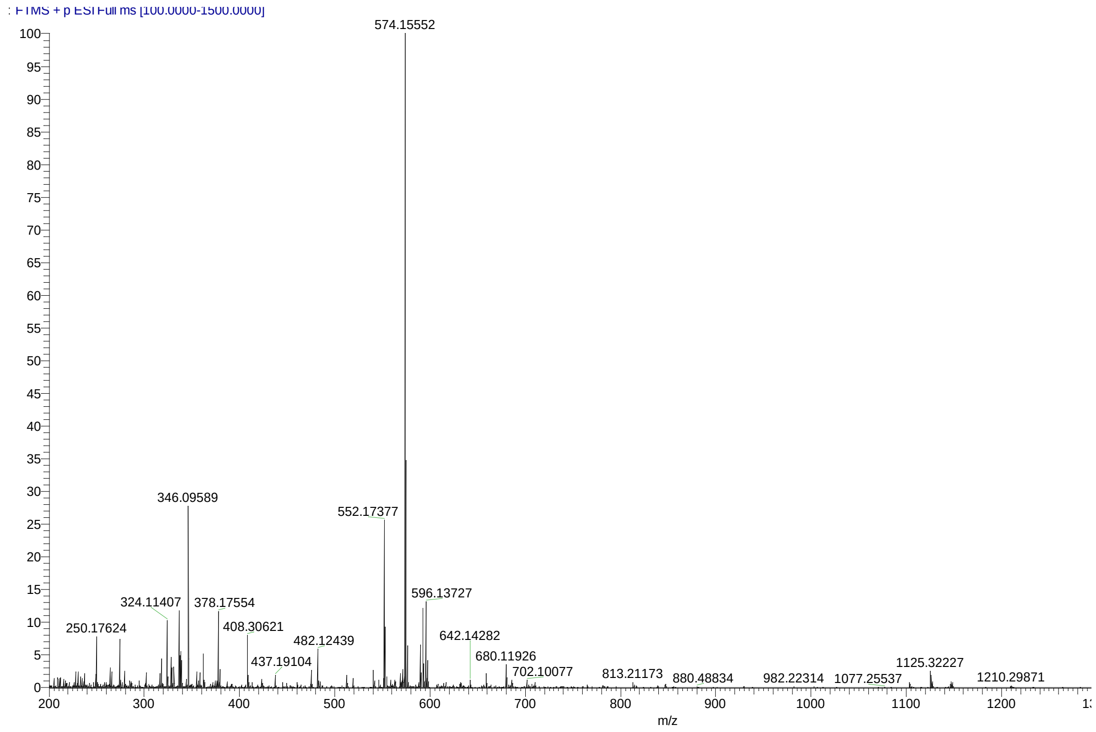

HRMS spectrum of **A17**

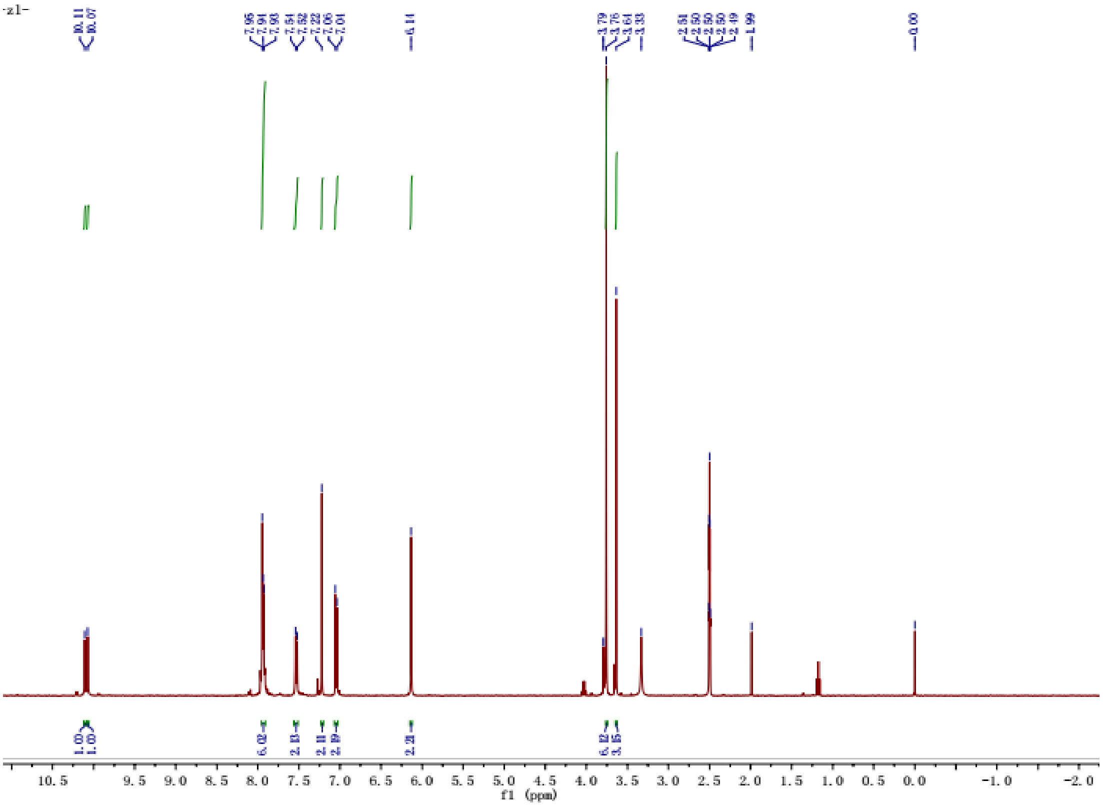

^{1}H-NMR spectrum of **A17**

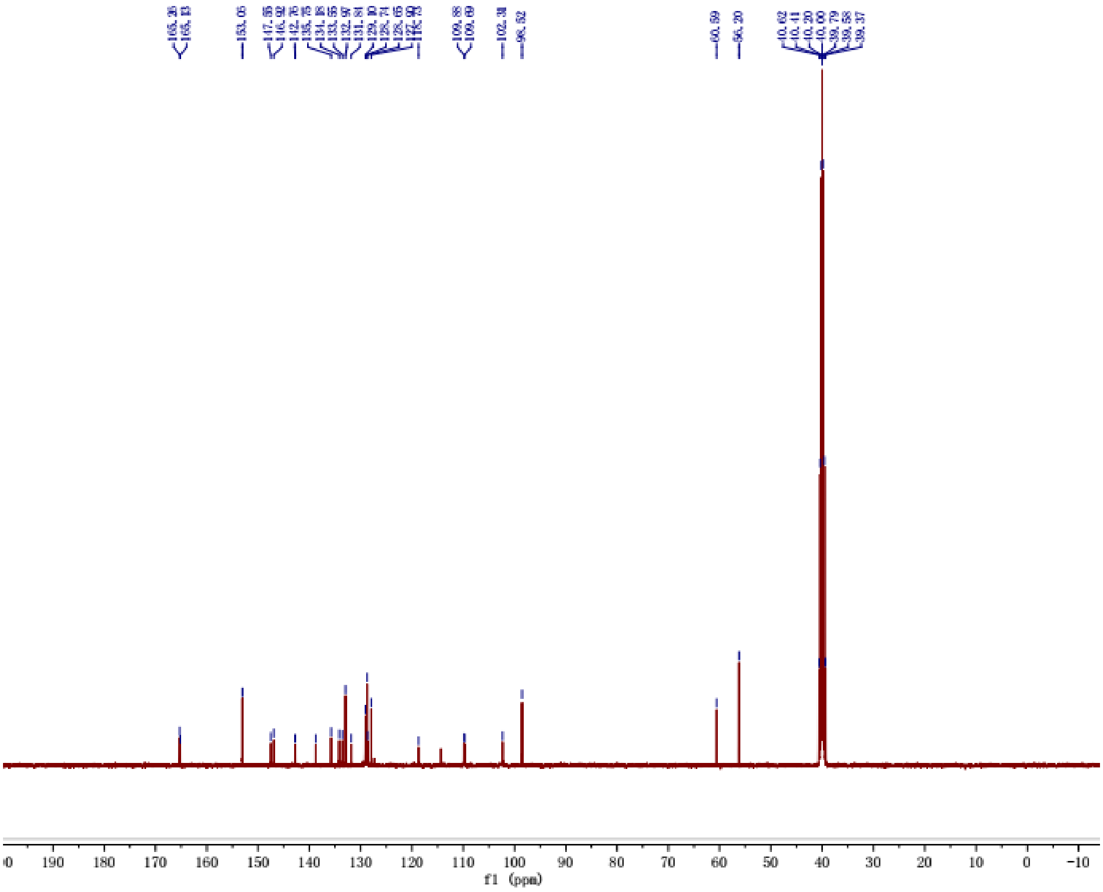

^{13}C-NMR spectrum of **A17**

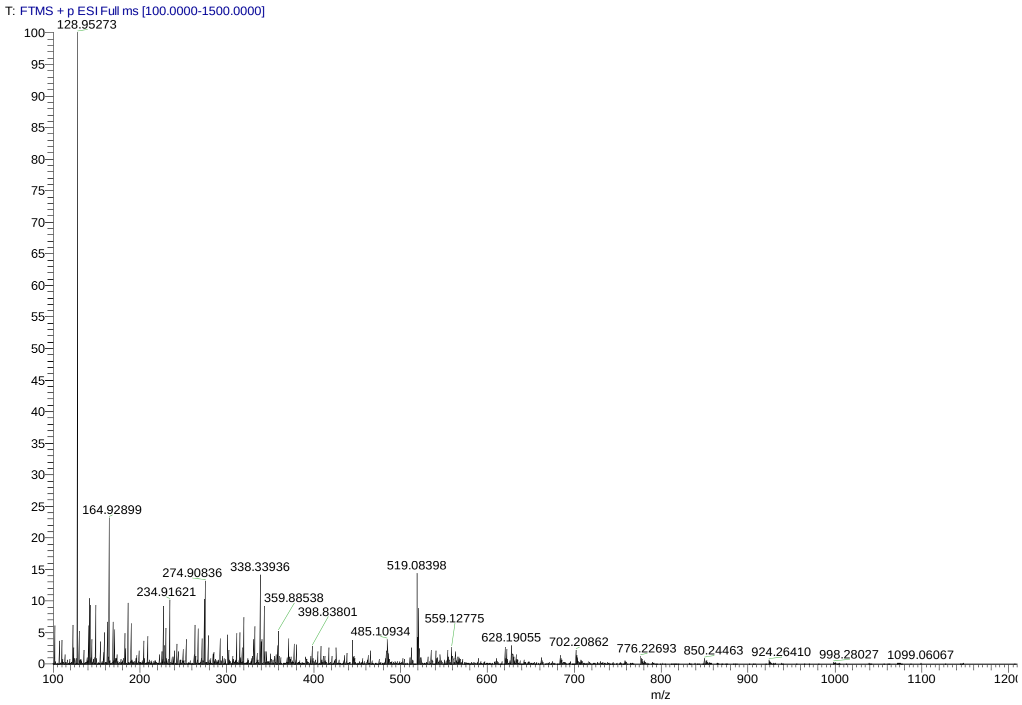

HRMS spectrum of **B1**

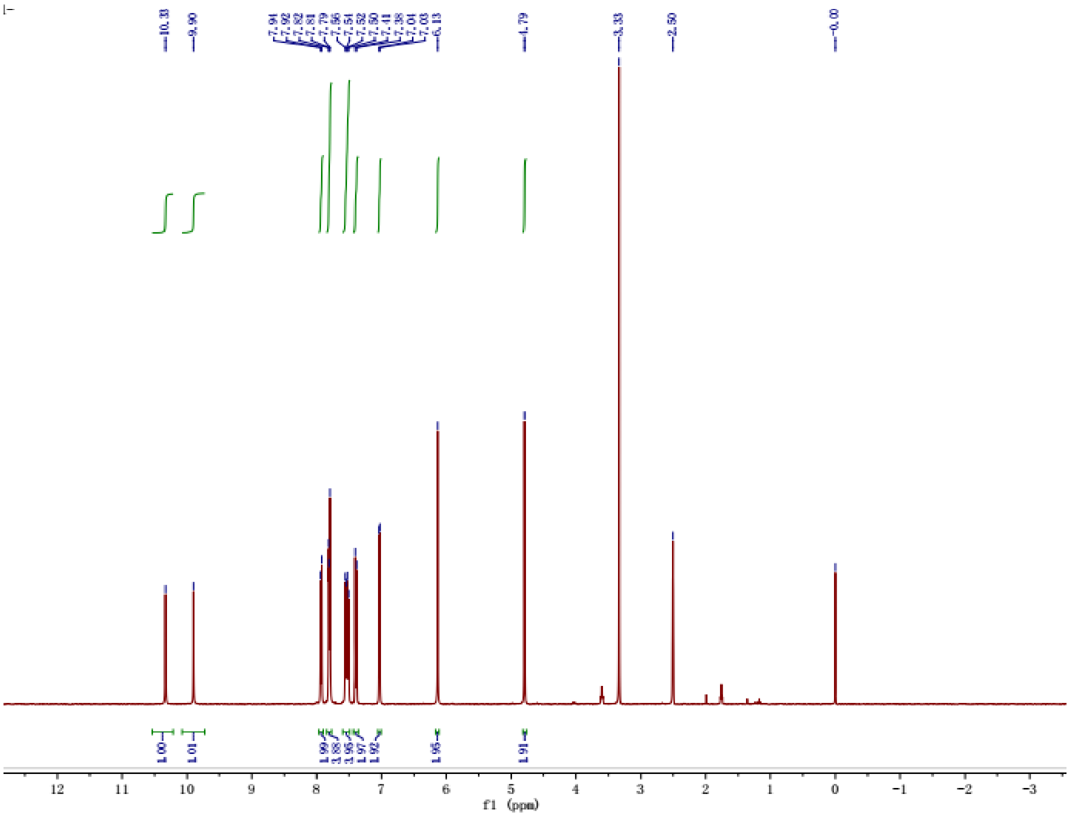

^{1}H-NMR spectrum of **B1**

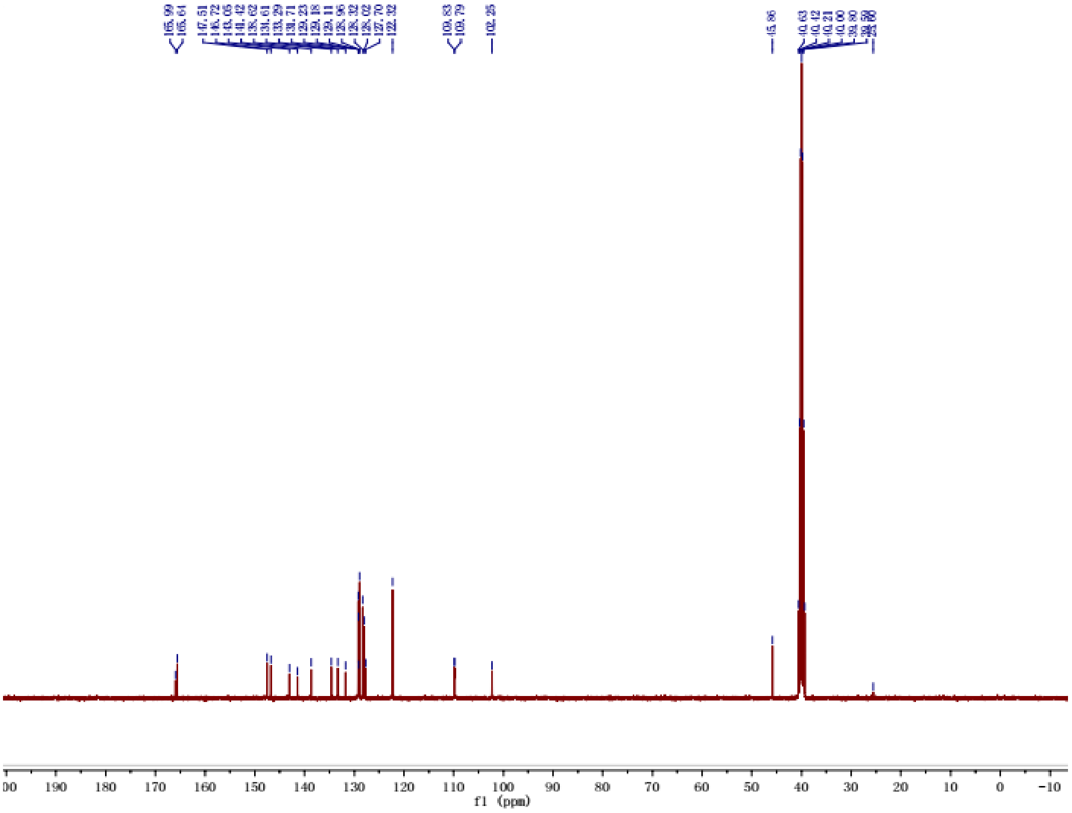

^{13}C-NMR spectrum of **B1**

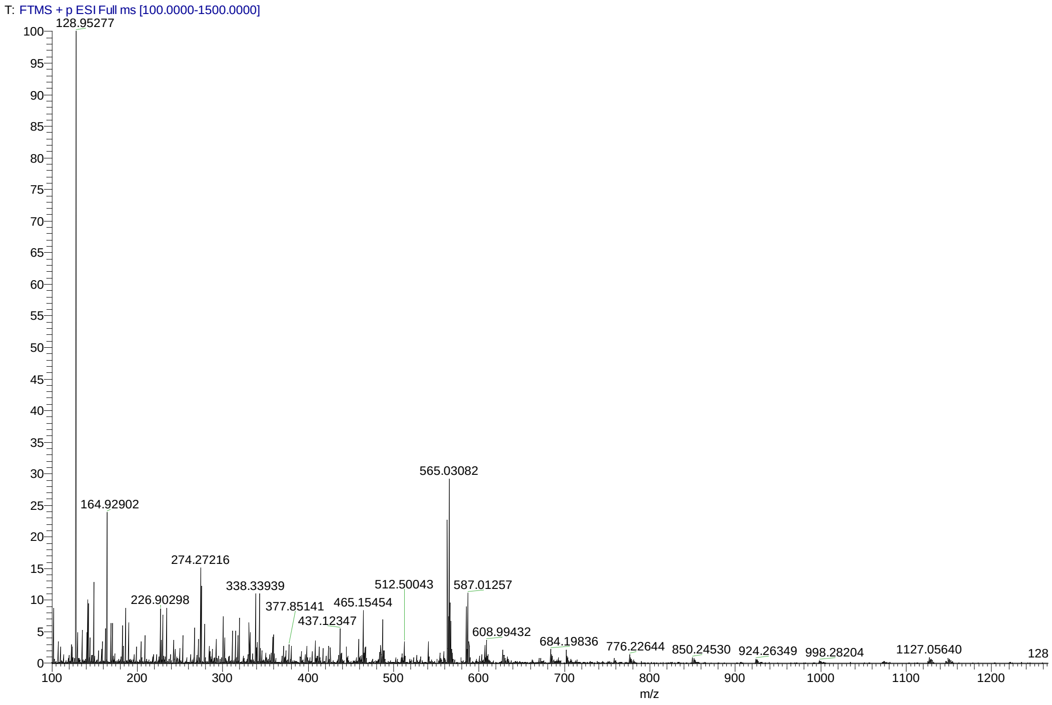

HRMS spectrum of **B2**

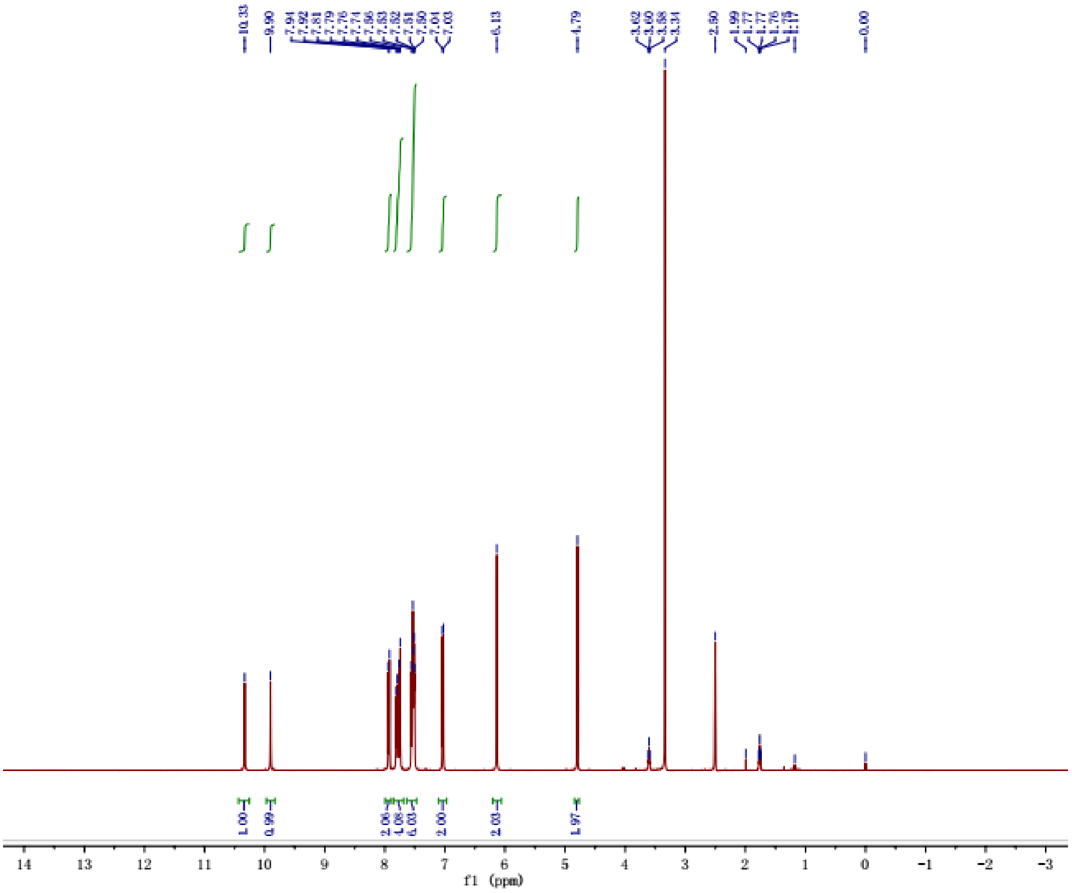

^{1}H-NMR spectrum of **B2**

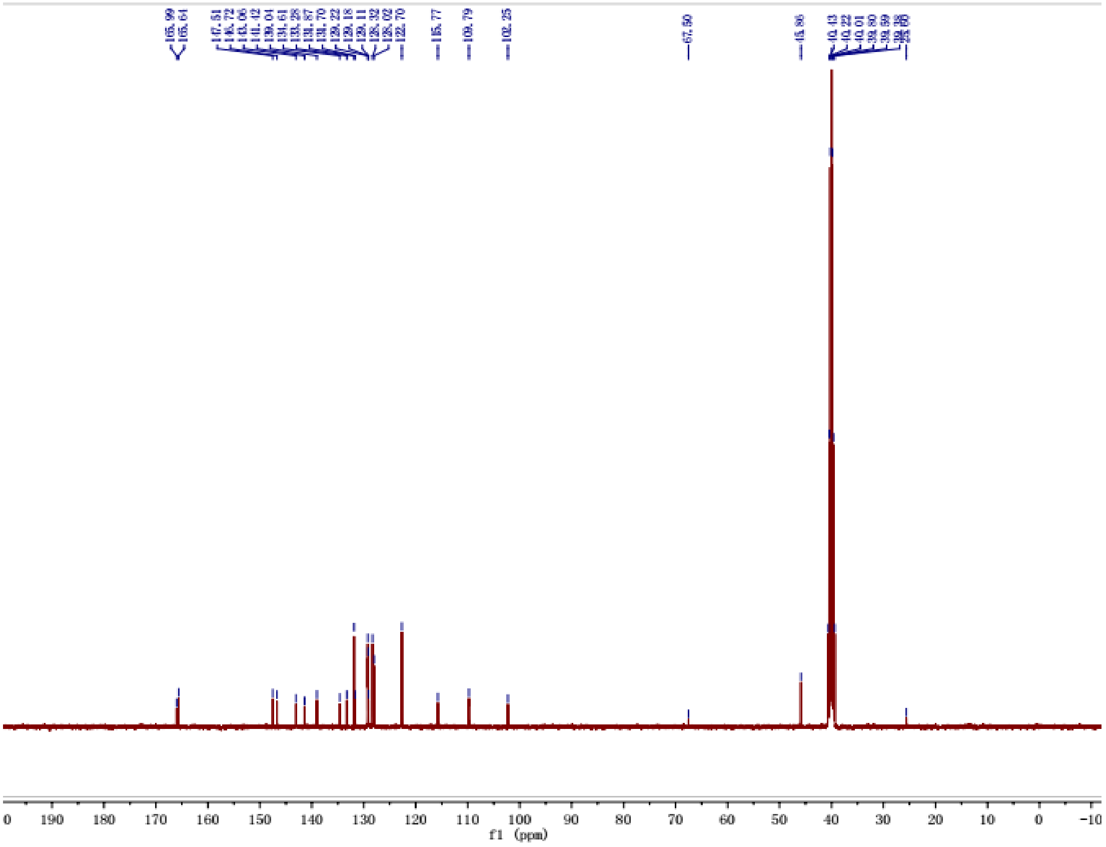

^{13}C-NMR spectrum of **B2**

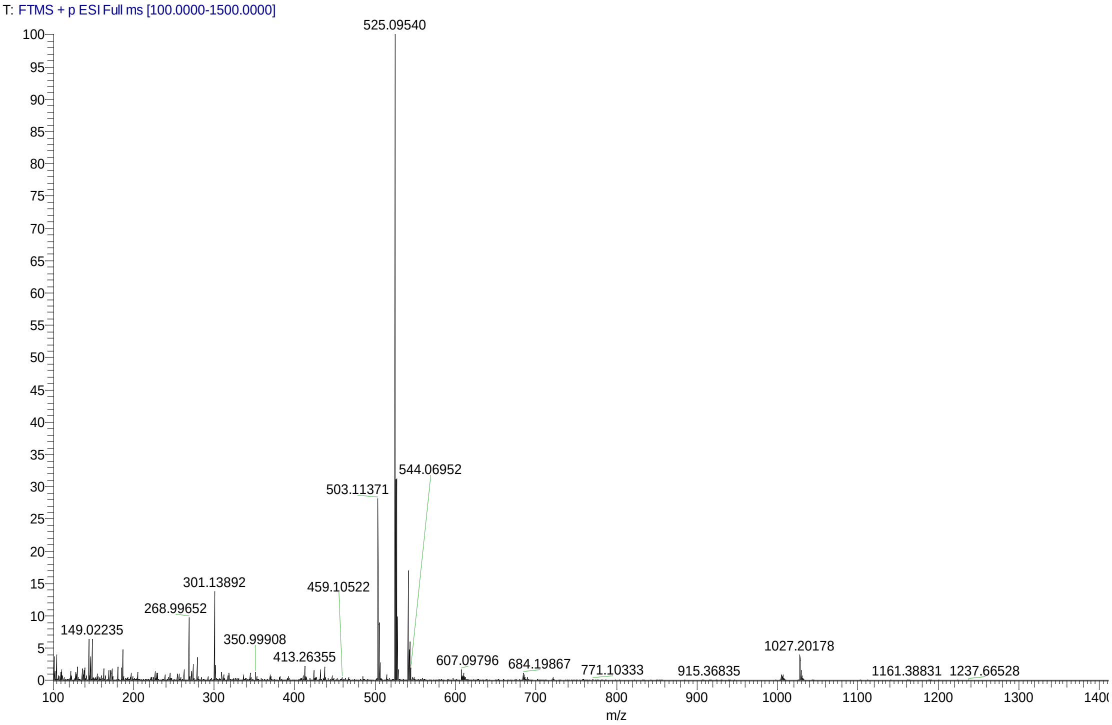

HRMS spectrum of **B3**

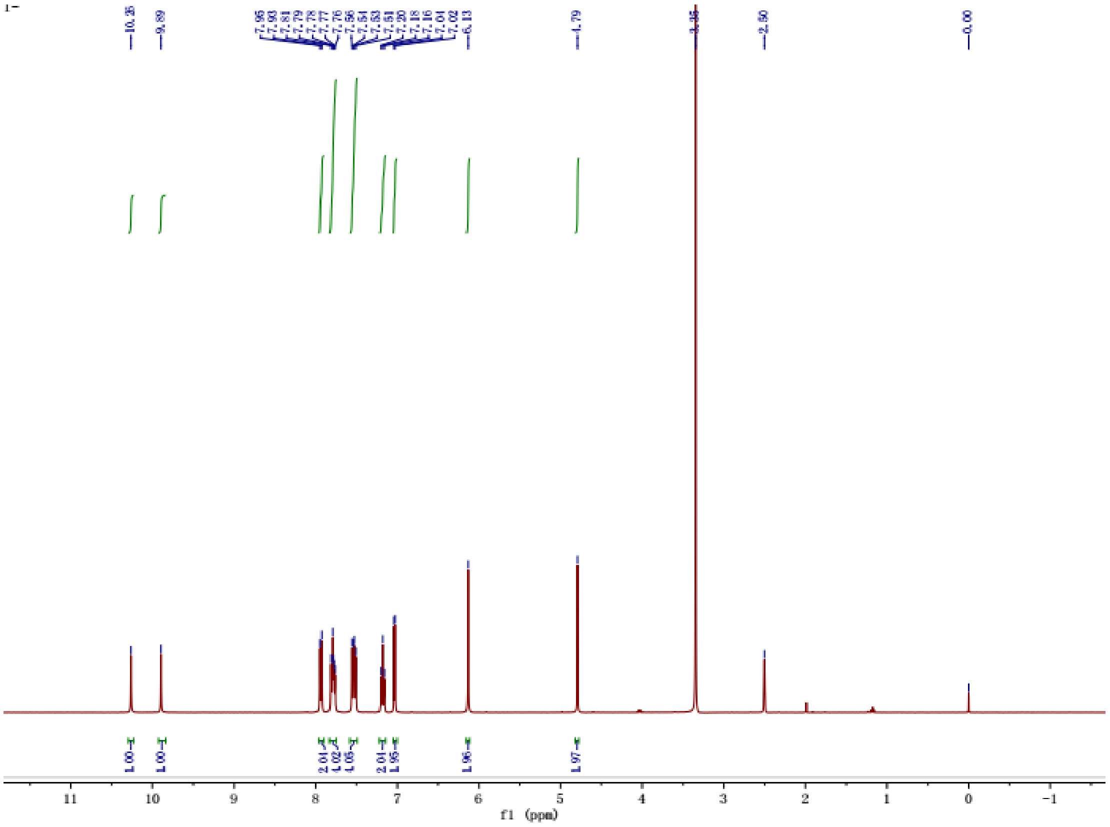

^{1}H-NMR spectrum of **B3**

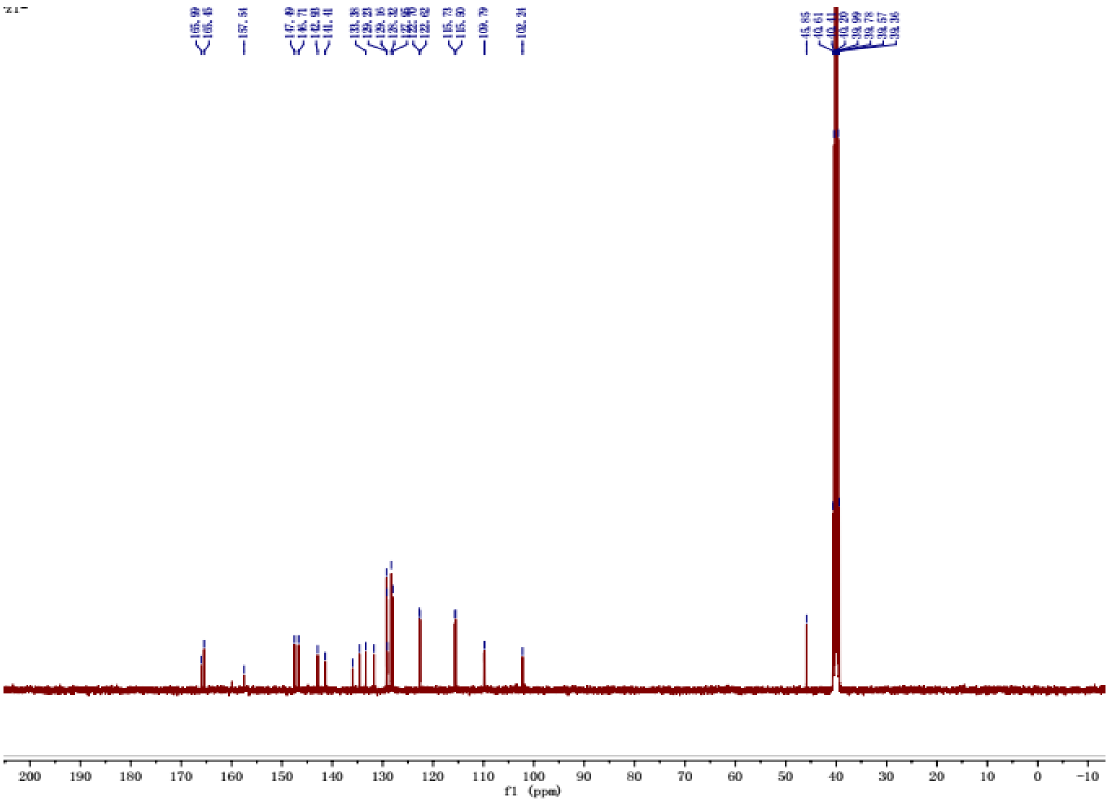

^{13}C-NMR spectrum of **B3**

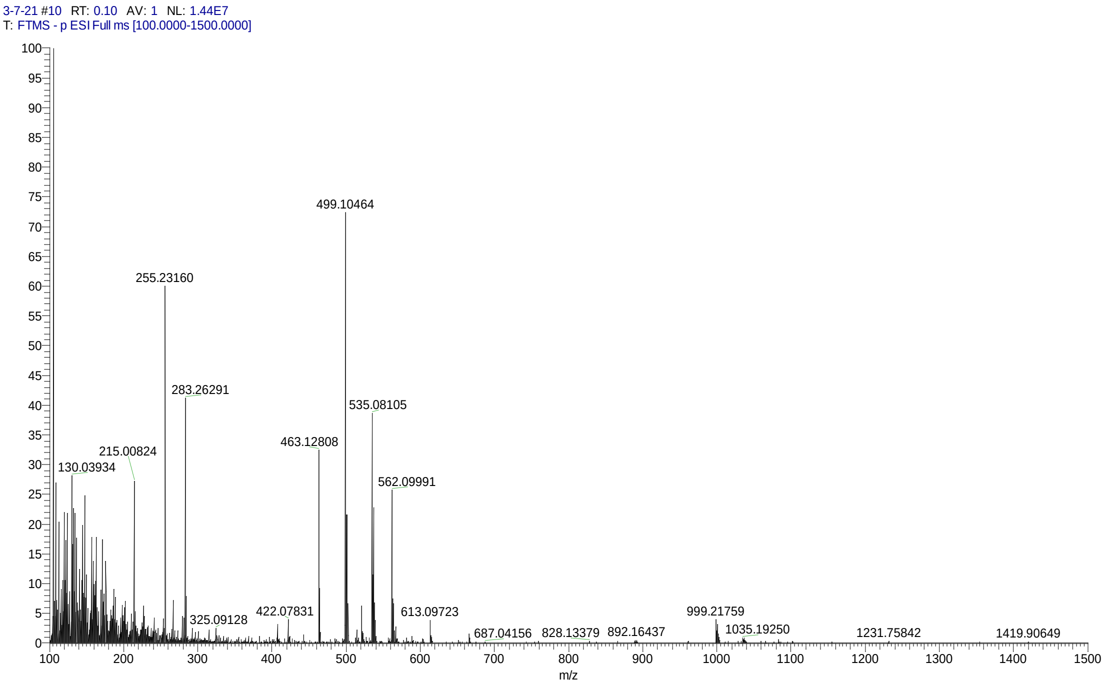

HRMS spectrum of **B4**

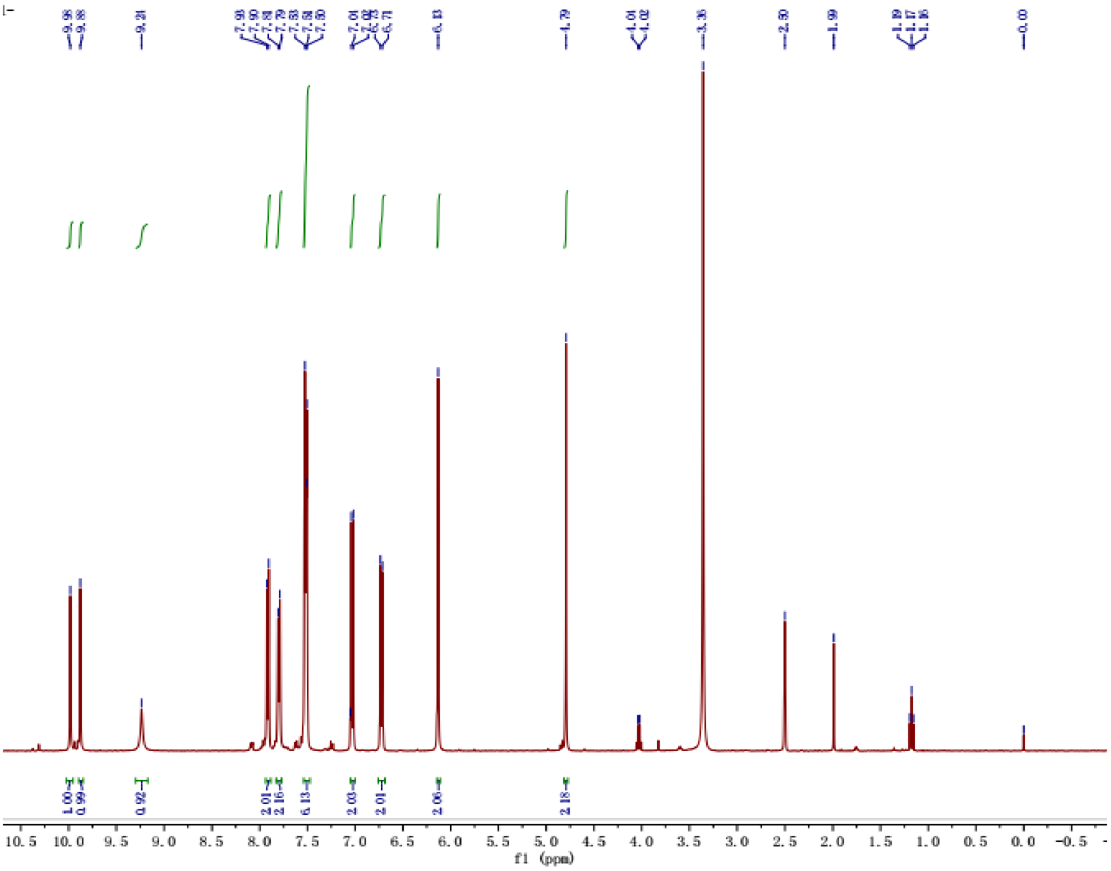

^{1}H-NMR spectrum of **B4**

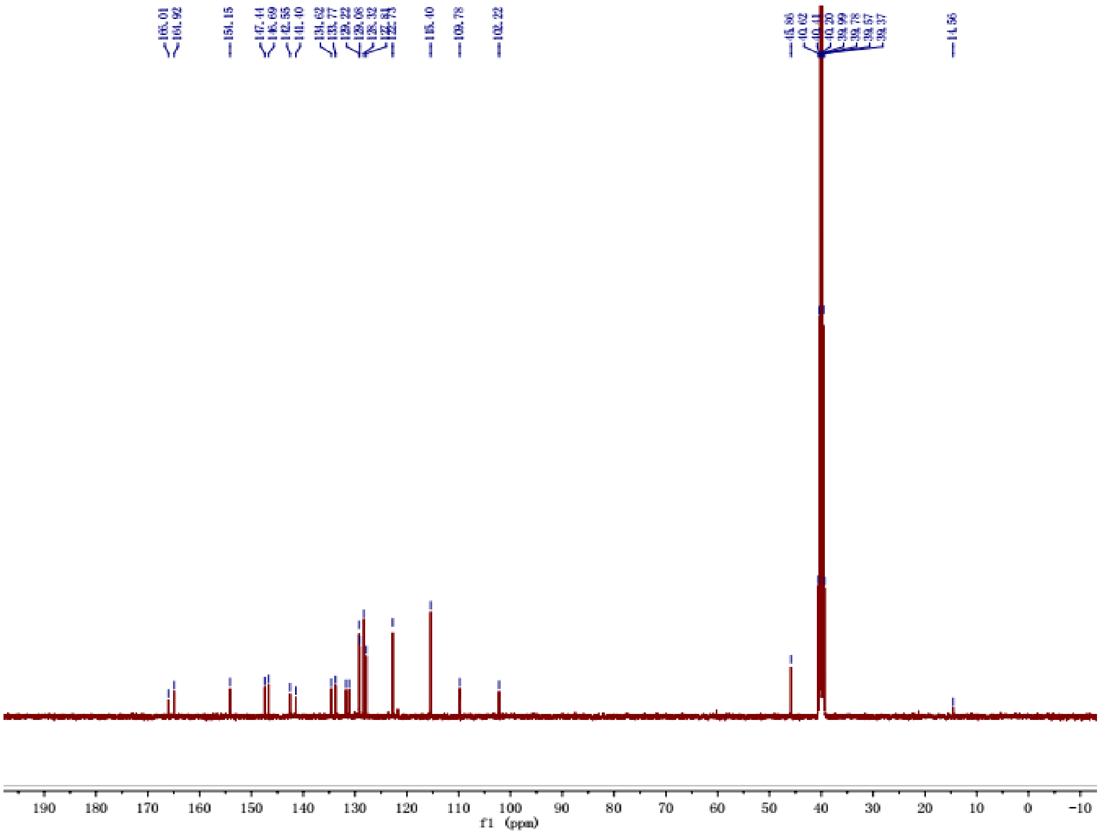

^{13}C-NMR spectrum of **B4**

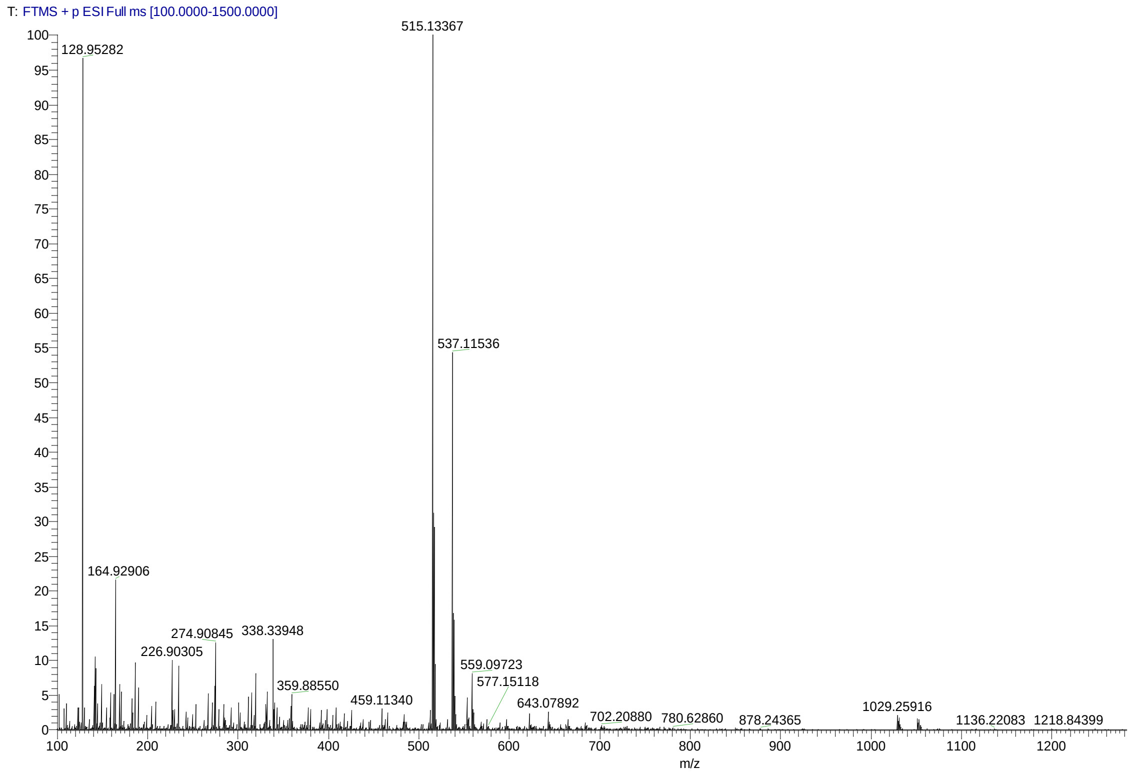

HRMS spectrum of **B5**

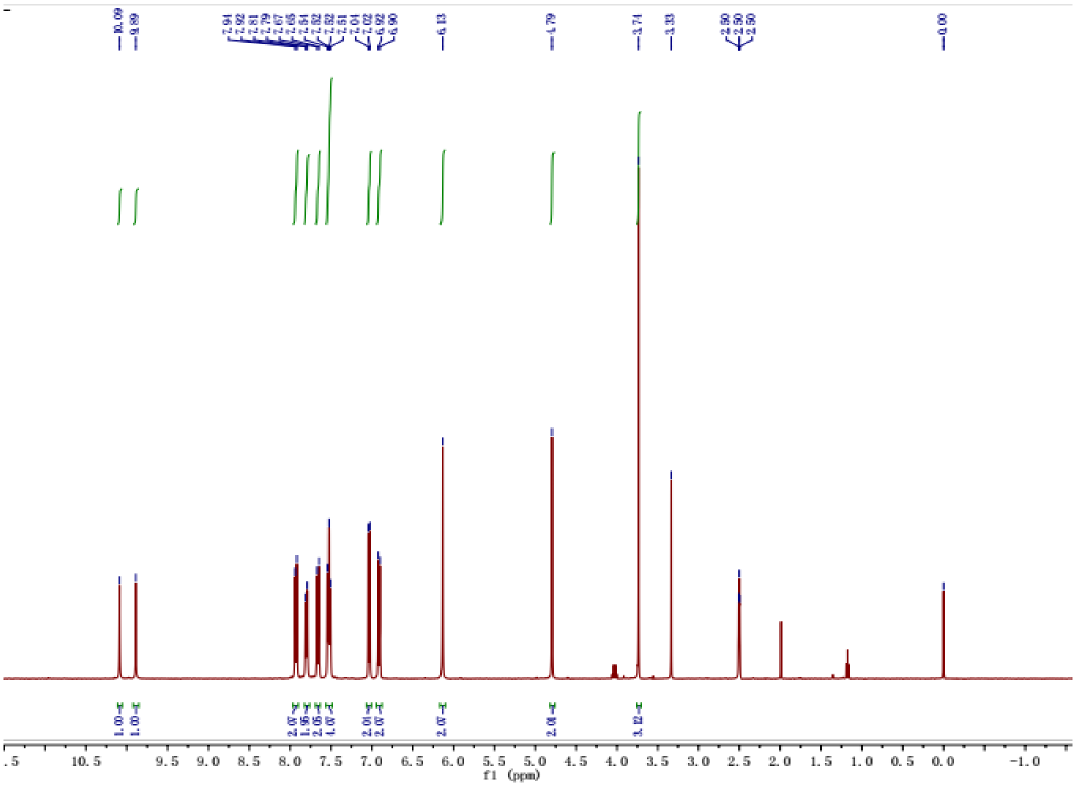

^{1}H-NMR spectrum of **B5**

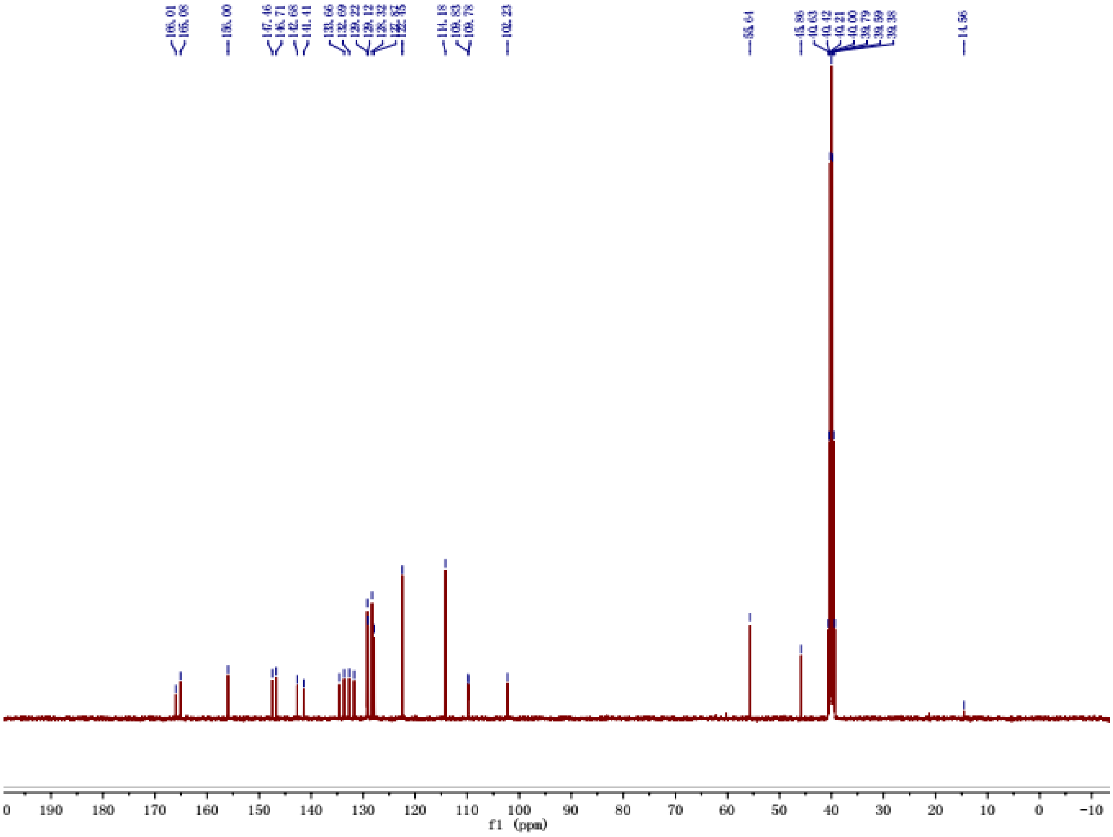

^{13}C-NMR spectrum of **B5**

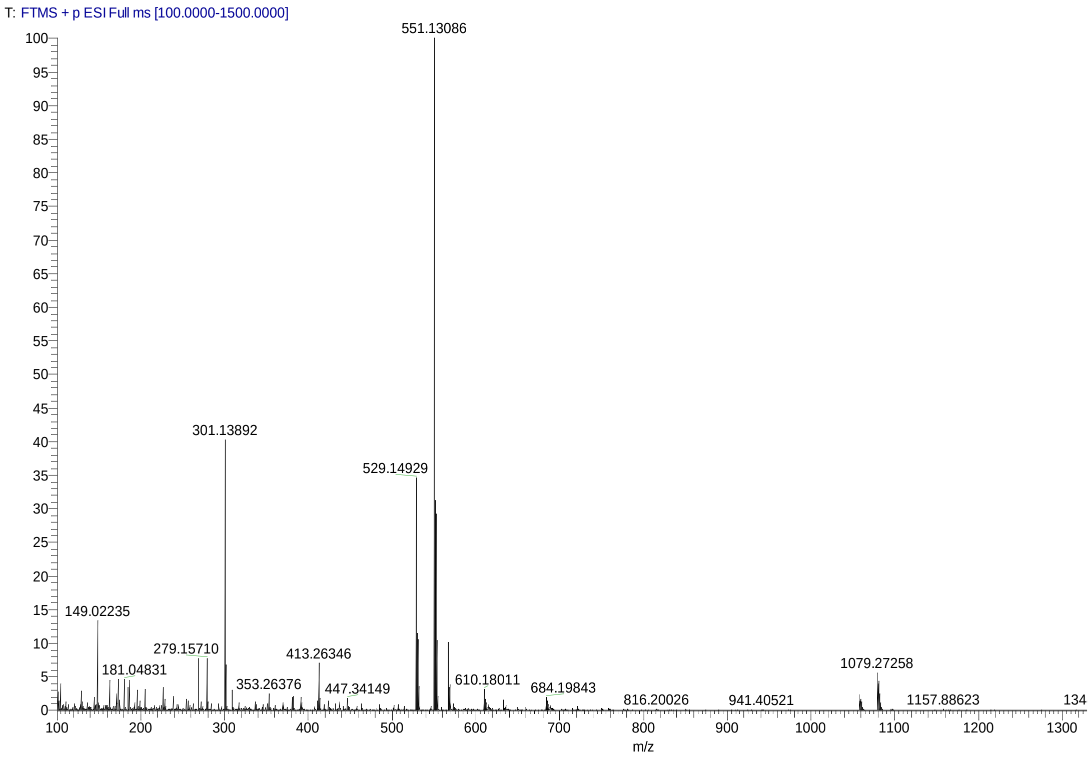

HRMS spectrum of **B6**

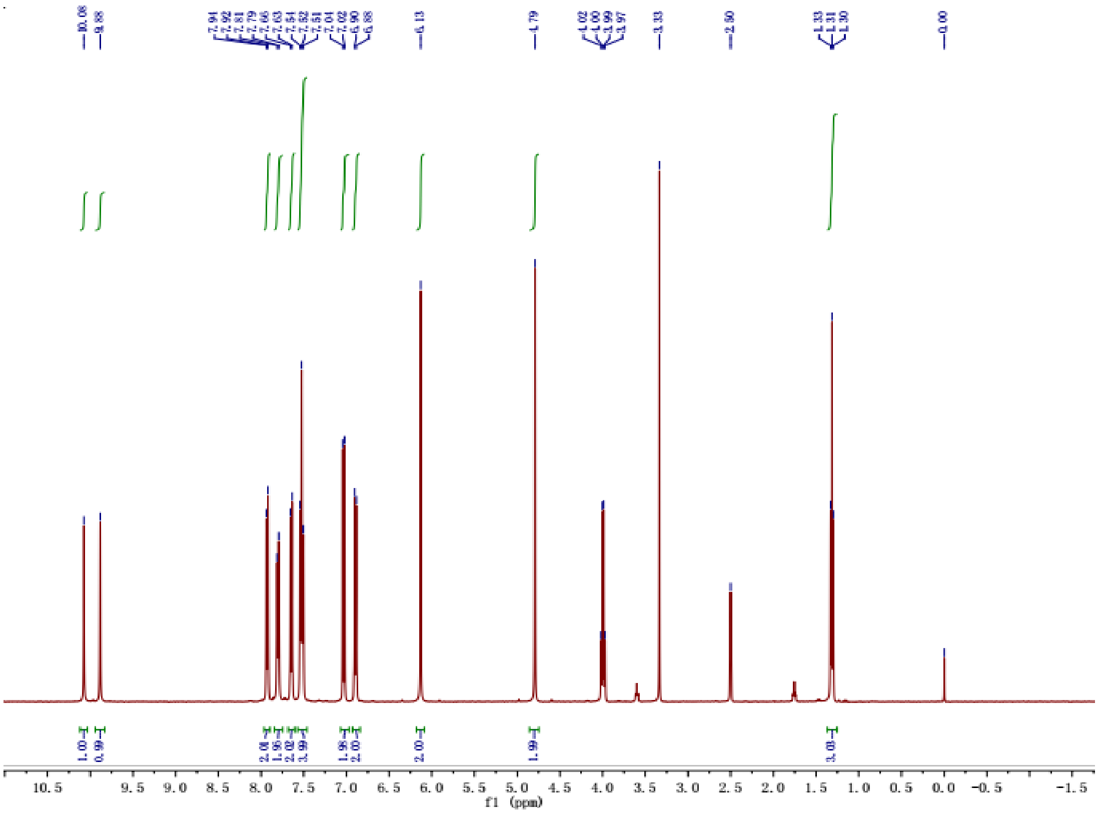

^{1}H-NMR spectrum of **B6**

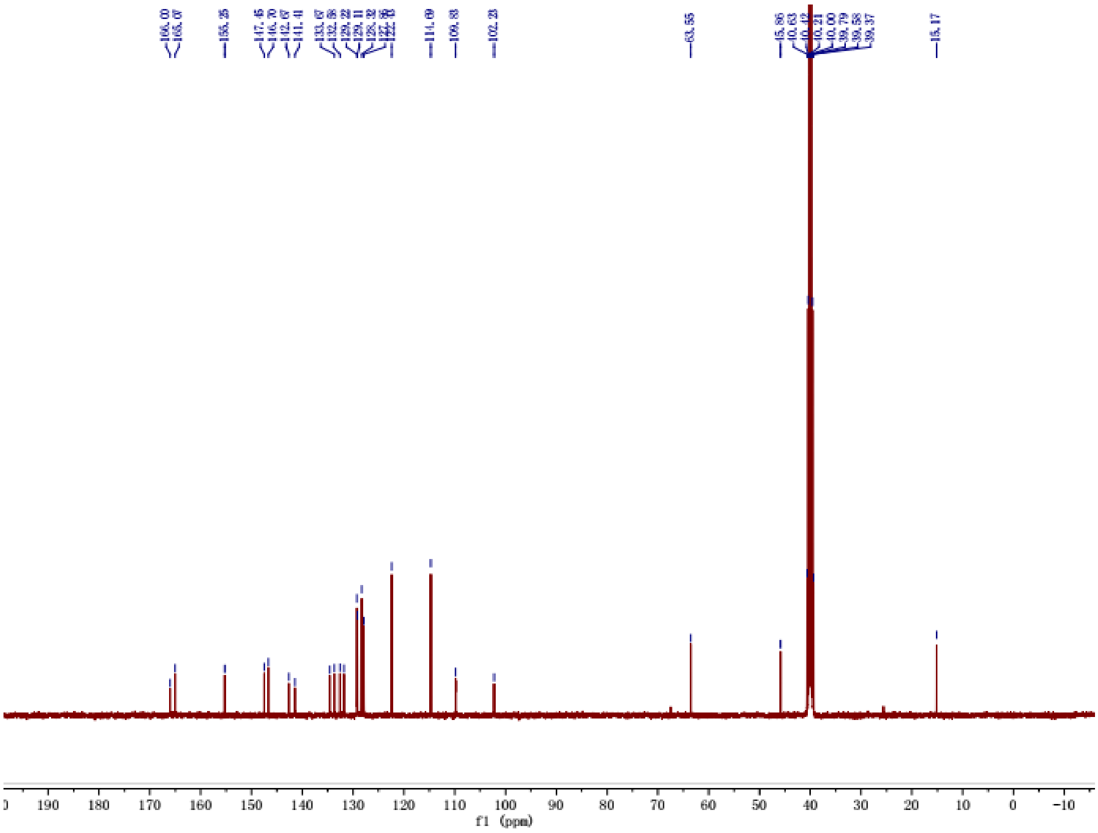

^{13}C-NMR spectrum of **B6**

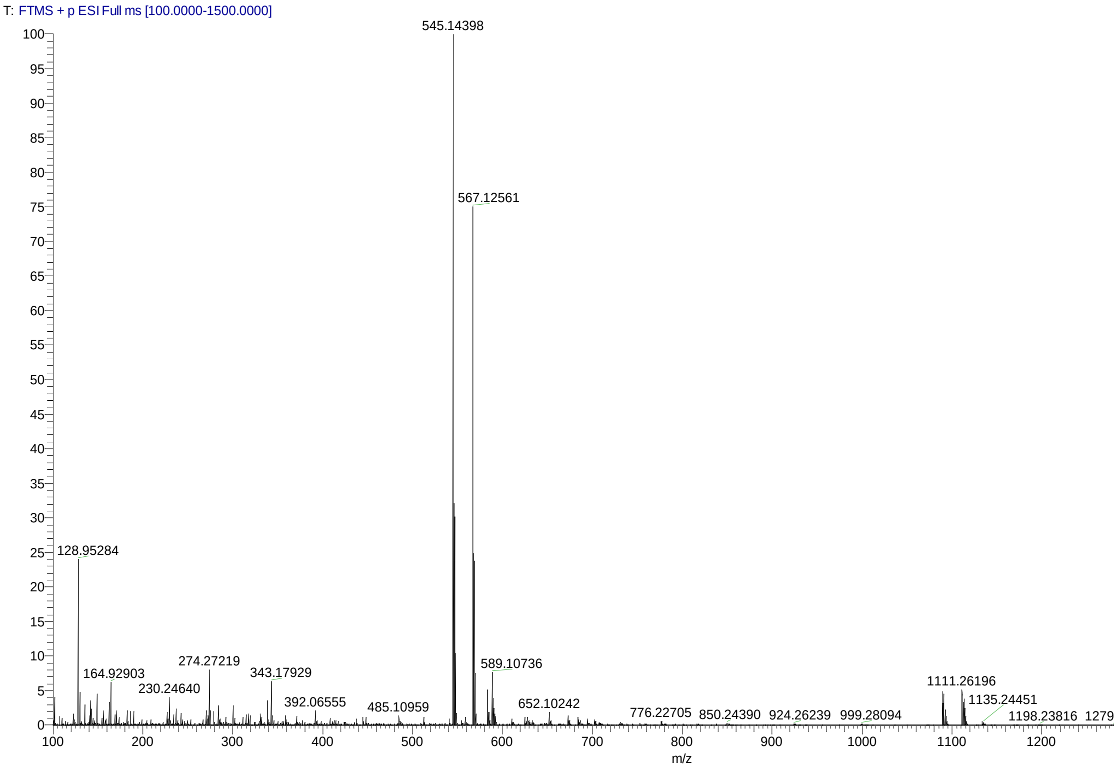

HRMS spectrum of **B7`**

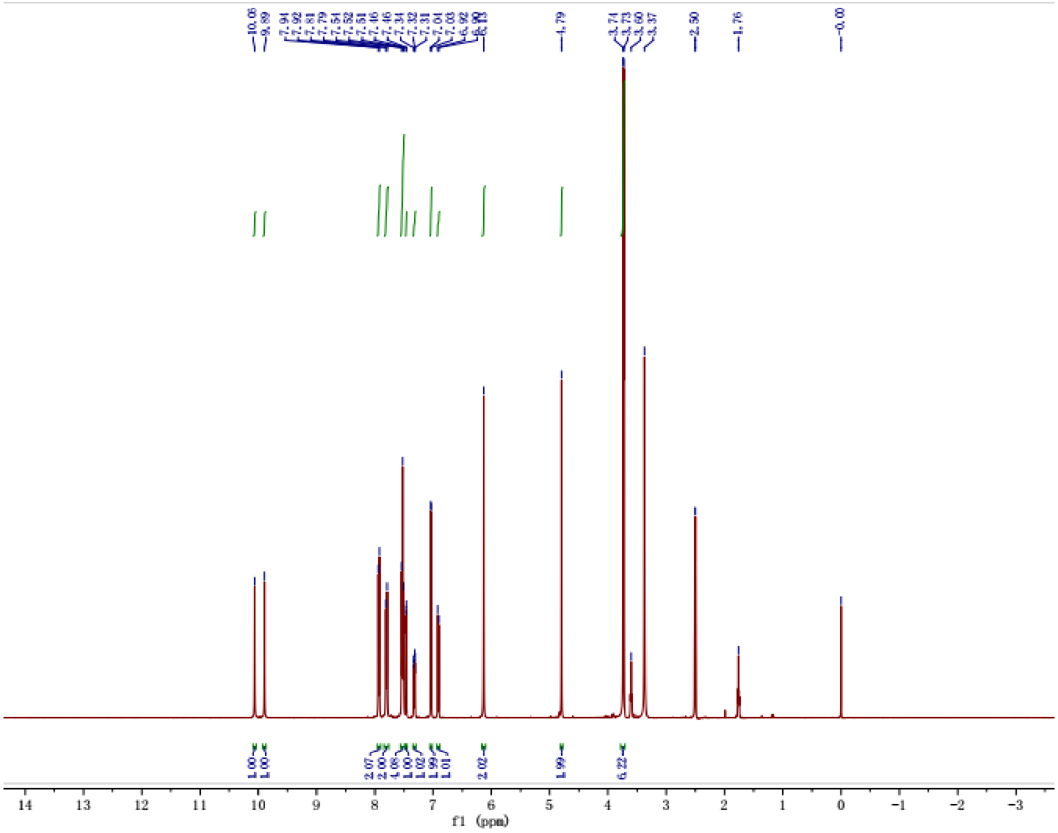

^{1}H-NMR spectrum of **B7**

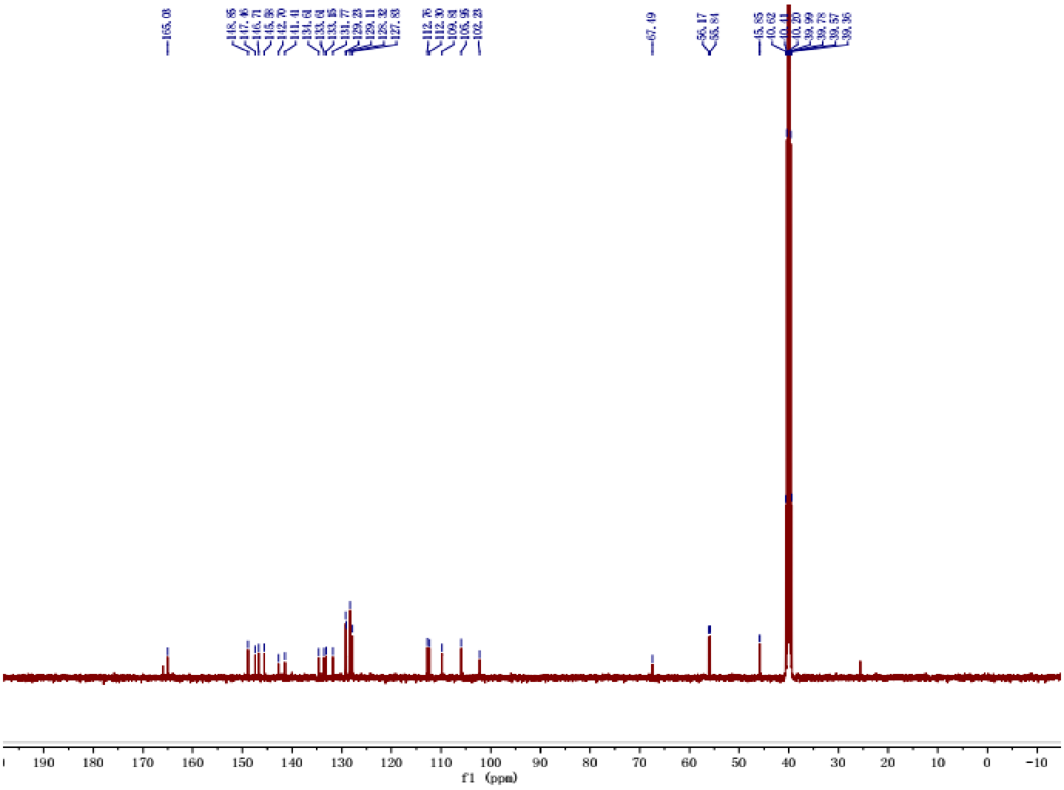

^{13}C-NMR spectrum of **B7**

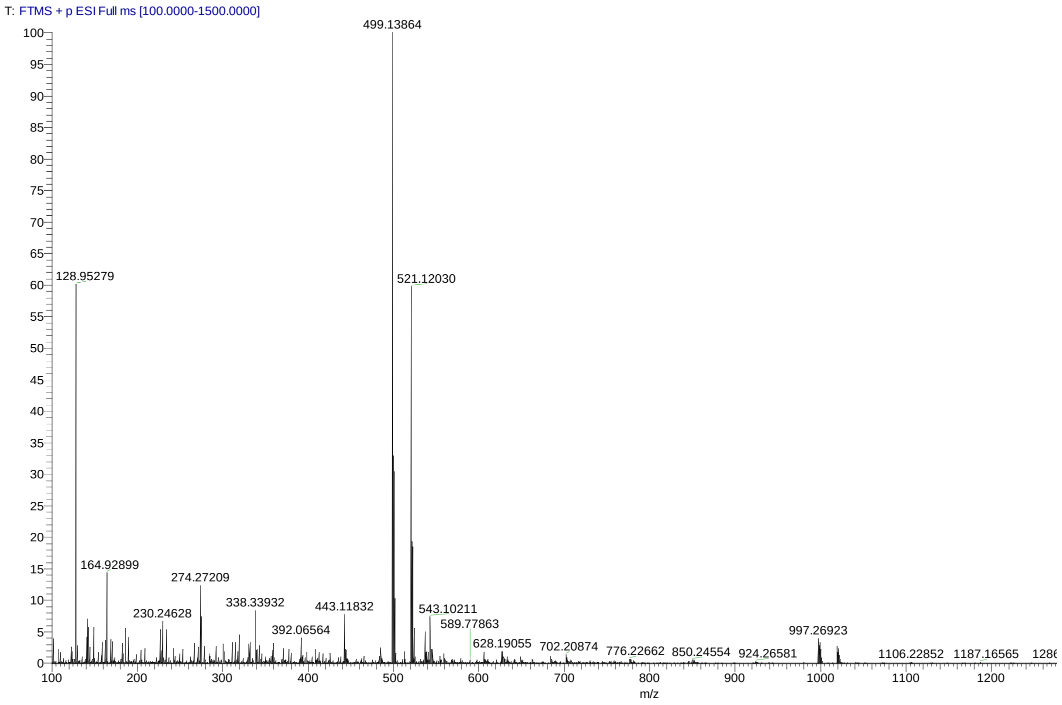

HRMS spectrum of **B8**

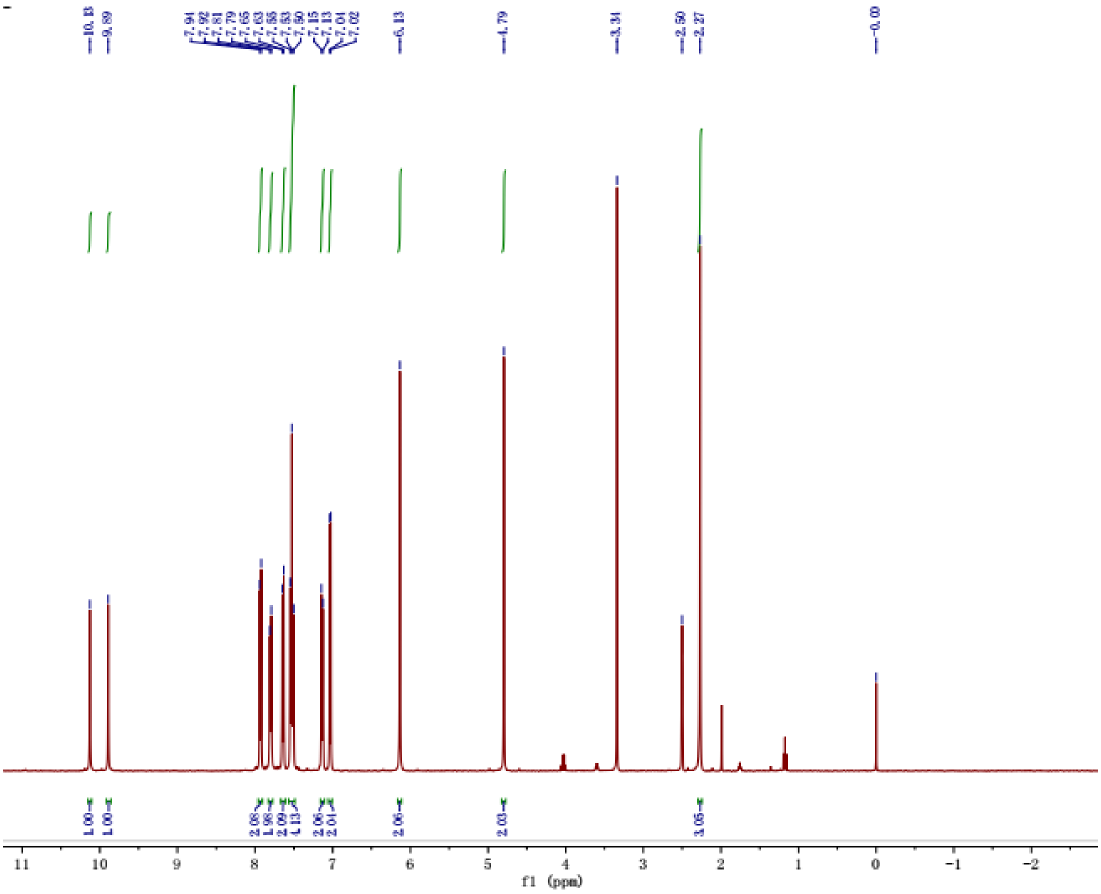

^{1}H-NMR spectrum of **B8**

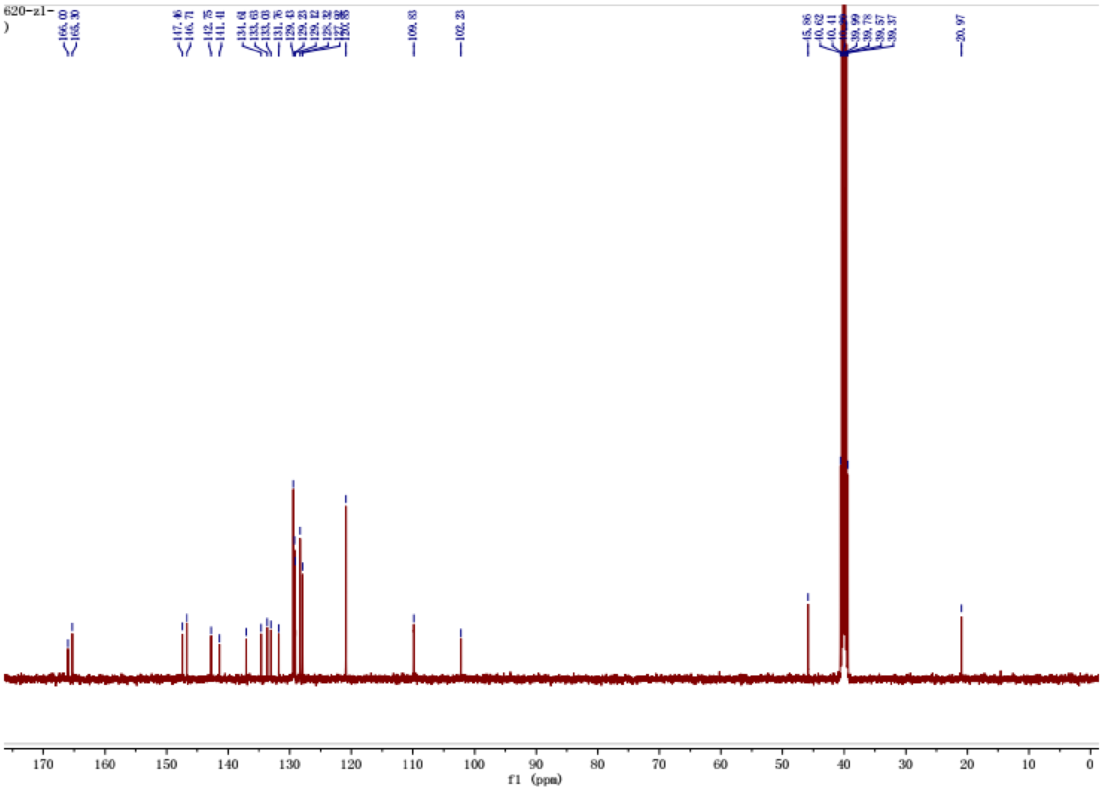

^{13}C-NMR spectrum of **B8**

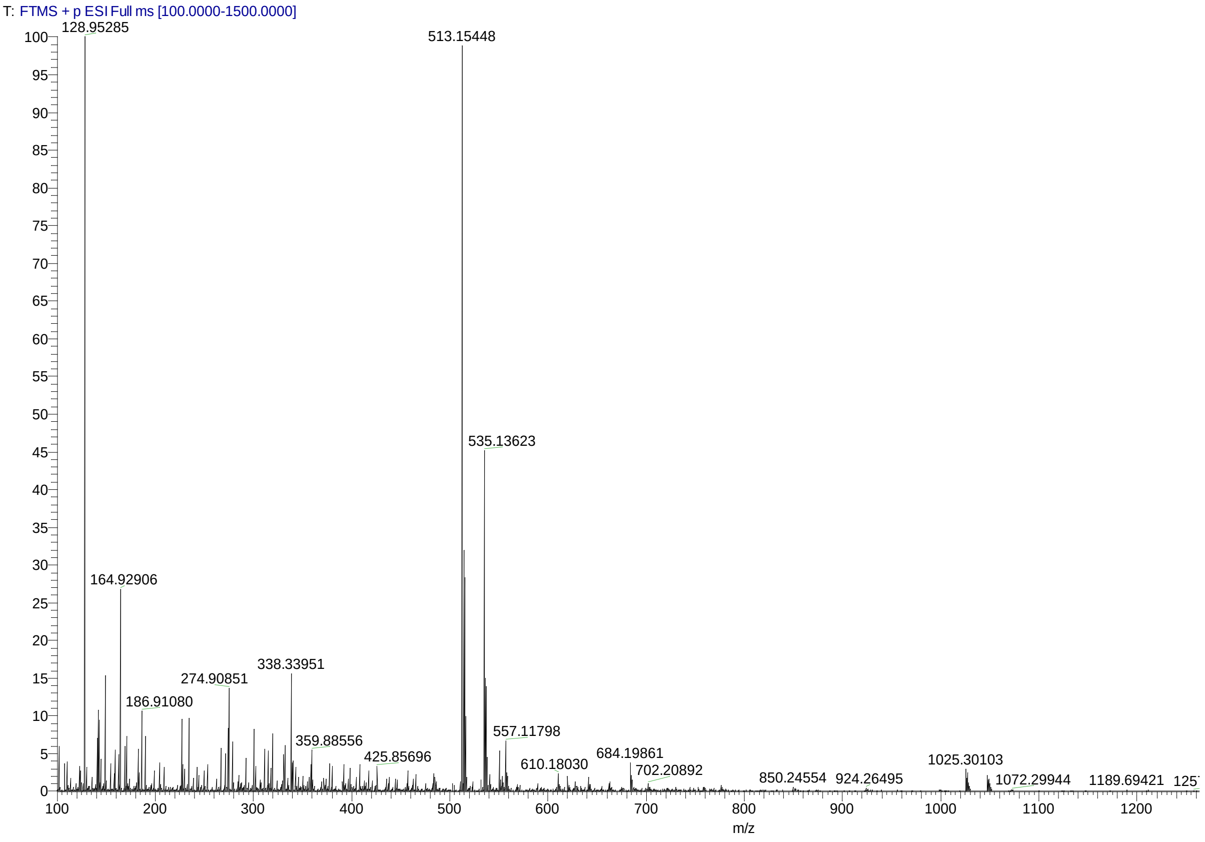

HRMS spectrum of **B9**

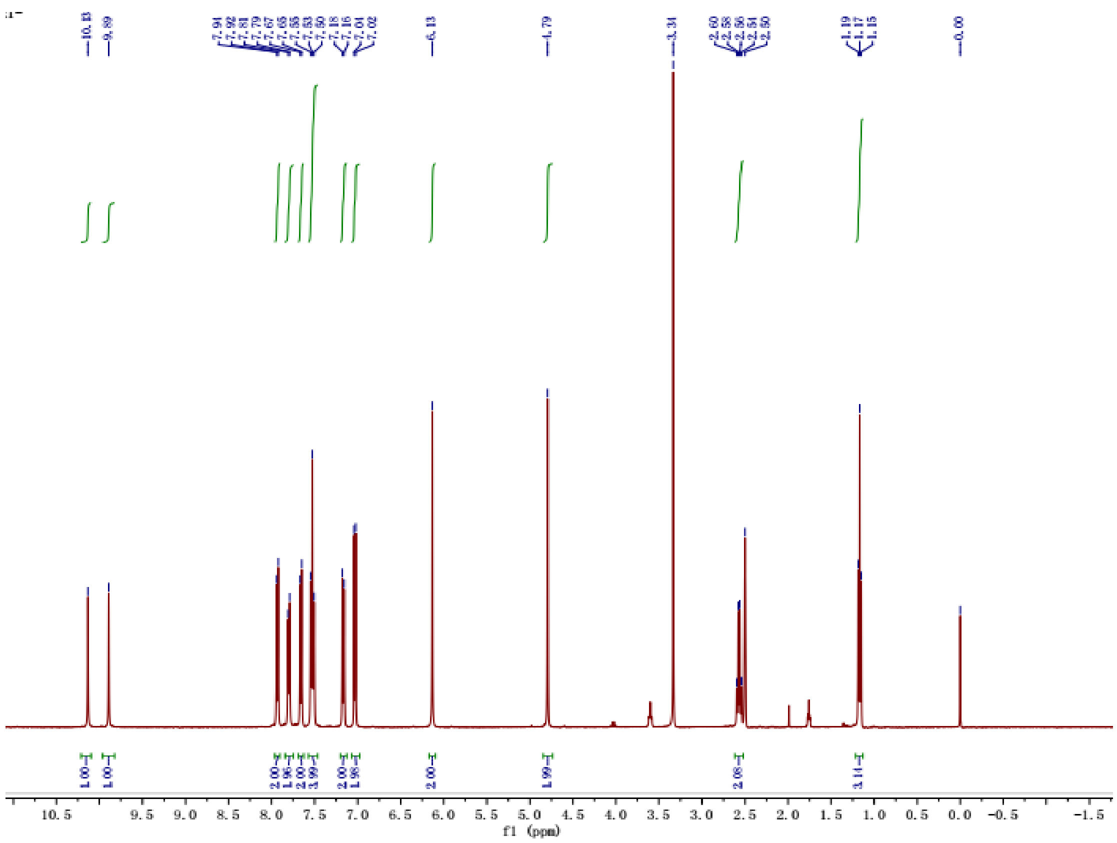

^{1}H-NMR spectrum of **B9**

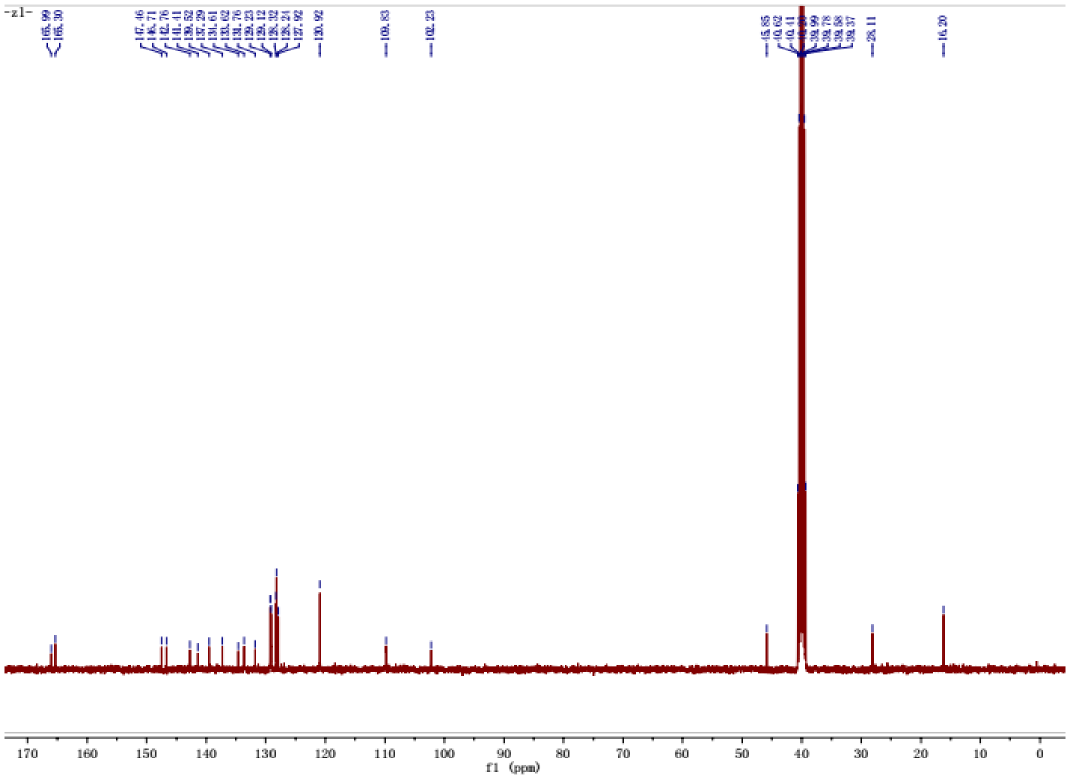

^{13}C-NMR spectrum of **B9**

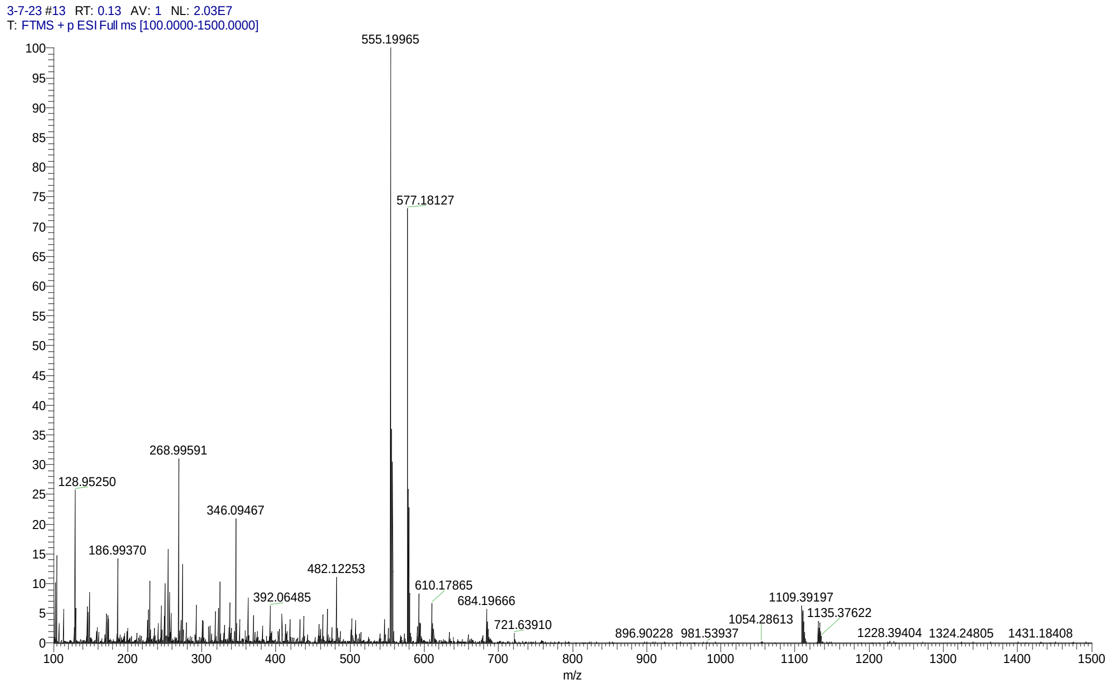

HRMS spectrum of **B10**

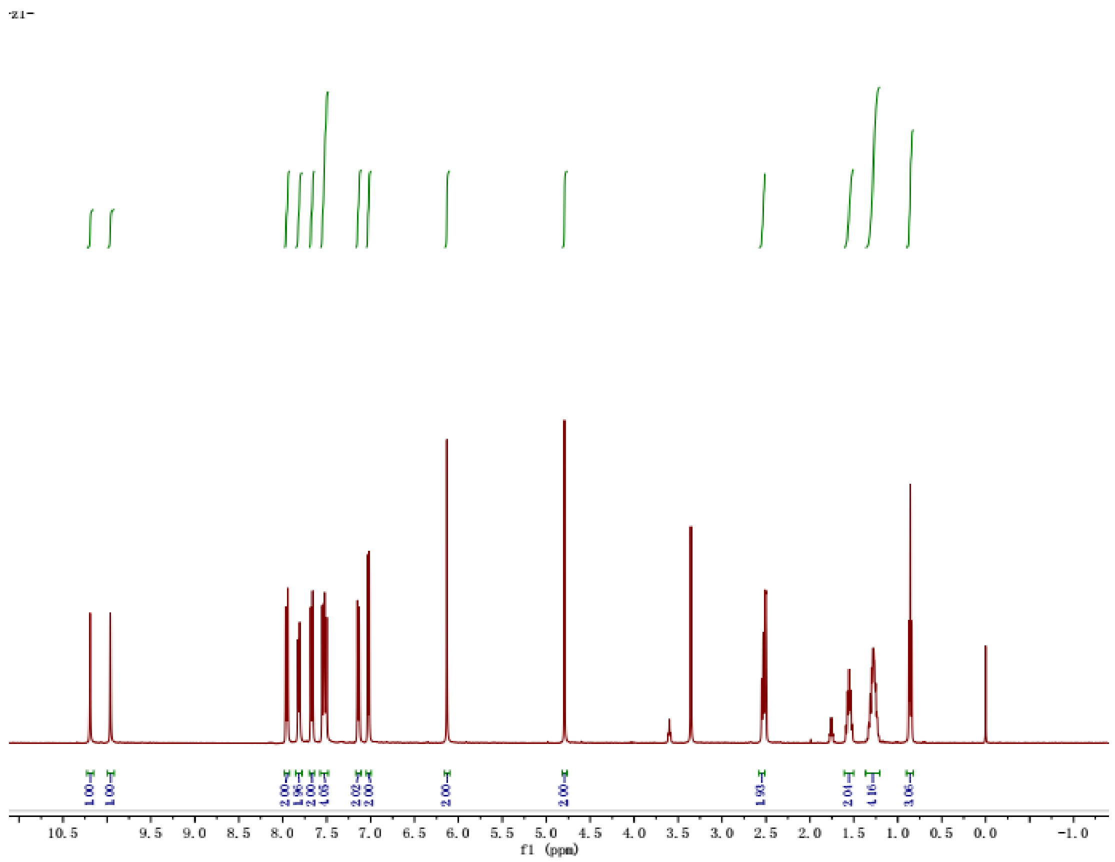

^{1}H-NMR spectrum of **B10**

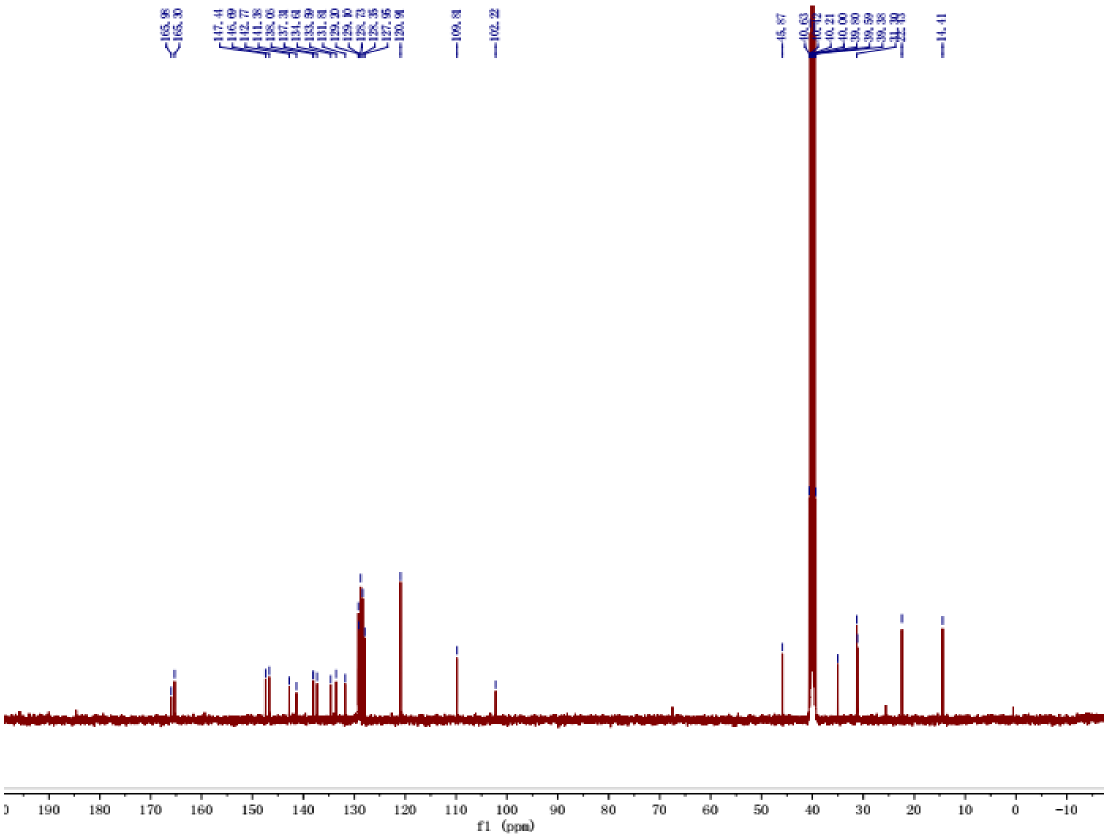

^{13}C-NMR spectrum of **B10**

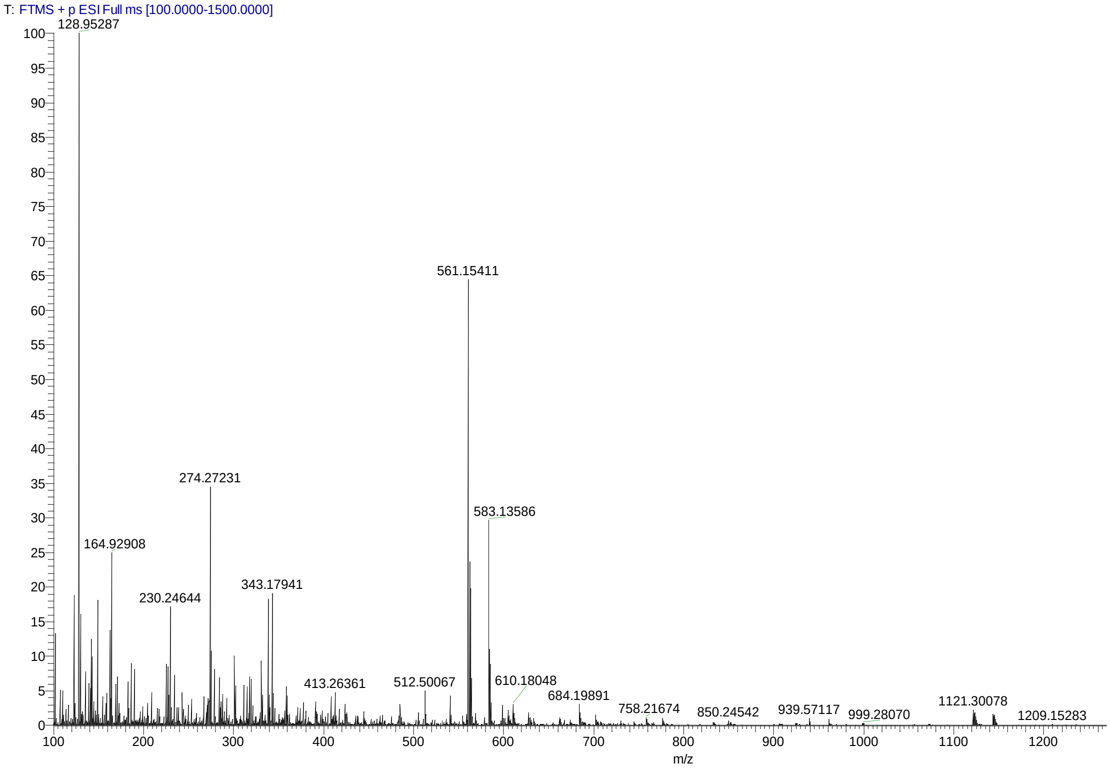

HRMS spectrum of **B11**

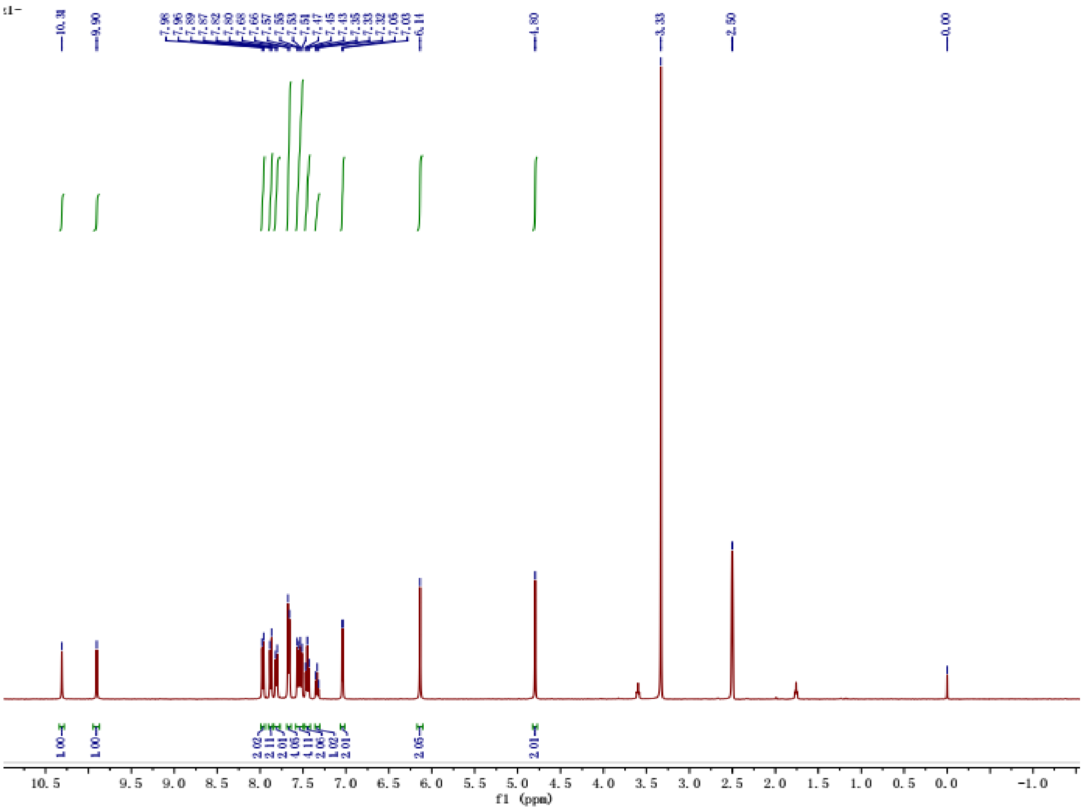

^{1}H-NMR spectrum of **B11**

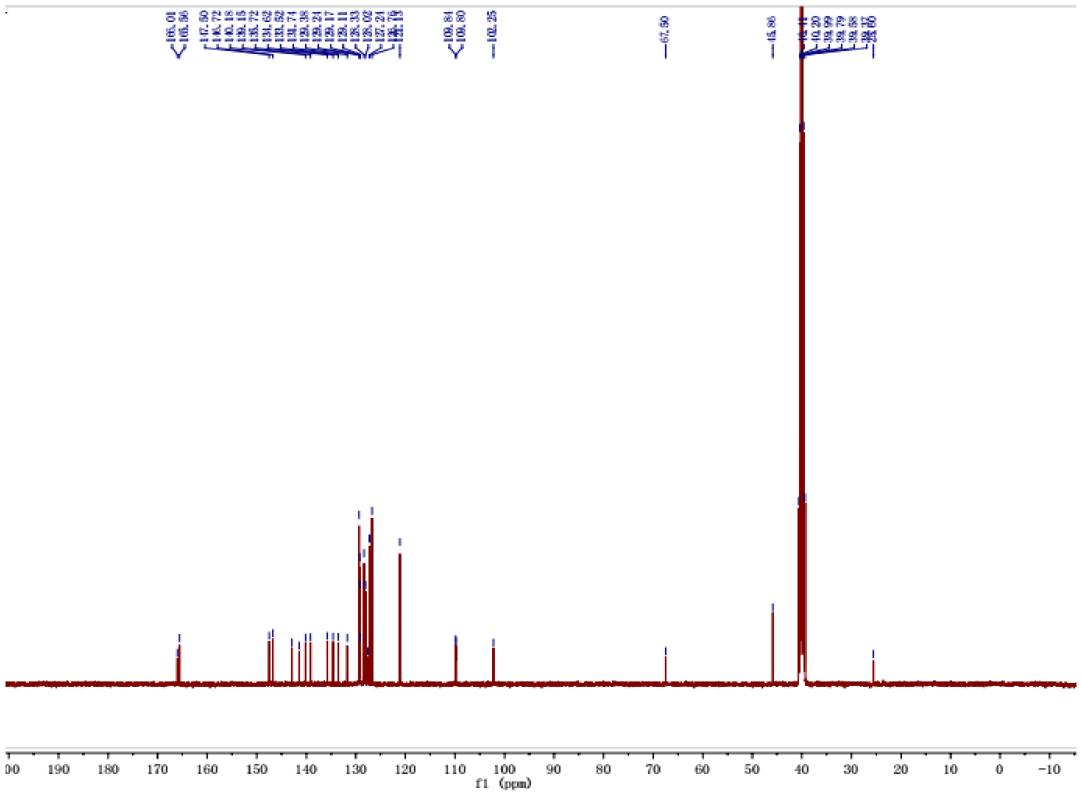

^{13}C-NMR spectrum of **B11**

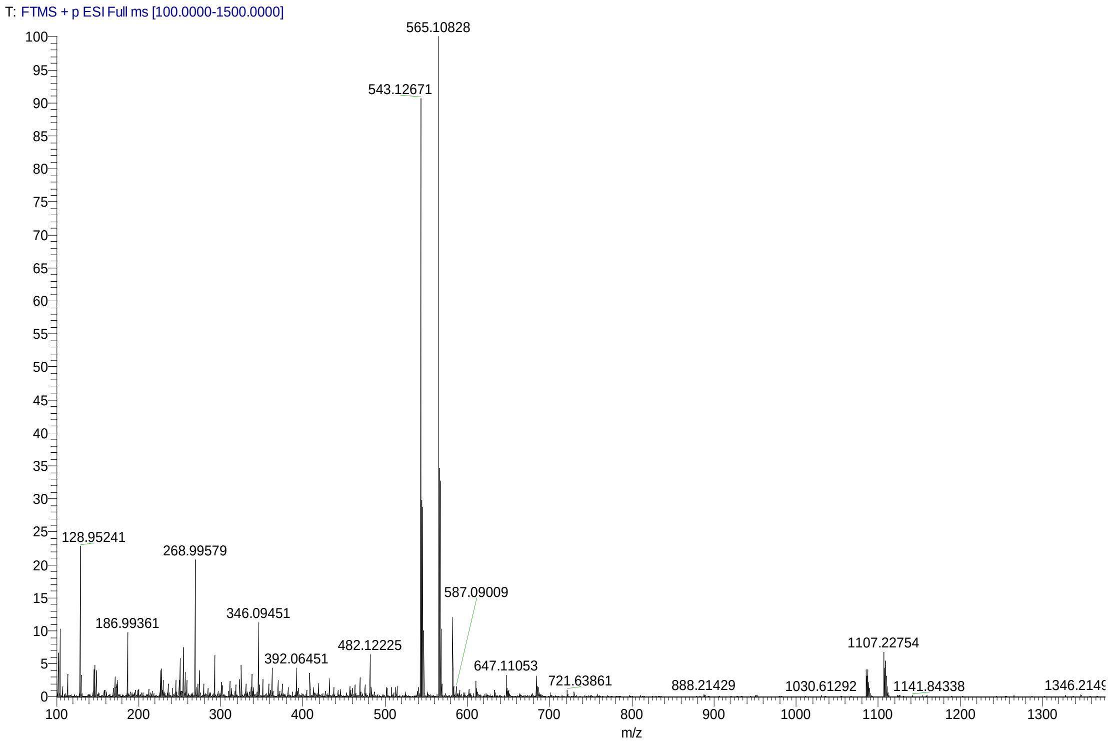

HRMS spectrum of **B12**

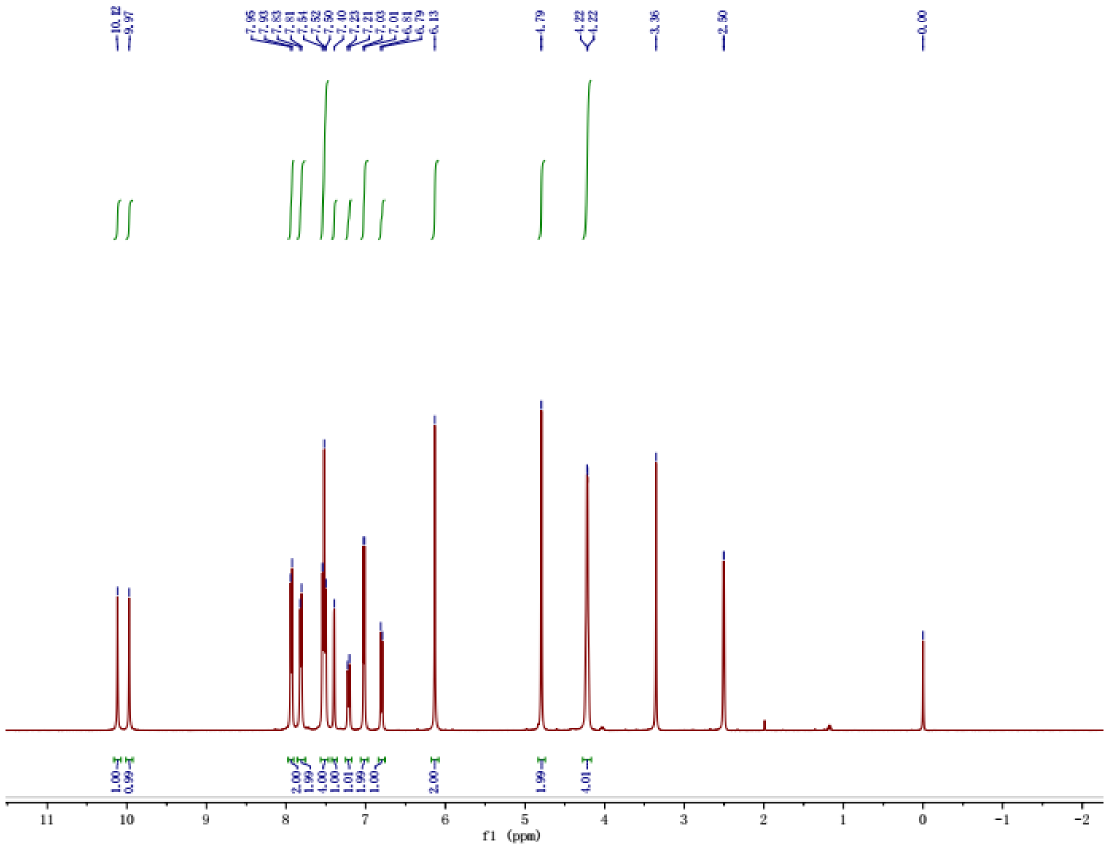

^{1}H-NMR spectrum of **B12**

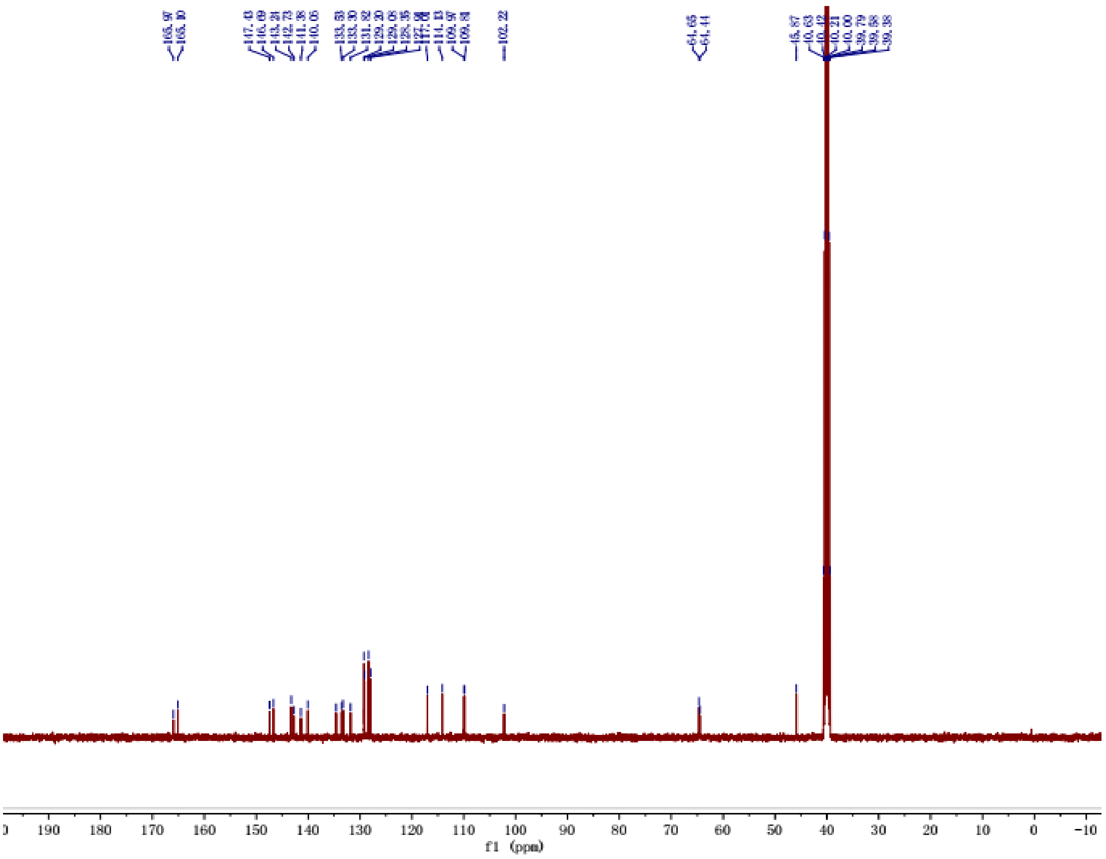

^{13}C-NMR spectrum of **B12**

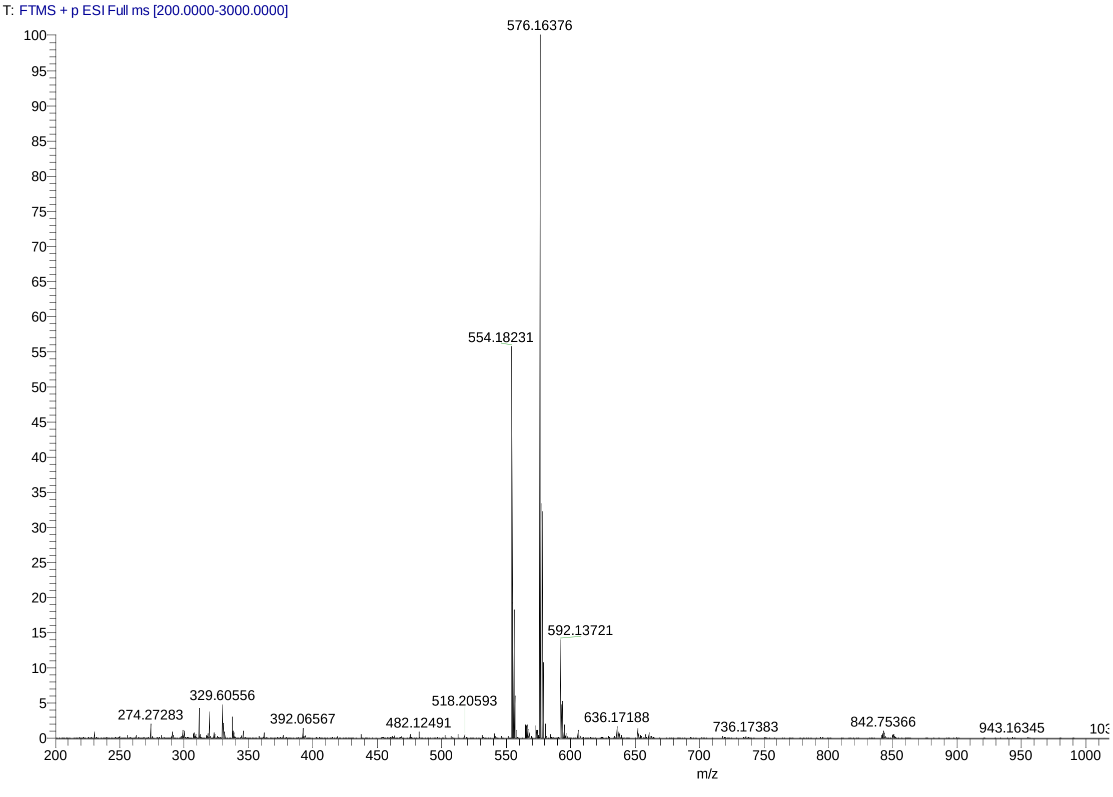

HRMS spectrum of **B13**

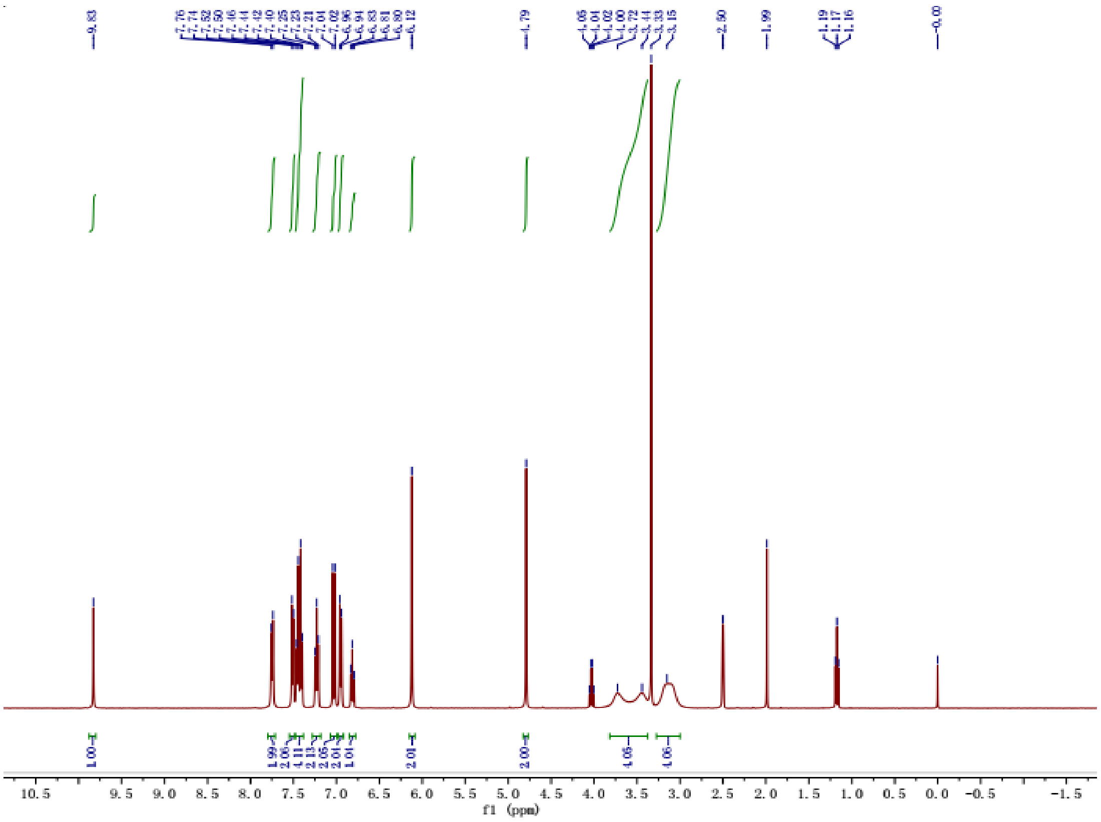

^{1}H-NMR spectrum of **B13**

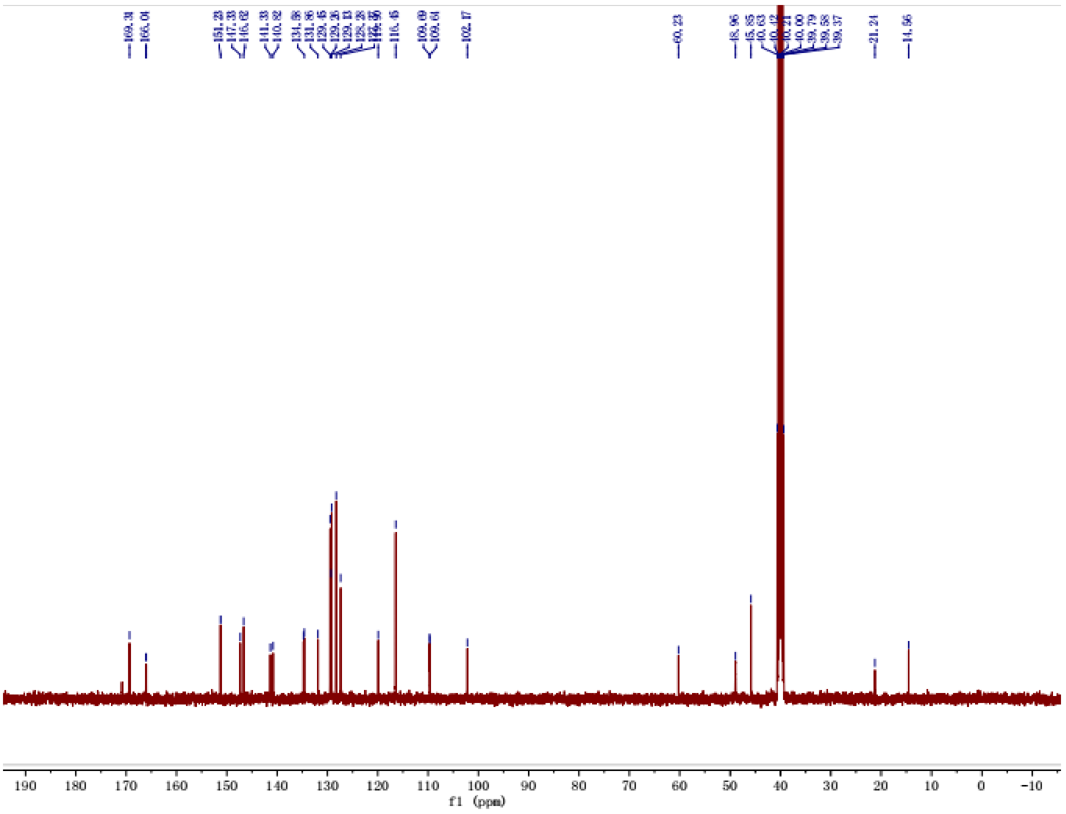

^{13}C-NMR spectrum of **B13**

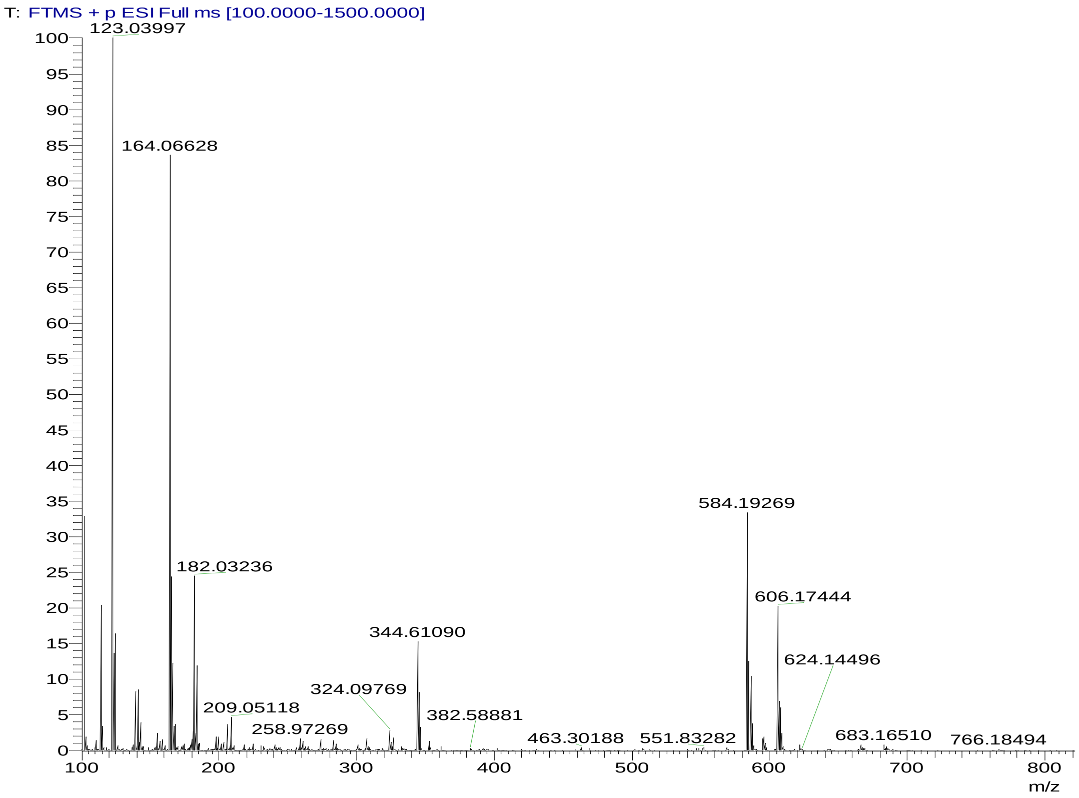

HRMS spectrum of **B14**

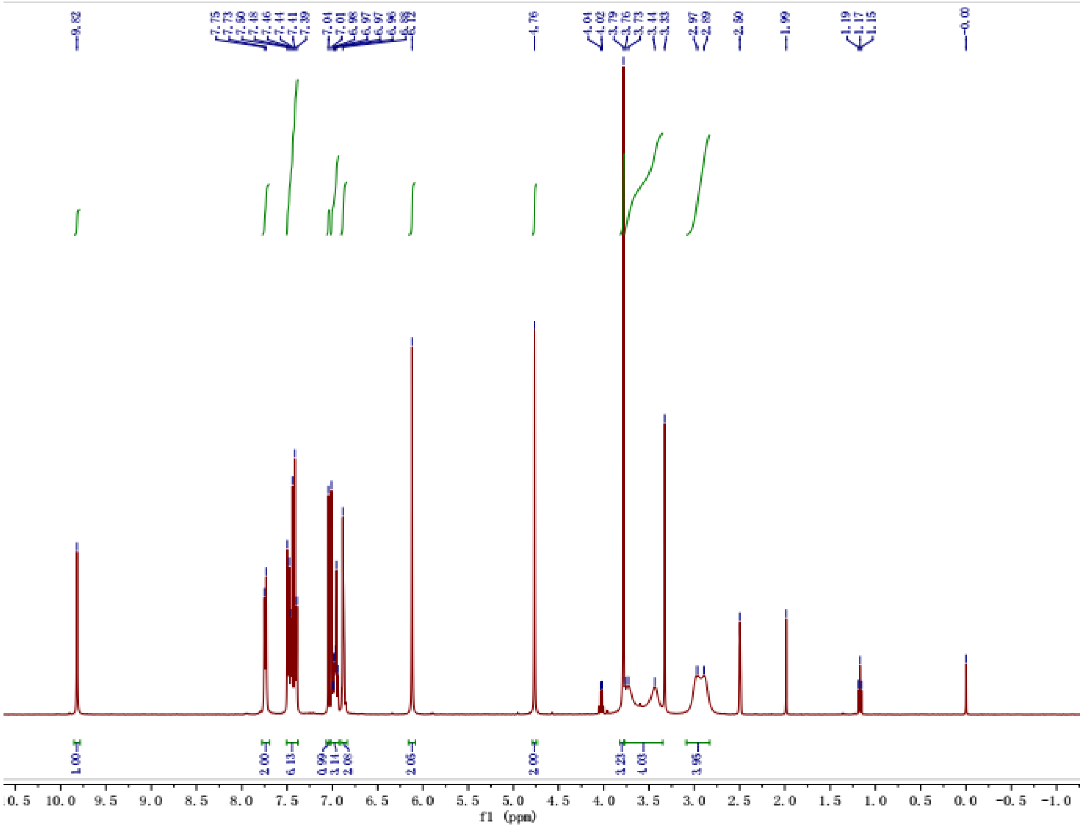

^{1}H-NMR spectrum of **B14**

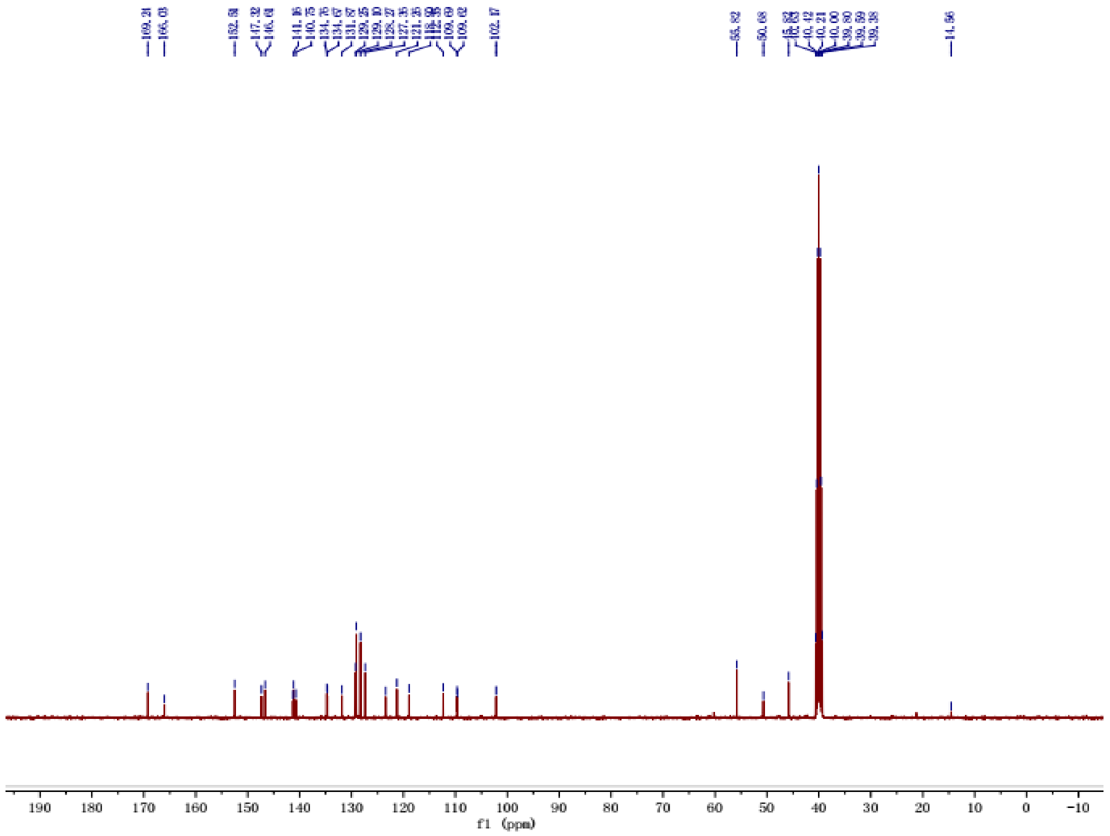

^{13}C-NMR spectrum of **B14**

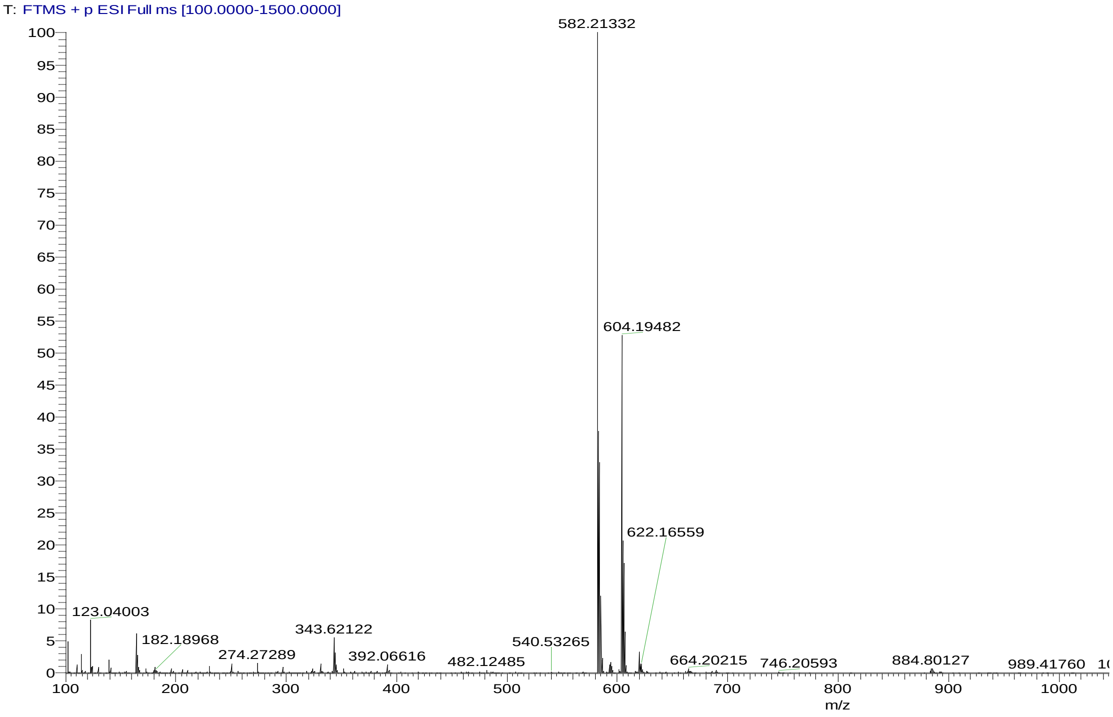

HRMS spectrum of **B15**

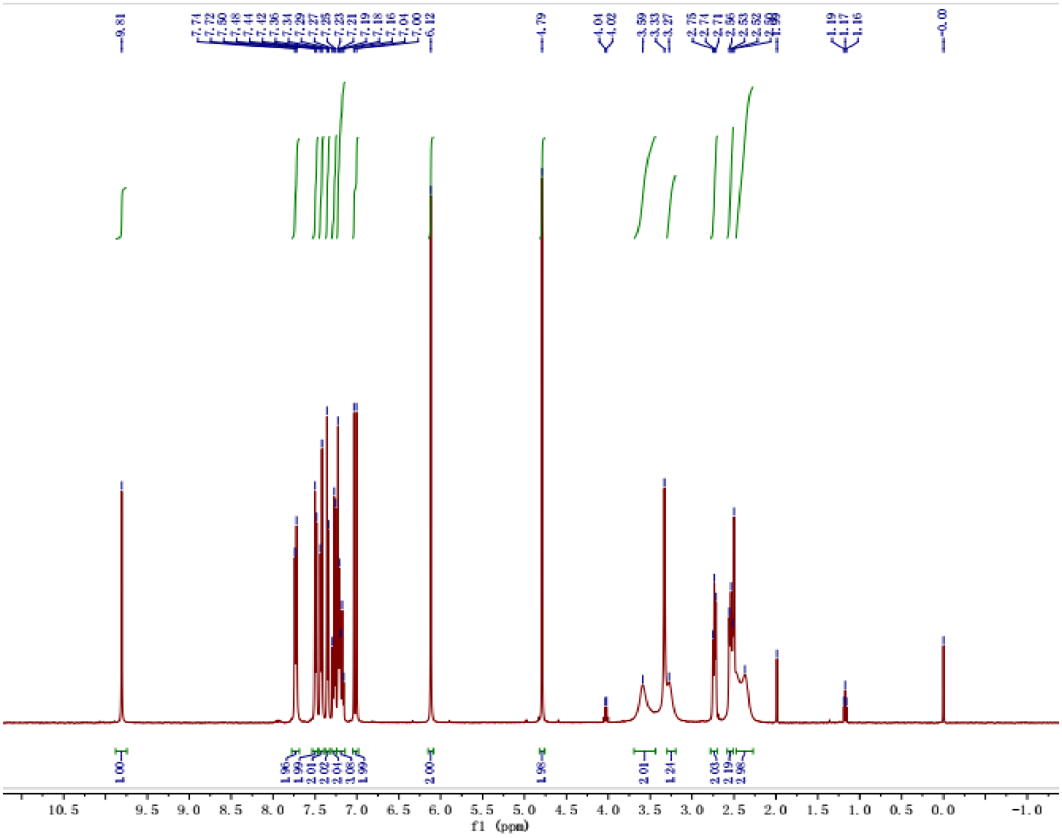

^{1}H-NMR spectrum of **B15**

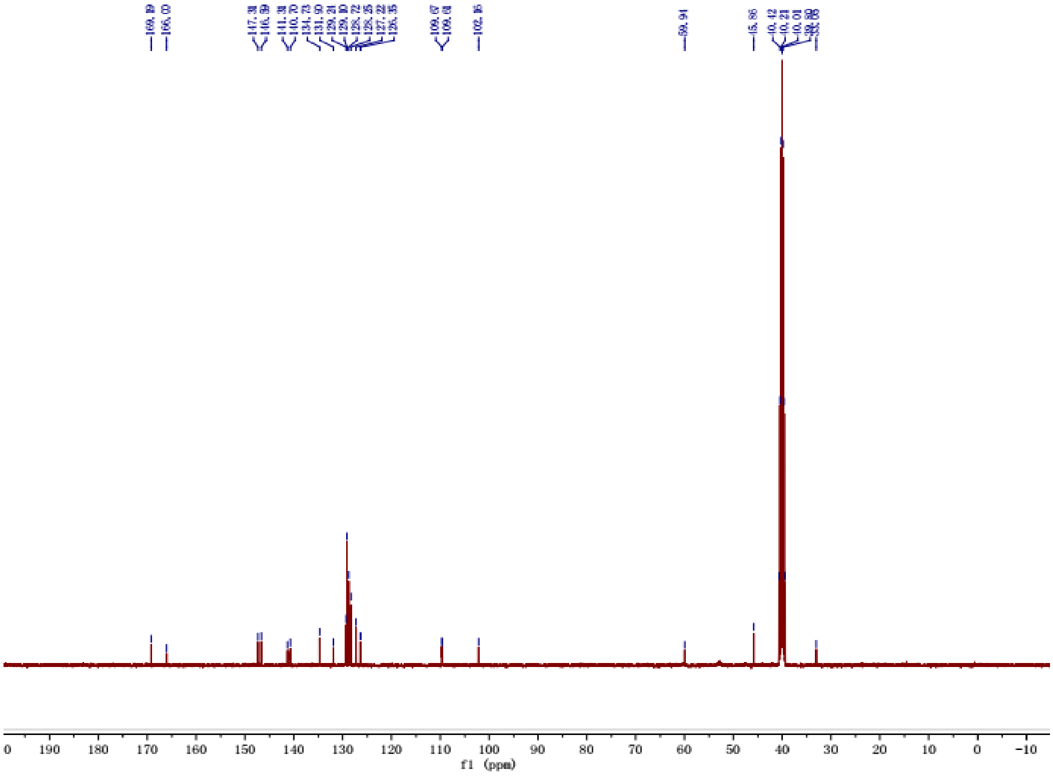

^{13}C-NMR spectrum of **B15**

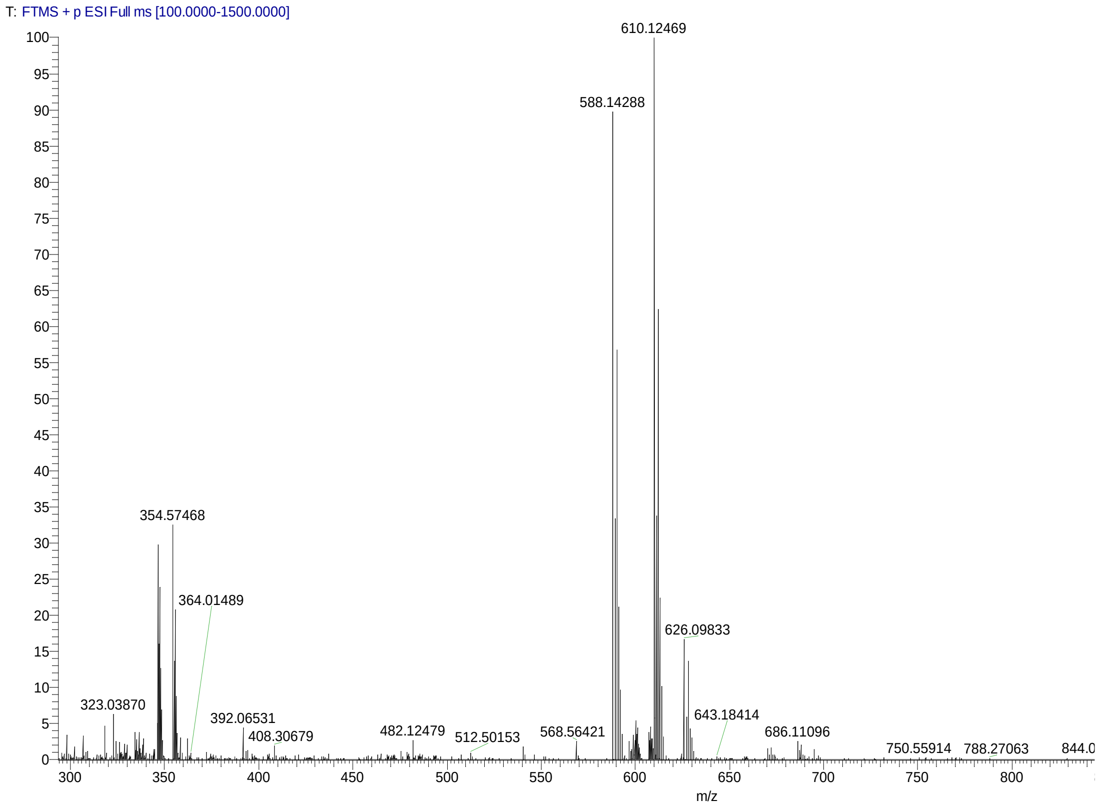

HRMS spectrum of **B16**

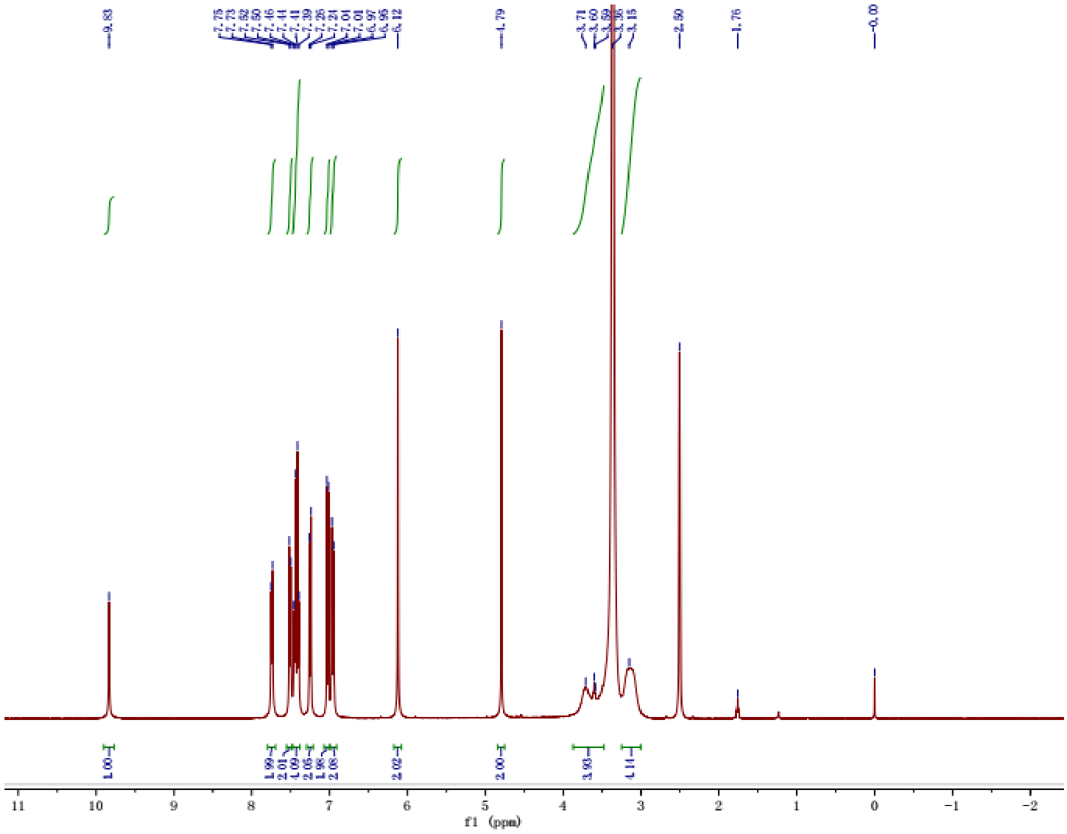

^{1}H-NMR spectrum of **B16**

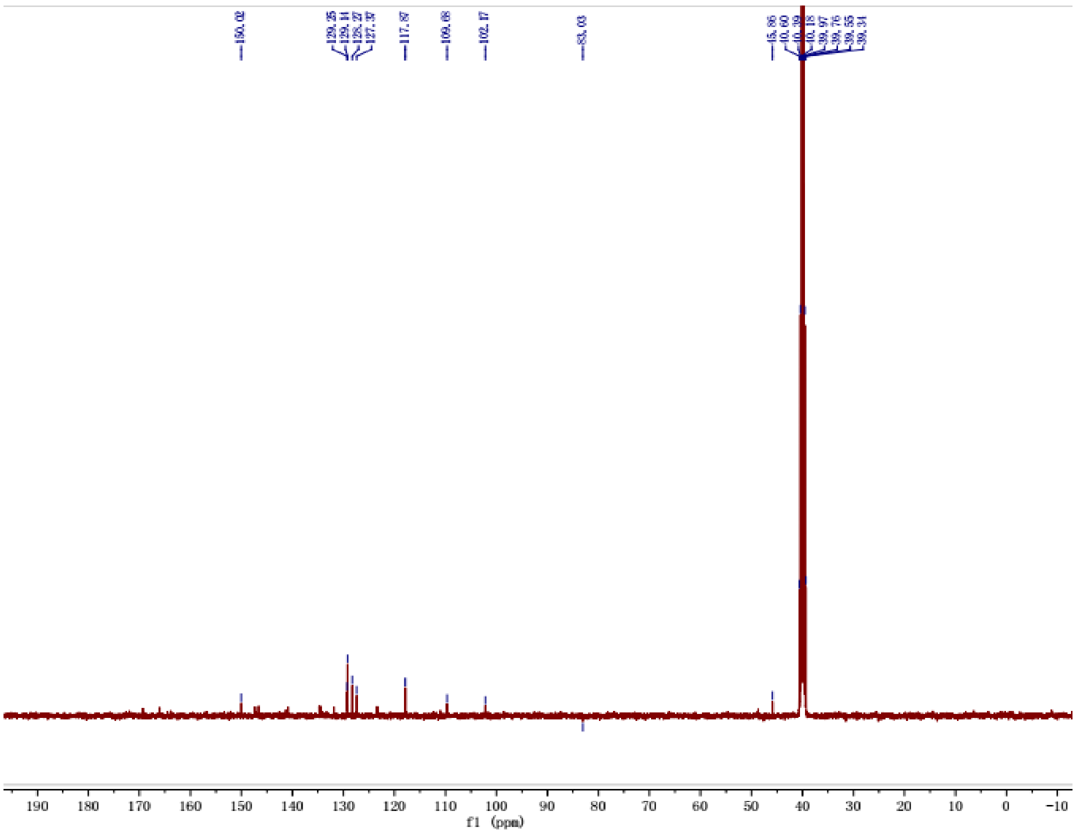

^{13}C-NMR spectrum of **B16**

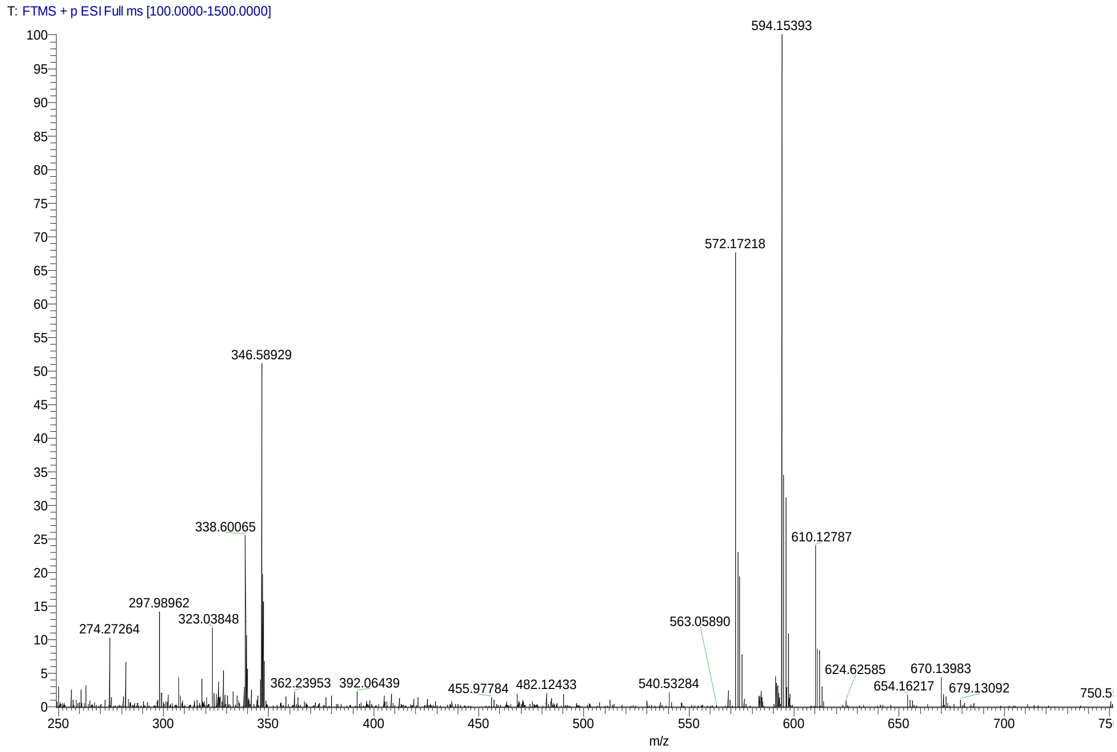

HRMS spectrum of **B17**

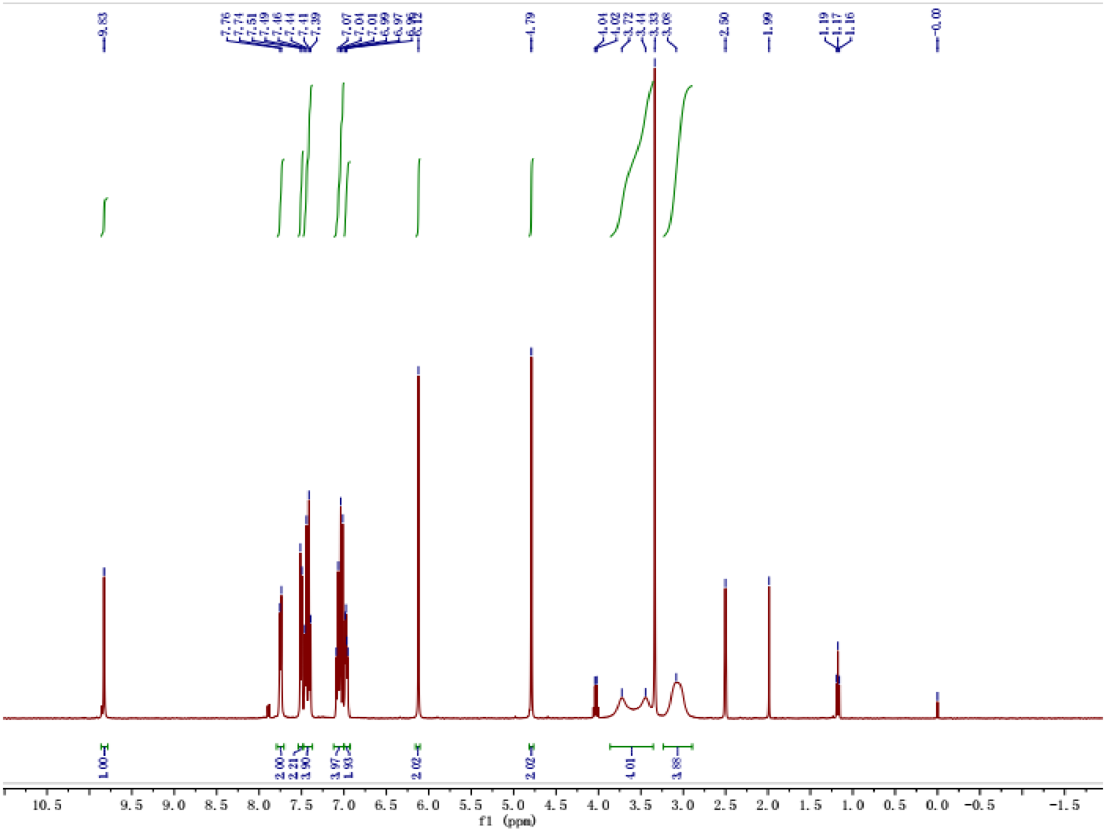

^{1}H-NMR spectrum of **B17**

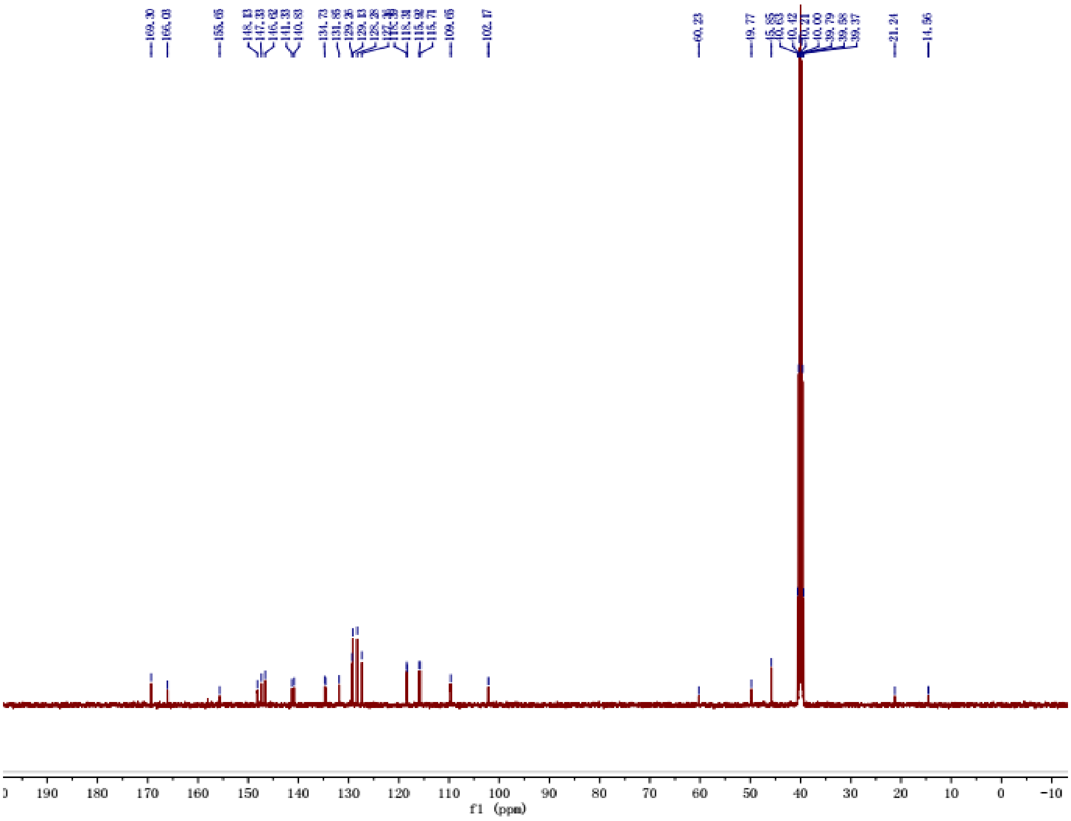

^{13}C-NMR spectrum of **B17**

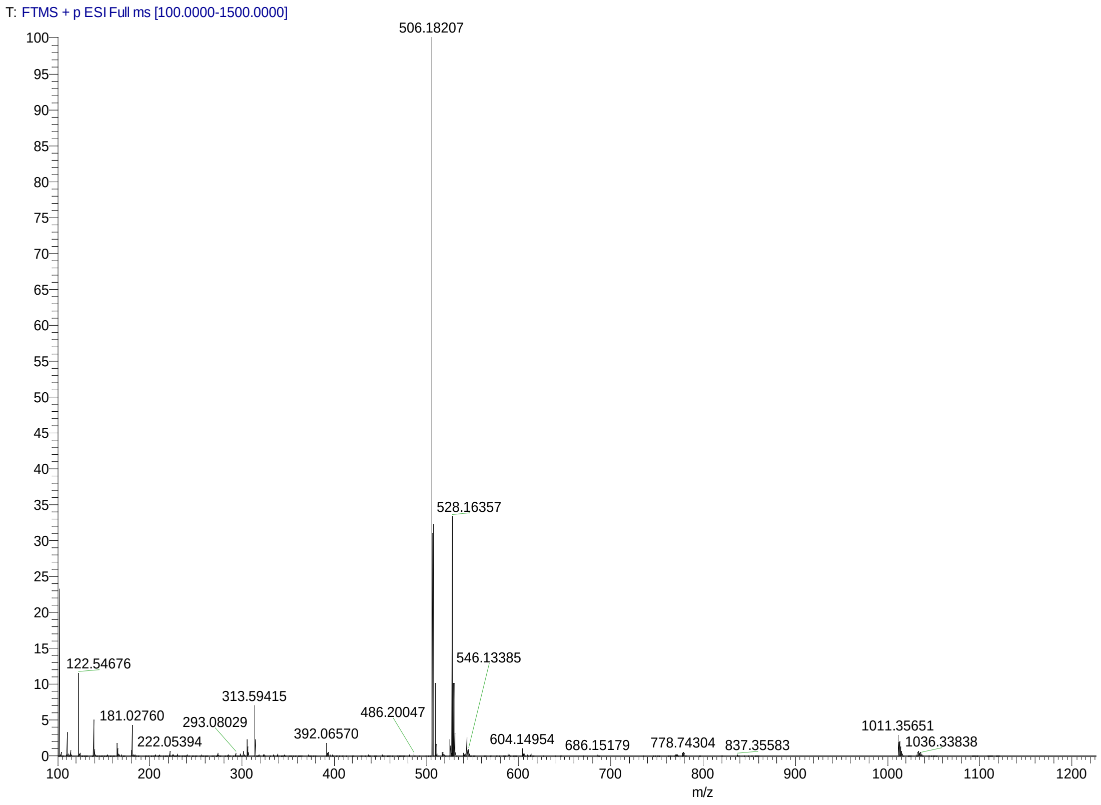

HRMS spectrum of **B1**8

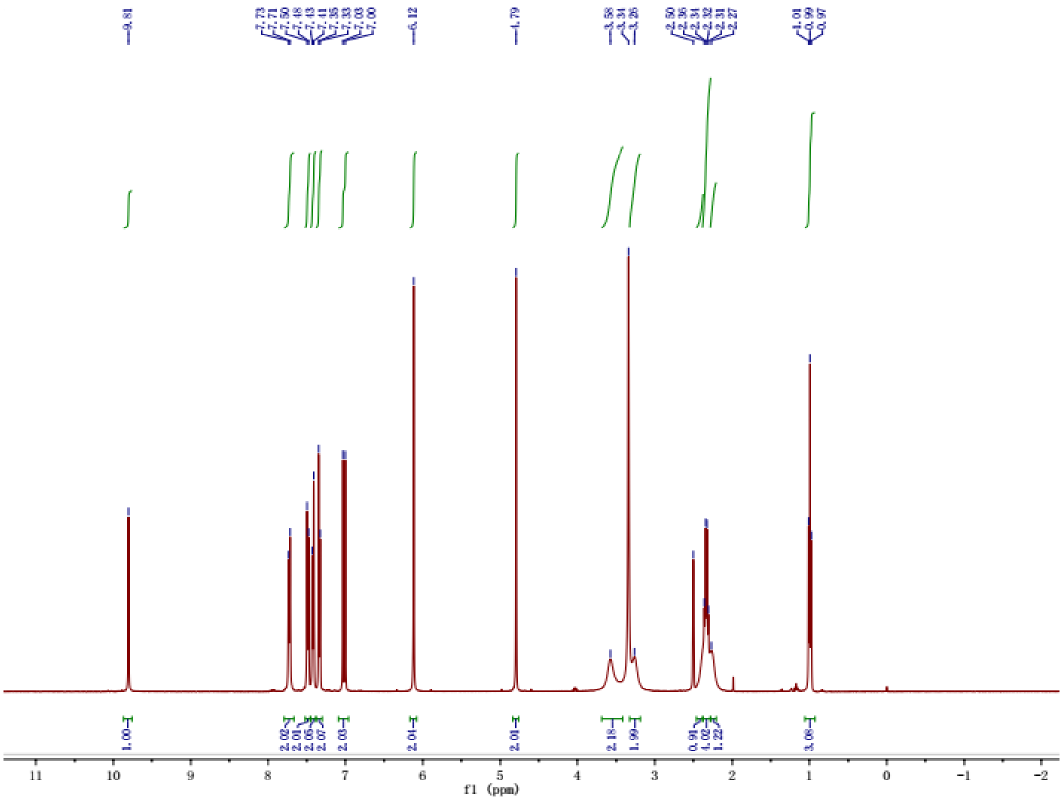

^{1}H-NMR spectrum of **B18**

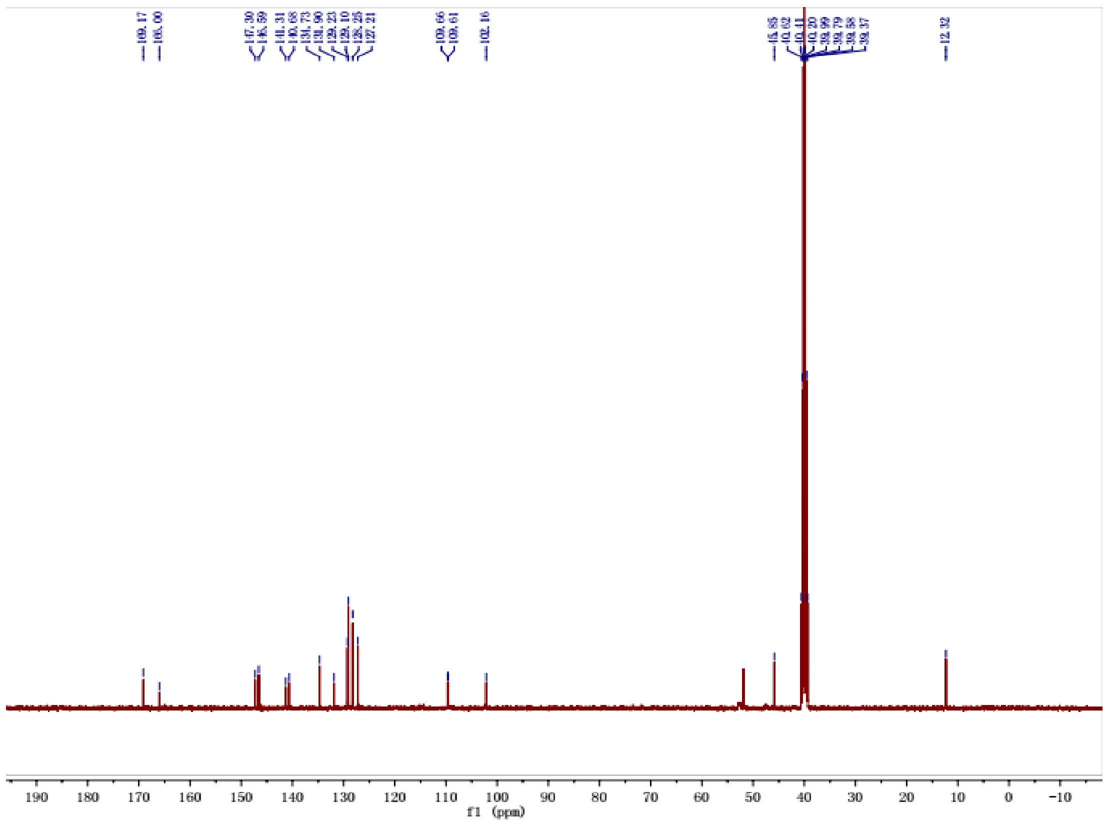

^{13}C-NMR spectrum of **B18**

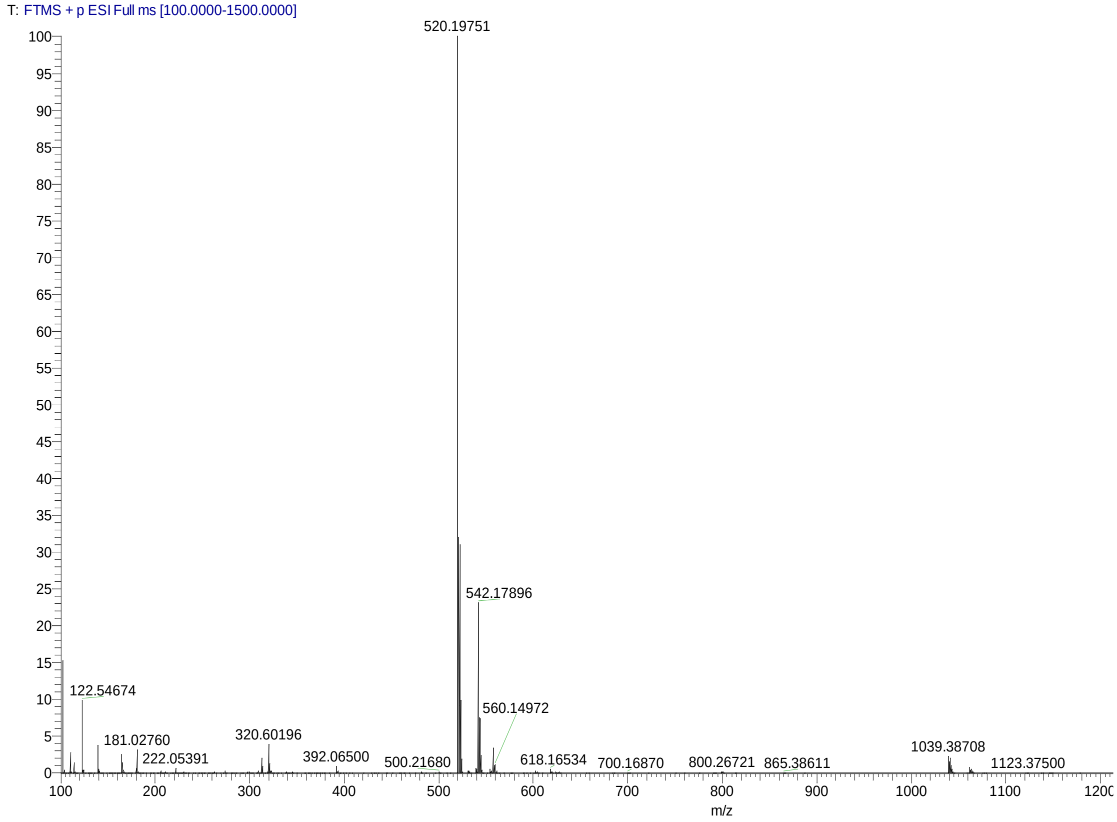

HRMS spectrum of **B19**

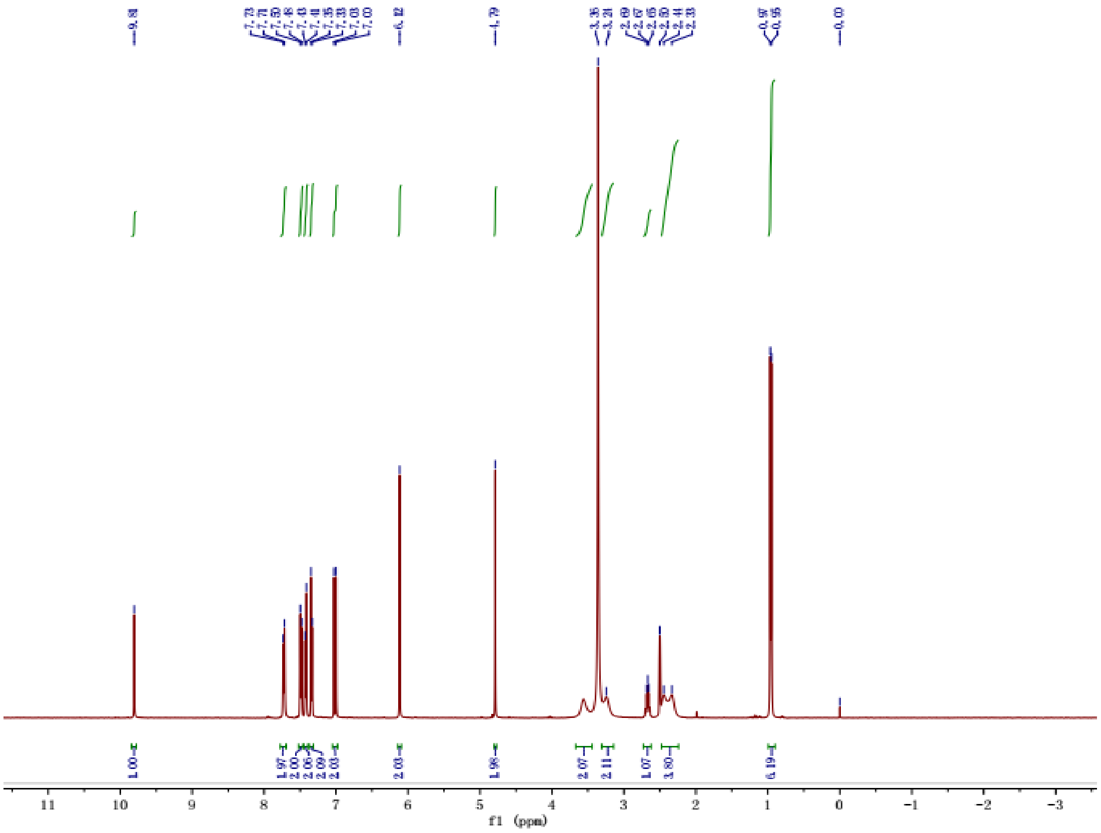

^{1}H-NMR spectrum of **B19**

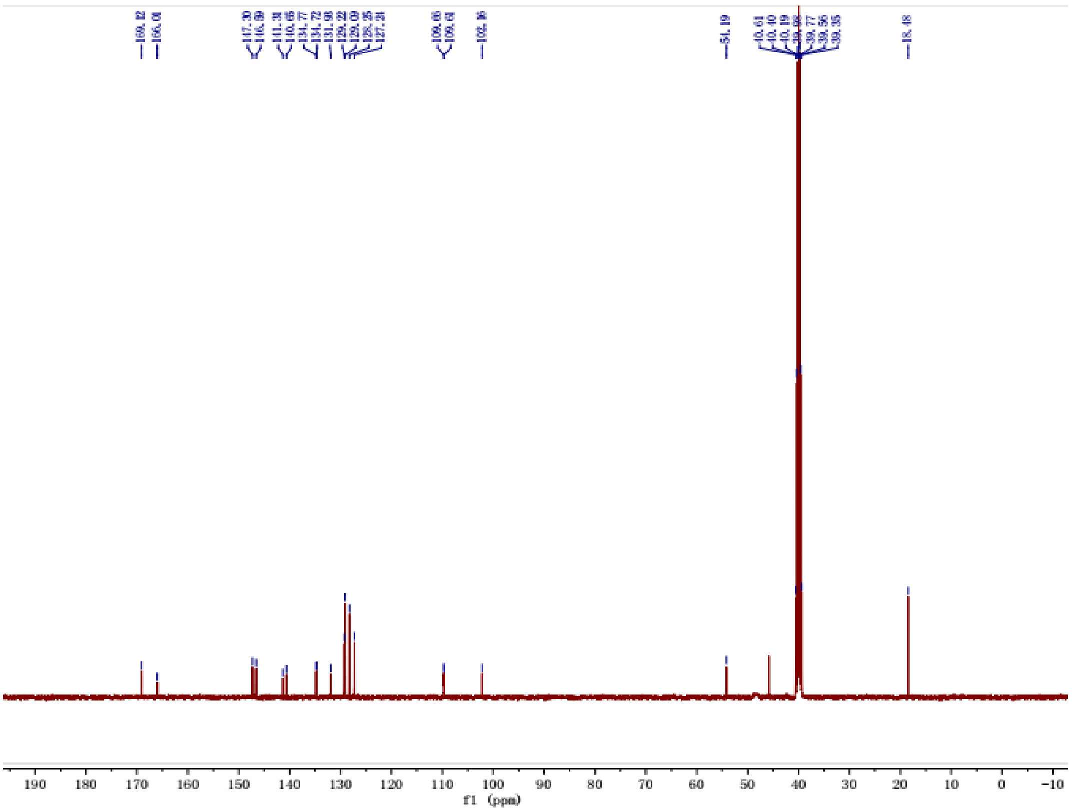

^{13}C-NMR spectrum of **B19**

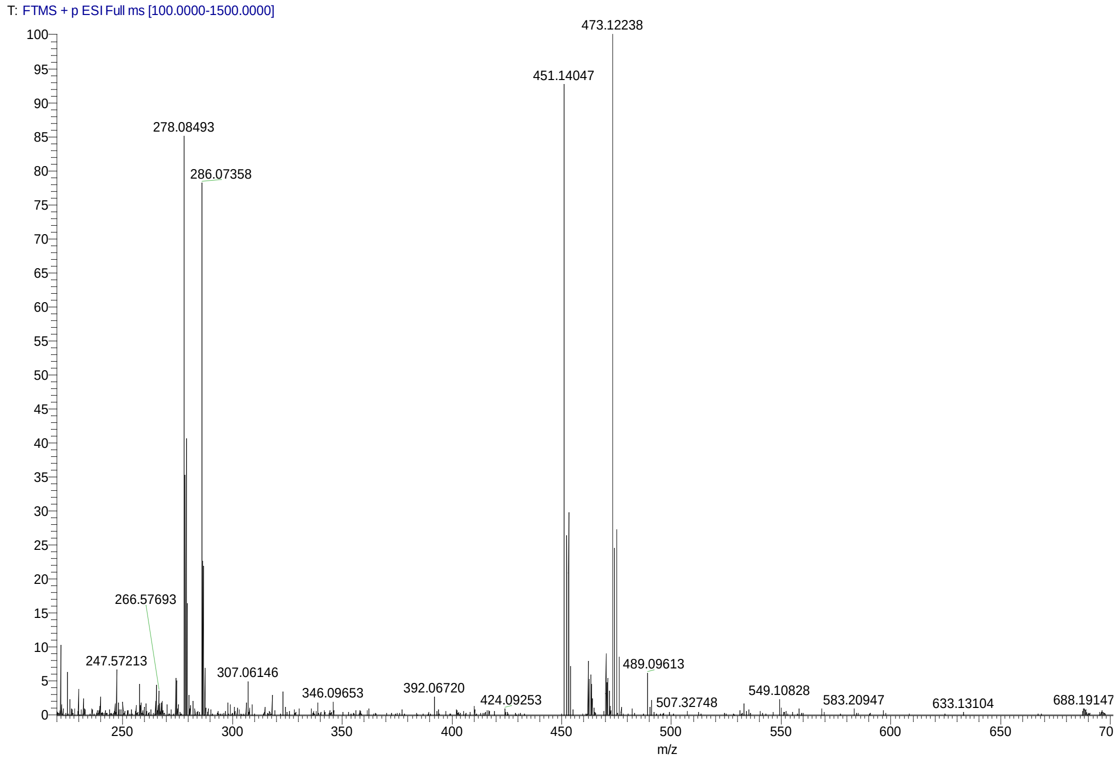

HRMS spectrum of **B20**

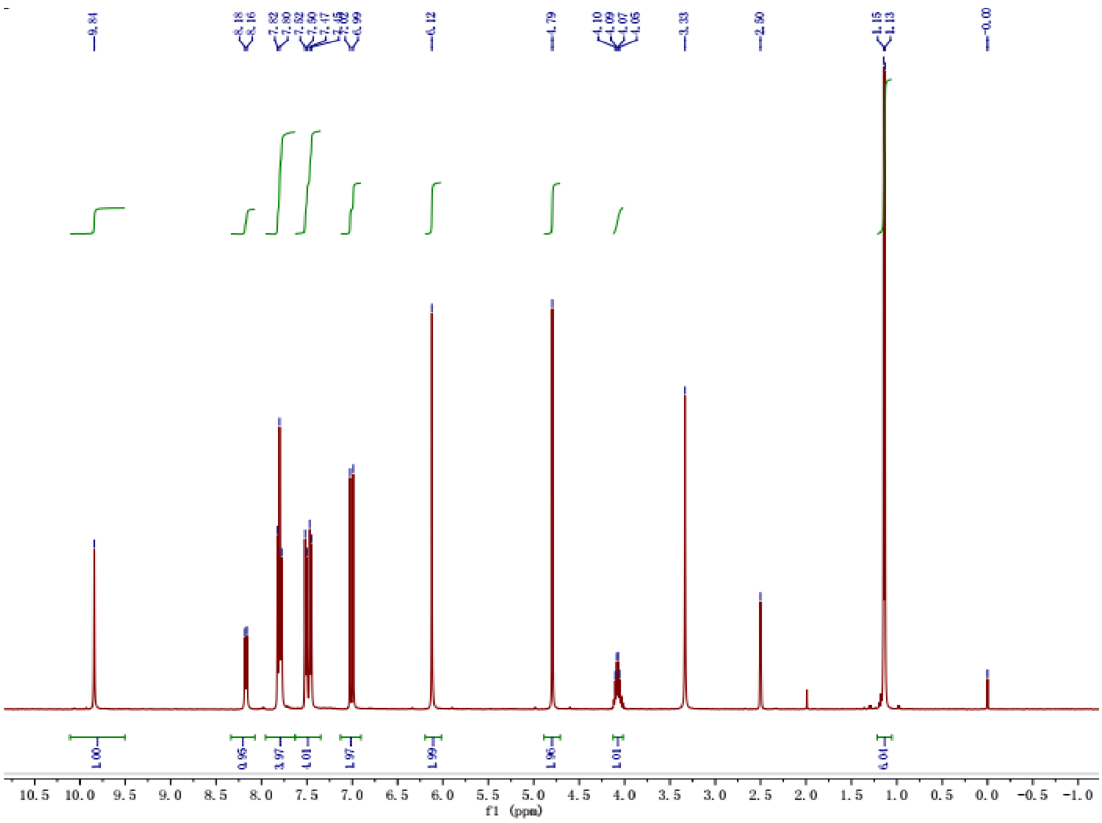

^{1}H-NMR spectrum of **B20**

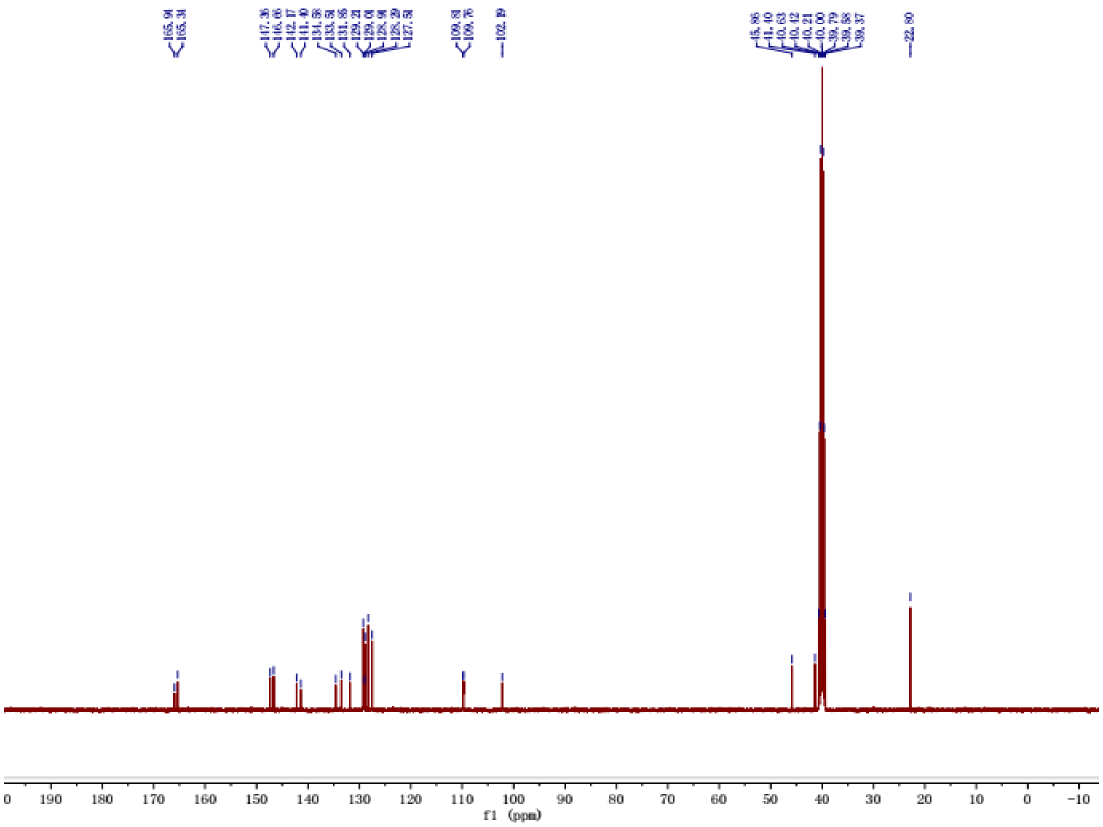

^{13}C-NMR spectrum of **B20**

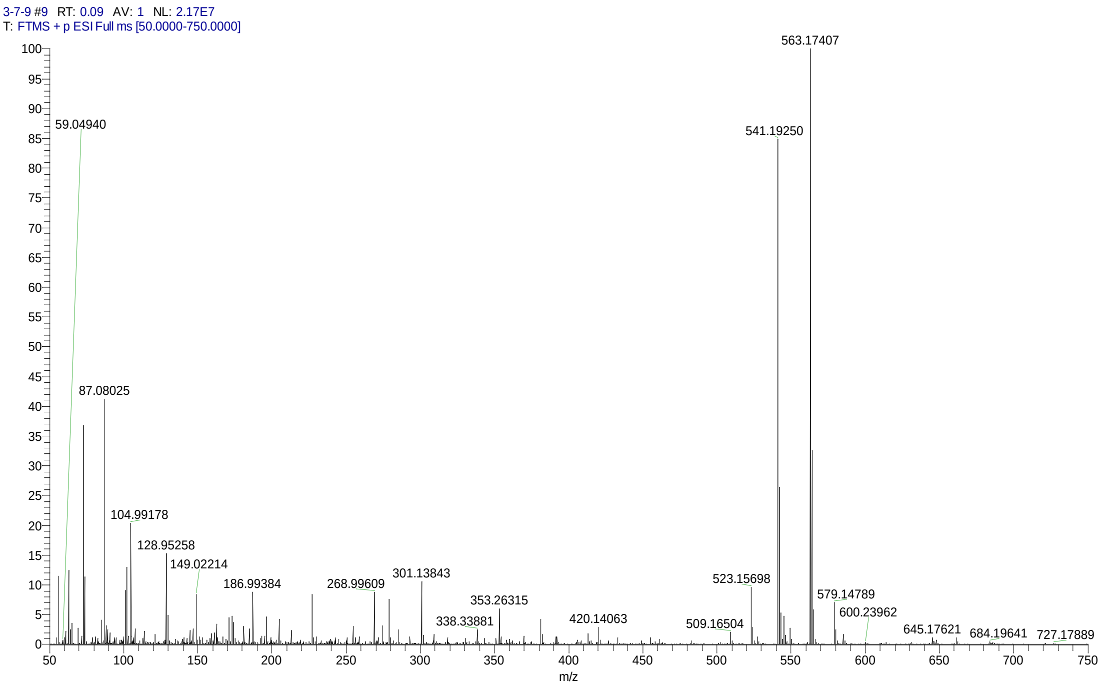

HRMS spectrum of **C1**

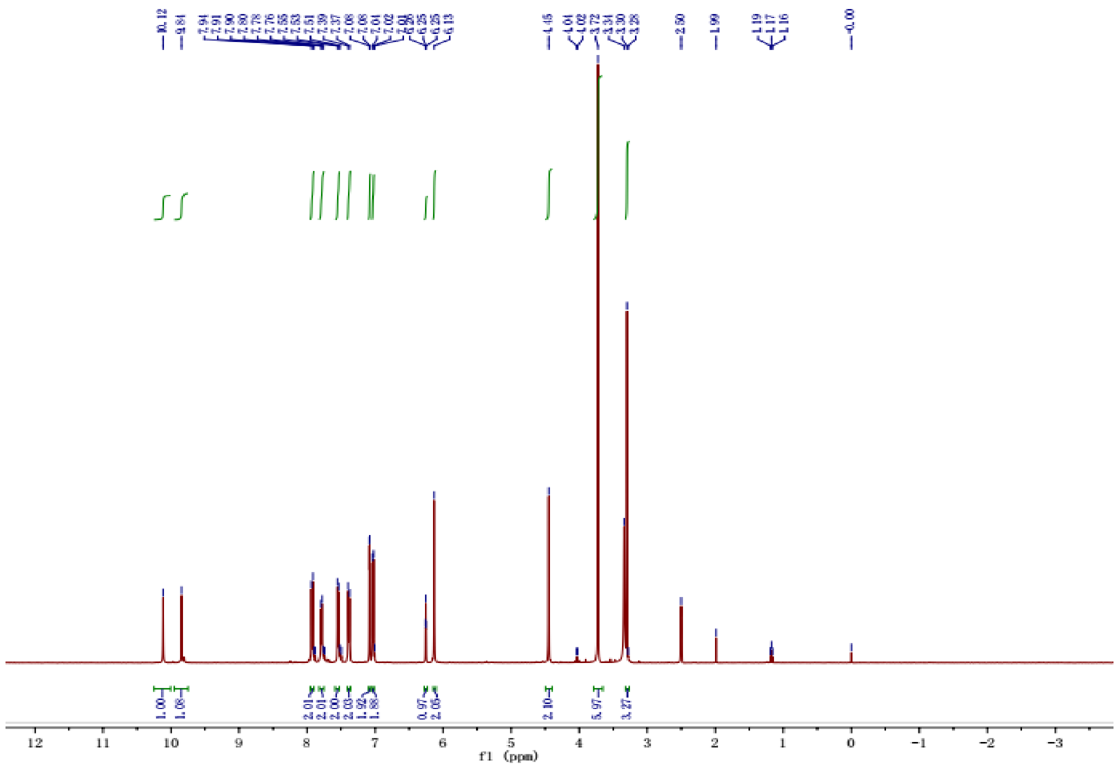

^{1}H-NMR spectrum of **C1**

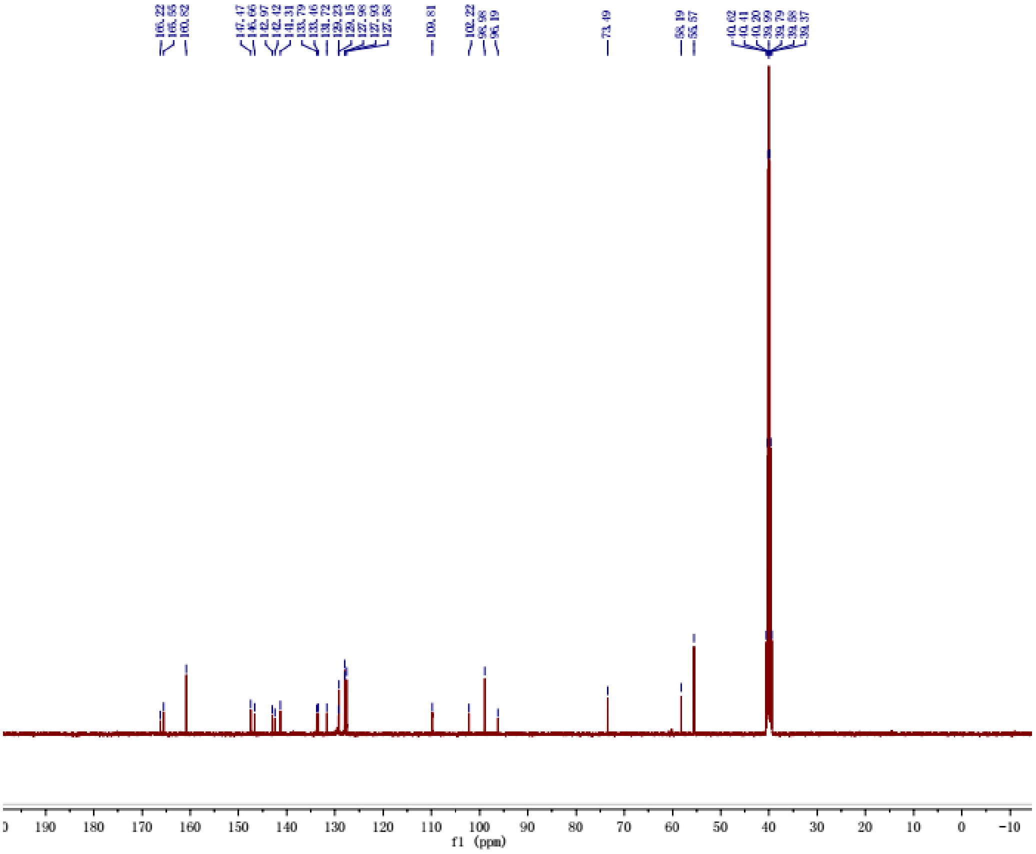

^{13}C-NMR spectrum of **C1**

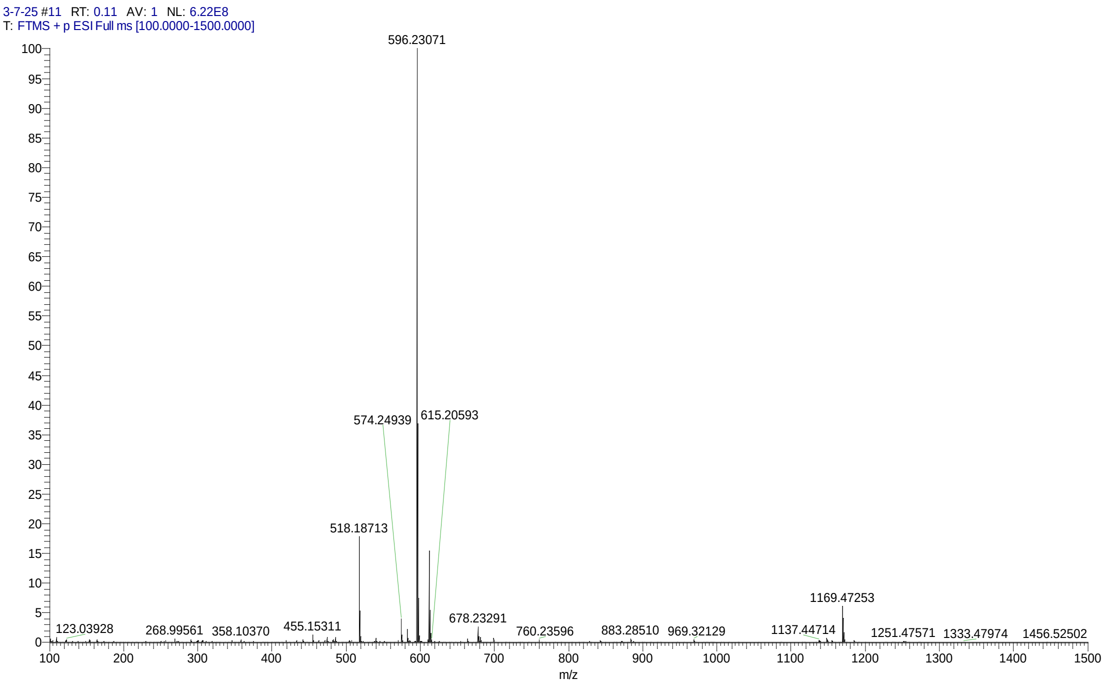

HRMS spectrum of **C2**

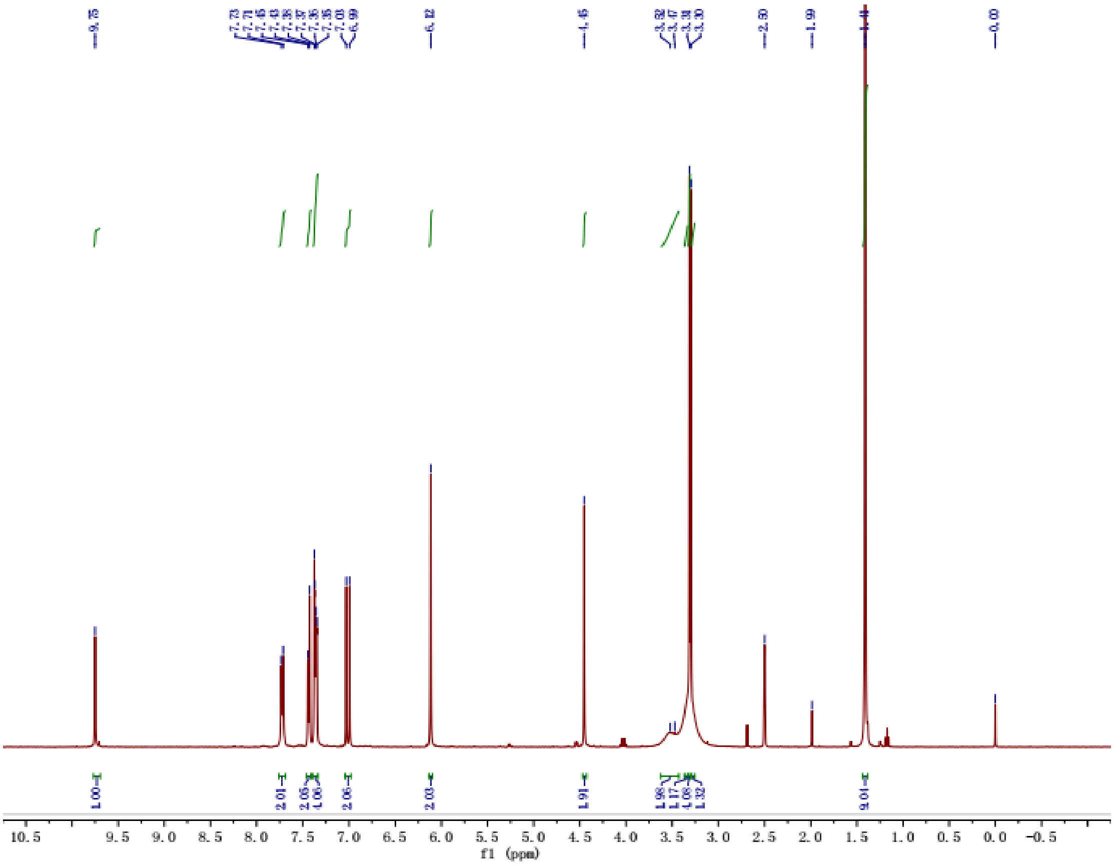

^{1}H-NMR spectrum of **C2**

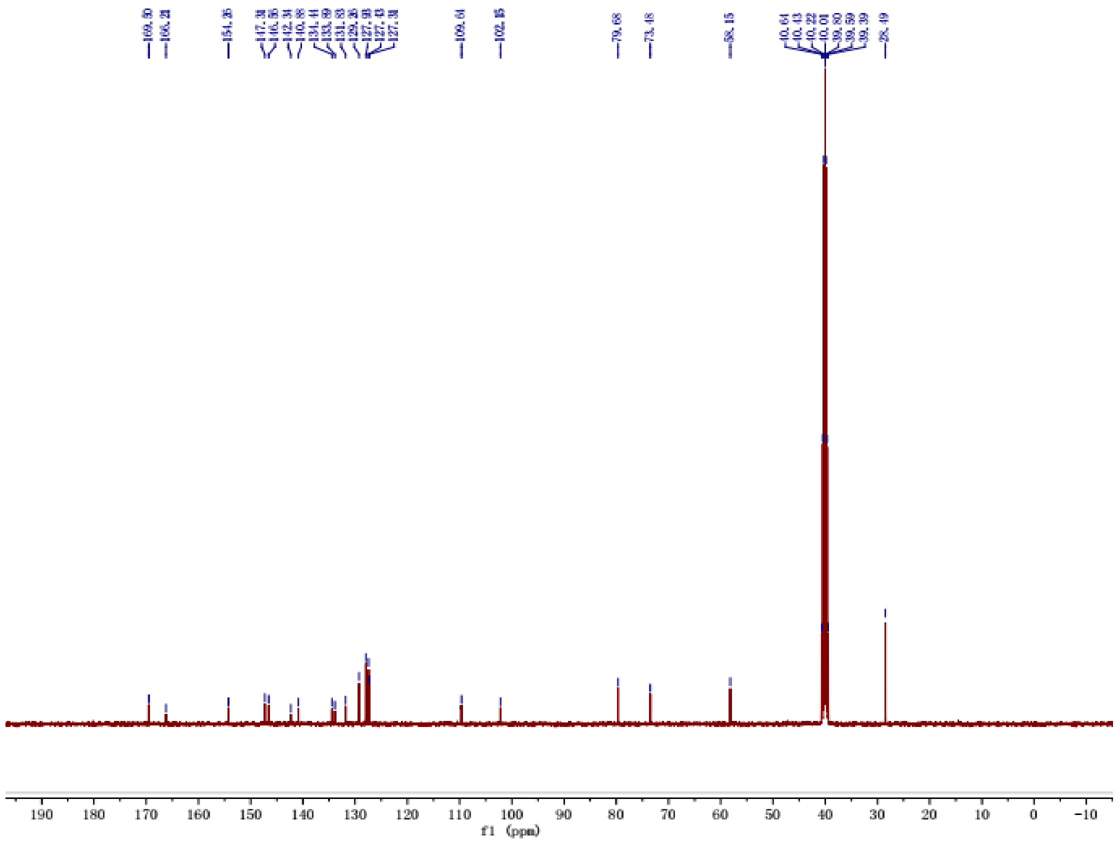

^{13}C-NMR spectrum of **C2**

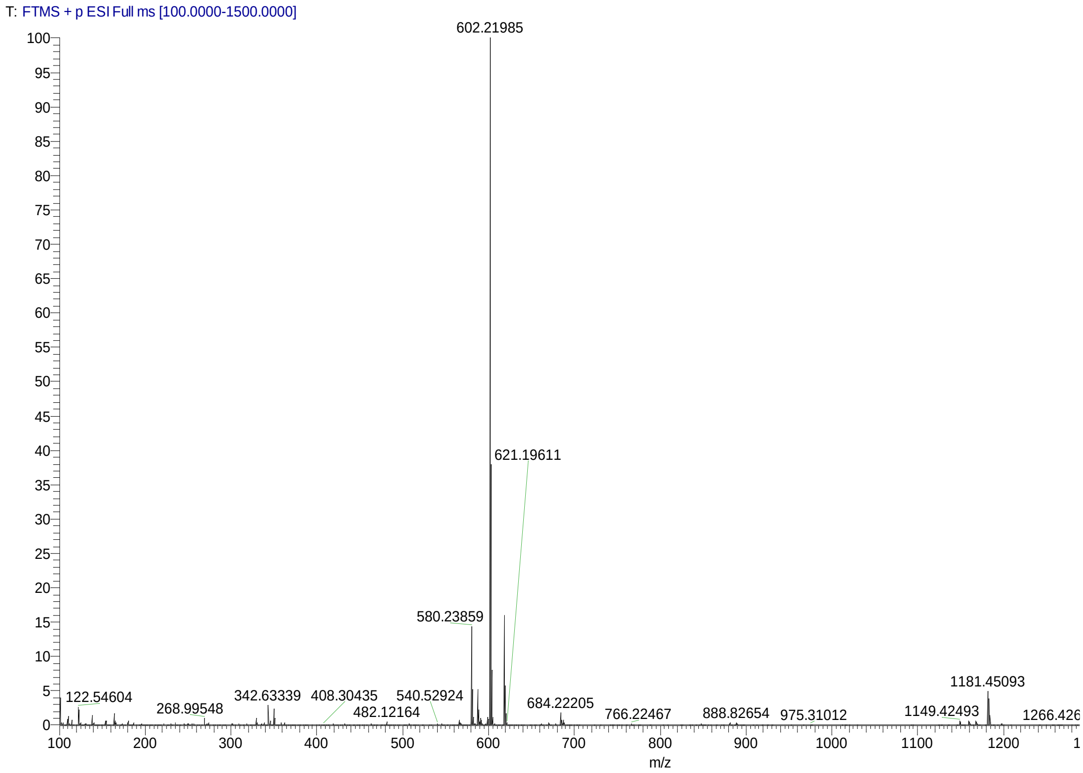

HRMS spectrum of **C3**

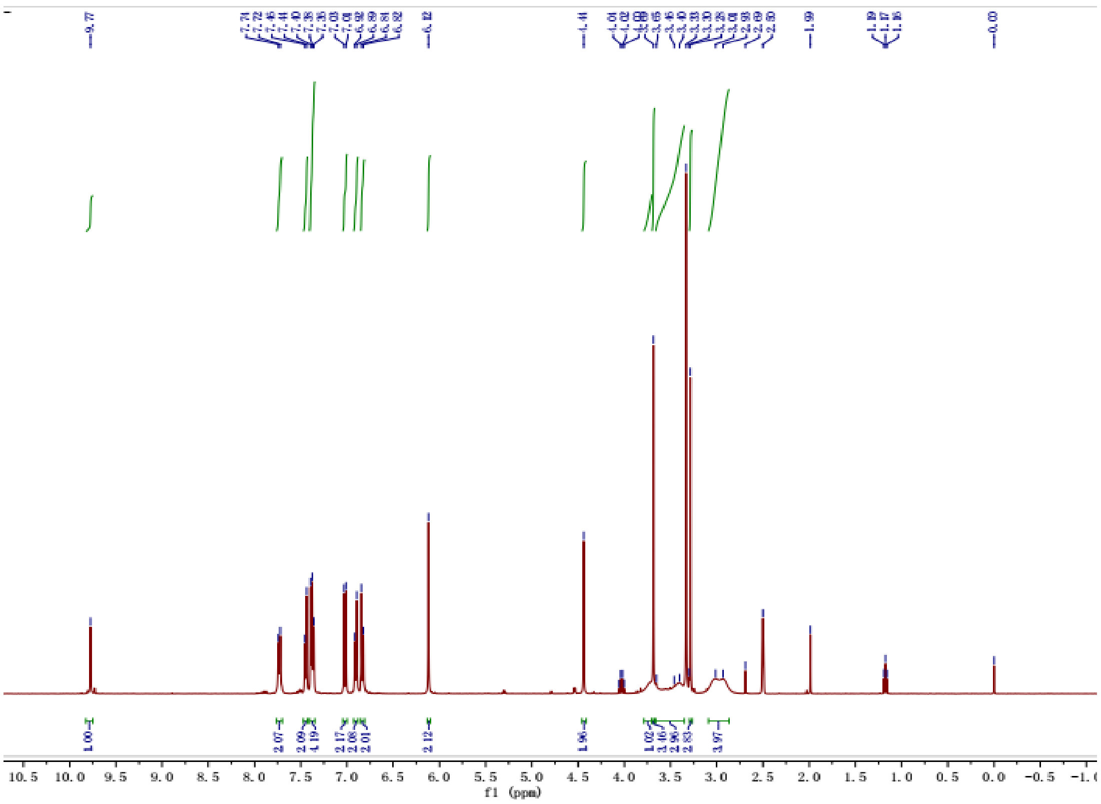

^{1}H-NMR spectrum of **C3**

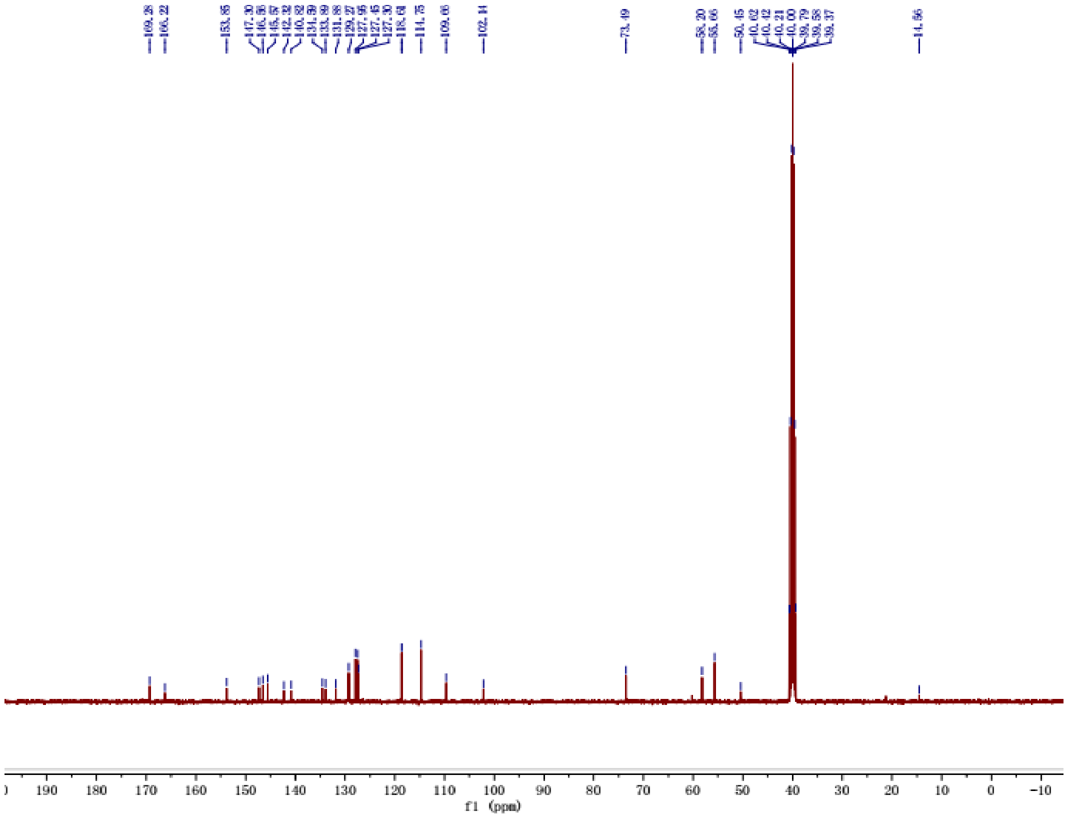

^{13}C-NMR spectrum of **C3**

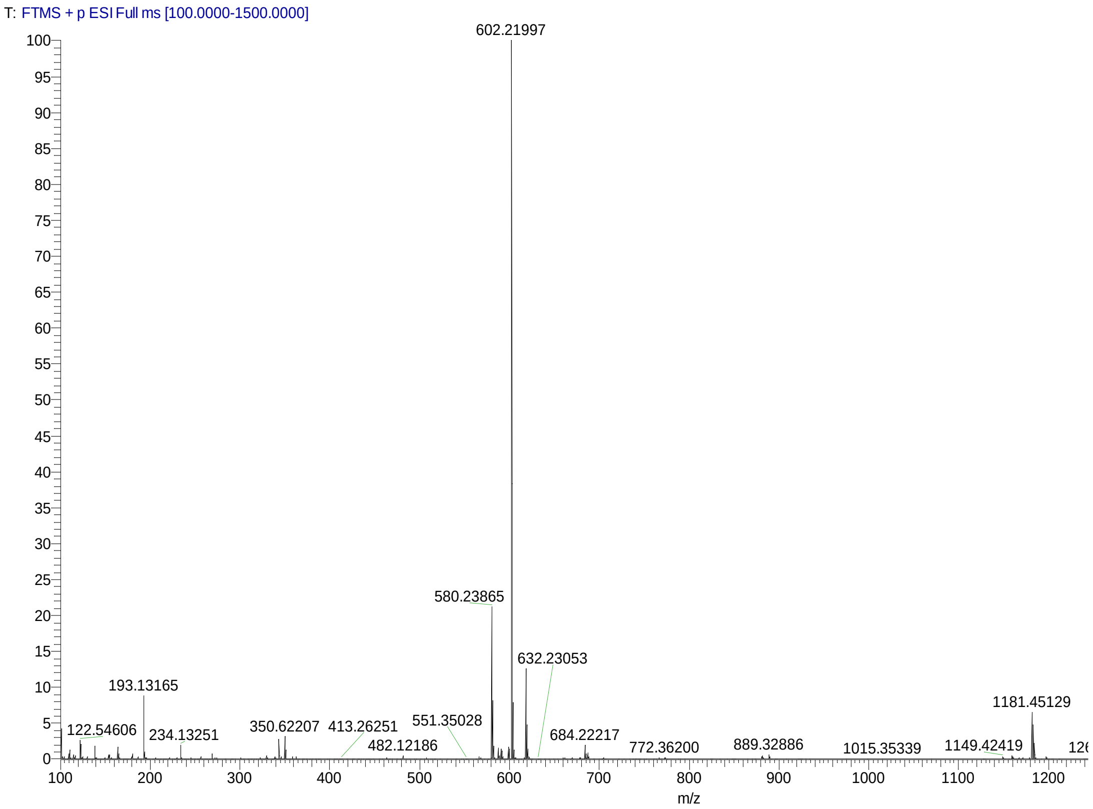

HRMS spectrum of **C4**

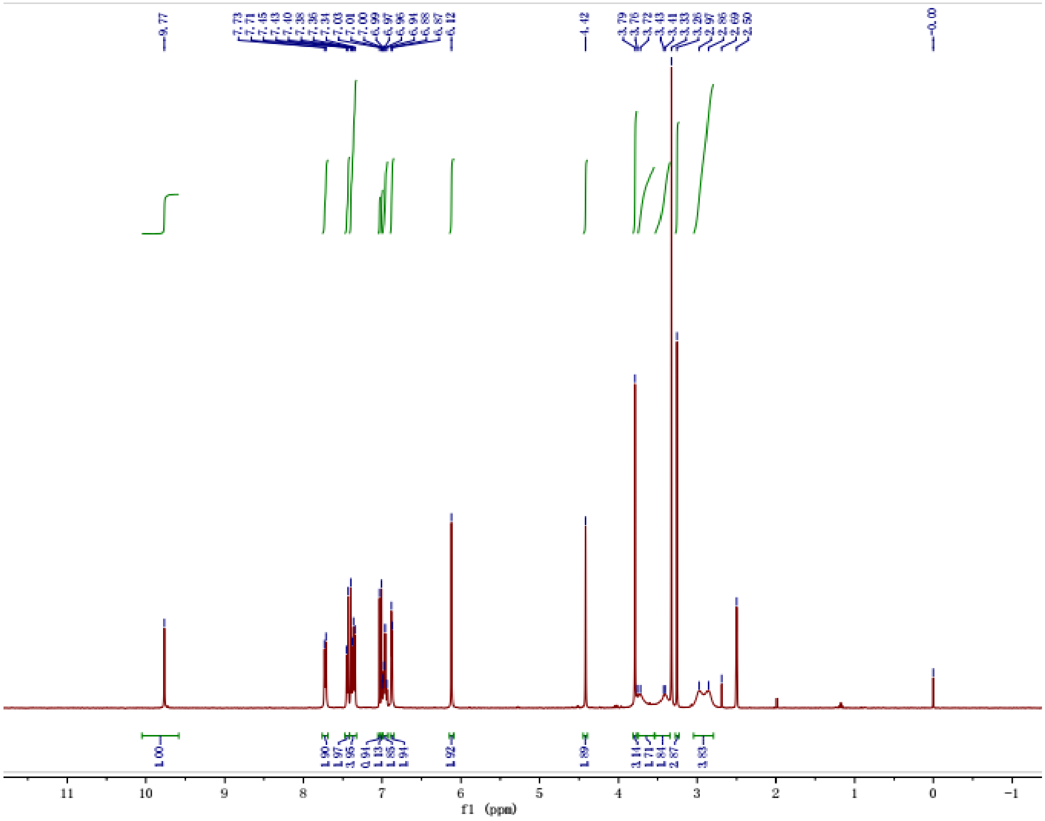

1H-NMR spectrum of **C4**

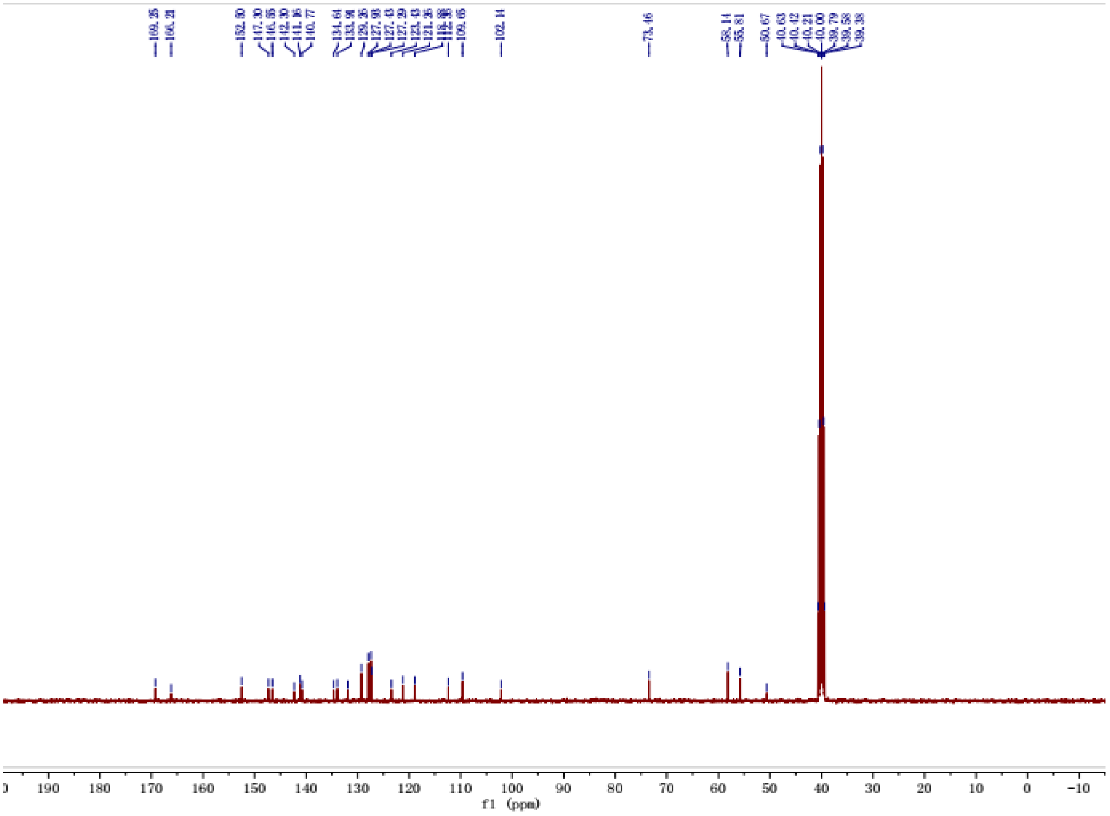

^{13}C-NMR spectrum of **C4**

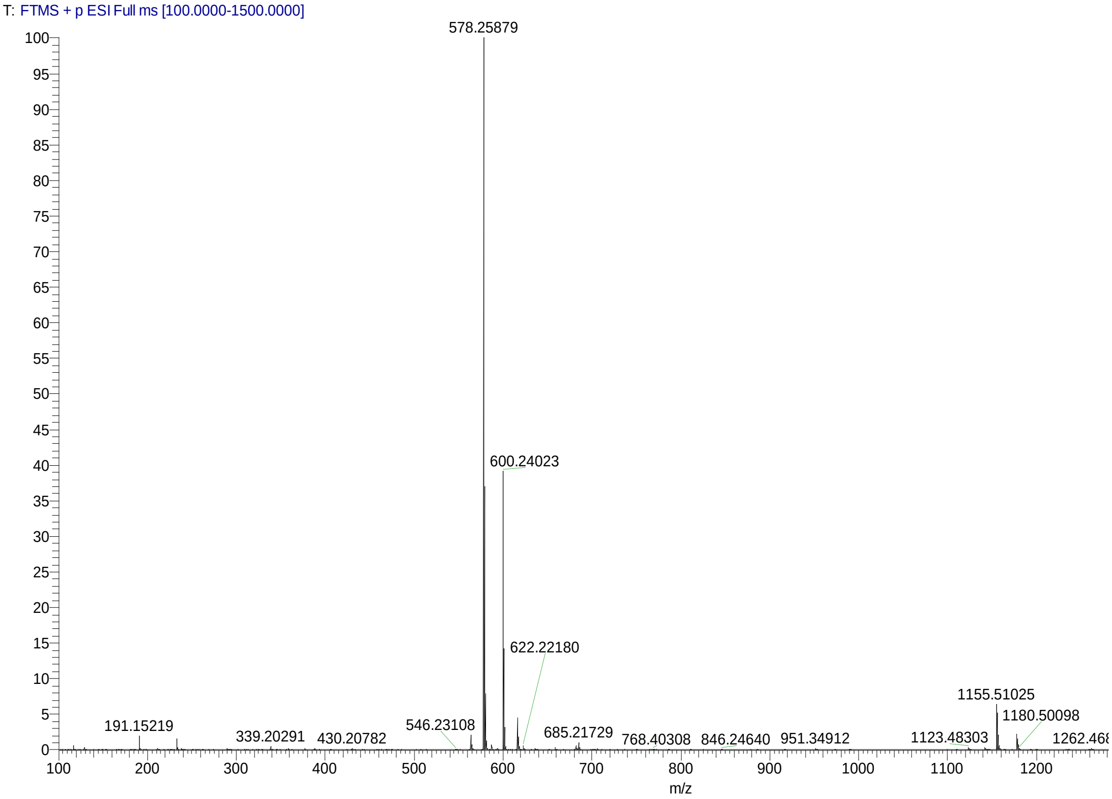

HRMS spectrum of **C5**

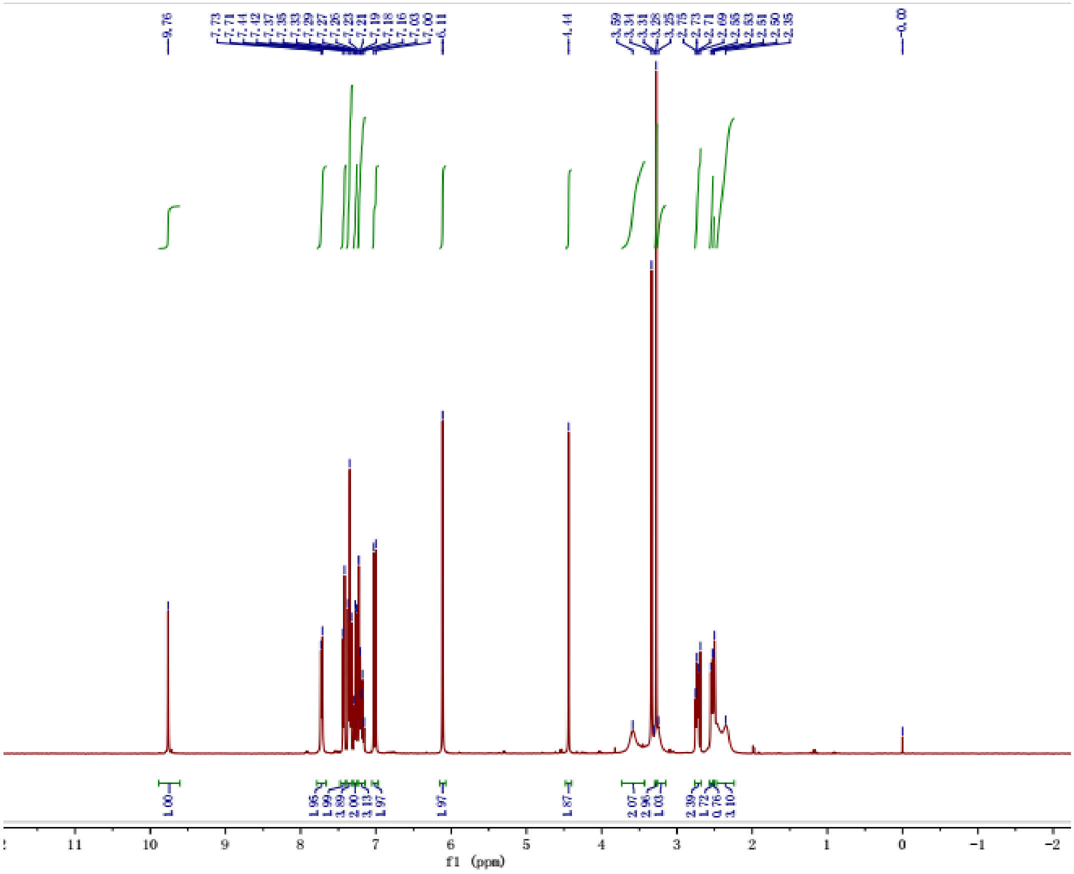

^{1}H-NMR spectrum of **C5**

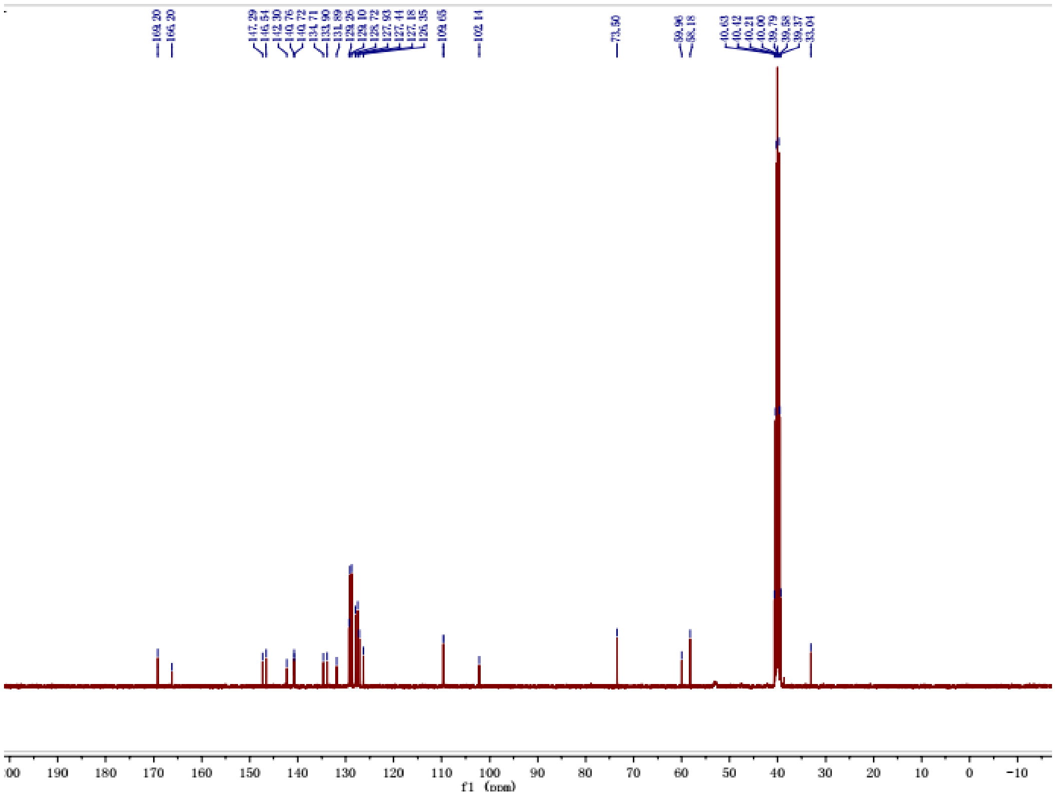

^{13}C-NMR spectrum of **C5**

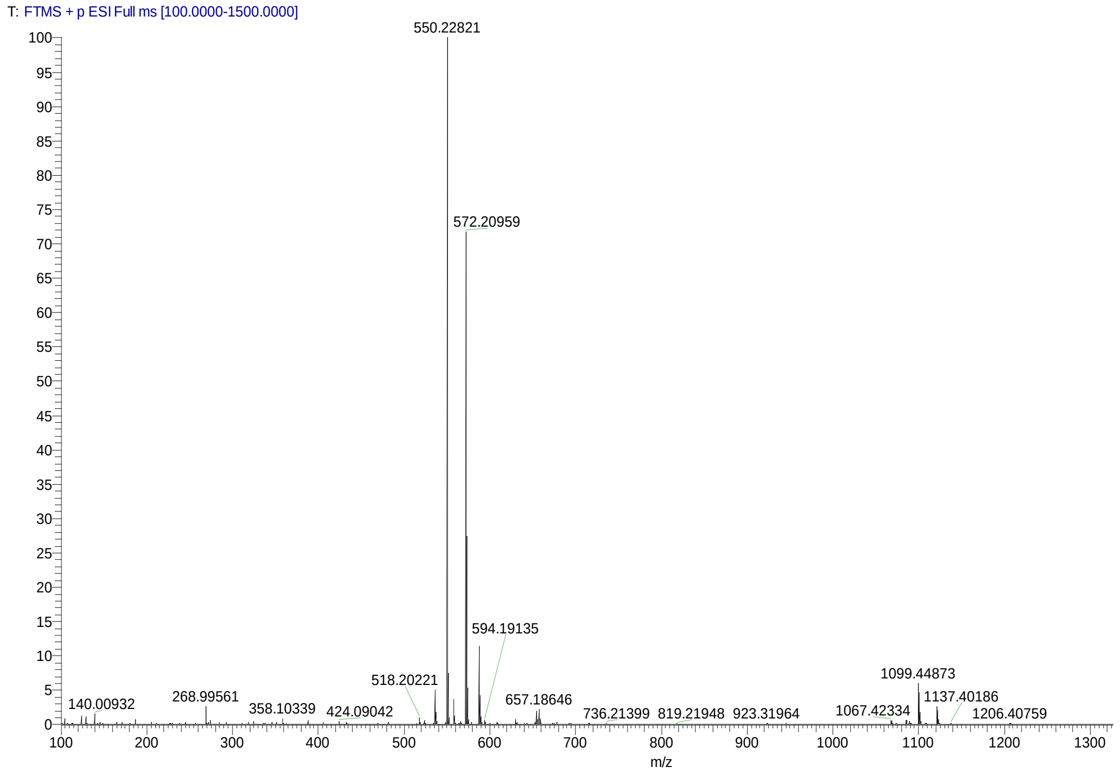

HRMS spectrum of **C6**

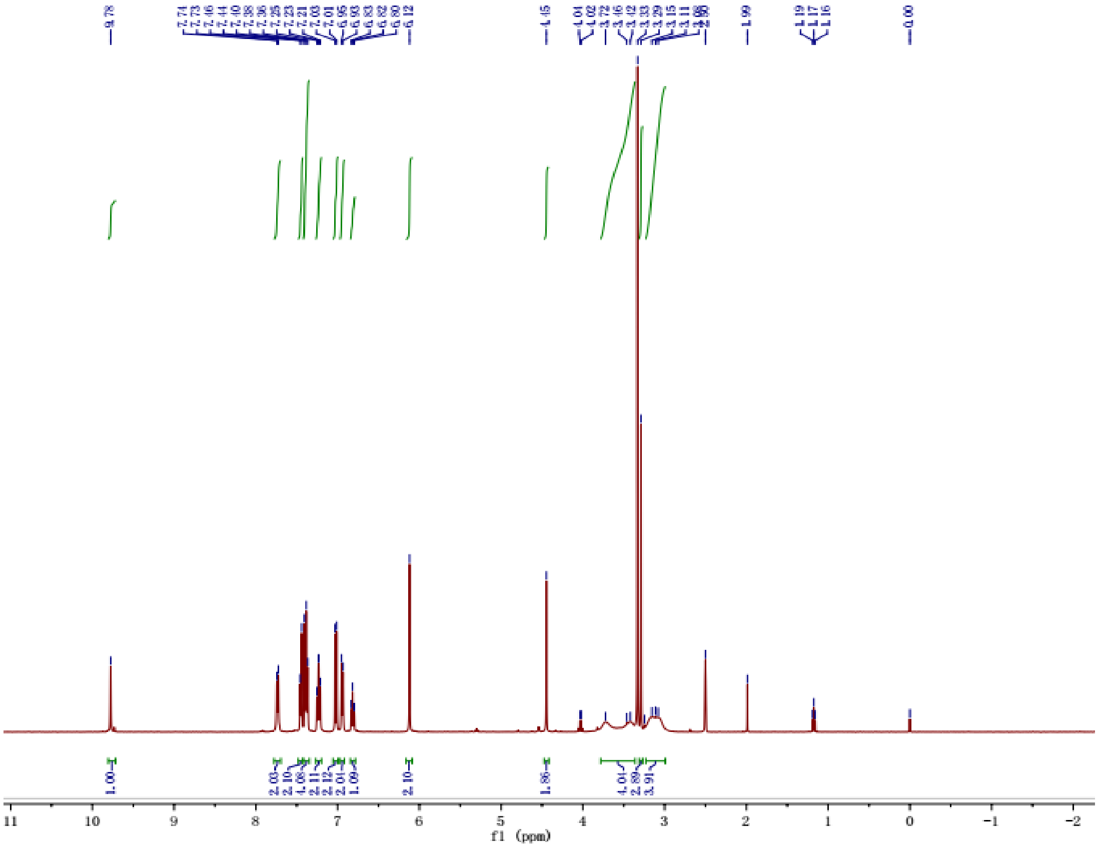

^{1}H-NMR spectrum of **C6**

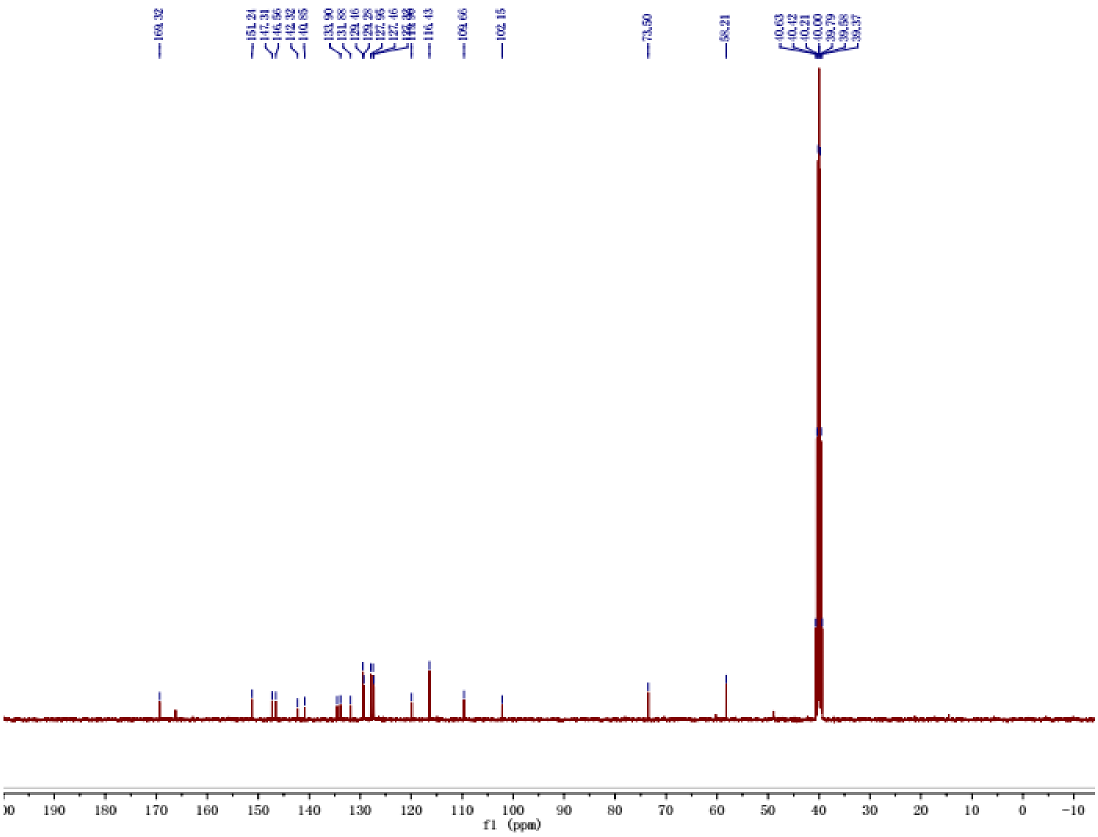

^{13}C-NMR spectrum of **C6**

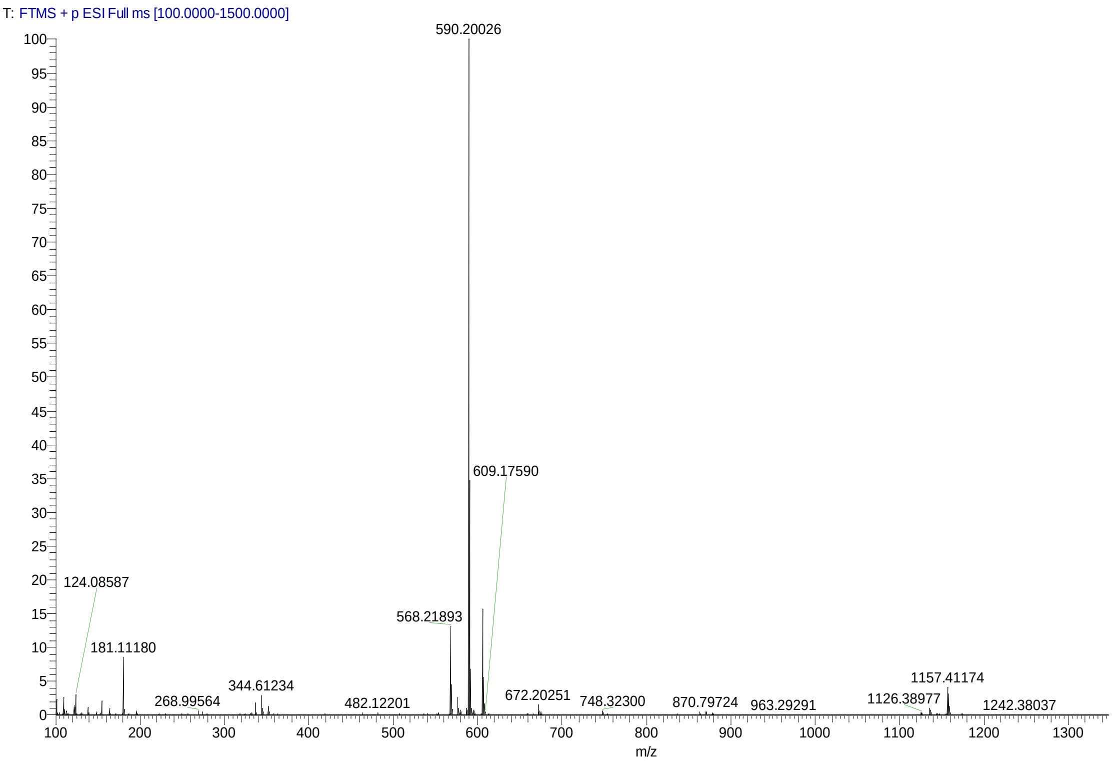

HRMS spectrum of **C7**

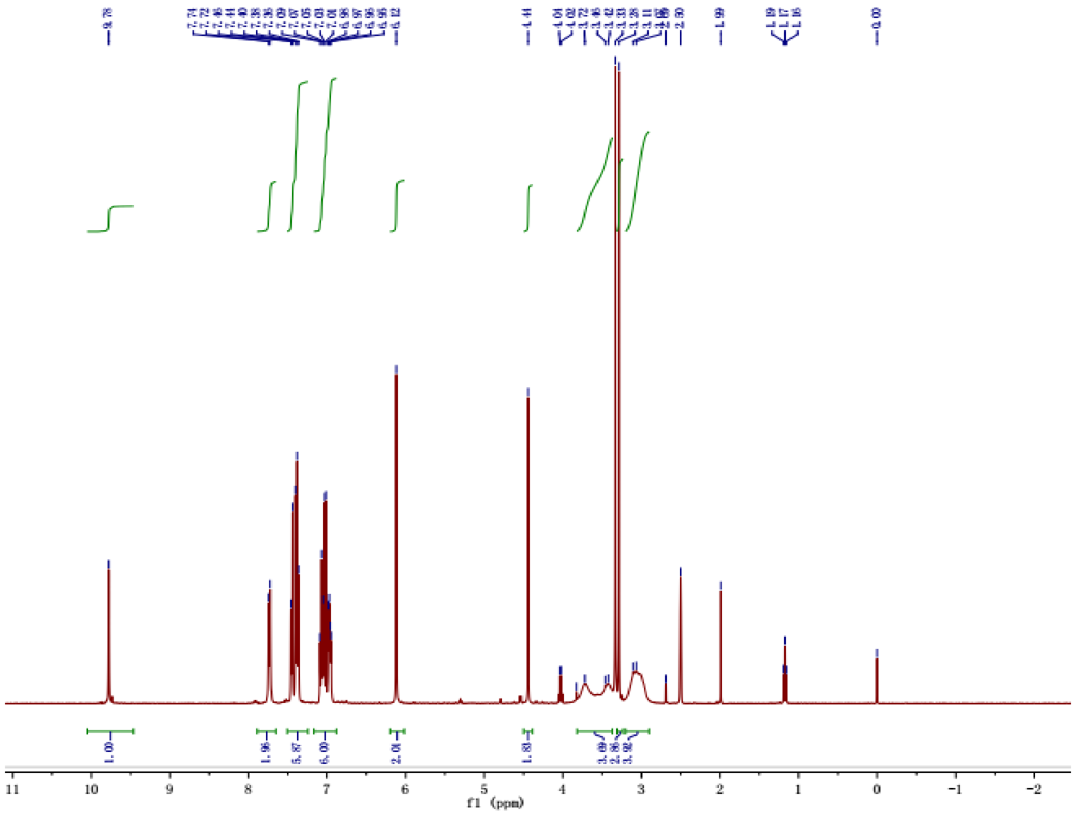

^{1}H-NMR spectrum of **C7**

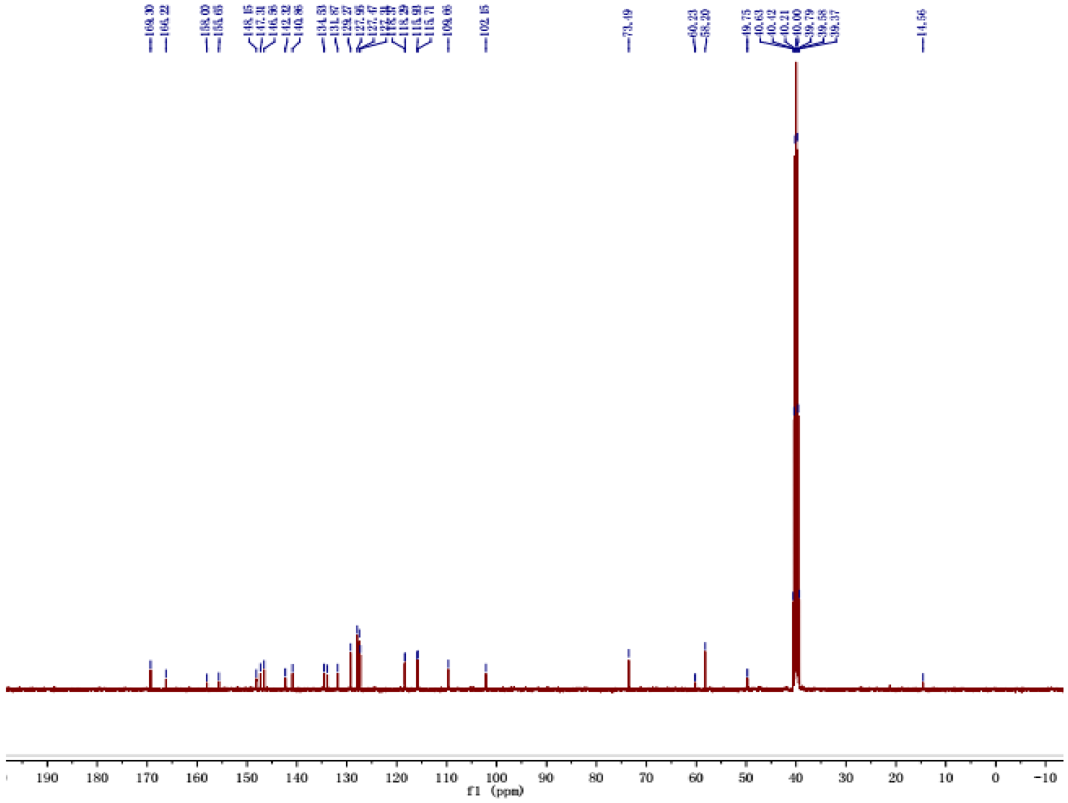

^{13}C-NMR spectrum of **C7**

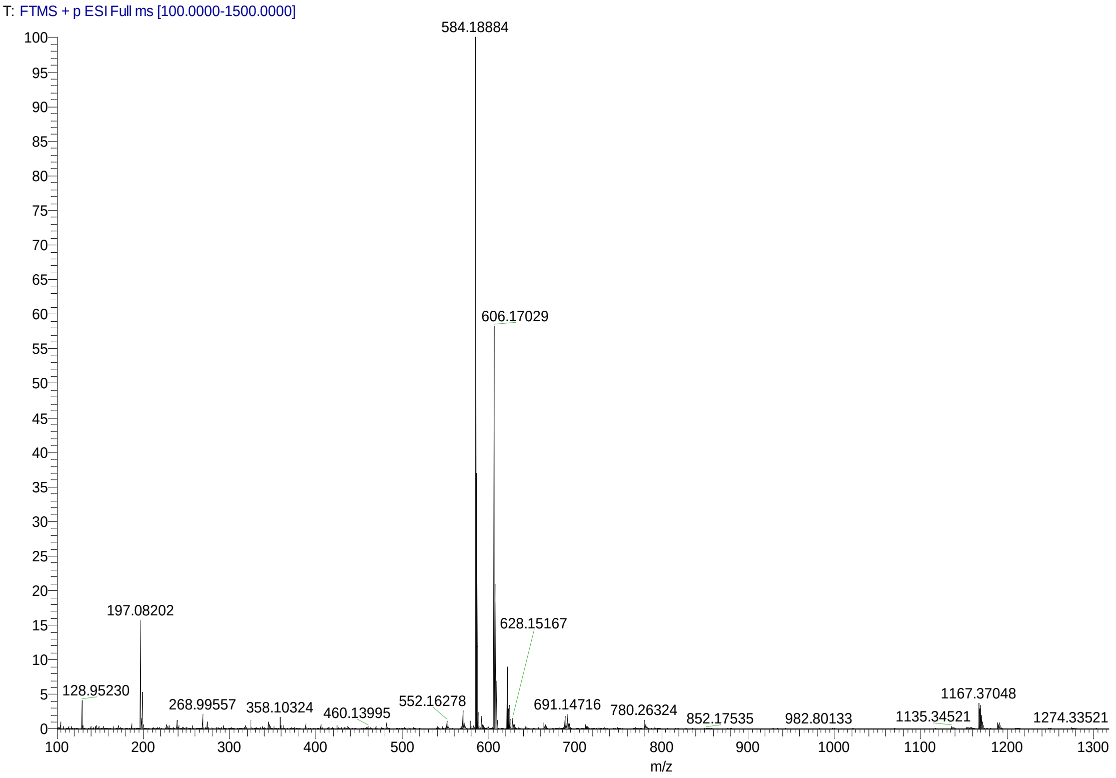

HRMS spectrum of **C8**

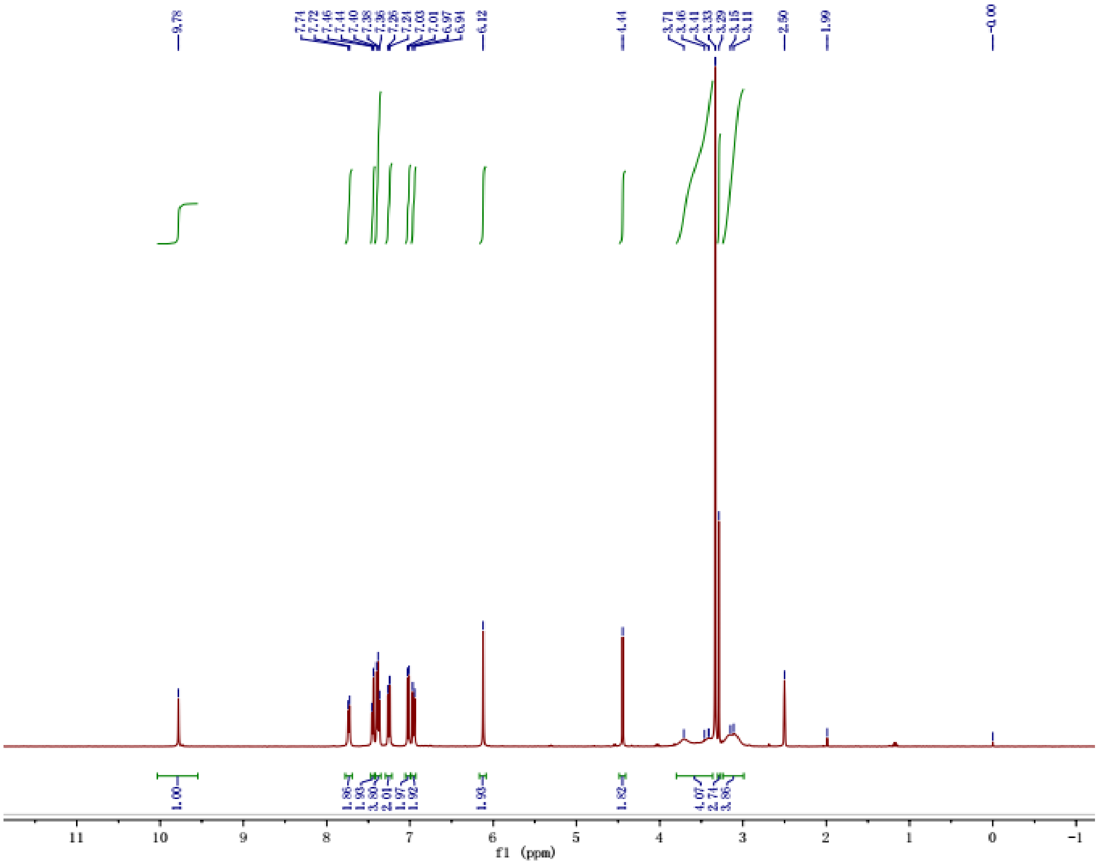

^{1}H-NMR spectrum of **C8**

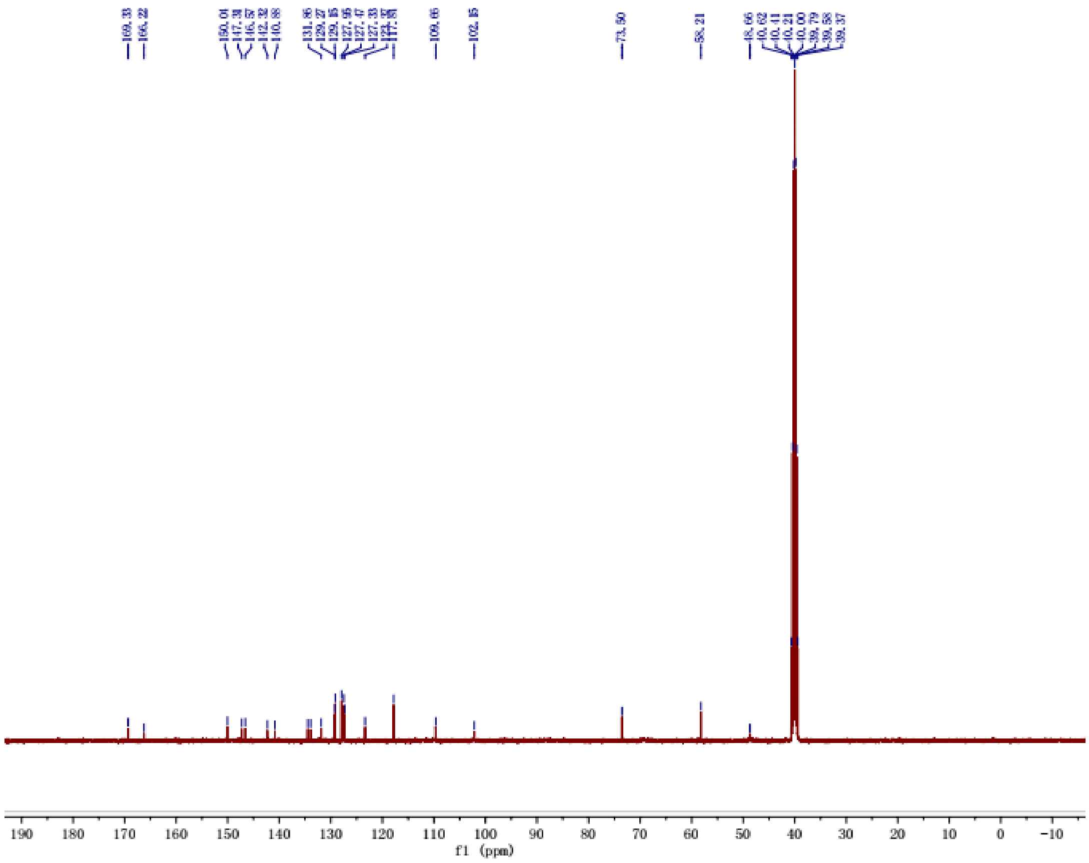

^{13}C-NMR spectrum of **C8**

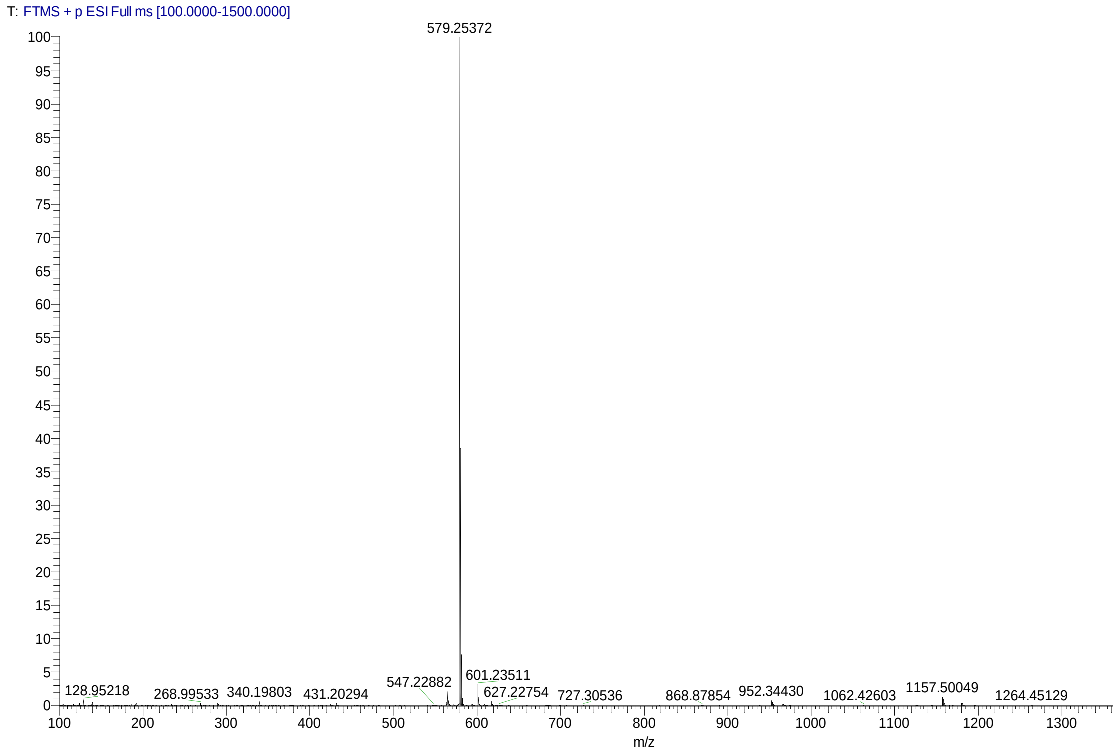

HRMS spectrum of **C9**

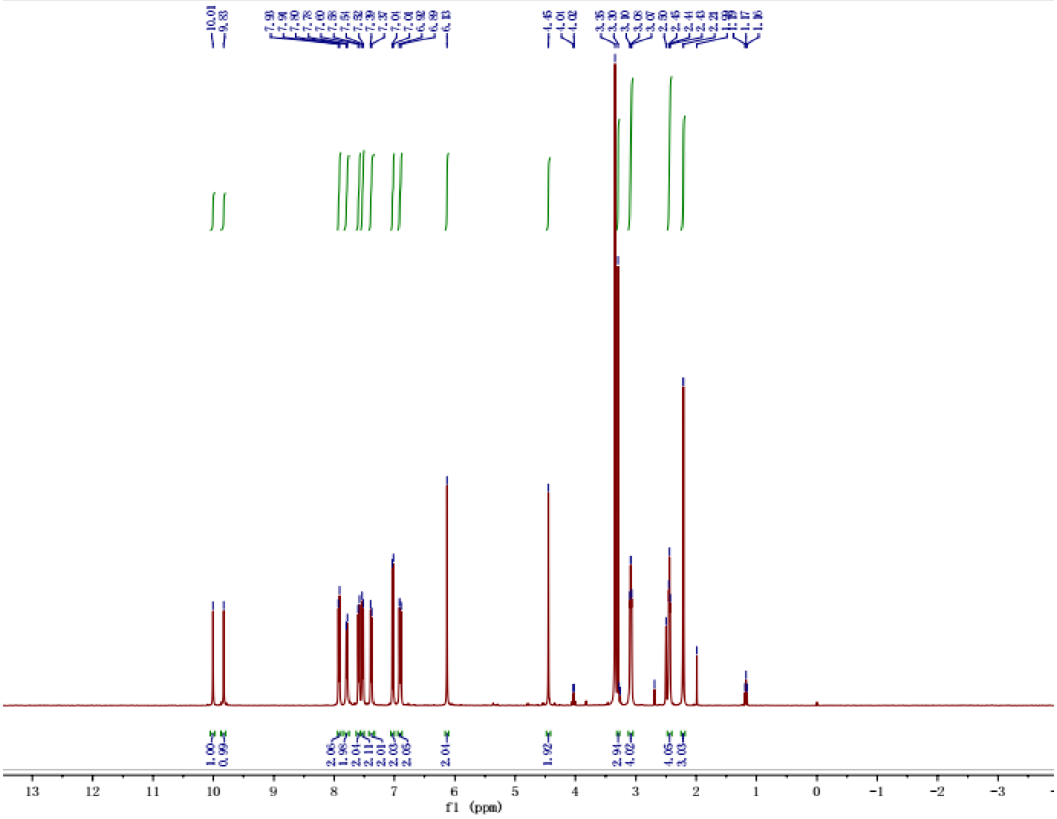

^{1}H-NMR spectrum of **C9**

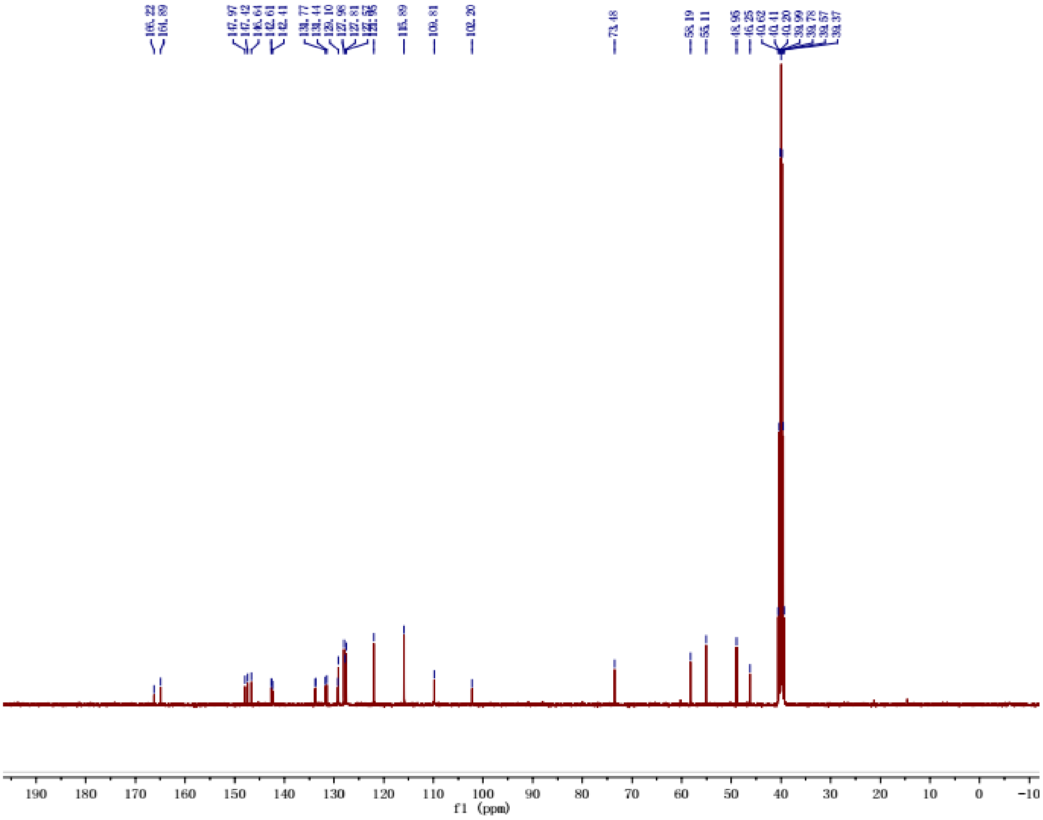

^{13}C-NMR spectrum of **C9**

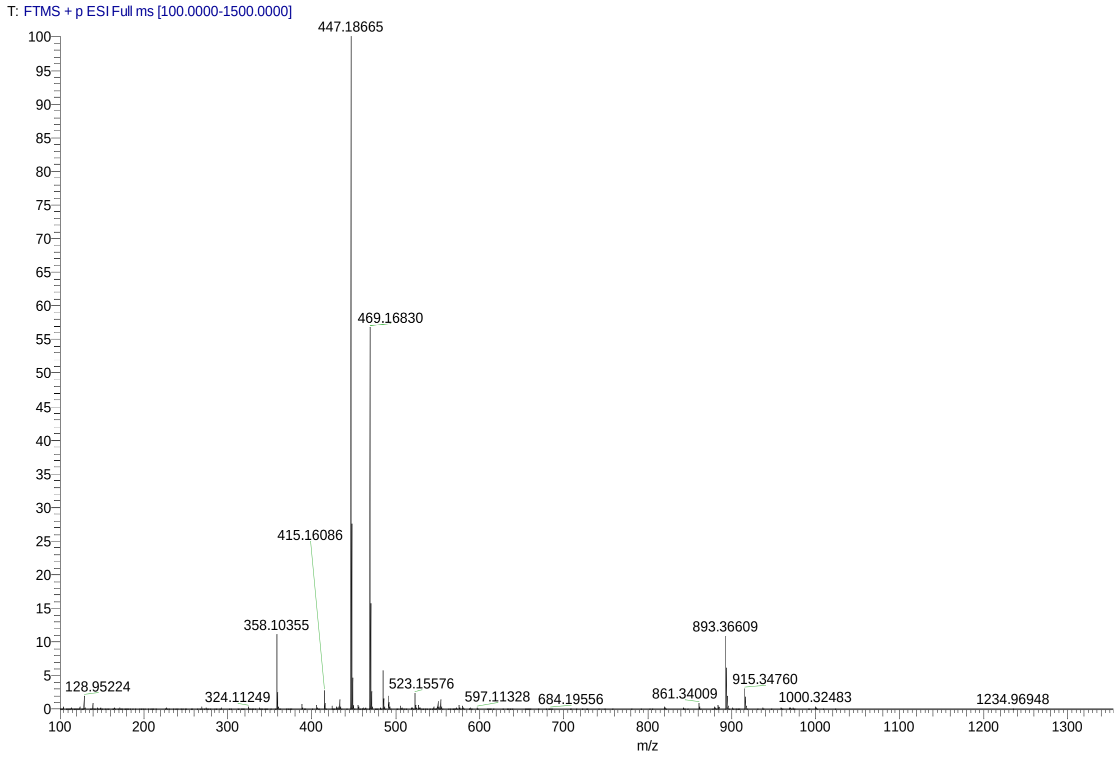

HRMS spectrum of **C10**

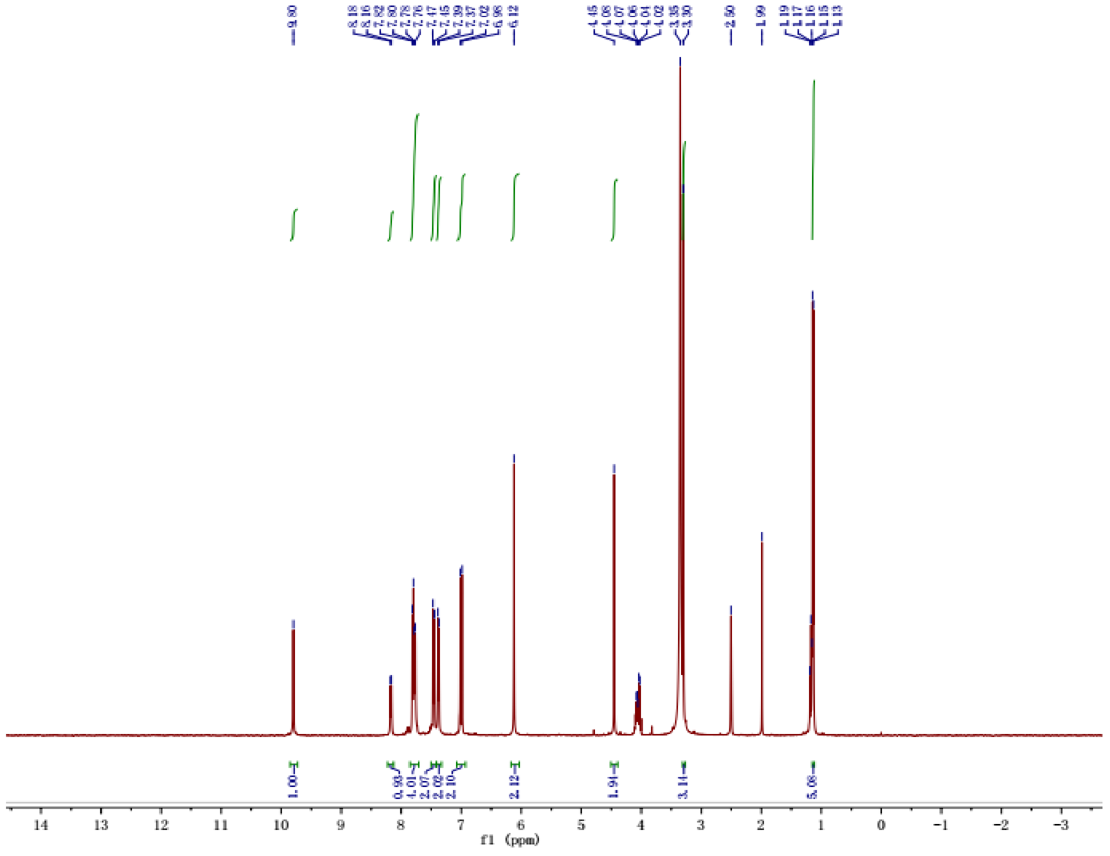

^{1}H-NMR spectrum of **C10**

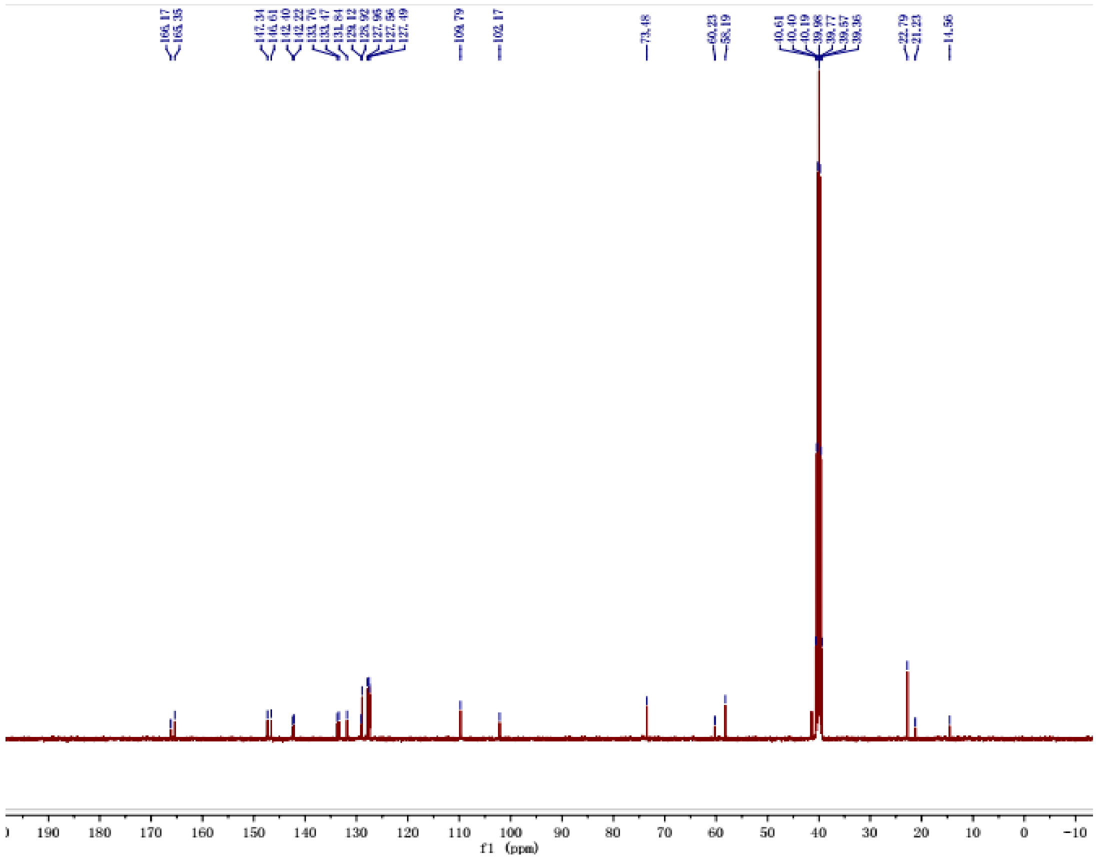

^{13}C-NMR spectrum of **C10**
